# Differential DNA Methylation of the Serotonin Receptor Signaling and Glutamatergic Synapse Pathways in Adult Twins Born Preterm

**DOI:** 10.3390/genes17060683

**Published:** 2026-06-10

**Authors:** Carl Peter Vittrup Rasmussen, Marianne Nygaard, Morten Frost Nielsen, Mette Soerensen, Kaare Christensen, Qihua Tan

**Affiliations:** 1 Epidemiology, Biostatistics and Biodemography, Department of Public Health, Faculty of Health Sciences, University of Southern Denmark, 5230 Odense, Denmark; carra20@student.sdu.dk (C.P.V.R.); mnygaard@health.sdu.dk (M.N.); msoerensen@health.sdu.dk (M.S.); kchristensen@health.sdu.dk (K.C.); 2 Research Unit of Endocrinology, Department of Clinical Research, Faculty of Health Sciences, University of Southern Denmark, 5230 Odense, Denmark; mmfnielsen@health.sdu.dk; 3 Research Unit of Clinical Genetics, Department of Clinical Research, Faculty of Health Sciences, University of Southern Denmark, 5230 Odense, Denmark

**Keywords:** epigenetics, preterm birth, serotonin receptor signaling pathway, glutamatergic synapse pathway

## Abstract

Background/Objectives: Early-life environment may influence long-term neurodevelopment through epigenetic regulation. Serotonergic and glutamatergic pathways are central to brain development and have been implicated in DNA methylation changes following prenatal adversity. In this study, we examined whether preterm birth (PTB) in birthweight-discordant twins is associated with differential DNA methylation in the serotonin receptor signaling pathway and the glutamatergic synapse pathway in adult twins. Methods: Genome-wide DNA methylation data were obtained from whole blood samples of 288 individuals (144 monozygotic birthweight-discordant twin pairs), including a younger cohort (140 individuals; mean age 33 years) and an older cohort (148 individuals; mean age 63 years). DNA methylation was measured using the Illumina HumanMethylation450 BeadChip. Linear models were fitted for association testing, adjusting for leukocyte composition and twin pair correlation. Pathway-level differential methylation was assessed using Rotation Gene Set Testing. Results: In the glutamatergic synapse pathway, no consistent directional enrichment of hypo- or hypermethylation was observed. However, gene-level analyses identified consistent hypomethylation of *GRIA2* and *GRIA4* across cohorts. In the serotonin receptor signaling pathway, the young cohort exhibited a mixed methylation pattern, whereas the old cohort showed significant enrichment of hypermethylation. At the gene level, *HTR1A* was hypomethylated in the young cohort but hypermethylated in the old cohort, indicating a cohort-dependent effect in the methylation patterns. Conclusions: These findings suggest that PTB is associated with long-term epigenetic variation in neurodevelopmentally relevant pathways, as reflected in blood cells. The results further indicate distinct methylation architectures across pathways, with more consistent pathway-level signals in the serotonergic system and more localized gene-level effects in the glutamatergic pathway.

## 1. Introduction

Early-life environmental conditions are increasingly recognized as key determinants of long-term health and disease. Within the Developmental Origins of Health and Disease (DOHaD), adverse prenatal exposures, including nutritional imbalance and maternal stress, may alter developmental trajectories during critical periods [1,2]. Because fetal development is characterized by rapid cellular differentiation and organ formation, environmental perturbation during this period may have lasting biological consequences.

Epigenetic regulation provides a plausible molecular mechanism linking early-life exposures to long-term phenotypic variation. Epigenetics encompasses heritable structural and biochemical modifications of chromatin that occur without changes to the DNA sequence and regulate gene activity by altering chromatin accessibility and transcriptional potential [3]. Among these mechanisms, DNA methylation is the most extensively studied and occurs predominantly at cytosine—phosphate—guanine (CpG) sites by adding a methyl group to the fifth carbon position of a cytosine ring in regulatory regions of the genome [4,5,6]. DNA methylation plays a key role in establishing and maintaining tissue-specific gene expression patterns during development and is particularly sensitive to environmental influences during early life.

Environmental exposures can induce locus-specific changes in DNA methylation that contribute to gene–environment interactions, often affecting genes involved in metabolic regulation, immune function, and stress responsiveness [1,7]. The timing of exposure is critical, as the epigenome undergoes extensive reprogramming during prenatal and early postnatal development, rendering DNA methylation patterns especially susceptible to environmental perturbations. Disruption during these critical windows may result in epigenetic alterations that persist across the life course, whereas exposures later in life tend to have more transient and context-dependent effects [1,2,8].

Human studies support the long-term impact of early-life environmental adversity on DNA methylation. Individuals prenatally exposed to famine exhibit persistent methylation differences detectable decades later, particularly in genes and pathways related to neurodevelopment and synaptic function [9,10]. Similarly, prenatal maternal stress (such as malnutrition, psychological and physiological stress during pregnancy) has been associated with altered DNA methylation in genes involved in stress-response and neurodevelopmental pathways, suggesting that early-life conditions may become biologically embedded through epigenetic mechanisms [2].

Preterm birth (PTB), defined as birth before 37 completed weeks of gestation, represents a clinically relevant example of an early-life condition associated with altered developmental trajectories. Epidemiological evidence shows that, compared with those born at full term, individuals born moderately or late preterm have an increased risk of neurodevelopmental impairments, including motor, cognitive, visual, hearing, and epileptic disorders. These risks follow a gradient across gestational age, with higher risks observed at earlier gestational weeks, indicating that neurodevelopmental outcomes are closely linked to the timing of birth [11].

The neurodevelopmental consequences of PTB are thought to arise partly from the disruption of critical periods of brain development. During late gestation and the early postnatal period, the brain undergoes rapid maturational processes, including neuronal and glial development, synaptogenesis, and early myelination. Birth before term therefore exposes the developing brain to extrauterine conditions during a phase of heightened vulnerability. Consistent with this, large population-based evidence shows that neurodevelopmental risk follows a gradient across gestational age. In a Swedish national cohort of more than 1.2 million singleton children, the risks of motor, cognitive, epileptic, visual, and hearing impairments were highest among children born at 32 weeks and gradually decreased toward full term, with moderately and late preterm children showing higher risks than those born at 39–40 weeks [11].

Although advances in neonatal care have reduced severe focal brain injuries, PTB remains associated with long-term impairments in cognition, motor function, behavior, attention, and executive function, including among children without overt brain injury on conventional neuroimaging [11,12].

Contemporary evidence suggests that these outcomes may reflect more subtle and diffuse alterations in brain structure, connectivity, and cellular maturation. Proposed mechanisms include hypoxia–ischemia, inflammation, and oxidative stress, which can interfere with normal neuronal and glial development, synaptic organization, and network formation [13]. This perspective provides a framework for investigating molecular mechanisms underlying the long-term effects of PTB, including the epigenetic regulation of neurodevelopmentally relevant pathways.

Serotonin and glutamate are central signaling systems in brain development and represent biologically relevant pathways through which early-life environmental conditions may influence neurodevelopmental trajectories. Both systems are active during prenatal and early postnatal periods, where they regulate neuronal proliferation, migration, synapse formation, and circuit maturation [14,15].

Serotonin appears early in embryogenesis and exerts effects before the establishment of mature synaptic transmission. During fetal development, serotonergic signaling regulates neuronal migration, axonal guidance, dendritic growth, and synaptic organization [14,15]. Fetal serotonin availability is influenced not only by embryonic sources but also by placental synthesis from maternal tryptophan, linking serotonergic signaling to maternal environmental conditions such as diet, stress, and immune activation [14]. Alterations in serotonin levels during critical developmental windows can affect cortical circuit formation and plasticity, as well as gene expression in brain regions involved in stress regulation and emotional processing [16,17]. In humans, prenatal famine exposure has been associated with differential DNA methylation in genes belonging to the serotonin receptor signaling pathway [9].

Glutamate is the principal excitatory neurotransmitter in the central nervous system and plays a fundamental role in shaping neural circuits during development. Glutamatergic signaling, mediated through ionotropic and metabotropic receptors, regulates synapse formation, activity-dependent plasticity, and the refinement of neuronal networks [18]. Early-life environmental perturbations have been associated with alterations in glutamatergic transmission and synaptic organization. Experimental studies indicate that nutritional and stress-related exposures can lead to long-term changes in glutamate concentrations and receptor function in brain regions such as the hippocampus, thalamus, and prefrontal cortex [19]. In humans, prenatal famine exposure has been associated with differential DNA methylation in genes involved in the glutamatergic synapse pathway [10].

These systems are highly interconnected during brain development. Serotonergic signaling modulates glutamatergic neurotransmission, influencing excitatory–inhibitory balance and synaptic plasticity [17]. Disruptions in serotonergic activity may therefore have downstream effects on glutamatergic signaling and broader neural network organization [15]. Consistent with this, epigenetic studies of prenatal adversity have identified differential methylation in genes belonging to both pathways [9,10], suggesting that they may jointly contribute to the neurodevelopmental consequences of early-life environmental exposure.

Based on this framework, the present study investigates whether proxy indicators of adverse early-life environment, namely PTB, are associated with differential DNA methylation in neurodevelopmentally relevant pathways. To address this, we utilize monozygotic twins to specifically examine DNA methylation in genes belonging to the serotonin receptor signaling pathway and the glutamatergic synapse pathway. Using genome-wide DNA methylation data, we apply pathway-based statistical approaches to assess coordinated methylation differences across these gene sets in association with PTB. By focusing on biologically defined pathways rather than individual CpG sites, this study aims to provide a more integrated understanding of how early-life environmental variation—captured through differences in gestational timing and fetal growth—relates to long-term epigenetic regulation of neurodevelopment.

## 2. Materials and Methods

### 2.1. Study Population

This study is based on DNA methylation data from monozygotic twins in the Danish Twin Registry, including twin pairs with large intra-pair differences in birthweight. Previous studies in this cohort have investigated long-term metabolic, endocrine, and skeletal outcomes in adult birthweight-discordant monozygotic twins [20,21,22], typically involving pairs with substantial birthweight differences (e.g., a median difference of around 0.5 kg). This design enables investigation of the long-term consequences of early-life differences while controlling for genetic and shared environmental factors. The cohort has also been used to study epigenetic variation associated with early-life exposures [23,24]. The dataset comprised 296 individuals, of whom eight were excluded due to inconsistencies in phenotype information, resulting in a final analytical sample of 288 individuals (144 complete twin pairs). Birthweight information was obtained from midwife records for twins born before 1973 and from the Danish Medical Birth Register for those born from 1973 onward [25]. Individuals born before 1973 were classified as the older cohort (n = 148, PTB = 57), where information on gestational status was provided by midwives categorically as term/preterm, whereas individuals born in or after 1973 were classified as the younger cohort (n = 140), where information on gestational age was recorded in weeks (Table 1), with PTB defined as gestational age < 37 weeks (PTB = 26).

### 2.2. DNA Methylation Data

Genome-wide DNA methylation profiling was performed using the Illumina Infinium HumanMethylation450 BeadChip (450K array) [23,24], which interrogates approximately 485,512 CpG sites across the genome, including promoters, CpG islands, gene bodies, and intergenic regions. DNA methylation levels were quantified as β-values using the Illumina formula,

$$\beta =\frac{M}{\left(M+U+100\right)},$$

where *M* and *U* represent methylated and unmethylated signal intensities, respectively. The normalized *β*-value matrix was used for all downstream analyses.

Quality control and preprocessing were performed as described by Sørensen et al. [24], using the R packages minfi [26] and MethylAid [27]. Samples were excluded if fewer than 95% of probes had a detection *p*-value below 0.01 or if they failed internal quality control metrics based on control probes. Probe-level filtering removed CpGs with detection *p*-values greater than 0.01, zero raw intensity, low bead count (<3 beads), a measurement success rate below 95%, or known cross-reactivity. After filtering, approximately 450,512 CpG sites remained for analysis. Functional normalization was applied to correct for technical variation and batch effects using five principal components [28]. Functional normalization is specifically designed to remove batch-to-batch variation that occurs when processing large-scale genomic datasets. Functional normalization was performed using the *preprocessFunnorm* function implemented in the *minfi* package. Blood cell composition was estimated from the DNA methylation data by the Houseman method implemented in the R package *minfi* [26].

### 2.3. Statistical Analysis

#### 2.3.1. Single-Site-Based Statistical Testing

For statistical modeling, β-values were transformed to M-values using a logit transformation,

M=log(β / (1−β)).

The single-site-based association analyses of CpGs mapped to genes in each biological pathway were conducted using the *limma* framework [29]. Linear models were fitted at each CpG site using *lmFit()*, followed by empirical Bayes moderation with *eBayes()*. DNA methylation (M-values) was modeled as a function of PTB, adjusting for leukocyte composition (i.e., the estimated proportions of leukocyte subtypes: CD4+ T cells, CD8+ T cells, natural killer cells, monocytes, and granulocytes) and intra-pair correlation (by including twin pair IDs as a random factor variable). To limit the number of parameters in the model, birthweight was not included for adjustment because of its non-significant correlation with the methylation of genes in the target pathways. Analyses were performed separately for the younger and older cohorts. Multiple testing correction was performed using the Benjamini–Hochberg procedure [30], and CpGs with a false discovery rate (FDR) q-value < 0.05 were considered statistically significant.

#### 2.3.2. Pathway-Based Statistical Testing

To facilitate biological interpretation, CpGs were mapped to genes within two predefined pathways: the glutamatergic synapse pathway from the Kyoto Encyclopedia of Genes and Genomes (KEGG) database (https://www.genome.jp/dbget-bin/www_bget?pathway+hsa04724) (accessed on 16 January 2026), with pathway ID: hsa04724, and the serotonin receptor signaling pathway from the Molecular Signatures Database (https://www.gsea-msigdb.org/gsea/msigdb/cards/GOBP_SEROTONIN_RECEPTOR_SIGNALING_PATHWAY) (accessed on 16 January 2026), with pathway ID: GO_0007210. All CpGs annotated to genes within each KEGG pathway were retained and reported for each cohort. In total, this included 2500 CpGs mapped to the 114 genes on autosomal chromosomes in the glutamatergic synapse pathway (Supplementary Table S1) and 382 CpGs mapped to the 39 genes on autosomal chromosomes in the serotonin receptor signaling pathway (Supplementary Table S2).

Pathway-level differential methylation was assessed using Rotation Gene Set Testing (ROAST) implemented in *limma* [31]. ROAST accounts for correlation among CpGs and evaluates whether predefined gene sets exhibit coordinated differential methylation [31]. The same design matrices used in the EWAS were applied. For each pathway and cohort, ROAST was used to test directional enrichment for increased methylation (“up”), decreased methylation (“down”), and bidirectional enrichment (“mixed”), where CpGs within the same pathway may show changes in both directions. Specifically, a “mixed” enrichment pattern indicates that a pathway contains significant differentially methylated CpGs, but these CpGs are methylated in both directions; some are hypermethylated and others are hypomethylated. The “Up-or-Down” test is also reported and evaluates evidence for differential methylation in either direction. The active proportion represents the estimated fraction of CpGs in the pathway contributing to the enrichment signal.

## 3. Results

### 3.1. PTB-Associated Regulation of the Glutamatergic Pathway

An overview of the descriptive characteristics of the study population is provided in Table 1.

In the young cohort (N = 140), no evidence of coordinated directional methylation changes was observed within the glutamatergic synapse pathway. Neither hypomethylation (active proportion = 16.6%, *p* = 0.801) nor hypermethylation (active proportion = 22.7%, *p* = 0.198) was significantly enriched, and the Up-or-Down test was also non-significant (*p* = 0.397). In contrast, a significant mixed pattern of differential methylation was detected (active proportion = 39.4%, *p* = 5.0 × 10^−4^), indicating bidirectional methylation changes across CpGs within the pathway.

**Figure 1:**
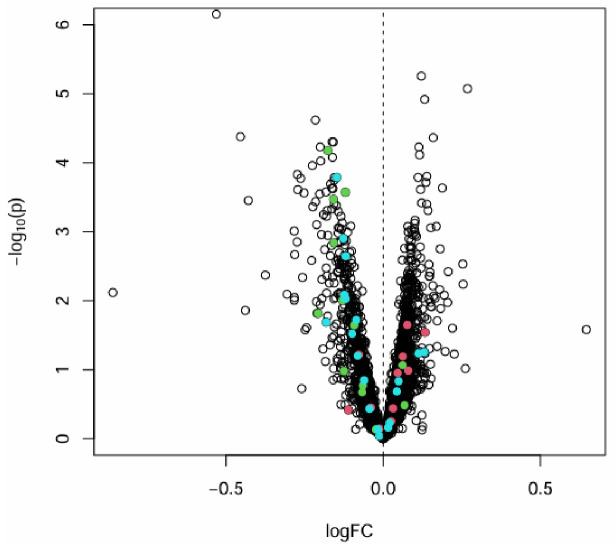
Volcano plot of CpGs mapped to genes within the glutamatergic synapse pathway in the young cohort. The *x*-axis represents the estimated effect size (logFC of M-values), and the *y*-axis shows the corresponding −log10(*p*-value) from the empirical Bayes-moderated t-statistics. CpGs mapped to AMPA receptor subunit genes (*GRIA1*, *GRIA2*, and *GRIA4*) are highlighted in red, green and blue, respectively.

This pattern is reflected in Figure 1 based on single-site association testing, where CpGs are distributed on both sides of zero effect size, indicating bidirectional methylation changes rather than a uniform directional effect. CpGs mapped to AMPA receptor subunit genes (*GRIA1*, *GRIA2*, and GRIA4) show a similar distribution, although several *GRIA2* and *GRIA4* CpGs are located on the negative logFC side, suggesting a tendency towards hypomethylation at the gene level. Gene-level analyses highlighted consistent hypomethylation in *GRIA2* and *GRIA4*. For *GRIA2*, hypomethylation was strongly enriched (active proportion = 61.5%, *p* = 5.0 × 10^−4^), with no evidence of hypermethylation (active proportion = 7.7%, *p* = 0.9997). *GRIA4* showed a similar pattern (active proportion = 52.6%, *p* = 0.0025 for hypomethylation; hypermethylation *p* = 0.9977). In contrast, *GRIA1* did not exhibit directional enrichment but showed a significant mixed pattern (active proportion = 50.0%, *p* = 0.0155), indicating heterogeneous methylation changes across CpGs within the gene.

**Figure 2:**
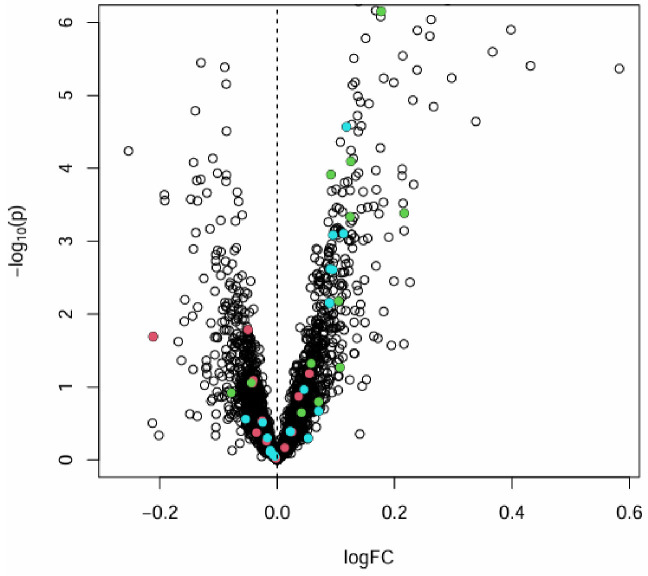
Volcano plot of CpGs mapped to genes within the glutamatergic synapse pathway in the older cohort. Effect sizes (logFC of M-values) are shown on the *x*-axis and −log10(*p*-values) on the *y*-axis. CpGs are spread across both positive and negative effect sizes, forming a broadly symmetric distribution, with several points reaching moderate statistical significance on either side. CpGs belonging to AMPA receptor subunit genes (*GRIA1*, *GRIA2*, and *GRIA4*) are highlighted in red, green and blue, respectively.

A similar pathway-level pattern was observed in the older cohort (N = 148), with no significant enrichment of hypomethylation (active proportion = 13.4%, *p* = 0.855) or hypermethylation (active proportion = 19.4%, *p* = 0.145), and a non-significant Up-or-Down test (*p* = 0.240). However, the mixed test remained significant (active proportion = 32.4%, *p* = 0.002), indicating bidirectional methylation changes. Figure 2 shows a symmetric distribution of CpGs around zero effect size, consistent with these findings.

Gene-level patterns were largely consistent with the young cohort. *GRIA2* again showed strong hypomethylation (active proportion = 61.5%, *p* = 5.0 × 10^−4^), and *GRIA4* displayed similar enrichment (active proportion = 52.6%, *p* = 0.0053). *GRIA1* remained non-directional, with a significant mixed pattern (active proportion = 50.0%, *p* = 0.0195).

Results of the ROAST analysis for the glutamatergic pathway are summarized in Table 2. Detailed results from the single-CpG analysis are provided in Supplementary Tables S3 and S4 for the young and old cohorts, respectively.

### 3.2. PTB-Associated Regulation of the Serotonin Receptor Signaling Pathway

In the young cohort (N = 140), no directional enrichment of DNA methylation was observed within the serotonin receptor signaling pathway. Neither hypomethylation (active proportion = 24.2%, *p* = 0.602) nor hypermethylation (active proportion = 25.2%, *p* = 0.398) was significant, and the Up-or-Down test was non-significant (*p* = 0.796). In contrast, a significant mixed pattern of differential methylation was detected (active proportion = 49.4%, *p* = 0.001), indicating substantial bidirectional methylation changes across CpGs.

**Figure 3:**
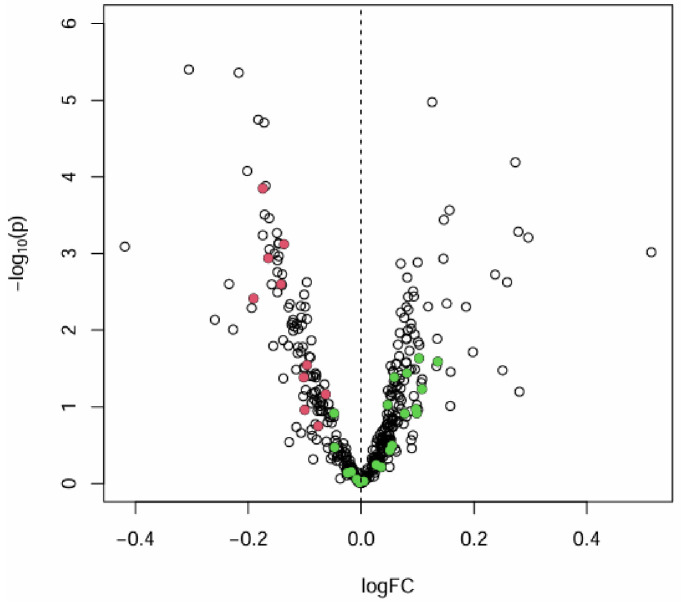
Volcano plot of CpGs mapped to genes within the serotonin receptor signaling pathway in the young cohort. The *x*-axis represents effect sizes (logFC of M-values), and the *y*-axis shows −log10(*p*-values). CpGs mapped to specific genes are highlighted in colors (*HTR1A* (red) and *HTR2A* (green)).

Figure 3 illustrates this distribution, with CpGs spanning both positive and negative effect sizes. CpGs mapped to *HTR1A* and *HTR2A* are distributed across both directions, consistent with the pathway-level findings.

At the gene level, *HTR1A* showed a strong enrichment of hypomethylation (active proportion = 90.0%, *p* = 2.5 × 10^−4^), whereas *HTR2A* showed no evidence of coordinated differential methylation and remained non-directional.

**Figure 4:**
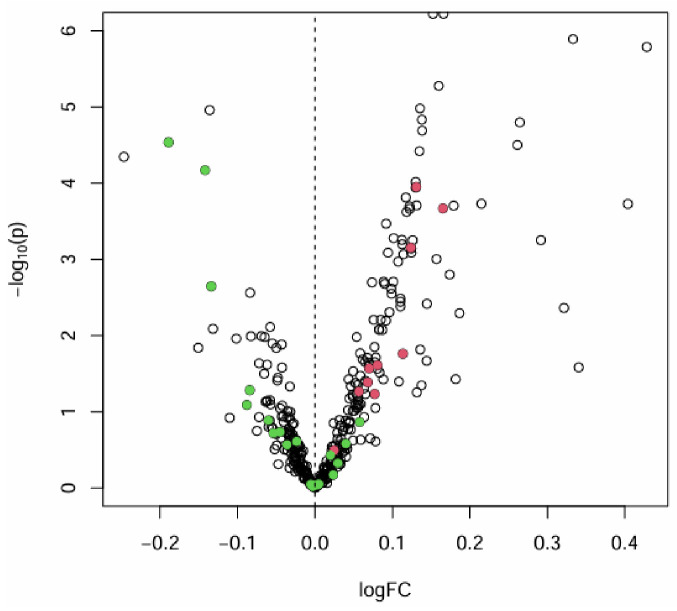
Volcano plot of CpGs mapped to genes within the serotonin receptor signaling pathway in the older cohort. The *x*-axis represents effect sizes (logFC of M-values), and the *y*-axis shows −log10(*p*-values). CpGs mapped to specific genes are highlighted in colors (*HTR1A* (red) and *HTR2A* (green)).

In contrast, the older cohort (N = 148) exhibited a clear predominance of hypermethylation. Significant enrichment of hypermethylated CpGs was observed (active proportion = 29.7%, *p* = 7.0 × 10^−4^), along with significant Up-or-Down (*p* = 0.001) and mixed (*p* = 5.0 × 10^−4^) results. Figure 4 shows a corresponding shift toward positive logFC values. CpGs mapped to *HTR1A* and *HTR2A* are predominantly located in the positive region.

Gene-level analysis showed a consistent pattern of hypermethylation for *HTR1A* (active proportion = 90.0%, *p* = 2.5 × 10^−4^), whereas *HTR2A* remained non-directional, displaying no coordinated enrichment.

ROAST results for the serotonin receptor signaling pathway are summarized in Table 3. The results for all CpGs mapped to the serotonin receptor signaling pathway are provided in Supplementary Tables S5 and S6 for the young and old cohorts, respectively.

## 4. Discussion

The present study investigated differential DNA methylation in the glutamatergic synapse and serotonin receptor signaling pathways across the young and the old cohorts. Both pathways showed evidence of epigenetic variation, but with distinct and cohort-dependent patterns. These findings, although based on blood cells, could reflect the long-term effects of PTB on neurodevelopmentally relevant pathways.

The glutamatergic synapse pathway showed no consistent directional enrichment across cohorts. Instead, both the young and old cohorts exhibited significant mixed patterns of methylation. This indicates widespread differential methylation without a coordinated shift in one direction. Biologically, the significance of the mixed patterns suggests that PTB-associated epigenetic regulation of the glutamatergic synapse pathway involves both up- and down-regulation of different genes in the pathway under different biological and environmental conditions. Previous studies have shown that early-life exposures can alter glutamatergic signaling, including long-term changes in glutamate levels in animal models [19] and hypomethylation of glutamatergic genes following prenatal famine exposure in humans [10]. In contrast, the present results suggest that pathway-level effects are heterogeneous. However, consistent hypomethylation of *GRIA2* and *GRIA4* across cohorts indicates that specific components of the pathway may be particularly sensitive to early-life perturbation.

The serotonin receptor signaling pathway showed a different pattern. The young cohort exhibited mixed methylation, whereas the old cohort showed significant enrichment of hypermethylation. This contrasts with previous reports of hypomethylation following prenatal famine exposure [9] and suggests that methylation patterns in this pathway may depend on cohort characteristics or the timing of measurement. Although the observed difference could be potentially due to age-related change in DNA methylation [32,33], our cross-sectional study design does not rule out the involvement of other factors. Alternatively, the observed differences may reflect birth cohort effects, as the young and old groups represent individuals exposed to different environmental, clinical, and societal conditions. These factors may independently influence epigenetic patterns and complicate the interpretation of longitudinal effects.

These results are consistent with the developmental origins framework, in which early-life conditions such as PTB influence long-term biological regulation. PTB disrupts late gestational brain development, including processes relevant to synaptic signaling, and is associated with an increased risk of neurodevelopmental impairment. The methylation patterns observed may therefore reflect persistent molecular effects of early-life disruption. At the same time, the variability across pathways and cohorts indicates that these effects are not uniform and may depend on developmental timing and context.

Since the study population consisted of twins, the extent to which the findings can be generalized to singletons should be interpreted carefully. Twins differ from singletons in several early-life characteristics, including a greater likelihood of preterm delivery and lower birthweight. Because earlier delivery is relatively common in twin pregnancies, PTB among twins may not reflect the same underlying phenotype as PTB in singleton pregnancies. The observed methylation patterns should therefore primarily be understood within the context of early-life development in twins [34].

A recent case–control study reported preterm birth as a risk factor for disorders of gut–brain interaction in adults, including functional gastroduodenal disorders, bowel disorders, and constipation [35]. It known that about 90% of the body’s serotonin is produced in the gut, and serotonin serves as a critical biomarker of the gut–brain axis [36], acting as a neurotransmitter in the brain and a paracrine signal in the gut. Moreover, there is epidemiological evidence linking PTB with gastric acid-related disorders in adulthood [37]. Since the target tissue for our DNA methylation analysis was whole blood rather than the brain, the detected patterns could also possibly reflect differential epigenetic regulation of the serotonin receptor signaling pathway in the gastrointestinal system. Tissue-specific DNA methylation profiling could help with more precise inference on the PTB-associated differential epigenetic regulation of the serotonin receptor signaling pathway in specific organs and its relevant functional consequences.

From a clinical perspective, alterations in glutamatergic and serotonin receptor signaling pathways are relevant, as both systems play central roles in neurodevelopment and are implicated in psychiatric outcomes following early-life adversity [9,10]. This is especially important for the twin population given the high prevalence of PTB in twin pregnancies. PTB accounts for over 60% of all twin births, and the risk of premature delivery is more than five times higher than that in singletons [38]. According to our results, adult twins are biologically predisposed to neurodevelopmental disorders, implying that individualized care should be taken for prevention, early diagnosis and treatment. The same could be generalized to adult PTB singletons upon further validation in the general population.

In addition to preterm birth, DNA methylation patterns are influenced by a range of factors across the lifespan, including environmental exposures, lifestyle factors, comorbidities, and medication use. These influences may interact with early-life programming effects and contribute to the observed heterogeneity in methylation patterns.

Several limitations should be considered. The use of the Illumina 450K array introduces bias in probe coverage, which may influence pathway-level analyses [39,40]. DNA methylation was measured in whole blood rather than brain tissue. Although blood-based methylation has been shown to capture aspects of brain-related variation [41], the results of blood-derived DNA might not fully reflect brain-specific methylation changes. Finally, multiple birth-related factors, such as placental factors, fetal growth restriction, birth era differences in neonatal management practices and gestational age recording methods, were not considered in the analysis, either due to a lack of data or due to statistical power considerations. Extra caution is needed when interpreting our findings.

In conclusion, the findings suggest that preterm birth may be associated with long-term epigenetic changes in pathways that are important for neurodevelopment. However, the patterns are not consistent across pathways or cohorts. The glutamatergic pathway showed a more mixed pattern, while the serotonin receptor signaling pathway appeared more structured and cohort-dependent. This indicates that the correlation between pathway regulation and early-life perturbation is complex and may vary depending on both the biological system and the timing of exposure. At the same time, the differences between cohorts suggest that later-life factors may also play a role. Future studies could explore whether similar patterns are observed in individuals born more preterm, as well as in larger study populations, and ideally include longitudinal data and clinical outcomes to better understand the relevance of these findings.

## Figures and Tables

**Table 1:** Descriptive statistics for the study samples.

	All Twins	Young Twins	Old Twins
Age, Years *			
Min	30	30	57
Max	76	38	76
Mean	50	33	64
**Sex**			
Female	140	64	76
Male	148	76	72
**Born Preterm**	83	26	57
**Gestational age, Weeks**			
Min		33	
Max		42	
Mean		38	

* Young twins were born after 1973, and old twins were born before 1973. Gestational age was not measured in weeks and was only available after 1973 through the Danish Medical Birth Registry.

**Table 2:** Gene set test results for the glutamatergic synapse pathway in the young and old cohorts.

Cohort/Pattern	Active Proportion	*p*-Value
**Young twins**		
Down	0.166	0.801
Up	0.227	0.198
UpOrDown	0.227	0.397
Mixed	0.394	0.0005
**Old twins**		
Down	0.134	0.855
Up	0.194	0.145
UpOrDown	0.194	0.240
Mixed	0.324	0.002

**Table 3:** Gene set test results for the serotonin signaling pathway in the young and old cohorts.

Cohort/Pattern	Active Proportion	*p*-Value
**Young twins**		
Down	0.242	0.602
Up	0.252	0.398
UpOrDown	0.252	0.796
Mixed	0.494	0.001
**Old twins**		
Down	0.116	0.999
Up	0.297	0.0007
UpOrDown	0.297	0.001
Mixed	0.414	0.0005

## Data Availability

Raw DNA methylation data have been deposited to the NCBI GEO database http://www.ncbi.nlm.nih.gov/geo/ (accessed on 16 January 2026) under accession number GSE61496.

## Supplementary Materials

The following supporting information can be downloaded at: https://www.mdpi.com/article/10.3390/genes17060683/s1; Table S1. Annotation of CpGs mapped to genes of the glutamatergic synapse pathway. Table S2. Annotation of CpGs mapped to genes of the serotonin receptor signaling pathway. Table S3. All CpG sites mapped to genes within the glutamatergic synapse pathway in the young cohort, including genomic annotation and results from epigenome-wide association analysis. Table S4. All CpG sites mapped to genes within the glutamatergic synapse pathway in the old cohort, including genomic annotation and results from single-site-based association analysis. Table S5. All CpG sites mapped to genes within the serotonin receptor signaling pathway in the young cohort, including genomic annotation and results from single-site-based association analysis. Table S6. All CpG sites mapped to genes within the serotonin receptor signaling pathway in the old cohort, including genomic annotation and results from single-site-based association analysis.
