# Supplementary material for: Differential DNA Methylation of the Serotonin Receptor Signaling and Glutamatergic Synapse Pathways in Adult Twins Born Preterm
Source: Genes (Basel). 2026 Jun 10;17(6):683. doi: 10.3390/genes17060683 (PMC13299586; doi:10.3390/genes17060683)
Supplement: Supplementary file 1 [file genes-17-00683-s001.zip › Supplementary captions.pdf]

Supplementary captions

Table S1. Annotation of CpGs mapped to genes of the glutamatergic synapse pathway.

Table S2. Annotation of CpGs mapped to genes of the serotonin receptor signaling pathway.

Table S3. All CpG sites mapped to genes within the glutamatergic synapse pathway in the young cohort, including genomic annotation and results from single-site-based association analysis.

Table S4. All CpG sites mapped to genes within the glutamatergic synapse pathway in the old cohort, including genomic annotation and results from single-site-based association analysis.

Table S5. All CpG sites mapped to genes within the serotonin receptor signaling pathway in the young cohort, including genomic annotation and results from single-site-based association analysis.

Table S6. All CpG sites mapped to genes within the serotonin receptor signaling pathway in the old cohort, including genomic annotation and results from single-site-based association analysis.
