# Supplementary material for: Differential DNA Methylation of the Serotonin Receptor Signaling and Glutamatergic Synapse Pathways in Adult Twins Born Preterm
Source: Genes (Basel). 2026 Jun 10;17(6):683. doi: 10.3390/genes17060683 (PMC13299586; doi:10.3390/genes17060683)
Supplement: Supplementary file 1 [file genes-17-00683-s001.zip › Supplementary Table S1 glutamate.pdf]

CpG	Chromosome	Position	Gene	CpG_Island_Context
cg10529401	chr1	53587046	SLC1A7	OpenSea
cg22291265	chr19	51182808	SHANK1	OpenSea
cg05112254	chr12	2224410	CACNA1C	
cg08098950	chr16	4033226	ADCY9	S_Shelf
cg00582971	chr5	178422128	GRM6	Island
cg06247406	chr6	101846791	GRIK2	
cg26351229	chr8	132053780	ADCY8	Island
cg00439658	chr17	72848669	GRIN2C	Island
cg11439695	chr12	2561024	CACNA1C	
cg08475096	chr4	158143750	GRIA2	
cg07456682	chr5	178414540	GRM6	N_Shore
cg22762091	chr8	132052843	ADCY8	Island
cg01281157	chr5	178422260	GRM6	Island
cg04138502	chr3	123167522	ADCY5	Island
cg07628416	chr16	4033297	ADCY9	S_Shelf
cg25909396	chr17	64300729	PRKCA	S_Shore
cg11308643	chr11	105480788	GRIA4	
cg01637841	chr3	7610381	GRM7	
cg12124094	chr17	64433744	PRKCA	OpenSea
cg01577933	chr1	37498025	GRIK3	N_Shore
cg24301620	chr6	101846872	GRIK2	
cg09108394	chr16	23850106	PRKCB	
cg10591607	chr6	101846916	GRIK2	
cg25649039	chr17	64335475	PRKCA	OpenSea
cg13515021	chr12	49177075	ADCY6	
cg01942127	chr3	53529259	CACNA1D	
cg15021670	chr15	42386726	PLA2G4D	
cg07365960	chr17	72848535	GRIN2C	Island
cg13483026	chr3	123167973	ADCY5	Island
cg15425280	chr4	158141492	GRIA2	
cg24213507	chr3	6902689	GRM7	
cg17605476	chr4	158143917	GRIA2	
cg14010550	chr19	1009642	GRIN3B	Island
cg14287235	chr14	24804339	ADCY4	Island
cg11844965	chr19	42510093	GRIK5	Island
cg14082127	chr6	101847349	GRIK2	
cg15519474	chr17	64300902	PRKCA	S_Shore
cg14859460	chr5	178422244	GRM6	Island
cg03451029	chr7	79764387	GNAI1	
cg00082310	chr3	171528573	PLD1	
cg01250212	chr19	2650755	GNG7	Island
cg04837533	chr12	26986864	ITPR2	S_Shore
cg21187352	chr3	6903327	GRM7	
cg26332560	chr8	132052887	ADCY8	Island
cg15335139	chr3	50242325	SLC38A3	N_Shore
cg02332525	chr3	6903153	GRM7	
cg16812352	chr16	4049855	ADCY9	OpenSea

cg08292023	chr11	88242253	GRM5	
cg07100700	chr6	34059787	GRM4	OpenSea
cg17627654	chr11	70508410	SHANK2	
cg08379637	chr21	31311387	GRIK1	
cg01622416	chr21	31310508	GRIK1	
cg27182923	chr3	123129387	ADCY5	OpenSea
cg04711050	chr9	4490757	SLC1A1	Island
cg15066837	chr7	79775261	GNAI1	OpenSea
cg20172500	chr3	6902432	GRM7	
cg22490420	chr15	42434040	PLA2G4F	
cg24342051	chr3	53527884	CACNA1D	
cg11640185	chr19	42509801	GRIK5	N_Shore
cg12934382	chr3	51741135	GRM2	
cg27428129	chr6	34051095	GRM4	OpenSea
cg26430597	chr1	182354699	GLUL	
cg05115424	chr17	64787379	PRKCA	OpenSea
cg19646112	chr14	24804342	ADCY4	Island
cg19657814	chr19	47137444	GNG8	N_Shore
cg20326410	chr1	53600821	SLC1A7	OpenSea
cg02140020	chr12	2613914	CACNA1C	
cg03663215	chr5	7396491	ADCY2	Island
cg25856632	chr17	7111551	DLG4	
cg09147777	chr11	105480771	GRIA4	
cg19237476	chr11	70501603	SHANK2	
cg23475371	chr21	31311842	GRIK1	
cg02110273	chr17	64727684	PRKCA	OpenSea
cg01245966	chr8	132053823	ADCY8	Island
cg27199820	chr3	6903019	GRM7	
cg22541254	chr6	101846779	GRIK2	
cg16415870	chr3	6904261	GRM7	
cg22597733	chr4	158142891	GRIA2	
cg02928699	chr11	88241946	GRM5	
cg01737026	chr6	101847656	GRIK2	
cg14214834	chr3	123139305	ADCY5	OpenSea
cg13757263	chr3	53807593	CACNA1D	
cg02504690	chr11	70319385	SHANK2	
cg03499570	chr11	70424529	SHANK2	
cg06099971	chr15	42386816	PLA2G4D	OpenSea
cg24863175	chr1	186840433	PLA2G4A	OpenSea
cg15228639	chr15	42289939	PLA2G4E	OpenSea
cg22231400	chr19	49935823	SLC17A7	Island
cg01352090	chr16	4103533	ADCY9	OpenSea
cg08550394	chr16	4013659	ADCY9	N_Shore
cg21816539	chr21	31312328	GRIK1	
cg17450585	chr6	34059718	GRM4	OpenSea
cg09434500	chr19	42502897	GRIK5	Island
cg13861180	chr9	140060268	GRIN1	
cg20640499	chr6	101850881	GRIK2	

cg03120091	chr8	132052779	ADCY8	
cg23676682	chr11	105480792	GRIA4	
cg07950000	chr21	31312333	GRIK1	
cg02194717	chr11	70415188	SHANK2	
cg25942860	chr5	178410055	GRM6	N_Shelf
cg03149432	chr1	37498721	GRIK3	Island
cg02155655	chr1	53566481	SLC1A7	OpenSea
cg15993383	chr3	123167507	ADCY5	Island
cg01596520	chr19	14225029	PRKACA	
cg13373757	chr12	2263558	CACNA1C	
cg23894980	chr11	70534718	SHANK2	OpenSea
cg20787196	chr12	26987031	ITPR2	S_Shore
cg18193094	chr6	101846905	GRIK2	
cg03764381	chr16	10275410	GRIN2A	
cg04907257	chr5	7395318	ADCY2	Island
cg03966406	chr12	26985412	ITPR2	N_Shore
cg11836949	chr1	1812359	GNB1	OpenSea
cg19809667	chr19	54401945	PRKCG	Island
cg14462402	chr11	120678447	GRIK4	OpenSea
cg18760587	chr7	79764888	GNAI1	Island
cg17199007	chr6	146349527	GRM1	
cg01722994	chr16	10277317	GRIN2A	
cg19914607	chr3	50242505	SLC38A3	N_Shore
cg23970331	chr6	33656237	ITPR3	Island
cg13286510	chr7	126893007	GRM8	Island
cg07060551	chr19	51198381	SHANK1	Island
cg02515133	chr7	86415687	GRM3	Island
cg22851944	chr6	101847388	GRIK2	
cg04695635	chr19	42510823	GRIK5	S_Shore
cg16761581	chr14	24803807	ADCY4	Island
cg21806090	chr12	2198034	CACNA1C	
cg03923850	chr12	2372169	CACNA1C	
cg19606462	chr5	7827964	ADCY2	OpenSea
cg26316946	chr6	101846967	GRIK2	
cg14872036	chr3	123049031	ADCY5	OpenSea
cg15562780	chr11	35441311	SLC1A2	Island
cg05346491	chr19	48917104	GRIN2D	N_Shore
cg05031016	chr14	24804153	ADCY4	Island
cg07312654	chr8	132053773	ADCY8	Island
cg19008133	chr3	123124015	ADCY5	OpenSea
cg24634422	chr11	35441593	SLC1A2	Island
cg19507068	chr7	79764176	GNAI1	
cg20626645	chr3	53528846	CACNA1D	
cg15705536	chr5	7825292	ADCY2	OpenSea
cg06722633	chr1	37499309	GRIK3	Island
cg09300795	chr16	4042428	ADCY9	OpenSea
cg13537240	chr12	2761549	CACNA1C	
cg09096555	chr17	72848358	GRIN2C	Island

cg12350325	chr12	2800909	CACNA1C	
cg03508063	chr17	7124385	DLG4	
cg24755189	chr11	62475373	BSCL2	
cg19196684	chr1	53608037	SLC1A7	OpenSea
cg03437186	chr7	45614848	ADCY1	Island
cg00076497	chr7	126891621	GRM8	Island
cg00834536	chr16	4013537	ADCY9	N_Shore
cg27369641	chr7	100274361	GNB2	S_Shore
cg07699277	chr6	34004226	GRM4	Island
cg05942459	chr6	101846805	GRIK2	
cg02837591	chr6	101850261	GRIK2	
cg10793758	chr22	51133417	SHANK3	N_Shelf
cg26536401	chr12	6956432	GNB3	OpenSea
cg06829391	chr16	9857151	GRIN2A	
cg27092975	chr11	70805455	SHANK2	OpenSea
cg24849633	chr22	51142900	SHANK3	Island
cg00664406	chr3	51740875	GRM2	
cg08958294	chr6	146350131	GRM1	
cg03503785	chr16	23962572	PRKCB	
cg17567700	chr22	51112218	SHANK3	Island
cg19707326	chr14	24787611	ADCY4	S_Shore
cg02309655	chr19	2588629	GNG7	S_Shore
cg23566401	chr17	7120484	DLG4	
cg09432792	chr16	56352311	GNAO1	
cg12228229	chr17	7122261	DLG4	
cg03991309	chr1	68237761	GNG12	OpenSea
cg03562531	chr3	53764604	CACNA1D	
cg26393354	chr11	70713937	SHANK2	OpenSea
cg23942984	chr12	14103087	GRIN2B	OpenSea
cg04583232	chr11	22362874	SLC17A6	Island
cg04149773	chr12	49179923	ADCY6	N_Shelf
cg23519329	chr16	4166914	ADCY9	Island
cg22989419	chr20	9340396	PLCB4	
cg06466348	chr16	50337922	ADCY7	OpenSea
cg23797439	chr20	8113355	PLCB1	
cg26564874	chr5	178416134	GRM6	Island
cg23321702	chr6	34031060	GRM4	OpenSea
cg02203881	chr15	42386909	PLA2G4D	OpenSea
cg00553487	chr19	42570406	GRIK5	OpenSea
cg06193383	chr16	10275767	GRIN2A	
cg25727569	chr3	53845287	CACNA1D	
cg09461286	chr16	10276081	GRIN2A	
cg11479156	chr11	70672388	SHANK2	N_Shore
cg05488168	chr19	13400637	CACNA1A	
cg06328100	chr6	33638806	ITPR3	OpenSea
cg14111697	chr9	80462928	GNAQ	OpenSea
cg13536060	chr19	51189671	SHANK1	N_Shore
cg00808175	chr12	6949119	GNB3	OpenSea

cg25702790	chr7	79765394	GNAI1	S_Shore
cg23159970	chr12	2690385	CACNA1C	
cg00518386	chr16	10276984	GRIN2A	
cg22891619	chr17	72839038	GRIN2C	Island
cg03132806	chr5	178414179	GRM6	N_Shore
cg27555529	chr19	13617518	CACNA1A	
cg03091752	chr19	51221605	SHANK1	Island
cg10806318	chr11	70374297	SHANK2	
cg26746936	chr19	42503392	GRIK5	Island
cg07232612	chr7	93551012	GNG11	OpenSea
cg25852492	chr15	42140150	JMJD7-PLA2G4B	
cg10286380	chr19	51171847	SHANK1	Island
cg13510813	chr19	42571339	GRIK5	OpenSea
cg15603568	chr11	105481283	GRIA4	
cg25001544	chr6	34073788	GRM4	OpenSea
cg14263118	chr20	57463787	GNAS	
cg11544138	chr19	1003455	GRIN3B	Island
cg15651980	chr19	48903304	GRIN2D	S_Shore
cg13555101	chr9	4490751	SLC1A1	Island
cg27178677	chr20	8834803	PLCB1	
cg18799510	chr9	104499700	GRIN3A	N_Shore
cg27073262	chr7	86493792	GRM3	OpenSea
cg08997253	chr9	104500729	GRIN3A	
cg04527363	chr3	6902337	GRM7	
cg04336164	chr2	68478630	PPP3R1	N_Shore
cg04396791	chr11	70508180	SHANK2	
cg09305491	chr16	24151191	PRKCB	
cg10409919	chr3	53530016	CACNA1D	
cg14093720	chr18	3712400	DLGAP1	
cg14019146	chr3	50243930	SLC38A3	S_Shore
cg13384396	chr3	123167677	ADCY5	Island
cg27333271	chr3	7268498	GRM7	
cg16086007	chr17	72855588	GRIN2C	N_Shore
cg08955995	chr19	42503412	GRIK5	Island
cg08310216	chr7	100271217	GNB2	Island
cg01663725	chr12	14133829	GRIN2B	N_Shore
cg10904109	chr6	146755494	GRM1	
cg21217024	chr11	105481406	GRIA4	
cg20132775	chr3	142444202	TRPC1	S_Shore
cg20459126	chr3	142443247	TRPC1	Island
cg18842187	chr6	33647826	ITPR3	OpenSea
cg02807849	chr19	48908102	GRIN2D	N_Shore
cg20882260	chr12	2374427	CACNA1C	
cg25744767	chr7	79764178	GNAI1	
cg25148589	chr4	158141936	GRIA2	
cg03403991	chr22	51167187	SHANK3	N_Shore
cg16359985	chr7	100276087	GNB2	S_Shelf
cg09106984	chr6	34004360	GRM4	S_Shore

cg22491927	chr19	13617091	CACNA1A	
cg13878010	chr3	123167276	ADCY5	Island
cg15828915	chr12	26801163	ITPR2	OpenSea
cg21563683	chr12	46767928	SLC38A2	S_Shore
cg26780231	chr17	64468338	PRKCA	OpenSea
cg00426968	chr19	47138284	GNG8	N_Shore
cg14926715	chr11	70318919	SHANK2	
cg14753385	chr11	70476422	SHANK2	
cg09354294	chr1	68188118	GNG12	OpenSea
cg19032532	chr19	2547067	GNG7	S_Shore
cg17483510	chr3	179168677	GNB4	N_Shore
cg19063061	chr19	49935893	SLC17A7	Island
cg14483383	chr11	64022763	PLCB3	N_Shelf
cg19965023	chr17	72838366	GRIN2C	N_Shore
cg04453050	chr3	51740896	GRM2	
cg02551234	chr11	64023126	PLCB3	N_Shelf
cg24275501	chr12	2198070	CACNA1C	
cg18391758	chr16	10274963	GRIN2A	
cg24611631	chr9	4490288	SLC1A1	Island
cg20018057	chr20	57465139	GNAS	
cg25407736	chr1	68296179	GNG12	N_Shelf
cg06421614	chr17	7121116	DLG4	
cg14530764	chr3	123124018	ADCY5	OpenSea
cg12377578	chr17	72856181	GRIN2C	Island
cg18356448	chr18	3881547	DLGAP1	S_Shore
cg13203394	chr12	26951217	ITPR2	OpenSea
cg03856723	chr19	14229466	PRKACA	S_Shore
cg20871277	chr6	33656548	ITPR3	S_Shore
cg10262891	chr19	48904928	GRIN2D	N_Shelf
cg15805568	chr19	51199000	SHANK1	Island
cg07309764	chr7	79763914	GNAI1	Island
cg14724613	chr7	86273429	GRM3	
cg02303571	chr5	36606769	SLC1A3	
cg12682032	chr15	83617937	HOMER2	
cg04537738	chr22	51143999	SHANK3	S_Shore
cg01207684	chr16	4103167	ADCY9	OpenSea
cg03193168	chr22	51159995	SHANK3	Island
cg01780685	chr17	7099875	DLG4	
cg24607686	chr2	191827930	GLS	OpenSea
cg06954761	chr6	33601863	ITPR3	S_Shore
cg04023483	chr3	6904134	GRM7	
cg14036830	chr19	42503207	GRIK5	Island
cg22689690	chr12	49183468	ADCY6	S_Shore
cg18175690	chr15	40580770	PLCB2	N_Shelf
cg21734356	chr18	3498854	DLGAP1	
cg12191293	chr12	56882314	GLS2	Island
cg07716032	chr17	7122846	DLG4	
cg09408768	chr2	155555053	KCNJ3	Island

cg13570585	chr20	8113573	PLCB1	
cg22198397	chr19	15067457	SLC1A6	OpenSea
cg27644513	chr15	42281679	PLA2G4E	OpenSea
cg17540575	chr19	42504627	GRIK5	S_Shore
cg10648542	chr5	178416050	GRM6	Island
cg13448720	chr9	140052246	GRIN1	
cg03153115	chr19	2604559	GNG7	N_Shelf
cg12709244	chr17	7123282	DLG4	
cg00699993	chr4	158141570	GRIA2	
cg17401938	chr12	2228442	CACNA1C	
cg18707858	chr19	13366101	CACNA1A	
cg04510788	chr1	37498900	GRIK3	Island
cg15995075	chr3	142451487	TRPC1	OpenSea
cg11855555	chr1	68232134	GNG12	OpenSea
cg10123654	chr16	4162541	ADCY9	N_Shelf
cg16341159	chr17	7121370	DLG4	
cg03070741	chr19	2650727	GNG7	Island
cg23183497	chr7	86273718	GRM3	
cg09965996	chr16	56390429	GNAO1	OpenSea
cg12887832	chr11	70805627	SHANK2	OpenSea
cg17754876	chr11	35441260	SLC1A2	Island
cg12778476	chr11	22359345	SLC17A6	N_Shelf
cg17871403	chr5	7827115	ADCY2	OpenSea
cg12496211	chr12	2193060	CACNA1C	
cg16696270	chr8	132052934	ADCY8	Island
cg20790998	chr1	68290436	GNG12	OpenSea
cg24159247	chr3	4575483	ITPR1	
cg25693099	chr18	3879303	DLGAP1	Island
cg26555126	chr6	33998729	GRM4	S_Shelf
cg05716556	chr17	47287410	ABI3	
cg16378117	chr16	9857804	GRIN2A	
cg05616819	chr16	24231485	PRKCB	
cg01975093	chr11	62474759	BSCL2	
cg20771332	chr11	70332620	SHANK2	
cg10614021	chr12	14134486	GRIN2B	N_Shore
cg18872881	chr4	102199803	PPP3CA	
cg09143713	chr20	9141615	PLCB4	
cg04569608	chr11	64018309	PLCB3	N_Shore
cg22920586	chr3	171472629	PLD1	
cg15706539	chr11	70924914	SHANK2	OpenSea
cg07254421	chr5	36657993	SLC1A3	
cg04590790	chr5	7770690	ADCY2	OpenSea
cg17604429	chr5	7827133	ADCY2	OpenSea
cg10509626	chr11	70333993	SHANK2	
cg16848712	chr12	46767747	SLC38A2	S_Shore
cg03489495	chr6	33588875	ITPR3	Island
cg14679587	chr12	56882324	GLS2	Island
cg20073686	chr11	105481863	GRIA4	

cg24539500	chr6	102115051	GRIK2	
cg13722123	chr6	146350346	GRM1	
cg23715749	chr1	37413867	GRIK3	OpenSea
cg19228334	chr6	101851283	GRIK2	
cg24082826	chr12	26985738	ITPR2	
cg17742947	chr19	42546977	GRIK5	S_Shore
cg13891121	chr12	26987045	ITPR2	S_Shore
cg24620508	chr21	31310605	GRIK1	
cg00146655	chr3	7517194	GRM7	
cg24868359	chr21	31312535	GRIK1	
cg16803737	chr6	33592658	ITPR3	S_Shelf
cg20818778	chr1	235814145	GNG4	
cg03225817	chr11	105481317	GRIA4	
cg10774282	chr1	53608280	SLC1A7	
cg07906046	chr16	4131584	ADCY9	OpenSea
cg15331781	chr7	86274443	GRM3	OpenSea
cg02693486	chr11	64030862	PLCB3	N_Shelf
cg06068039	chr6	34031208	GRM4	OpenSea
cg24643102	chr3	6903921	GRM7	
cg00392377	chr19	49939882	SLC17A7	Island
cg06844159	chr11	70374355	SHANK2	
cg27630678	chr11	70565201	SHANK2	S_Shore
cg19755318	chr3	50243323	SLC38A3	Island
cg27335600	chr3	53528857	CACNA1D	
cg09465746	chr3	6904386	GRM7	
cg27027803	chr20	57464742	GNAS	
cg05410012	chr17	72857095	GRIN2C	Island
cg17921248	chr17	64298993	PRKCA	Island
cg12389770	chr6	101847706	GRIK2	
cg17987968	chr5	152869882	GRIA1	
cg23734973	chr4	158141449	GRIA2	
cg24445167	chr12	2383231	CACNA1C	
cg23310850	chr19	19051337	HOMER3	
cg27181295	chr19	2511475	GNG7	Island
cg01132471	chr1	53556482	SLC1A7	N_Shore
cg25397191	chr19	14224992	PRKACA	
cg17214089	chr1	182354912	GLUL	
cg11300838	chr19	19049950	HOMER3	
cg03972076	chr11	64023183	PLCB3	N_Shelf
cg25557995	chr12	2761091	CACNA1C	
cg00303541	chr3	51741280	GRM2	
cg15436476	chr19	2626283	GNG7	S_Shelf
cg20227471	chr2	25065550	ADCY3	OpenSea
cg20684528	chr12	14133667	GRIN2B	N_Shore
cg11284196	chr19	51190047	SHANK1	Island
cg15174564	chr11	120856801	GRIK4	Island
cg13709913	chr9	104334427	GRIN3A	OpenSea
cg10271186	chr11	70908897	SHANK2	OpenSea

cg02882755	chr6	34100963	GRM4	OpenSea
cg14468634	chr5	78758952	HOMER1	OpenSea
cg03777288	chr12	13717033	GRIN2B	Island
cg17298751	chr11	22363370	SLC17A6	Island
cg25217317	chr1	235811994	GNG4	
cg01962826	chr6	34100967	GRM4	OpenSea
cg21496785	chr5	178420865	GRM6	N_Shore
cg14301531	chr12	2226907	CACNA1C	
cg02774856	chr19	19052293	HOMER3	
cg16222802	chr3	50295474	GNAI2	
cg12265829	chr14	24804022	ADCY4	Island
cg06371583	chr19	2581343	GNG7	S_Shore
cg02471325	chr15	42290555	PLA2G4E	OpenSea
cg01806181	chr19	1007843	GRIN3B	Island
cg08429705	chr19	2583601	GNG7	S_Shelf
cg05659265	chr16	56225846	LOC283856	
cg19234705	chr19	19042181	HOMER3	
cg05736079	chr9	140063205	GRIN1	
cg10943398	chr11	70319250	SHANK2	
cg09963080	chr16	4017270	ADCY9	S_Shore
cg03100801	chr20	9075962	PLCB4	
cg26875958	chr6	146751590	GRM1	
cg01793368	chr11	64022905	PLCB3	N_Shelf
cg09146232	chr17	64672129	PRKCA	OpenSea
cg01348055	chr16	10272788	GRIN2A	
cg22632947	chr17	64787784	PRKCA	OpenSea
cg17799599	chr17	64787605	PRKCA	OpenSea
cg25988118	chr6	34101785	GRM4	OpenSea
cg09327847	chr16	24087793	PRKCB	
cg04884798	chr14	24791720	ADCY4	OpenSea
cg04209460	chr17	4711018	PLD2	S_Shore
cg09662638	chr3	53795946	CACNA1D	
cg05850280	chr9	140056489	GRIN1	
cg13583454	chr5	153038077	GRIA1	
cg10785385	chr22	51114364	SHANK3	S_Shore
cg17767099	chr19	1009048	GRIN3B	Island
cg07735790	chr6	34101545	GRM4	OpenSea
cg21006600	chr12	2342206	CACNA1C	
cg02484455	chr11	70559534	SHANK2	OpenSea
cg23559689	chr11	105481292	GRIA4	
cg09640070	chr12	26876374	ITPR2	OpenSea
cg00110790	chr6	33655814	ITPR3	N_Shore
cg24454829	chr11	22363053	SLC17A6	Island
cg05484458	chr12	6949260	GNB3	OpenSea
cg21895324	chr11	35385435	SLC1A2	OpenSea
cg17168836	chr1	68256161	GNG12	OpenSea
cg09598225	chr20	57466839	GNAS	
cg05509359	chr11	70432884	SHANK2	

cg22187630	chr19	13616871	CACNA1A	
cg21323244	chr11	70415439	SHANK2	
cg13724160	chr9	104500958	GRIN3A	Island
cg21245981	chr5	36607390	SLC1A3	
cg16999602	chr1	53608163	SLC1A7	
cg19274368	chr11	70331491	SHANK2	
cg24874003	chr19	2602614	GNG7	OpenSea
cg01081636	chr6	33994263	GRM4	N_Shore
cg08236022	chr7	93551014	GNG11	OpenSea
cg15884992	chr6	34028192	GRM4	S_Shelf
cg12198934	chr17	64378035	PRKCA	OpenSea
cg26626089	chr19	54385865	PRKCG	S_Shore
cg08586541	chr19	51198888	SHANK1	Island
cg26381514	chr12	26963489	ITPR2	OpenSea
cg09973502	chr12	46766012	SLC38A2	Island
cg26350373	chr15	42449015	PLA2G4F	
cg02569086	chr12	26985672	ITPR2	Island
cg13907146	chr3	50243565	SLC38A3	Island
cg08258650	chr11	35441900	SLC1A2	S_Shore
cg10546626	chr20	57424521	GNASAS	
cg21201396	chr11	70665271	SHANK2	N_Shore
cg07642043	chr16	10276674	GRIN2A	
cg22941646	chr1	235814339	GNG4	
cg01427575	chr19	51171712	SHANK1	Island
cg01542384	chr3	50284305	GNAI2	
cg22953407	chr5	178408081	GRM6	OpenSea
cg04158792	chr19	2514622	GNG7	S_Shore
cg19915762	chr11	64023086	PLCB3	N_Shelf
cg16684939	chr7	100272223	GNB2	Island
cg20490197	chr6	34000298	GRM4	N_Shelf
cg20875807	chr15	83620951	HOMER2	
cg02640558	chr8	22299141	PPP3CC	Island
cg05460776	chr16	4031231	ADCY9	S_Shore
cg15164708	chr19	49936274	SLC17A7	Island
cg03578926	chr11	70508032	SHANK2	
cg09407429	chr3	4534383	ITPR1	
cg10013716	chr3	179168760	GNB4	Island
cg11833293	chr11	70557519	SHANK2	OpenSea
cg02399044	chr12	2500229	CACNA1C	
cg26562691	chr16	23850404	PRKCB	
cg20342105	chr11	62474910	BSCL2	
cg03760316	chr18	3594197	DLGAP1	
cg04364463	chr1	37498270	GRIK3	N_Shore
cg05068686	chr11	70419186	SHANK2	
cg13896105	chr12	2304473	CACNA1C	
cg15338449	chr15	83620910	HOMER2	
cg09649610	chr1	235814039	GNG4	
cg06047881	chr20	57465132	GNAS	

cg12502079	chr22	51169028	SHANK3	Island
cg03485674	chr16	50347895	ADCY7	OpenSea
cg03029664	chr17	72840306	GRIN2C	S_Shore
cg24428099	chr2	25065702	ADCY3	OpenSea
cg16177440	chr7	100275304	GNB2	S_Shore
cg13936125	chr16	56225599	LOC283856	
cg00140112	chr18	3879595	DLGAP1	Island
cg00683984	chr7	45615337	ADCY1	Island
cg26640467	chr7	126893304	GRM8	Island
cg01291761	chr12	14017080	GRIN2B	OpenSea
cg14291291	chr6	33656083	ITPR3	Island
cg24454695	chr1	235814326	GNG4	
cg04743945	chr7	86273058	GRM3	OpenSea
cg02380914	chr22	51143114	SHANK3	Island
cg20837354	chr12	2398146	CACNA1C	
cg15008401	chr4	102267974	PPP3CA	
cg05926269	chr20	57463906	GNAS	
cg05725666	chr12	2224644	CACNA1C	
cg07678592	chr12	49178406	ADCY6	
cg00150025	chr15	42448079	PLA2G4F	
cg16135716	chr12	14133887	GRIN2B	N_Shore
cg10341242	chr16	50347849	ADCY7	OpenSea
cg08218799	chr14	24804930	ADCY4	S_Shore
cg14869721	chr2	25065924	ADCY3	OpenSea
cg13763339	chr11	70516627	SHANK2	OpenSea
cg02585344	chr16	10276092	GRIN2A	
cg21858376	chr3	4534791	ITPR1	
cg26268742	chr19	48563560	PLA2G4C	
cg02011392	chr6	101847541	GRIK2	
cg13401531	chr11	70333281	SHANK2	
cg24764979	chr16	10276600	GRIN2A	
cg07417708	chr5	78809348	HOMER1	
cg17960347	chr12	2457373	CACNA1C	
cg22175856	chr19	15084302	SLC1A6	OpenSea
cg12581769	chr19	13412999	CACNA1A	
cg23369234	chr12	2511478	CACNA1C	
cg14061491	chr9	80433462	GNAQ	OpenSea
cg10590857	chr5	7397021	ADCY2	Island
cg12647801	chr11	64028732	PLCB3	S_Shore
cg10362475	chr11	70507825	SHANK2	
cg20955817	chr11	70936560	SHANK2	OpenSea
cg25839745	chr4	101969341	PPP3CA	
cg02224372	chr11	70858695	SHANK2	OpenSea
cg20877313	chr12	56881753	GLS2	Island
cg14101380	chr11	70718575	SHANK2	OpenSea
cg07366462	chr3	123162899	ADCY5	N_Shelf
cg10106561	chr2	25050913	ADCY3	OpenSea
cg05793288	chr15	42375586	PLA2G4D	S_Shelf

cg27279652	chr12	26986506	ITPR2	S_Shore
cg19200285	chr12	2800755	CACNA1C	
cg14351692	chr12	13716374	GRIN2B	Island
cg00226831	chr15	42371511	PLA2G4D	N_Shore
cg04894216	chr7	86377879	GRM3	OpenSea
cg14696064	chr3	123010055	ADCY5	OpenSea
cg22639787	chr20	57464973	GNAS	
cg07813421	chr17	7123626	DLG4	
cg18011401	chr19	13617366	CACNA1A	
cg02218200	chr22	51135138	SHANK3	N_Shore
cg18827756	chr15	42130735	JMJD7-PLA2G4B	
cg17006443	chr11	70628938	SHANK2	OpenSea
cg04505435	chr11	70672511	SHANK2	N_Shore
cg06736148	chr15	52416833	GNB5	
cg05846851	chr16	10172054	GRIN2A	
cg14537332	chr11	70508113	SHANK2	
cg23766996	chr7	86272023	GRM3	OpenSea
cg03645007	chr3	50255295	SLC38A3	OpenSea
cg15334006	chr15	42449916	PLA2G4F	
cg27222147	chr12	2224755	CACNA1C	
cg14829063	chr11	70731587	SHANK2	OpenSea
cg15134033	chr16	10274415	GRIN2A	
cg26213368	chr11	62474978	BSCL2	
cg13180375	chr8	22298119	PPP3CC	Island
cg11169463	chr6	33653411	ITPR3	N_Shelf
cg16753846	chr11	70318894	SHANK2	
cg05282459	chr22	51117157	SHANK3	S_Shelf
cg06628693	chr1	84543156	PRKACB	
cg24693368	chr2	155554844	KCNJ3	Island
cg04270835	chr11	22359188	SLC17A6	N_Shelf
cg00350942	chr9	140034073	GRIN1	
cg25960567	chr12	26985181	ITPR2	N_Shore
cg21899500	chr3	51740850	GRM2	
cg19471040	chr6	34031142	GRM4	OpenSea
cg01980361	chr3	53843939	CACNA1D	
cg22518097	chr4	101948183	PPP3CA	
cg20151098	chr6	33995991	GRM4	Island
cg26160180	chr1	1822883	GNB1	Island
cg21725265	chr19	19051201	HOMER3	
cg18558423	chr6	33653506	ITPR3	N_Shelf
cg17721618	chr15	42376692	PLA2G4D	OpenSea
cg05060704	chr3	50275694	GNAI2	S_Shore
cg23832825	chr11	70842453	SHANK2	OpenSea
cg12089094	chr3	171463962	PLD1	
cg03827772	chr11	70912450	SHANK2	OpenSea
cg14397813	chr9	80522508	GNAQ	OpenSea
cg24497686	chr1	53606574	SLC1A7	OpenSea
cg16168199	chr17	7123990	DLG4	

cg15014684	chr17	7093301	DLG4	
cg27368776	chr11	105793986	GRIA4	
cg00341980	chr1	84544220	PRKACB	
cg03654598	chr19	51202141	SHANK1	S_Shelf
cg16142824	chr5	178413721	GRM6	Island
cg10425005	chr16	10133433	GRIN2A	
cg27371466	chr6	34003640	GRM4	Island
cg25134567	chr17	64782369	PRKCA	
cg23068476	chr12	2613703	CACNA1C	
cg00898486	chr6	34006557	GRM4	S_Shelf
cg07190947	chr11	70349808	SHANK2	
cg18920858	chr17	64617748	PRKCA	OpenSea
cg27484541	chr20	57461542	GNAS	
cg08875948	chr6	146755900	GRM1	
cg24098927	chr7	86273180	GRM3	OpenSea
cg05082609	chr11	105692831	GRIA4	
cg03412431	chr6	34023418	GRM4	N_Shore
cg11871050	chr15	52477794	GNB5	OpenSea
cg08461840	chr19	2620967	GNG7	N_Shore
cg11444428	chr16	850802	GNG13	Island
cg12013817	chr6	34008384	GRM4	OpenSea
cg22900607	chr19	2546938	GNG7	Island
cg08478447	chr3	53528849	CACNA1D	
cg16512895	chr19	13410117	CACNA1A	
cg24891846	chr19	13319523	CACNA1A	
cg06310285	chr3	53796066	CACNA1D	
cg10024799	chr12	2641381	CACNA1C	
cg26419941	chr12	49183133	ADCY6	Island
cg07855933	chr19	13349725	CACNA1A	
cg16079774	chr11	22364498	SLC17A6	S_Shore
cg06419562	chr16	24148747	PRKCB	
cg07824914	chr20	57465815	GNAS	
cg07192048	chr11	70560211	SHANK2	N_Shelf
cg17020834	chr5	152870258	GRIA1	
cg09428623	chr2	68480128	PPP3R1	S_Shore
cg00444360	chr11	88270612	GRM5	
cg08170375	chr3	50247180	SLC38A3	S_Shelf
cg20741386	chr1	53556189	SLC1A7	N_Shelf
cg26282150	chr1	110091625	GNAI3	S_Shore
cg21072025	chr19	47137863	GNG8	Island
cg17107112	chr6	34021546	GRM4	N_Shelf
cg24363298	chr3	50242671	SLC38A3	Island
cg12150066	chr1	1823305	GNB1	S_Shore
cg07533824	chr19	13363864	CACNA1A	
cg12754421	chr11	105480790	GRIA4	
cg22971402	chr6	33993822	GRM4	N_Shelf
cg00287829	chr16	10276375	GRIN2A	
cg22125912	chr3	171428263	PLD1	

cg26914334	chr11	120561236	GRIK4	OpenSea
cg22454005	chr19	54392916	PRKCG	N_Shore
cg04955246	chr17	64720790	PRKCA	OpenSea
cg20801637	chr1	1795408	GNB1	OpenSea
cg19694519	chr8	22389206	PPP3CC	OpenSea
cg03412547	chr16	4057728	ADCY9	OpenSea
cg07633435	chr5	152869009	GRIA1	
cg19033906	chr3	53532972	CACNA1D	
cg12012319	chr7	126675528	GRM8	
cg04929165	chr1	186807235	PLA2G4A	OpenSea
cg25961618	chr11	35360531	SLC1A2	OpenSea
cg11803392	chr17	64712432	PRKCA	OpenSea
cg01814344	chr5	178413313	GRM6	Island
cg09134640	chr1	37337877	GRIK3	OpenSea
cg14597908	chr20	57414960	GNASAS	
cg08037774	chr1	53608962	SLC1A7	OpenSea
cg09050775	chr12	46762708	SLC38A2	N_Shelf
cg26472511	chr16	4034240	ADCY9	OpenSea
cg04521626	chr17	4714200	PLD2	S_Shelf
cg00586732	chr3	7755472	GRM7	
cg04658038	chr17	64800166	PRKCA	OpenSea
cg12544392	chr19	42544587	GRIK5	N_Shelf
cg17642145	chr8	22298578	PPP3CC	Island
cg25000623	chr17	72848918	GRIN2C	S_Shore
cg03078593	chr12	26789311	ITPR2	OpenSea
cg14235271	chr20	57462812	GNAS	
cg27541048	chr12	6954816	GNB3	OpenSea
cg07284407	chr20	57429858	GNAS	
cg19942459	chr11	70508110	SHANK2	
cg08809418	chr12	56881865	GLS2	Island
cg02035425	chr9	140062632	GRIN1	
cg00701890	chr16	4102293	ADCY9	OpenSea
cg21844450	chr20	8112956	PLCB1	
cg19385628	chr3	6904640	GRM7	
cg10956480	chr12	2338945	CACNA1C	
cg06872721	chr19	19040258	HOMER3	
cg06758670	chr16	10276383	GRIN2A	
cg24801123	chr7	45615503	ADCY1	Island
cg02327001	chr4	101972762	PPP3CA	
cg14653281	chr9	104500954	GRIN3A	Island
cg03896542	chr16	56378687	GNAO1	
cg02937055	chr3	171489625	PLD1	
cg04440551	chr2	25051151	ADCY3	OpenSea
cg25803139	chr19	2539626	GNG7	N_Shore
cg04119977	chr5	7826972	ADCY2	OpenSea
cg17509967	chr19	13617094	CACNA1A	
cg12933359	chr7	86416314	GRM3	S_Shore
cg11306735	chr19	49943812	SLC17A7	N_Shore

cg15704369	chr1	84543558	PRKACB	
cg13135654	chr11	70842116	SHANK2	OpenSea
cg19622911	chr18	3771570	DLGAP1	
cg14919164	chr7	45729156	ADCY1	OpenSea
cg09031790	chr16	24129948	PRKCB	
cg21518089	chr11	22362708	SLC17A6	N_Shore
cg00161247	chr9	140060986	GRIN1	
cg08685096	chr21	31312643	GRIK1	
cg20950146	chr5	7827524	ADCY2	OpenSea
cg26801014	chr11	88241769	GRM5	
cg20315739	chr15	42303032	PLA2G4E	OpenSea
cg09436713	chr12	2323135	CACNA1C	
cg19621460	chr19	14225945	PRKACA	
cg26985666	chr11	35441088	SLC1A2	
cg19727439	chr1	37500508	GRIK3	Island
cg03047070	chr12	2787827	CACNA1C	
cg07636145	chr16	56228188	DKFZP434H168	
cg06432462	chr9	80646879	GNAQ	Island
cg03650282	chr17	64451448	PRKCA	OpenSea
cg15527678	chr12	49171898	ADCY6	
cg03324851	chr7	100274414	GNB2	S_Shore
cg16468914	chr3	50242735	SLC38A3	
cg22620614	chr11	70794709	SHANK2	OpenSea
cg11025960	chr3	51749188	GRM2	
cg02306526	chr12	2202821	CACNA1C	
cg12691534	chr3	50275394	GNAI2	Island
cg15329866	chr3	171455826	PLD1	
cg13856573	chr11	62475078	BSCL2	
cg11046772	chr12	2353479	CACNA1C	
cg02066343	chr6	33590458	ITPR3	S_Shore
cg20103018	chr6	33996522	GRM4	S_Shore
cg15122327	chr20	57435146	GNAS	
cg00036258	chr16	4029218	ADCY9	Island
cg21931938	chr15	40600493	PLCB2	OpenSea
cg04576607	chr1	235787279	GNG4	
cg04496615	chr2	68479620	PPP3R1	
cg15245951	chr3	50283471	GNAI2	
cg23475045	chr3	7248510	GRM7	
cg26334023	chr17	47287492	ABI3	
cg01091261	chr16	4029363	ADCY9	Island
cg05340882	chr19	2543750	GNG7	Island
cg06112910	chr6	33995939	GRM4	Island
cg02295678	chr17	7123415	DLG4	
cg13298384	chr19	42546648	GRIK5	Island
cg04525943	chr19	2579529	GNG7	Island
cg05362860	chr16	50320692	ADCY7	OpenSea
cg08325885	chr8	22297108	PPP3CC	N_Shore
cg23232299	chr1	68212170	GNG12	OpenSea

cg03425609	chr12	6952374	GNB3	OpenSea
cg22849059	chr12	56882693	GLS2	Island
cg00587834	chr3	51749959	GRM2	
cg16128363	chr18	3880558	DLGAP1	S_Shore
cg09448677	chr11	70672740	SHANK2	N_Shore
cg02738298	chr3	51749852	GRM2	
cg23911372	chr19	42546731	GRIK5	Island
cg27066052	chr16	4015761	ADCY9	N_Shore
cg08289346	chr6	34089350	GRM4	OpenSea
cg15852446	chr15	40583422	PLCB2	Island
cg26229990	chr14	24801301	ADCY4	N_Shore
cg07340423	chr15	52472383	GNB5	
cg07823688	chr16	56310091	GNAO1	
cg18851100	chr22	51158550	SHANK3	Island
cg22967080	chr14	52333365	GNG2	OpenSea
cg02105211	chr3	4625188	ITPR1	
cg16777106	chr4	158281194	GRIA2	
cg02026498	chr19	47139338	GNG8	Island
cg08576827	chr20	9075493	PLCB4	
cg04926767	chr11	62476194	GNG3	
cg09190579	chr7	79763888	GNAI1	Island
cg04016326	chr12	14132940	GRIN2B	
cg04348872	chr2	25141696	ADCY3	N_Shore
cg26621408	chr7	100275863	GNB2	S_Shore
cg06996175	chr19	2546877	GNG7	Island
cg11281320	chr11	120553251	GRIK4	OpenSea
cg16677144	chr1	1790217	GNB1	OpenSea
cg01538522	chr20	57463974	GNAS	
cg20646491	chr5	36608769	SLC1A3	
cg16312514	chr11	70650521	SHANK2	OpenSea
cg07217499	chr12	2416339	CACNA1C	
cg07287255	chr16	56374688	GNAO1	
cg03861217	chr2	155652401	KCNJ3	OpenSea
cg22798925	chr20	57464129	GNAS	
cg04763994	chr19	42506259	GRIK5	N_Shelf
cg12046677	chr12	2360663	CACNA1C	
cg08599266	chr2	25142473	ADCY3	Island
cg00866976	chr16	56224782	GNAO1	
cg25885280	chr11	70760166	SHANK2	OpenSea
cg08480458	chr3	53529655	CACNA1D	
cg01231108	chr2	155556016	KCNJ3	Island
cg27418217	chr15	83518427	HOMER2	
cg20110535	chr19	14225647	PRKACA	
cg26726141	chr17	64612159	PRKCA	OpenSea
cg03300177	chr16	56390811	GNAO1	OpenSea
cg07036561	chr15	42118869	JMJD7	
cg18247436	chr11	120856646	GRIK4	N_Shore
cg04062190	chr7	86413438	GRM3	N_Shelf

cg23159236	chr20	57464002	GNAS	
cg10668781	chr12	2307325	CACNA1C	
cg23153707	chr17	7121040	DLG4	
cg03723730	chr6	34031694	GRM4	OpenSea
cg01364969	chr16	56389029	GNAO1	OpenSea
cg03319638	chr15	42387193	PLA2G4D	OpenSea
cg24276988	chr20	57463106	GNAS	
cg10804438	chr3	51747196	GRM2	
cg11971789	chr19	42545623	GRIK5	N_Shore
cg13327846	chr15	52472389	GNB5	
cg02598335	chr7	86337918	GRM3	OpenSea
cg07176385	chr5	7397756	ADCY2	S_Shore
cg06512271	chr5	7394927	ADCY2	N_Shore
cg06828043	chr11	70398862	SHANK2	
cg10453337	chr5	7502014	ADCY2	OpenSea
cg09830455	chr11	70886197	SHANK2	OpenSea
cg20090108	chr11	88242488	GRM5	
cg21213853	chr3	51741473	GRM2	
cg16623098	chr16	56374383	GNAO1	
cg15417641	chr3	53700141	CACNA1D	
cg16543027	chr15	40599680	PLCB2	OpenSea
cg00147943	chr1	68225713	GNG12	OpenSea
cg09927287	chr15	42447989	PLA2G4F	
cg10698424	chr9	114423570	GNG10	
cg24250393	chr16	23846838	PRKCB	
cg22804770	chr12	2786316	CACNA1C	
cg27179693	chr11	120530818	GRIK4	OpenSea
cg17826344	chr19	51169660	SHANK1	Island
cg20008140	chr20	57463455	GNAS	
cg08471319	chr2	25141735	ADCY3	N_Shore
cg06439941	chr7	93550756	GNG11	OpenSea
cg00369202	chr6	33989844	GRM4	OpenSea
cg23501962	chr11	35440252	SLC1A2	N_Shore
cg09655520	chr17	64786064	PRKCA	OpenSea
cg04993286	chr16	4027595	ADCY9	Island
cg22374237	chr7	126891197	GRM8	N_Shore
cg14654306	chr1	186798107	PLA2G4A	
cg15674997	chr5	178421314	GRM6	Island
cg17800870	chr1	182362757	GLUL	
cg00110769	chr1	110122089	GNAI3	OpenSea
cg13647052	chr12	2800382	CACNA1C	
cg08535918	chr16	56256748	GNAO1	
cg22758916	chr15	42303292	PLA2G4E	OpenSea
cg12913957	chr6	33589131	ITPR3	Island
cg11797430	chr12	2761364	CACNA1C	
cg21146273	chr5	152869193	GRIA1	
cg27074174	chr6	101847318	GRIK2	
cg24043604	chr12	2613948	CACNA1C	

cg00521993	chr10	75199411	PPP3CB	
cg03109047	chr9	140032686	GRIN1	
cg10410142	chr4	158141542	GRIA2	
cg05029189	chr3	123168386	ADCY5	Island
cg06025216	chr16	4164891	ADCY9	Island
cg18845797	chr5	7794275	ADCY2	OpenSea
cg00160359	chr12	49178192	ADCY6	
cg03938110	chr21	31120485	NCRNA00110	
cg03527683	chr7	126752816	GRM8	
cg00515755	chr19	1005248	GRIN3B	Island
cg16701848	chr19	2588479	GNG7	Island
cg02441543	chr22	51157530	SHANK3	N_Shore
cg14870792	chr12	2163532	CACNA1C	
cg16069986	chr11	70650456	SHANK2	OpenSea
cg20439283	chr5	178411249	GRM6	N_Shelf
cg08458678	chr11	70565177	SHANK2	S_Shore
cg16564940	chr17	7117310	DLG4	
cg17329110	chr11	70708812	SHANK2	OpenSea
cg05666036	chr6	101848236	GRIK2	
cg18411150	chr19	51172144	SHANK1	S_Shore
cg00317626	chr1	53600741	SLC1A7	OpenSea
cg05432017	chr15	42119684	JMJD7	
cg16261581	chr1	84972327	GNG5	
cg04002822	chr12	2483094	CACNA1C	
cg06728579	chr16	56224901	GNAO1	
cg23165500	chr5	152949095	GRIA1	
cg23409374	chr19	49934742	SLC17A7	Island
cg27431037	chr12	2330387	CACNA1C	
cg23580000	chr16	50322156	ADCY7	OpenSea
cg26863600	chr19	2616921	GNG7	S_Shelf
cg14643330	chr3	4534051	ITPR1	
cg25185429	chr3	4739461	ITPR1	
cg19256368	chr15	83619037	HOMER2	
cg08288223	chr11	70563131	SHANK2	N_Shore
cg13343565	chr17	4710032	PLD2	Island
cg01833890	chr12	2564063	CACNA1C	
cg22147917	chr6	33656031	ITPR3	Island
cg27012424	chr18	3773224	DLGAP1	
cg15056189	chr12	49176428	ADCY6	
cg09772382	chr20	57463775	GNAS	
cg00020720	chr1	84972482	SPATA1	
cg06517489	chr11	22359333	SLC17A6	N_Shelf
cg10371483	chr16	4152045	ADCY9	OpenSea
cg24902435	chr12	2790095	CACNA1C	
cg13370485	chr12	2762840	CACNA1C	
cg20809470	chr11	22364837	SLC17A6	S_Shore
cg19930620	chr3	7340148	GRM7	
cg05876496	chr6	33638550	ITPR3	OpenSea

cg14728235	chr20	57415177	GNASAS	
cg26912314	chr16	56291781	GNAO1	
cg15728692	chr22	51135736	SHANK3	Island
cg01866630	chr6	33601722	ITPR3	Island
cg21269738	chr12	2356703	CACNA1C	
cg15486374	chr15	83621710	HOMER2	
cg07166235	chr12	49183018	ADCY6	N_Shore
cg24287125	chr12	2692308	CACNA1C	
cg01118752	chr4	102264326	PPP3CA	
cg20557935	chr15	52484322	GNB5	OpenSea
cg13599596	chr9	4541807	SLC1A1	OpenSea
cg24719827	chr1	110091174	GNAI3	Island
cg22600443	chr15	42388240	PLA2G4D	OpenSea
cg22746789	chr19	2579221	GNG7	Island
cg24944109	chr11	35440136	SLC1A2	N_Shore
cg22960869	chr17	47287521	ABI3	
cg22060073	chr8	132052942	ADCY8	Island
cg05492714	chr9	140042286	GRIN1	
cg21370856	chr16	23848003	PRKCB	
cg01895482	chr19	2556145	GNG7	Island
cg00788521	chr12	2229269	CACNA1C	
cg03821543	chr20	57463925	GNAS	
cg19747632	chr15	52472703	GNB5	
cg06996976	chr17	64576105	PRKCA	OpenSea
cg04029168	chr16	23963538	PRKCB	
cg03466124	chr3	179168156	GNB4	N_Shore
cg26459372	chr7	45613676	ADCY1	Island
cg02218260	chr3	51742878	GRM2	
cg07386190	chr1	182361453	GLUL	
cg09067029	chr1	186954259	PLA2G4A	OpenSea
cg00390253	chr3	50241300	SLC38A3	N_Shore
cg24603235	chr6	33588219	ITPR3	N_Shore
cg03173525	chr7	100273221	GNB2	Island
cg07178968	chr15	42130662	JMJD7-PLA2G4B	
cg17658854	chr20	57462798	GNAS	
cg23494413	chr12	6954534	GNB3	OpenSea
cg04156464	chr16	56224504	GNAO1	
cg20582984	chr20	57417233	GNASAS	
cg23202253	chr12	26902211	ITPR2	OpenSea
cg03885818	chr12	2299830	CACNA1C	
cg07091154	chr11	70562728	SHANK2	N_Shore
cg10037905	chr12	26986999	ITPR2	S_Shore
cg10738479	chr19	1000105	GRIN3B	N_Shore
cg15425921	chr19	2643085	GNG7	N_Shore
cg26337841	chr1	37329331	GRIK3	OpenSea
cg20917920	chr11	70416238	SHANK2	
cg25976563	chr3	179169592	GNB4	Island
cg05806233	chr7	79763748	GNAI1	N_Shore

cg10748817	chr20	57465175	GNAS	
cg17106653	chr19	48897279	GRIN2D	S_Shore
cg17125585	chr1	68176471	GNG12	OpenSea
cg17006204	chr11	70448210	SHANK2	
cg02624701	chr19	49937176	SLC17A7	Island
cg13802605	chr9	4495359	SLC1A1	S_Shelf
cg07121488	chr15	40581105	PLCB2	N_Shore
cg16480969	chr6	33996203	GRM4	S_Shore
cg07267600	chr12	2750053	CACNA1C	
cg03773989	chr10	75255862	PPP3CB	
cg10159951	chr11	35441881	SLC1A2	Island
cg05248742	chr16	24142386	PRKCB	
cg02984142	chr1	84971681	SPATA1	
cg09991710	chr1	37472559	GRIK3	OpenSea
cg24319825	chr3	4534939	ITPR1	
cg12467435	chr17	64651126	PRKCA	OpenSea
cg18389339	chr18	3730593	DLGAP1	
cg03973705	chr16	24174850	PRKCB	
cg16307325	chr12	2339235	CACNA1C	
cg09554596	chr19	1004620	GRIN3B	Island
cg04933990	chr16	10133501	GRIN2A	
cg17652507	chr20	57463653	GNAS	
cg07080031	chr11	70675170	SHANK2	S_Shelf
cg05414613	chr1	53558470	SLC1A7	Island
cg00336149	chr3	53700195	CACNA1D	
cg03371918	chr17	64297988	PRKCA	N_Shore
cg00534626	chr16	9864730	GRIN2A	
cg13883984	chr15	83621779	HOMER2	
cg01103812	chr12	26986269	ITPR2	Island
cg25638611	chr11	70508420	SHANK2	
cg23398700	chr5	78808194	HOMER1	N_Shore
cg02602411	chr9	104357177	GRIN3A	
cg16899036	chr19	19052705	HOMER3	S_Shore
cg07502066	chr1	110091086	GNAI3	Island
cg05065846	chr7	126885001	GRM8	
cg05166022	chr12	2800471	CACNA1C	
cg15827003	chr1	1822912	GNB1	Island
ch.5.240336F	chr5	7757969	ADCY2	OpenSea
cg01007458	chr19	15084527	SLC1A6	OpenSea
cg01355739	chr20	57416888	GNASAS	
cg13523713	chr19	48614733	PLA2G4C	
cg02624051	chr15	42371635	PLA2G4D	Island
cg27433516	chr1	37266621	GRIK3	OpenSea
cg08982381	chr9	80647290	GNAQ	Island
cg02640306	chr2	191745287	GLS	Island
cg06614951	chr19	14229385	PRKACA	S_Shore
cg01686093	chr11	70491582	SHANK2	
cg19343464	chr11	105481509	GRIA4	

cg11692123	chr11	70935949	SHANK2	OpenSea
cg14123942	chr9	104500322	GRIN3A	
cg24753760	chr6	101846767	GRIK2	
cg09530407	chr11	22359486	SLC17A6	N_Shelf
cg12054318	chr20	57414529	GNASAS	
cg06783533	chr16	56388908	GNAO1	OpenSea
cg19088553	chr6	101901884	GRIK2	
cg20920827	chr11	70858475	SHANK2	OpenSea
cg09558195	chr6	146350585	GRM1	
cg07587653	chr11	70338480	SHANK2	
cg02794451	chr12	2800446	CACNA1C	
cg10474377	chr15	42131658	JMJD7-PLA2G4B	
cg08747970	chr11	70666452	SHANK2	Island
cg13823003	chr17	72856049	GRIN2C	Island
cg11996914	chr20	57414578	GNASAS	
cg02808075	chr3	4534881	ITPR1	
cg12452386	chr15	42302500	PLA2G4E	OpenSea
cg02725014	chr5	78809520	HOMER1	
cg01111718	chr11	105762374	GRIA4	
cg13588054	chr7	126892578	GRM8	Island
cg20492121	chr1	182361258	GLUL	
cg03832839	chr19	54401967	PRKCG	Island
cg06952307	chr17	47287974	ABI3	
cg09890339	chr12	2734150	CACNA1C	
cg05632631	chr16	24099614	PRKCB	
cg26534489	chr20	57427495	GNAS	
cg06085579	chr3	171509822	PLD1	
cg13830799	chr6	146750462	GRM1	
cg15464481	chr3	123151962	ADCY5	OpenSea
cg04269530	chr19	48919401	GRIN2D	Island
cg01141838	chr11	62474744	BSCL2	
cg11706780	chr15	42289766	PLA2G4E	OpenSea
cg21809160	chr20	57428309	GNAS	
cg01617139	chr19	2543861	GNG7	Island
cg20748533	chr19	51189975	SHANK1	Island
cg21102121	chr19	54401295	PRKCG	N_Shore
cg07408989	chr17	7116792	DLG4	
cg02736560	chr1	68257754	GNG12	OpenSea
cg17839232	chr5	36686601	SLC1A3	
cg10623198	chr12	6949114	GNB3	OpenSea
cg00050312	chr17	64299065	PRKCA	Island
cg20782596	chr20	57462978	GNAS	
cg13624528	chr6	101846409	GRIK2	
cg09551072	chr1	53567678	SLC1A7	OpenSea
cg18295203	chr3	171528273	PLD1	
cg08445323	chr16	4015030	ADCY9	Island
cg09529437	chr16	24136792	PRKCB	
cg03616148	chr16	9911529	GRIN2A	

cg16919771	chr7	126892599	GRM8	Island
cg07688749	chr12	46767132	SLC38A2	S_Shore
cg23696752	chr6	146349312	GRM1	
cg23491599	chr2	155554688	KCNJ3	Island
cg11802781	chr11	70370534	SHANK2	
cg12276123	chr2	155555157	KCNJ3	
cg26011633	chr5	178411667	GRM6	N_Shore
cg26952925	chr16	4166391	ADCY9	N_Shore
cg26379672	chr3	4534954	ITPR1	
cg15802396	chr17	7108305	DLG4	
cg13861294	chr12	100750473	SLC17A8	
cg05794931	chr11	70493931	SHANK2	
cg01716959	chr11	70515714	SHANK2	OpenSea
cg20645074	chr12	49182479	ADCY6	Island
cg14526297	chr6	146349488	GRM1	
cg17789138	chr19	49936880	SLC17A7	Island
cg13971030	chr11	35366721	SLC1A2	OpenSea
cg14767950	chr19	49939773	SLC17A7	Island
cg14158769	chr7	126698156	MIR592	
cg18950779	chr17	64685057	PRKCA	OpenSea
cg21797718	chr6	34024416	GRM4	Island
cg17665552	chr11	70455599	SHANK2	
cg10577016	chr9	114423680	GNG10	
cg25047001	chr16	10277017	GRIN2A	
cg19242688	chr19	47139391	GNG8	Island
cg08997444	chr20	57464970	GNAS	
cg21392385	chr15	52428589	GNB5	
cg22692013	chr12	2354618	CACNA1C	
cg22133366	chr11	70385327	SHANK2	
cg22065976	chr6	33589061	ITPR3	Island
cg13411554	chr3	53700276	CACNA1D	
cg27629673	chr5	7462856	ADCY2	OpenSea
cg25762078	chr1	53554504	SLC1A7	N_Shelf
cg00267746	chr20	57463984	GNAS	
cg13591723	chr12	46765135	SLC38A2	Island
cg18318307	chr11	70368307	SHANK2	
cg08464513	chr16	30136024	MAPK3	
cg01041222	chr4	158142863	GRIA2	
cg11820929	chr4	102258631	PPP3CA	
cg27369423	chr16	56228901	DKFZP434H168	
cg26904140	chr19	2703086	GNG7	S_Shore
cg11803871	chr3	123039831	ADCY5	OpenSea
cg12076692	chr1	1718852	GNB1	OpenSea
cg15916804	chr10	75255500	PPP3CB	
cg10259111	chr14	52429547	GNG2	OpenSea
cg25904372	chr11	70628970	SHANK2	OpenSea
cg11021321	chr20	57471660	GNAS	
cg21188533	chr3	53700263	CACNA1D	

cg02071600	chr5	78808852	HOMER1	
cg14851284	chr11	70713732	SHANK2	OpenSea
cg21885159	chr19	2645923	GNG7	S_Shelf
cg24995240	chr1	84971124	SPATA1	
cg12159995	chr11	70398671	SHANK2	
cg25451120	chr17	47287444	ABI3	
cg22983529	chr7	93551132	GNG11	
cg09858208	chr3	50283852	GNAI2	
cg10272968	chr19	2611105	GNG7	Island
cg15639581	chr19	13318873	CACNA1A	
cg08525508	chr19	48920297	GRIN2D	S_Shore
cg04127894	chr15	40594732	PLCB2	OpenSea
cg26503038	chr19	2699659	GNG7	N_Shelf
cg01864982	chr19	54409631	PRKCG	N_Shore
cg03242834	chr19	13317326	CACNA1A	
cg08111863	chr11	70882036	SHANK2	OpenSea
cg05884705	chr15	40600099	PLCB2	
cg15083678	chr12	2724200	CACNA1C	
cg23055735	chr11	70557464	SHANK2	OpenSea
cg22522961	chr1	68288817	GNG12	OpenSea
cg22598669	chr19	51186978	SHANK1	N_Shelf
cg18384228	chr14	24789158	ADCY4	S_Shelf
cg26672104	chr14	52327433	GNG2	
cg04988514	chr19	48947560	GRIN2D	
cg19120580	chr12	49173414	ADCY6	
cg13355041	chr18	3593715	FLJ35776	
cg08897844	chr5	36606102	SLC1A3	
cg27073113	chr16	56228744	DKFZP434H168	
cg21633134	chr5	7816399	ADCY2	OpenSea
cg16739976	chr6	34101401	GRM4	
cg20984065	chr3	51745321	GRM2	
cg16163543	chr11	70449219	SHANK2	
cg04761722	chr16	10274315	GRIN2A	
cg07571637	chr22	51143260	SHANK3	Island
cg10486865	chr17	64355892	PRKCA	OpenSea
cg10116505	chr16	10274064	GRIN2A	
cg09157251	chr11	70733251	SHANK2	OpenSea
cg11669839	chr20	57426322	GNAS	
cg02274728	chr20	57414407	GNASAS	
cg10891888	chr12	46765615	SLC38A2	Island
cg26361533	chr12	2445561	CACNA1C	
cg23725394	chr2	25057656	ADCY3	OpenSea
cg22569496	chr19	13409671	CACNA1A	
cg23464041	chr6	33663938	ITPR3	OpenSea
cg19946638	chr11	70859647	SHANK2	OpenSea
cg01449218	chr7	86273083	GRM3	OpenSea
cg10302550	chr20	57427821	GNAS	
cg24723883	chr19	2608495	GNG7	S_Shore

cg02521996	chr16	30134825	MAPK3	
cg09286797	chr20	8116999	PLCB1	
cg11758458	chr17	64575021	PRKCA	OpenSea
cg20699497	chr19	49944642	SLC17A7	
cg21216562	chr9	140045508	GRIN1	
cg19636672	chr12	56881121	GLS2	N_Shore
cg16671069	chr6	146350969	GRM1	
cg12566890	chr5	7494531	ADCY2	OpenSea
cg01354782	chr12	26522758	ITPR2	OpenSea
cg04903912	chr11	70374289	SHANK2	
cg10707626	chr3	51747098	GRM2	
cg00070899	chr6	34024479	GRM4	S_Shore
cg20579012	chr12	2803823	CACNA1C	
cg12667048	chr11	70644526	SHANK2	OpenSea
cg00043510	chr1	235814134	GNG4	
cg25652859	chr20	57427412	GNAS	
cg15878555	chr11	62473962	BSCL2	
cg03547757	chr20	57425515	GNASAS	
cg14212966	chr1	53558392	SLC1A7	Island
cg10851168	chr11	70317508	SHANK2	
cg08886546	chr19	19051335	HOMER3	
cg22885821	chr20	57465921	GNAS	
cg09726240	chr11	70672878	SHANK2	Island
cg07687951	chr11	70666560	SHANK2	Island
cg20213508	chr20	57463325	GNAS	
cg15442907	chr12	2800463	CACNA1C	
cg00997853	chr12	26844579	ITPR2	OpenSea
cg09659734	chr16	56227065	DKFZP434H168	
cg24008901	chr12	13715497	GRIN2B	N_Shore
cg00601648	chr8	131974587	ADCY8	OpenSea
cg27661264	chr20	57427738	GNAS	
cg12116020	chr19	14228622	PRKACA	Island
cg21625881	chr20	57430313	GNAS	
cg24033471	chr12	2735579	CACNA1C	
cg06194010	chr12	2493567	CACNA1C	
cg11480627	chr11	70672876	SHANK2	Island
cg07746960	chr19	42546662	GRIK5	Island
cg09555879	chr19	42569956	GRIK5	
cg00765653	chr20	57415144	GNASAS	
cg09320113	chr1	186799481	PLA2G4A	OpenSea
cg25338454	chr12	26900022	ITPR2	OpenSea
cg14648237	chr17	64422393	PRKCA	OpenSea
cg20936920	chr3	7782210	GRM7	
cg24824840	chr19	51219975	SHANK1	N_Shore
cg03399271	chr16	56228385	DKFZP434H168	
cg08166863	chr20	57426391	GNAS	
cg04473078	chr16	4165886	ADCY9	
cg17222829	chr11	70433293	SHANK2	

cg11830694	chr12	2289797	CACNA1C	
cg24086869	chr15	52471581	GNB5	
cg21350778	chr17	64297603	PRKCA	N_Shore
cg09742895	chr11	105781302	GRIA4	
cg04803128	chr7	100273280	GNB2	Island
cg14396328	chr7	45761429	ADCY1	OpenSea
cg10482356	chr16	56328421	GNAO1	
cg25521963	chr17	7123553	DLG4	
cg14208013	chr20	57485765	GNAS	
cg06932616	chr19	48908335	GRIN2D	Island
cg24009995	chr14	52434702	GNG2	OpenSea
cg07700514	chr16	56228467	DKFZP434H168	
cg04446870	chr17	64440273	PRKCA	OpenSea
cg03306374	chr16	23847325	PRKCB	
cg16620233	chr3	53781107	CACNA1D	
cg01331810	chr7	86414302	GRM3	N_Shore
cg13914083	chr4	158141526	GRIA2	
cg09017174	chr11	35440525	SLC1A2	
cg26613742	chr19	14225000	PRKACA	
cg11953334	chr19	48897863	GRIN2D	S_Shore
cg08505135	chr19	19045615	HOMER3	
cg11692021	chr12	6949472	GNB3	
cg08021727	chr16	4056879	ADCY9	OpenSea
cg07218663	chr6	146350618	GRM1	
cg02240622	chr15	40601467	PLCB2	OpenSea
cg05279172	chr17	7113697	DLG4	
cg17846122	chr17	64351144	PRKCA	OpenSea
cg12544243	chr19	1005641	GRIN3B	N_Shore
cg02954212	chr16	56226350	LOC283856	
cg10820904	chr17	64412745	PRKCA	OpenSea
cg19794207	chr9	140040231	GRIN1	
cg12240358	chr15	83619523	HOMER2	
cg11906607	chr19	2524064	GNG7	N_Shore
cg23956071	chr17	4710044	PLD2	Island
cg16400825	chr6	33589418	ITPR3	Island
cg10671676	chr5	178413560	GRM6	Island
cg14090916	chr9	140044904	GRIN1	
cg20822365	chr15	83621694	HOMER2	
cg25423647	chr3	51746723	GRM2	
cg03264550	chr20	57465448	GNAS	
cg10503359	chr3	142468059	TRPC1	OpenSea
cg21636577	chr17	7121881	ACADVL	
cg05185634	chr14	24804750	ADCY4	S_Shore
cg16600501	chr19	15083842	SLC1A6	OpenSea
cg07926858	chr10	75252761	PPP3CB	
cg17726655	chr19	42509860	GRIK5	Island
cg04260676	chr1	1774322	GNB1	OpenSea
cg02328440	chr1	235813839	GNG4	

cg11155924	chr11	70449258	SHANK2	
cg06874426	chr17	47287526	ABI3	
cg09152120	chr16	4016669	ADCY9	Island
cg13016048	chr9	140054056	GRIN1	
cg07024458	chr16	56390600	GNAO1	OpenSea
cg18046365	chr1	53608367	SLC1A7	OpenSea
cg06864895	chr12	46767683	SLC38A2	S_Shore
cg02288564	chr19	49934404	SLC17A7	Island
cg16601231	chr19	13367946	CACNA1A	
cg23740474	chr11	70455140	SHANK2	
cg14992273	chr1	37337827	GRIK3	OpenSea
cg27318000	chr18	3845667	DLGAP1	
cg27643147	chr11	70517294	SHANK2	OpenSea
cg02953559	chr3	123164964	ADCY5	N_Shore
cg23613253	chr11	70440347	SHANK2	
cg09997760	chr3	179169556	GNB4	Island
cg05161074	chr15	42289885	PLA2G4E	OpenSea
cg23965720	chr1	235805770	GNG4	
cg24151995	chr11	22364293	SLC17A6	S_Shore
cg00383081	chr1	182362088	GLUL	
cg19324023	chr22	51170003	SHANK3	Island
cg21830821	chr15	52475339	GNB5	S_Shelf
cg13000134	chr19	48896922	GRIN2D	Island
cg24339704	chr19	2529022	GNG7	S_Shelf
cg16009558	chr6	101846707	GRIK2	
cg08038054	chr7	93550781	GNG11	OpenSea
cg17119568	chr19	14229237	PRKACA	Island
cg14203613	chr3	4714928	ITPR1	
cg02991464	chr12	2788732	CACNA1C	
cg25193885	chr11	70328867	SHANK2	
cg22290117	chr20	57427173	GNAS	
cg24092939	chr12	49181056	ADCY6	N_Shore
cg12664560	chr15	83621517	HOMER2	
cg17159473	chr7	126893890	GRM8	Island
cg05397010	chr15	42448259	PLA2G4F	
cg20772037	chr6	34067242	GRM4	OpenSea
cg11435239	chr19	51220297	SHANK1	Island
cg07451034	chr17	47283774	GNGT2	OpenSea
cg25534294	chr2	155554931	KCNJ3	Island
cg08374499	chr19	49941241	SLC17A7	S_Shore
cg08460548	chr19	49944817	SLC17A7	N_Shore
cg13916928	chr11	62474735	BSCL2	
cg12981270	chr19	42509946	GRIK5	Island
cg19293162	chr19	2513302	GNG7	Island
cg05722993	chr1	1727796	GNB1	OpenSea
cg16253976	chr5	7686199	ADCY2	OpenSea
cg11601336	chr19	19053012	HOMER3	S_Shore
cg03016097	chr15	83621726	HOMER2	

cg26266429	chr12	49174651	ADCY6	
cg19853565	chr19	2540907	GNG7	Island
cg22459924	chr19	2607850	GNG7	Island
cg01399255	chr7	100271274	GNB2	Island
cg09853822	chr17	4712456	PLD2	S_Shore
cg13523557	chr7	45613725	ADCY1	Island
cg05546044	chr22	22222597	MAPK1	
cg06692957	chr9	80647629	GNAQ	S_Shore
cg24067803	chr11	70653378	SHANK2	OpenSea
cg09641955	chr12	2716844	CACNA1C	
cg08028452	chr3	53545444	CACNA1D	
cg18522549	chr11	62473861	BSCL2	
cg18761756	chr18	3732002	DLGAP1	
cg08104845	chr1	182356337	GLUL	
cg25894071	chr4	101953935	PPP3CA	
cg08505222	chr22	51139277	SHANK3	N_Shelf
cg02929073	chr7	126892249	GRM8	
cg11801011	chr19	51220537	SHANK1	N_Shore
cg26235243	chr6	33656836	ITPR3	S_Shore
cg00050938	chr3	50296100	GNAI2	
cg21028562	chr3	50287909	GNAI2	
cg02380983	chr19	48568071	PLA2G4C	
cg05960039	chr20	57465123	GNAS	
cg05848509	chr17	72857354	GRIN2C	Island
cg10917153	chr15	42448786	PLA2G4F	
cg14792155	chr15	42289618	PLA2G4E	OpenSea
cg26322763	chr19	48914444	GRIN2D	N_Shelf
cg10139742	chr16	56352151	GNAO1	
cg25475999	chr11	35282046	SLC1A2	OpenSea
cg20759084	chr5	7395875	ADCY2	Island
cg23374892	chr19	51165845	SHANK1	S_Shore
cg15279308	chr12	2800500	CACNA1C	
cg06185738	chr11	22359868	SLC17A6	
cg22860367	chr20	57426538	GNAS	
cg10083824	chr6	34102147	GRM4	OpenSea
cg19892433	chr11	70331861	SHANK2	
cg06646622	chr5	78766902	HOMER1	OpenSea
cg16774375	chr16	4102333	ADCY9	OpenSea
cg13533759	chr9	114431056	GNG10	
cg13065504	chr15	42448234	PLA2G4F	
cg04251662	chr3	4535075	ITPR1	
cg10206594	chr1	37314665	GRIK3	OpenSea
cg08931917	chr12	14109569	GRIN2B	OpenSea
cg27529848	chr15	52472330	GNB5	
cg07482508	chr5	36606981	SLC1A3	
cg00986191	chr5	178419655	GRM6	N_Shore
cg26601922	chr3	50243174	SLC38A3	Island
cg21635870	chr6	101847058	GRIK2	

cg12372477	chr20	57465915	GNAS	
cg15680973	chr5	7420177	ADCY2	OpenSea
cg16040341	chr15	83544284	HOMER2	
cg00671386	chr16	851834	GNG13	S_Shore
cg23854103	chr19	2543602	GNG7	N_Shore
cg04498418	chr6	101850540	GRIK2	
cg02232377	chr7	86297084	GRM3	OpenSea
cg14957718	chr3	50243260	SLC38A3	Island
cg26454299	chr4	102268957	PPP3CA	
cg06849501	chr11	70458964	SHANK2	
cg26764244	chr1	68299511	GNG12	S_Shore
cg10324572	chr3	51749334	GRM2	
cg20490175	chr12	2797745	CACNA1C	
cg00720707	chr16	4016845	ADCY9	Island
cg27277859	chr19	51165632	SHANK1	Island
cg05765440	chr1	68252343	GNG12	OpenSea
cg03104569	chr19	13615864	CACNA1A	
cg15219163	chr11	70842128	SHANK2	OpenSea
cg03100024	chr19	42571402	GRIK5	OpenSea
cg00350503	chr19	13613614	CACNA1A	
cg06147822	chr20	57466905	GNAS	
cg01246398	chr3	123165872	ADCY5	N_Shore
cg02500883	chr9	104356619	GRIN3A	
cg25983380	chr20	57465439	GNAS	
cg15218096	chr11	70858342	SHANK2	OpenSea
cg27299660	chr3	171527797	PLD1	
cg12312205	chr18	3594173	DLGAP1	
cg00943909	chr20	57427942	GNAS	
cg14731698	chr11	120738292	GRIK4	OpenSea
cg13353325	chr20	57485837	GNAS	
cg16890681	chr2	68479269	PPP3R1	
cg22134372	chr15	52455211	GNB5	
cg21462934	chr19	2622858	GNG7	Island
cg25751482	chr17	64301327	PRKCA	S_Shore
cg12008034	chr6	33996580	GRM4	S_Shore
cg22088263	chr3	51746830	GRM2	
cg25210134	chr15	40600265	PLCB2	OpenSea
cg03043696	chr1	1820656	GNB1	N_Shore
cg19217955	chr17	7123994	DLG4	
cg10011623	chr20	57463527	GNAS	
cg13740815	chr19	51170356	SHANK1	Island
cg02892153	chr18	3593461	FLJ35776	
cg10347199	chr17	7123561	DLG4	
cg14337339	chr3	53529481	CACNA1D	
cg20484832	chr20	9075810	PLCB4	
cg07851738	chr8	131896788	ADCY8	OpenSea
cg24000444	chr18	3771452	DLGAP1	
cg21673873	chr11	70511688	SHANK2	S_Shelf

cg19251850	chr3	171428254	PLD1	
cg26129110	chr5	7619983	ADCY2	OpenSea
cg15109207	chr19	48614773	PLA2G4C	
cg19244300	chr1	110113304	GNAI3	OpenSea
cg00732970	chr20	57414162	GNASAS	
cg14791525	chr11	70732224	SHANK2	OpenSea
cg16850687	chr18	3594398	DLGAP1	
cg01157070	chr16	56228511	DKFZP434H168	
cg13416129	chr9	140037808	GRIN1	
cg08698835	chr16	56279276	GNAO1	
cg22076160	chr1	235805690	GNG4	
cg01748573	chr20	57463530	GNAS	
cg13054613	chr3	7742036	GRM7	
cg27552287	chr19	54385396	PRKCG	Island
cg08644463	chr1	110106962	GNAI3	OpenSea
cg22484822	chr6	33996111	GRM4	Island
cg01090161	chr6	34032747	GRM4	OpenSea
cg07713849	chr6	33624841	ITPR3	OpenSea
cg16986624	chr15	52471717	GNB5	
cg01865825	chr16	850240	GNG13	Island
cg11118235	chr3	50284010	GNAI2	
cg14594362	chr19	48947631	GRIN2D	
cg26212328	chr12	46767665	SLC38A2	S_Shore
cg11676382	chr22	51141829	SHANK3	N_Shore
cg06181697	chr3	50294603	GNAI2	
cg10283969	chr11	70666639	SHANK2	Island
cg17641631	chr3	4535021	ITPR1	
cg19047292	chr16	56228442	DKFZP434H168	
cg06173536	chr1	235814462	GNG4	
cg09505516	chr2	25110296	ADCY3	OpenSea
cg05255330	chr18	3498963	DLGAP1	
cg22531801	chr1	235806070	GNG4	
cg15114328	chr1	182361557	GLUL	
cg14788049	chr11	64018549	PLCB3	Island
cg08897759	chr5	178415944	GRM6	Island
cg12452300	chr3	51751788	GRM2	
cg10181414	chr19	2546598	GNG7	N_Shore
cg16548911	chr16	50347766	ADCY7	OpenSea
cg03199239	chr19	2588553	GNG7	Island
cg02910037	chr16	4102366	ADCY9	OpenSea
cg06898306	chr6	33996673	GRM4	S_Shore
cg10306450	chr12	56882479	GLS2	Island
cg26635576	chr11	35275997	SLC1A2	OpenSea
cg12732284	chr3	171320284	PLD1	
cg27644733	chr16	9857216	GRIN2A	
cg11414276	chr12	2166831	CACNA1C	
cg10699496	chr3	123005576	ADCY5	OpenSea
cg21422400	chr1	1747243	GNB1	OpenSea

cg20528838	chr20	57427730	GNAS	
cg15212295	chr17	64710687	PRKCA	OpenSea
cg06940168	chr17	64370665	PRKCA	OpenSea
cg14399447	chr19	48613950	PLA2G4C	
cg07986199	chr12	2743038	CACNA1C	
cg09654471	chr3	4624132	ITPR1	
cg25418001	chr7	79780310	GNAI1	OpenSea
cg17952046	chr1	37379413	GRIK3	OpenSea
cg07561162	chr16	10174417	GRIN2A	
cg21971807	chr20	57471654	GNAS	
cg04576491	chr19	1005427	GRIN3B	Island
cg06441398	chr11	70317455	SHANK2	
cg12727358	chr3	53839610	CACNA1D	
cg20495738	chr12	2338399	CACNA1C	
cg13980113	chr5	36607333	SLC1A3	
cg14583825	chr19	54393040	PRKCG	Island
cg24266105	chr6	146351044	GRM1	
cg00498360	chr17	64504304	PRKCA	OpenSea
cg14967731	chr3	4748984	ITPR1	
cg11727252	chr3	51752143	GRM2	
cg17566735	chr3	171527257	PLD1	
cg27501686	chr12	2184874	CACNA1C	
cg11221524	chr1	84969091	GNG5	N_Shelf
cg08884490	chr22	51160651	SHANK3	S_Shore
cg23522194	chr19	48565189	PLA2G4C	
cg11921270	chr19	19051154	HOMER3	
cg24155399	chr1	235781578	GNG4	
cg27112585	chr11	70378117	SHANK2	
cg05765011	chr16	4103225	ADCY9	OpenSea
cg08407014	chr19	2541104	GNG7	Island
cg13759674	chr9	140051205	GRIN1	
cg27451362	chr6	101846650	GRIK2	
cg27363558	chr17	7108792	DLG4	
cg14149552	chr9	80642464	GNAQ	N_Shelf
cg01520586	chr3	50280404	GNAI2	OpenSea
cg08411235	chr11	35297026	SLC1A2	OpenSea
cg24090202	chr19	54410030	PRKCG	Island
cg06541349	chr16	4015096	ADCY9	Island
cg02840199	chr16	4165257	ADCY9	Island
cg02757172	chr4	158141076	GRIA2	
cg01661235	chr16	23988974	PRKCB	
cg18619398	chr20	57416506	GNASAS	
cg19653589	chr19	2614177	GNG7	S_Shore
cg09611472	chr16	850371	GNG13	Island
cg15067127	chr1	182352978	GLUL	
cg04367107	chr11	88238963	GRM5	
cg12102973	chr19	13320368	CACNA1A	
cg08860136	chr17	7111414	DLG4	

cg02760164	chr15	42371967	PLA2G4D	S_Shore
cg05366189	chr19	13397419	CACNA1A	
cg15815156	chr15	52472201	GNB5	
cg17402889	chr12	2527099	CACNA1C	
cg25456593	chr11	70672858	SHANK2	Island
cg10533538	chr16	4166864	ADCY9	Island
cg23542572	chr17	64780054	PRKCA	OpenSea
cg26320601	chr12	26986803	ITPR2	S_Shore
cg02754494	chr5	78810199	HOMER1	Island
cg24882525	chr11	70565226	SHANK2	S_Shore
cg16850173	chr9	140033611	GRIN1	
cg19577617	chr2	191745301	GLS	Island
cg01509809	chr17	47284233	GNGT2	OpenSea
cg16833551	chr20	57427237	GNAS	
cg08619378	chr7	45616358	ADCY1	S_Shore
cg09284949	chr19	51190179	SHANK1	S_Shore
cg05607461	chr2	191745416	GLS	Island
cg01700524	chr14	24803014	ADCY4	Island
cg22901212	chr19	1003348	GRIN3B	Island
cg19999705	chr6	34026903	GRM4	S_Shelf
cg21142456	chr15	42376744	PLA2G4D	OpenSea
cg08001559	chr14	52326905	GNG2	OpenSea
cg27272547	chr19	13347337	CACNA1A	
cg12535596	chr1	182360697	GLUL	
cg16829998	chr19	49944964	SLC17A7	N_Shore
cg13371705	chr12	2452955	CACNA1C	
cg26345619	chr12	2602405	CACNA1C	
cg19700341	chr17	72857076	GRIN2C	Island
cg26343183	chr5	153008721	GRIA1	
cg09576209	chr12	2339614	CACNA1C	
cg07064537	chr3	171515212	PLD1	
cg08578734	chr5	152870490	GRIA1	
cg25562925	chr2	25110049	ADCY3	OpenSea
cg04202736	chr15	42371721	PLA2G4D	Island
cg17867333	chr5	178423163	GRM6	S_Shore
cg14011070	chr1	235811811	GNG4	
cg03716942	chr1	1821981	GNB1	Island
cg20408693	chr12	46767289	SLC38A2	S_Shore
cg25869295	chr11	70433086	SHANK2	
cg19206040	chr1	37500441	GRIK3	Island
cg18574254	chr7	126889015	GRM8	N_Shelf
cg20518994	chr2	25141532	ADCY3	N_Shore
cg00348762	chr16	56228114	DKFZP434H168	
cg05511872	chr11	70824260	SHANK2	OpenSea
cg22798121	chr5	152896564	GRIA1	
cg01130792	chr22	51112683	SHANK3	Island
cg11335969	chr22	22118304	MAPK1	OpenSea
cg27271486	chr16	56227690	DKFZP434H168	

cg15372689	chr7	93550986	GNG11	OpenSea
cg14638988	chr11	62476544	BSCL2	
cg22363670	chr7	86273169	GRM3	OpenSea
cg21779904	chr20	57425157	GNASAS	
cg20019489	chr20	57414351	GNASAS	
cg12281620	chr19	2525290	GNG7	Island
cg05456713	chr19	2513356	GNG7	Island
cg01397507	chr11	70440395	SHANK2	
cg20306837	chr11	105481988	GRIA4	
cg00377653	chr12	56882535	GLS2	Island
cg08572336	chr19	51165404	SHANK1	Island
cg16448399	chr18	3880076	DLGAP1	
cg07959068	chr16	24057138	PRKCB	
cg14116756	chr10	75255721	PPP3CB	
cg25733708	chr19	54393220	PRKCG	Island
cg14241370	chr9	104499850	GRIN3A	Island
cg03731464	chr12	2801158	CACNA1C	
cg04742605	chr11	70385511	SHANK2	
cg10487659	chr16	4152081	ADCY9	OpenSea
cg19542445	chr12	2596220	CACNA1C	
cg15389472	chr1	182361528	GLUL	
cg17155859	chr11	64026541	PLCB3	Island
cg03321319	chr22	22222323	MAPK1	
cg07114886	chr3	51745946	GRM2	
cg04190002	chr22	51113604	SHANK3	Island
cg17024257	chr3	171528758	PLD1	
cg18589960	chr19	51220392	SHANK1	N_Shore
cg15418783	chr11	70557693	SHANK2	OpenSea
cg17260383	chr8	22298246	PPP3CC	Island
cg07268119	chr7	45717573	ADCY1	OpenSea
cg06137273	chr3	6905031	GRM7	
cg24904943	chr5	7781077	ADCY2	OpenSea
cg04518808	chr19	54384822	PRKCG	N_Shore
cg02540833	chr11	70562363	SHANK2	N_Shore
cg07601741	chr5	153160425	GRIA1	
cg02274788	chr1	68232457	GNG12	OpenSea
cg13592780	chr3	123010034	ADCY5	OpenSea
cg24671939	chr18	3593798	FLJ35776	
cg04378167	chr19	49944943	SLC17A7	N_Shore
cg25592107	chr19	19042864	HOMER3	
cg21650436	chr12	49162444	ADCY6	
cg25196508	chr12	49181554	ADCY6	N_Shore
cg07947033	chr20	57426545	GNAS	
cg16098545	chr12	26492312	ITPR2	OpenSea
cg01450274	chr3	171396325	PLD1	
cg08066673	chr14	52325747	GNG2	OpenSea
cg09787442	chr21	31119294	GRIK1	
cg04005969	chr19	51171247	SHANK1	Island

cg15877314	chr3	50273895	GNAI2	Island
cg03014008	chr20	57463767	GNAS	
cg20998200	chr11	22370099	SLC17A6	OpenSea
cg07559526	chr16	4164735	ADCY9	N_Shore
cg07960450	chr7	45614300	ADCY1	Island
cg10336707	chr11	62476526	BSCL2	
cg11435826	chr12	2792111	CACNA1C	
cg14851700	chr1	182362230	GLUL	
cg24037166	chr10	75255724	PPP3CB	
cg08515427	chr16	848997	GNG13	N_Shore
cg26983544	chr5	78784024	HOMER1	OpenSea
cg06192619	chr19	15083616	SLC1A6	OpenSea
cg02557189	chr7	100273384	GNB2	Island
cg22562461	chr7	86274769	GRM3	OpenSea
cg14350337	chr9	140060064	GRIN1	
cg14815005	chr22	22222162	MAPK1	
cg23427362	chr12	2335272	CACNA1C	
cg03058660	chr19	48919198	GRIN2D	Island
cg25121007	chr7	126854672	GRM8	
cg00269140	chr7	86389542	GRM3	OpenSea
cg24946911	chr12	2788654	CACNA1C	
cg02879453	chr16	50321818	ADCY7	OpenSea
cg20170028	chr11	70917283	SHANK2	OpenSea
cg24218925	chr19	2578938	GNG7	N_Shore
cg09150064	chr11	22364185	SLC17A6	S_Shore
cg06489744	chr11	70557772	SHANK2	OpenSea
cg05902503	chr16	30133175	MAPK3	
cg02525785	chr17	7117684	DLG4	
cg08033640	chr11	70419197	SHANK2	
cg27184649	chr11	70935963	SHANK2	OpenSea
cg17752088	chr5	78810367	HOMER1	Island
cg03330642	chr5	178411124	GRM6	N_Shelf
cg10797197	chr20	57444000	GNAS	
cg15350840	chr11	70475709	SHANK2	
cg23281712	chr2	25143391	ADCY3	Island
cg21114126	chr4	102267189	PPP3CA	
cg22986870	chr7	126765549	GRM8	
cg01638185	chr17	64530027	PRKCA	OpenSea
cg23698058	chr1	84544097	PRKACB	
cg10331829	chr11	35343789	SLC1A2	OpenSea
cg00792185	chr16	9855960	GRIN2A	
cg04603130	chr19	2550027	GNG7	S_Shelf
cg18846074	chr14	24801073	ADCY4	N_Shore
cg09437522	chr20	57431202	GNAS	
cg16143105	chr5	78689278	HOMER1	OpenSea
cg04398695	chr16	850713	GNG13	
cg01386883	chr3	53529144	CACNA1D	
cg11116429	chr5	36607417	SLC1A3	

cg04903916	chr6	33638413	ITPR3	OpenSea
cg27107076	chr2	25050844	ADCY3	OpenSea
cg20288565	chr1	235805403	GNG4	
cg04835297	chr3	142443257	TRPC1	Island
cg08204867	chr16	10208426	GRIN2A	
cg20979061	chr19	49939949	SLC17A7	Island
cg03871526	chr3	142447949	TRPC1	S_Shelf
cg26495109	chr16	56225952	LOC283856	
cg10639428	chr2	25138879	ADCY3	N_Shelf
cg24432193	chr6	102062733	GRIK2	
cg04085699	chr11	64026024	PLCB3	Island
cg05339056	chr7	86391162	GRM3	OpenSea
cg14150378	chr9	80335274	GNAQ	OpenSea
cg19589727	chr20	57427762	GNAS	
cg24194077	chr19	54386020	PRKCG	S_Shore
cg00116766	chr16	9857614	GRIN2A	
cg06490627	chr16	50344198	ADCY7	OpenSea
cg27351813	chr19	51197056	SHANK1	N_Shore
cg15160746	chr12	2743239	CACNA1C	
cg14351882	chr9	140061878	GRIN1	
cg08969344	chr6	34031597	GRM4	OpenSea
cg10801143	chr11	88245465	GRM5	
cg03011594	chr16	56370697	GNAO1	
cg10371523	chr11	70395513	SHANK2	
cg16218964	chr11	62473680	BSCL2	
cg01010868	chr19	14228654	PRKACA	Island
cg19151292	chr6	33653502	ITPR3	N_Shelf
cg12128893	chr10	75255807	PPP3CB	
cg18618964	chr2	68480222	PPP3R1	S_Shore
cg23460210	chr19	54385404	PRKCG	Island
cg26010751	chr3	51742513	GRM2	
cg21130255	chr11	70368676	SHANK2	
cg25144574	chr5	36655019	SLC1A3	
cg08364956	chr5	178407122	GRM6	OpenSea
cg26826325	chr11	70459053	SHANK2	
cg01014438	chr12	2762689	CACNA1C	
cg06200857	chr20	57426420	GNAS	
cg13651483	chr19	48566587	PLA2G4C	
cg03010274	chr20	57427274	GNAS	
cg26389955	chr17	64640562	PRKCA	OpenSea
cg04404381	chr11	70563580	SHANK2	N_Shore
cg21599324	chr6	34074282	GRM4	OpenSea
cg26674800	chr17	7108653	DLG4	
cg23913904	chr11	70590130	SHANK2	OpenSea
cg13139998	chr3	51740201	GRM2	
cg06015218	chr6	146350434	GRM1	
cg16730369	chr11	120823575	GRIK4	OpenSea
cg06739873	chr19	19040289	HOMER3	

cg10692302	chr3	51747227	GRM2	
cg03478199	chr6	146348913	GRM1	
cg17755730	chr3	50285392	GNAI2	
cg13844463	chr11	35436672	SLC1A2	N_Shelf
cg07212894	chr3	50243021	SLC38A3	Island
cg03882437	chr9	140051729	GRIN1	
cg26452915	chr20	57486076	GNAS	
cg10887945	chr3	4805396	ITPR1	
cg09405076	chr11	70477139	SHANK2	
cg23077606	chr11	70540206	SHANK2	OpenSea
cg12996903	chr3	50275575	GNAI2	Island
cg19640589	chr20	57427973	GNAS	
cg10144604	chr20	57465599	GNAS	
cg19325477	chr17	64688496	PRKCA	OpenSea
cg16862319	chr12	13899195	GRIN2B	OpenSea
cg11357538	chr20	57463397	GNAS	
cg22242216	chr19	48947578	GRIN2D	
cg06952422	chr6	33647685	ITPR3	OpenSea
cg22956310	chr2	25142878	ADCY3	Island
cg08123425	chr12	13939517	GRIN2B	OpenSea
cg17540499	chr7	126698451	MIR592	
cg11317158	chr19	49938204	SLC17A7	N_Shore
cg18166990	chr11	70515565	SHANK2	OpenSea
cg13329789	chr7	45757644	ADCY1	OpenSea
cg24867458	chr1	110090677	GNAI3	N_Shore
cg14414124	chr2	68465373	PPP3R1	OpenSea
cg25673737	chr4	101966414	PPP3CA	
cg00951869	chr14	24805349	ADCY4	
cg10468484	chr20	57431303	GNAS	
cg18162783	chr1	1795891	GNB1	OpenSea
cg16475558	chr16	56388945	GNAO1	OpenSea
cg19270265	chr7	126275080	GRM8	
cg16446012	chr5	78772259	HOMER1	OpenSea
cg00318899	chr3	50284137	GNAI2	
cg12536809	chr17	72852514	GRIN2C	N_Shelf
cg00991400	chr7	126883137	GRM8	
cg25976932	chr3	123138958	ADCY5	OpenSea
cg23753795	chr6	146531540	GRM1	
cg12771777	chr11	70692038	SHANK2	OpenSea
cg04509024	chr1	37270540	GRIK3	OpenSea
cg11856810	chr2	155554961	KCNJ3	Island
cg12363682	chr5	178420690	GRM6	N_Shore
cg17334845	chr20	57463572	GNAS	
cg26060003	chr1	53558511	SLC1A7	Island
cg03182218	chr17	7100221	DLG4	
cg01637551	chr12	2161661	CACNA1C	
cg00417823	chr16	56330268	GNAO1	
cg06163629	chr20	57414884	GNASAS	

cg24183324	chr11	35440062	SLC1A2	N_Shore
cg03606258	chr20	57426935	GNAS	
cg16693012	chr1	68283821	GNG12	OpenSea
cg09868882	chr7	126883640	GRM8	
cg11841246	chr15	52414751	GNB5	
cg14298577	chr7	100272703	GNB2	Island
cg09710790	chr3	4534905	ITPR1	
cg13641156	chr11	70515650	SHANK2	OpenSea
cg12201698	chr11	70634975	SHANK2	OpenSea
cg08834938	chr16	4136053	ADCY9	OpenSea
cg00631706	chr5	78751239	HOMER1	OpenSea
cg06979118	chr11	70601971	SHANK2	OpenSea
cg25367568	chr20	57428437	GNAS	
cg18457944	chr3	171428279	PLD1	
cg21156276	chr9	4491917	SLC1A1	S_Shore
cg09635994	chr22	51171263	SHANK3	S_Shore
cg04476846	chr5	78791415	HOMER1	OpenSea
cg03162045	chr3	142443933	TRPC1	Island
cg16106068	chr14	24792081	ADCY4	OpenSea
cg15620385	chr12	26986279	ITPR2	Island
cg00925020	chr19	2543877	GNG7	Island
cg18080819	chr11	70505972	SHANK2	
cg16909293	chr1	68171537	GNG12	OpenSea
cg14694901	chr10	75255186	PPP3CB	
cg26201811	chr22	51111714	SHANK3	N_Shore
cg25381331	chr1	53556414	SLC1A7	N_Shore
cg08486432	chr6	33598003	ITPR3	N_Shelf
cg08712808	chr3	7342929	GRM7	
cg19128261	chr3	53839251	CACNA1D	
cg10276272	chr16	10271822	GRIN2A	
cg10613332	chr19	13368845	CACNA1A	
cg15765694	chr9	104358101	PPP3R2	
cg26332715	chr2	191745502	GLS	Island
cg09179079	chr6	146348690	GRM1	
cg13078421	chr19	2624622	GNG7	S_Shore
cg23315601	chr11	120590134	GRIK4	OpenSea
cg18287522	chr3	179115547	GNB4	OpenSea
cg10161743	chr19	48917816	GRIN2D	N_Shore
cg09950871	chr16	4029235	ADCY9	Island
cg11010575	chr15	42129464	JMJD7-PLA2G4B	
cg11539664	chr6	33604664	ITPR3	S_Shelf
cg27642181	chr20	57413694	GNASAS	
cg05626242	chr10	75255789	PPP3CB	
cg17961101	chr22	22222050	MAPK1	
cg11814875	chr19	2611237	GNG7	Island
cg02902102	chr19	48902290	GRIN2D	S_Shore
cg24030173	chr19	48922140	GRIN2D	S_Shelf
cg11422541	chr12	49182586	ADCY6	Island

cg18117347	chr19	51196754	SHANK1	N_Shore
cg01944370	chr3	50286969	GNAI2	
cg01937808	chr16	4025588	ADCY9	N_Shore
cg01174786	chr16	4027541	ADCY9	Island
cg01017090	chr20	57427046	GNAS	
cg18160880	chr20	57463903	GNAS	
cg07526227	chr19	51168305	SHANK1	N_Shore
cg07121340	chr12	2173987	CACNA1C	
cg10998242	chr3	53530847	CACNA1D	
cg08505076	chr17	7122063	ACADVL	
cg12760563	chr18	3594396	DLGAP1	
cg05329317	chr16	30126595	MAPK3	
cg04733951	chr17	64545218	PRKCA	OpenSea
cg15522719	chr2	191745248	GLS	N_Shore
cg25316569	chr11	120530952	GRIK4	OpenSea
cg11659796	chr19	2560038	GNG7	S_Shelf
cg07547788	chr7	93534693	GNGT1	OpenSea
cg21250721	chr9	140056227	GRIN1	
cg18414579	chr4	102266325	PPP3CA	
cg12650926	chr6	33589118	ITPR3	Island
cg14116052	chr5	7596454	ADCY2	OpenSea
cg05921947	chr5	7715579	ADCY2	OpenSea
cg27176392	chr19	2652259	GNG7	S_Shore
cg02607130	chr19	1008643	GRIN3B	Island
cg16379885	chr1	37500369	GRIK3	Island
cg01104717	chr3	53545550	CACNA1D	
cg22834542	chr11	88288530	GRM5	
cg06930757	chr19	51216389	SHANK1	N_Shelf
cg25804443	chr18	3875823	DLGAP1	N_Shelf
cg02433656	chr16	56322654	GNAO1	
cg16920001	chr15	42448760	PLA2G4F	
cg27586581	chr19	19051157	HOMER3	
cg00800141	chr16	4014161	ADCY9	N_Shore
cg26199552	chr11	70368372	SHANK2	
cg21340148	chr19	2702986	GNG7	Island
cg03654504	chr1	37495105	GRIK3	N_Shelf
cg01728682	chr2	25057480	ADCY3	OpenSea
cg24825722	chr17	7121848	ACADVL	
cg13939602	chr12	2467410	CACNA1C	
cg01823958	chr1	53557455	SLC1A7	N_Shore
cg01419479	chr1	182360822	GLUL	
cg21375506	chr17	64470354	PRKCA	OpenSea
cg02637414	chr19	13613091	CACNA1A	
cg15122716	chr9	140050568	GRIN1	
cg19015708	chr2	68418292	PPP3R1	OpenSea
cg04994975	chr1	1795945	GNB1	OpenSea
cg04279973	chr16	23846968	PRKCB	
cg23686556	chr8	131961316	ADCY8	OpenSea

cg27019717	chr14	52351055	GNG2	OpenSea
cg27304369	chr20	57427483	GNAS	
cg27279809	chr12	49183212	ADCY6	Island
cg26496204	chr20	57427210	GNAS	
cg20910008	chr8	22301378	PPP3CC	S_Shelf
cg19558628	chr14	24801616	ADCY4	N_Shore
cg07841877	chr1	182359858	GLUL	
cg17641710	chr3	50279038	GNAI2	S_Shelf
cg20716703	chr19	13397750	CACNA1A	
cg18854735	chr1	1822972	GNB1	Island
cg21793437	chr12	2734591	CACNA1C	
cg26999577	chr12	2340831	CACNA1C	
cg23264278	chr19	19051482	HOMER3	
cg17038626	chr12	2435931	CACNA1C	
cg20740024	chr11	35303612	SLC1A2	OpenSea
cg17867243	chr15	42371653	PLA2G4D	Island
cg23524436	chr12	2161437	CACNA1C	
cg24603152	chr1	84543539	PRKACB	
cg19742341	chr11	70385301	SHANK2	
cg17500055	chr1	235805560	GNG4	
cg17483139	chr1	1822401	GNB1	
cg19356346	chr12	100749441	SLC17A8	
cg09169283	chr17	72856452	GRIN2C	Island
cg04142017	chr1	53558596	SLC1A7	Island
cg22188571	chr6	34074955	GRM4	OpenSea
cg09001143	chr16	9857475	GRIN2A	
cg03641740	chr4	102087758	PPP3CA	
cg13729891	chr17	7108468	DLG4	
cg25766748	chr19	13614882	CACNA1A	
cg00380835	chr19	51165752	SHANK1	Island
cg12321149	chr20	57427426	GNAS	
cg07748847	chr12	14124857	GRIN2B	OpenSea
cg08987995	chr3	4788106	ITPR1	
cg06456864	chr6	34101399	GRM4	
cg21988465	chr20	57429277	GNAS	
cg10961484	chr11	120530860	GRIK4	OpenSea
cg04103918	chr17	4726687	PLD2	OpenSea
cg10126715	chr1	37500195	GRIK3	Island
cg16875863	chr19	48947572	GRIN2D	
cg24203465	chr20	57425986	GNASAS	
cg10031793	chr12	2734257	CACNA1C	
cg24791862	chr7	126893237	GRM8	Island
cg00014104	chr22	22220367	MAPK1	
cg26560871	chr6	146348616	GRM1	
cg15844381	chr19	14228577	PRKACA	Island
cg19572362	chr1	110091012	GNAI3	N_Shore
cg19466160	chr17	7117160	DLG4	
cg24794531	chr3	142443837	TRPC1	Island

cg24384034	chr11	70858463	SHANK2	OpenSea
cg23638849	chr11	70477184	SHANK2	
cg09262171	chr16	4140361	ADCY9	OpenSea
cg08193910	chr19	54410103	PRKCG	Island
cg27083019	chr19	49945958	SLC17A7	N_Shore
cg19296354	chr20	57415697	GNASAS	
cg07028661	chr21	31312905	GRIK1	
cg00536080	chr19	13409931	CACNA1A	
cg09447435	chr6	101854480	GRIK2	
cg00791406	chr12	14118880	GRIN2B	OpenSea
cg17414107	chr20	57427830	GNAS	
cg14395444	chr12	2800182	CACNA1C	
cg18689402	chr3	4630986	ITPR1	
cg02771299	chr6	33609240	ITPR3	OpenSea
cg10901633	chr3	4814459	ITPR1	
cg12216470	chr19	51200481	SHANK1	S_Shore
cg00056257	chr3	6901652	GRM7	
cg27552955	chr3	142442915	TRPC1	Island
cg04865531	chr22	51159147	SHANK3	Island
cg00610508	chr16	4013337	ADCY9	N_Shore
cg24678505	chr12	56882429	GLS2	Island
cg16200584	chr11	62473981	BSCL2	
cg14090219	chr19	13347085	CACNA1A	
cg23666299	chr6	102326919	GRIK2	
cg19512268	chr6	146348901	GRM1	
cg07067241	chr3	142442711	TRPC1	Island
cg05745748	chr6	33996013	GRM4	Island
cg01708648	chr11	64023044	PLCB3	N_Shelf
cg16281600	chr5	152869431	GRIA1	
cg08805338	chr10	75255936	PPP3CB	
cg23054925	chr1	84972704	SPATA1	
cg03490157	chr3	6906061	GRM7	
cg03389717	chr6	33601269	ITPR3	Island
cg01273580	chr19	49946108	SLC17A7	N_Shore
cg05120716	chr16	23881993	PRKCB	
cg17191518	chr15	52468850	GNB5	
cg14232870	chr11	70458782	SHANK2	
cg12211091	chr19	2512999	GNG7	N_Shore
cg18801906	chr11	70584165	SHANK2	OpenSea
cg07790747	chr16	56266223	GNAO1	
cg27262796	chr20	57426858	GNAS	
cg17969540	chr19	48908179	GRIN2D	Island
cg14910395	chr19	48941986	GRIN2D	N_Shelf
cg22364668	chr19	49944826	SLC17A7	N_Shore
cg27340723	chr16	4061608	ADCY9	OpenSea
cg14574037	chr2	155553986	KCNJ3	N_Shore
cg04798490	chr11	70517045	SHANK2	OpenSea
cg11244758	chr20	57463900	GNAS	

cg06869158	chr19	48900058	GRIN2D	N_Shore
cg05119467	chr1	1765440	GNB1	OpenSea
cg11480267	chr20	57463503	GNAS	
cg26362197	chr19	48948005	GRIN2D	
cg20698170	chr15	42120362	JMJD7	
cg17696044	chr11	70449316	SHANK2	
cg16204066	chr6	33638922	ITPR3	OpenSea
cg23868250	chr11	120764552	GRIK4	OpenSea
cg03837903	chr20	57464000	GNAS	
cg17069533	chr17	64657833	PRKCA	OpenSea
cg14564778	chr20	57427556	GNAS	
cg13756965	chr11	70917458	SHANK2	OpenSea
cg07774177	chr15	42387287	PLA2G4D	OpenSea
cg18151275	chr12	56873616	GLS2	OpenSea
cg17868128	chr17	64383859	PRKCA	OpenSea
cg03989758	chr5	36662950	SLC1A3	
cg16567056	chr15	40599985	PLCB2	
cg04153604	chr17	7099823	DLG4	
cg20326682	chr17	64345488	PRKCA	OpenSea
cg04077795	chr16	9857925	GRIN2A	
cg11067712	chr11	105850644	GRIA4	
cg14420982	chr9	80647609	GNAQ	S_Shore
cg23119809	chr17	72839750	GRIN2C	Island
cg04084618	chr5	36607065	SLC1A3	
cg06532779	chr19	1003622	GRIN3B	Island
cg07751222	chr4	102268245	PPP3CA	
cg08861556	chr16	850614	GNG13	Island
cg22857947	chr19	14225039	PRKACA	
cg15623249	chr19	48896947	GRIN2D	Island
cg24481035	chr19	54388422	PRKCG	S_Shelf
cg25423752	chr5	178422415	GRM6	S_Shore
cg23143233	chr20	57465864	GNAS	
cg19885037	chr3	4762242	ITPR1	
cg03357547	chr19	51165207	SHANK1	N_Shore
cg07843390	chr19	2541015	GNG7	Island
cg25228562	chr17	64718121	PRKCA	OpenSea
cg17300307	chr2	191744975	GLS	N_Shore
cg15128801	chr1	68202053	GNG12	OpenSea
cg25326570	chr20	57426757	GNAS	
cg02620388	chr7	100271115	GNB2	Island
cg00091960	chr7	126829514	GRM8	
cg11407328	chr17	64759926	PRKCA	OpenSea
cg11836212	chr1	37447865	GRIK3	OpenSea
cg17885091	chr1	68299057	GNG12	
cg26279745	chr14	24801970	ADCY4	Island
cg09722397	chr17	72855943	GRIN2C	
cg13715127	chr17	72856825	GRIN2C	Island
cg08779207	chr15	40586496	PLCB2	S_Shelf

cg18423469	chr18	3726858	DLGAP1	
cg06922606	chr16	10274632	GRIN2A	
cg13344206	chr6	34051016	GRM4	OpenSea
cg09239744	chr16	10276580	GRIN2A	
cg16707895	chr19	13394116	CACNA1A	
cg15114105	chr19	49944820	SLC17A7	N_Shore
cg08074971	chr16	850562	GNG13	Island
cg14746605	chr11	35374336	SLC1A2	OpenSea
cg08870587	chr11	70455278	SHANK2	
cg18352793	chr22	22210625	MAPK1	
cg11985287	chr19	2556805	GNG7	S_Shore
cg15814923	chr19	14228610	PRKACA	Island
cg14285012	chr9	104357967	PPP3R2	
cg06065549	chr20	57427443	GNAS	
cg20259256	chr3	142443266	TRPC1	
cg15160445	chr20	57426749	GNAS	
cg14898177	chr12	2486634	CACNA1C	
cg12190341	chr17	72838819	GRIN2C	Island
cg20152382	chr17	64783099	PRKCA	
cg12413242	chr12	2292890	CACNA1C	
cg06525750	chr2	25141152	ADCY3	N_Shore
cg27109030	chr19	2702898	GNG7	Island
cg03945800	chr16	4165515	ADCY9	Island
cg15985106	chr12	6954791	GNB3	OpenSea
cg23982607	chr1	1823379	GNB1	S_Shore
cg24131262	chr3	4549756	ITPR1	
cg24889366	chr16	850646	GNG13	Island
cg05693127	chr6	33643684	ITPR3	OpenSea
cg06500096	chr19	13405582	CACNA1A	
cg00940140	chr20	57480494	GNAS	
cg04677683	chr20	57426743	GNAS	
cg26204322	chr11	64018687	PLCB3	Island
cg08994082	chr19	2525459	GNG7	Island
cg24617313	chr20	57427146	GNAS	
cg01347776	chr3	179121836	GNB4	OpenSea
cg24036292	chr11	70416269	SHANK2	
cg23816431	chr3	171524969	PLD1	
cg02084729	chr6	33589691	ITPR3	Island
cg27567416	chr16	4117281	ADCY9	OpenSea
cg15355952	chr5	36662829	SLC1A3	
cg00652727	chr1	235812198	GNG4	
cg13851211	chr16	50321678	ADCY7	OpenSea
cg01344243	chr16	9855280	GRIN2A	
cg11661914	chr12	49180849	ADCY6	N_Shore
cg08111661	chr12	2223540	CACNA1C	
cg11227541	chr1	84972317	GNG5	
cg22023664	chr19	2619608	GNG7	N_Shelf
cg06324048	chr20	57427103	GNAS	

cg05558390	chr20	57415377	GNASAS	
cg05795849	chr3	4794082	ITPR1	
cg19696388	chr19	19042720	HOMER3	
cg11293016	chr19	54402647	PRKCG	S_Shore
cg24676071	chr7	45613410	ADCY1	Island
cg02374107	chr16	4163907	ADCY9	N_Shore
cg18982286	chr22	51136325	SHANK3	S_Shore
cg03303857	chr11	120619307	GRIK4	OpenSea
cg06716686	chr3	4535154	ITPR1	
cg26811638	chr20	57427493	GNAS	
cg24226238	chr11	120530774	GRIK4	OpenSea
cg07964163	chr20	57413417	GNASAS	
cg00578437	chr22	22217249	MAPK1	
cg09604333	chr20	57465125	GNAS	
cg04266169	chr1	1822862	GNB1	Island
cg18554395	chr19	2555791	GNG7	Island
cg05627987	chr19	51220286	SHANK1	Island
cg20098420	chr22	51155589	SHANK3	N_Shelf
cg26752663	chr2	25142016	ADCY3	
cg03626208	chr12	2443169	CACNA1C	
cg07582829	chr19	54408440	PRKCG	N_Shore
cg17174980	chr12	14109514	GRIN2B	OpenSea
cg08091561	chr20	57426425	GNAS	
cg11207372	chr11	70385365	SHANK2	
cg01833923	chr17	47286719	GNGT2	
cg16399393	chr11	70368768	SHANK2	
cg13631572	chr14	24803903	ADCY4	Island
cg08370077	chr16	851288	GNG13	S_Shore
cg16916688	chr6	34101441	GRM4	
cg15908975	chr7	126698829	MIR592	
cg20788479	chr3	179169536	GNB4	Island
cg10622236	chr15	42120082	JMJD7	
cg25229306	chr20	57426374	GNAS	
cg03630683	chr3	4534997	ITPR1	
cg25594486	chr19	51165441	SHANK1	Island
cg12345953	chr4	102207435	PPP3CA	
cg02858053	chr1	84971927	SPATA1	
cg02371119	chr3	171527346	PLD1	
cg12079381	chr21	31310920	GRIK1	
cg07938763	chr19	2516966	GNG7	S_Shelf
cg06887224	chr5	7399037	ADCY2	S_Shelf
cg17841572	chr20	57426368	GNAS	
cg24778538	chr12	14131975	GRIN2B	N_Shelf
cg11658986	chr12	49177605	ADCY6	
cg24146288	chr15	42302342	PLA2G4E	OpenSea
cg12463346	chr4	102268854	PPP3CA	
cg03263685	chr2	68480160	PPP3R1	S_Shore
cg13885159	chr11	62473858	BSCL2	

cg17298543	chr7	79802016	GNAI1	OpenSea
cg06868991	chr11	70774323	SHANK2	OpenSea
cg15333818	chr12	46766724	SLC38A2	S_Shore
cg06465194	chr11	120531034	GRIK4	OpenSea
cg01935096	chr11	70391215	SHANK2	
cg21032008	chr3	7724717	GRM7	
cg12743970	chr17	64536520	PRKCA	OpenSea
cg08966293	chr16	30134858	MAPK3	
cg17509220	chr19	13617012	CACNA1A	
cg21725954	chr6	146348890	GRM1	
cg04708601	chr6	101880078	GRIK2	
cg09845015	chr11	70458994	SHANK2	
cg21938532	chr20	57426931	GNAS	
cg02847220	chr3	171523157	PLD1	
cg22827707	chr6	34100899	GRM4	OpenSea
cg24661595	chr11	70456848	SHANK2	
cg07008386	chr20	8113630	PLCB1	
cg12446629	chr8	132052044	ADCY8	N_Shore
cg22323942	chr19	2543655	GNG7	Island
cg27122965	chr11	70882053	SHANK2	OpenSea
cg13948330	chr11	70559064	SHANK2	OpenSea
cg23848889	chr11	70455304	SHANK2	
cg20325479	chr7	100271106	GNB2	Island
cg14792912	chr3	51742779	GRM2	
cg20152891	chr19	49944506	SLC17A7	Island
cg06044900	chr20	57467811	GNAS	
cg01360067	chr10	75256027	PPP3CB	
cg12451177	chr22	22222048	MAPK1	
cg14120436	chr15	52483498	GNB5	OpenSea
cg18949315	chr20	57418015	GNASAS	
cg04908625	chr3	123166882	ADCY5	Island
cg04107939	chr3	171520494	PLD1	
cg26226650	chr3	50276265	GNAI2	S_Shore
cg03601797	chr2	155556321	KCNJ3	S_Shore
cg10241462	chr2	191746790	GLS	S_Shore
cg04017672	chr1	182359056	GLUL	
cg16377872	chr19	15084823	SLC1A6	OpenSea
cg20231694	chr11	70691944	SHANK2	OpenSea
cg13824515	chr9	140047122	GRIN1	
cg05087008	chr11	105483680	GRIA4	
cg05035616	chr16	56374523	GNAO1	
cg11343713	chr7	45749313	ADCY1	OpenSea
cg06840723	chr15	52484492	GNB5	OpenSea
cg22990158	chr14	24802150	ADCY4	Island
cg01255513	chr19	13365923	CACNA1A	
cg26670249	chr19	14223997	PRKACA	
cg20240931	chr19	2613894	GNG7	N_Shore
cg12043631	chr11	70643814	SHANK2	OpenSea

cg23465427	chr17	7111429	DLG4	
cg26424956	chr6	34101526	GRM4	OpenSea
cg19006947	chr11	64034861	PLCB3	Island
cg08266286	chr2	25141901	ADCY3	
cg09444818	chr4	102097226	PPP3CA	
cg23325230	chr7	100272578	GNB2	Island
cg07783800	chr1	235803662	GNG4	
cg13300911	chr15	42119951	JMJD7	
cg01846046	chr11	64034019	PLCB3	N_Shore
cg03503758	chr19	2614104	GNG7	Island
cg24737505	chr11	70564116	SHANK2	S_Shore
cg25963822	chr7	100270831	GNB2	N_Shore
cg03697708	chr19	13617549	CACNA1A	
cg26265279	chr3	53566044	CACNA1D	
cg14099468	chr1	235814814	GNG4	
cg08076125	chr6	146350230	GRM1	
cg14943539	chr20	57420942	GNASAS	
cg14295482	chr19	2555717	GNG7	N_Shore
cg25781595	chr19	48918712	GRIN2D	N_Shore
cg18571531	chr11	70477192	SHANK2	
cg13399816	chr1	68299468	GNG12	S_Shore
cg02664157	chr19	2702877	GNG7	Island
cg02423534	chr12	49160180	ADCY6	
cg25335435	chr11	22399705	SLC17A6	OpenSea
cg23484981	chr20	57426626	GNAS	
cg06344265	chr11	120530973	GRIK4	OpenSea
cg11264539	chr11	62474940	BSCL2	
cg06836849	chr12	100751051	SLC17A8	
cg14843888	chr3	53530247	CACNA1D	
cg16582156	chr1	110091224	GNAI3	
cg23332732	chr12	26986274	ITPR2	Island
cg25556008	chr19	2525384	GNG7	Island
cg20668321	chr5	153192843	GRIA1	
cg14317712	chr9	140034900	GRIN1	
cg25316853	chr5	36606347	SLC1A3	
cg21810373	chr11	70917159	SHANK2	OpenSea
cg00536924	chr7	93551004	GNG11	OpenSea
cg21330323	chr20	57414596	GNASAS	
cg20915897	chr7	45717588	ADCY1	OpenSea
cg13469748	chr1	84971910	SPATA1	
cg02063520	chr6	102516590	GRIK2	
cg03335128	chr1	1731415	GNB1	OpenSea
cg02046017	chr11	70707406	SHANK2	OpenSea
cg15093766	chr17	64408569	PRKCA	OpenSea
cg03140521	chr1	68299388	GNG12	S_Shore
cg05313261	chr16	30134350	MAPK3	
cg03836615	chr3	4856096	ITPR1	
cg18229071	chr19	2695245	GNG7	OpenSea

cg17151604	chr10	75197928	PPP3CB	
cg17202839	chr17	64685036	PRKCA	OpenSea
cg09902254	chr11	70858237	SHANK2	OpenSea
cg15631127	chr20	57426580	GNAS	
cg08481112	chr19	2544100	GNG7	S_Shore
cg00233948	chr5	36619356	SLC1A3	
cg06111374	chr12	14109584	GRIN2B	OpenSea
cg06171406	chr16	4050400	ADCY9	OpenSea
cg24220046	chr19	51171640	SHANK1	Island
cg06223539	chr11	70517374	SHANK2	OpenSea
cg14975881	chr19	54389945	PRKCG	N_Shelf
cg07237830	chr11	62474725	BSCL2	
cg23055496	chr3	6906371	GRM7	
cg14167033	chr11	70424559	SHANK2	
cg08462108	chr11	70680470	SHANK2	OpenSea
cg02098786	chr14	24801794	ADCY4	Island
cg04421162	chr19	54406293	PRKCG	N_Shelf
cg26648054	chr17	7117995	DLG4	
cg22294773	chr19	2606054	GNG7	N_Shore
cg11264635	chr19	15083868	SLC1A6	OpenSea
cg26739975	chr4	158144318	GRIA2	
cg09336589	chr17	7107939	DLG4	
cg25090051	chr20	57414059	GNASAS	
cg01588464	chr1	182360063	GLUL	
cg10504751	chr16	56390830	GNAO1	OpenSea
cg15129608	chr19	54393153	PRKCG	Island
cg01895374	chr17	64536954	PRKCA	OpenSea
cg06697294	chr19	54385412	PRKCG	Island
cg09980522	chr11	105481802	GRIA4	
cg23982812	chr12	14004950	GRIN2B	OpenSea
cg16175911	chr12	26985133	ITPR2	N_Shore
cg15812599	chr11	70849065	SHANK2	OpenSea
cg01817393	chr20	57427642	GNAS	
cg20830447	chr12	46764929	SLC38A2	N_Shore
cg12198334	chr11	70692032	SHANK2	OpenSea
cg07105596	chr20	57427472	GNAS	
cg23399933	chr4	102112217	PPP3CA	
cg09340615	chr16	4021030	ADCY9	S_Shelf
cg14418176	chr2	25050403	ADCY3	OpenSea
cg17838127	chr8	22298935	PPP3CC	Island
cg24081764	chr19	54402116	PRKCG	Island
cg13650938	chr19	2579075	GNG7	Island
cg09885502	chr20	57463991	GNAS	
cg13707945	chr3	4714992	ITPR1	
cg13559773	chr19	48562267	PLA2G4C	
cg21409965	chr1	37283904	GRIK3	OpenSea
cg03217795	chr16	23847556	PRKCB	
cg21429394	chr12	100750899	SLC17A8	

cg15972294	chr3	50273096	GNAI2	N_Shore
cg25900614	chr7	126079083	GRM8	
cg24039816	chr19	51220098	SHANK1	Island
cg12863967	chr7	93534920	GNGT1	OpenSea
cg22218695	chr7	126446519	GRM8	
cg02090654	chr7	126698344	MIR592	
cg07793724	chr1	53609371	SLC1A7	OpenSea
cg01729401	chr1	1750560	GNB1	OpenSea
cg06088782	chr11	70563839	SHANK2	Island
cg18935491	chr20	57425979	GNASAS	
cg04583195	chr3	179165115	GNB4	N_Shelf
cg05800983	chr6	34102530	GRM4	OpenSea
cg24591182	chr11	64019217	PLCB3	Island
cg02676523	chr16	4027674	ADCY9	Island
cg21233003	chr9	140057464	GRIN1	
cg02415992	chr16	10102278	GRIN2A	
cg04132853	chr20	57414039	GNASAS	
cg01187464	chr16	50351302	ADCY7	OpenSea
cg04153722	chr17	64783041	PRKCA	
cg04779428	chr20	57463355	GNAS	
cg12568707	chr19	19042904	HOMER3	
cg15692593	chr6	101993140	GRIK2	
cg12282391	chr12	2162491	CACNA1C	
cg18225409	chr11	70713375	SHANK2	OpenSea
cg13680388	chr20	57471844	GNAS	
cg06563300	chr12	100750811	SLC17A8	
cg18150383	chr19	49933217	SLC17A7	N_Shore
cg21997766	chr17	72846113	GRIN2C	N_Shelf
cg09822192	chr14	24801191	ADCY4	N_Shore
cg16848624	chr7	45614290	ADCY1	Island
cg20721022	chr19	54386355	PRKCG	S_Shore
cg25322847	chr7	45617892	ADCY1	S_Shelf
cg24171047	chr17	64765921	PRKCA	OpenSea
cg00041368	chr18	3879131	DLGAP1	N_Shore
cg13231680	chr3	7693146	GRM7	
cg08992229	chr7	126866923	GRM8	
cg21116900	chr12	100750760	SLC17A8	
cg03969219	chr19	2611456	GNG7	Island
cg18753811	chr12	2162232	CACNA1C	
cg14637685	chr12	2411116	CACNA1C	
cg09248655	chr19	48897955	GRIN2D	S_Shore
cg25556841	chr19	2511263	GNG7	N_Shore
cg12872693	chr3	179168798	GNB4	Island
cg18399935	chr3	6906994	GRM7	
cg08134671	chr19	2542837	GNG7	N_Shore
cg12246156	chr17	64522604	PRKCA	OpenSea
cg13396607	chr6	102055059	GRIK2	
cg04169369	chr16	4053199	ADCY9	OpenSea

cg14416930	chr17	64498178	PRKCA	OpenSea
cg16395366	chr1	53558245	SLC1A7	N_Shore
cg27491190	chr12	46767943	SLC38A2	S_Shore
cg12204773	chr17	7123253	DLG4	
cg25293328	chr19	2611690	GNG7	S_Shore
cg22090419	chr3	53844172	CACNA1D	
cg21157507	chr11	70830058	SHANK2	OpenSea
cg02647408	chr11	88241594	GRM5	
cg04696980	chr19	2586206	GNG7	N_Shelf
cg13934625	chr15	52472770	GNB5	
cg17354190	chr17	72856064	GRIN2C	Island
cg01871907	chr19	2703055	GNG7	S_Shore
cg01035815	chr6	33600828	ITPR3	N_Shore
cg21961771	chr12	100750652	SLC17A8	
cg24640697	chr1	84970057	GNG5	N_Shore
cg05780228	chr11	70713608	SHANK2	OpenSea
cg13052954	chr1	37467416	GRIK3	OpenSea
cg02959759	chr12	2801584	CACNA1C	
cg27356165	chr19	2613933	GNG7	Island
cg16669395	chr16	10208417	GRIN2A	
cg16182691	chr7	79762956	GNAI1	N_Shore
cg14111579	chr19	48614090	PLA2G4C	
cg26314755	chr19	42550298	GRIK5	S_Shelf
cg24190415	chr11	35441012	SLC1A2	
cg21970929	chr5	36608598	SLC1A3	
cg20000940	chr14	52327486	GNG2	
cg14176797	chr20	57426801	GNAS	
cg03657031	chr19	54385215	PRKCG	N_Shore
cg24058407	chr20	57428282	GNAS	
cg00007326	chr19	13366046	CACNA1A	
cg23323297	chr19	51195418	SHANK1	N_Shelf
cg04987335	chr11	35288779	SLC1A2	OpenSea
cg09241929	chr20	57465560	GNAS	
cg22749173	chr19	2614039	GNG7	Island
cg26968767	chr17	72843650	GRIN2C	S_Shelf
cg27270541	chr19	48614177	PLA2G4C	
cg16279290	chr11	70368624	SHANK2	
cg06485596	chr16	24112658	PRKCB	
cg25419928	chr6	33656793	ITPR3	S_Shore
cg11706467	chr2	155554707	KCNJ3	Island
cg06986989	chr1	235802839	GNG4	
cg16361867	chr11	120581355	GRIK4	OpenSea
cg11663780	chr19	1001892	GRIN3B	N_Shore
cg01242196	chr6	33990181	GRM4	OpenSea
cg23123694	chr12	46766543	SLC38A2	
cg07456314	chr17	4708968	PLD2	N_Shore
cg23808301	chr17	4710015	PLD2	Island
cg04533189	chr17	64298763	PRKCA	Island

cg09583957	chr20	57428315	GNAS	
cg16644457	chr11	22359480	SLC17A6	N_Shelf
cg13762474	chr15	42371808	PLA2G4D	Island
cg21024916	chr3	4535815	ITPR1	
cg11265916	chr22	22221056	MAPK1	
cg25983305	chr8	22298586	PPP3CC	Island
cg01749530	chr10	75255289	PPP3CB	
cg04455869	chr5	7663853	ADCY2	OpenSea
cg18224653	chr20	57426979	GNAS	
cg19592829	chr20	57426215	GNAS	
cg11464074	chr7	126417126	GRM8	
cg15863841	chr3	171430173	PLD1	
cg18411237	chr11	70653173	SHANK2	OpenSea
cg08848088	chr1	235714526	GNG4	
cg04586622	chr2	25135609	ADCY3	OpenSea
cg00848945	chr12	2800919	CACNA1C	
cg11647651	chr17	4710373	PLD2	Island
cg08901242	chr19	15083667	SLC1A6	OpenSea
cg15131024	chr11	70338408	SHANK2	
cg00495303	chr18	3771110	DLGAP1	
cg05944877	chr16	24197863	PRKCB	
cg26429499	chr11	70563792	SHANK2	Island
cg15154232	chr19	48615306	PLA2G4C	
cg24591824	chr12	2762732	CACNA1C	
cg27173374	chr14	52413159	GNG2	OpenSea
cg05564552	chr15	42120091	JMJD7	
cg14616584	chr1	37388124	GRIK3	OpenSea
cg16815991	chr12	14133129	GRIN2B	N_Shore
cg17959824	chr11	70391706	SHANK2	
cg04037585	chr16	56231292	GNAO1	
cg09433558	chr3	171412917	PLD1	
cg16685860	chr17	4710619	PLD2	Island
cg23019936	chr12	13903266	GRIN2B	OpenSea
cg13280108	chr11	70398751	SHANK2	
cg13070193	chr7	45613752	ADCY1	Island
cg13878641	chr1	110090951	GNAI3	N_Shore
cg04019914	chr20	57463357	GNAS	
cg17895496	chr15	42449716	PLA2G4F	
cg17074573	chr22	51165537	SHANK3	N_Shelf
cg04355871	chr11	64022825	PLCB3	N_Shelf
cg14995148	chr5	36683916	SLC1A3	
cg24646457	chr15	42360292	PLA2G4D	OpenSea
cg14003231	chr6	33640908	ITPR3	OpenSea
cg10538151	chr9	140033364	GRIN1	
cg18379295	chr14	52326155	GNG2	OpenSea
cg15144016	chr3	51749782	GRM2	
cg08413366	chr10	75255930	PPP3CB	
cg23475725	chr12	2734205	CACNA1C	

cg02946850	chr7	126882944	GRM8	
cg06210447	chr11	70601842	SHANK2	OpenSea
cg19796640	chr17	72848197	GRIN2C	Island
cg27068206	chr11	70559053	SHANK2	OpenSea
cg08021532	chr16	50321878	ADCY7	
cg08436756	chr11	70781118	SHANK2	OpenSea
cg13455717	chr1	235814365	GNG4	
cg14331853	chr9	140054850	GRIN1	
cg09403559	chr16	56334857	GNAO1	
cg18420143	chr17	7123125	DLG4	
cg04122657	chr16	4014295	ADCY9	N_Shore
cg04898487	chr16	10272607	GRIN2A	
cg03282345	chr19	49934577	SLC17A7	Island
cg13148511	chr17	4710380	PLD2	Island
cg20091384	chr19	2700927	GNG7	N_Shore
cg03315058	chr11	62476542	BSCL2	
cg09947844	chr16	4163819	ADCY9	N_Shore
cg16124935	chr11	70559616	SHANK2	OpenSea
cg01332711	chr15	42120681	JMJD7-PLA2G4B	
cg14082123	chr15	42367977	PLA2G4D	N_Shelf
cg17986880	chr7	79848290	GNAI1	OpenSea
cg21163960	chr11	35441777	SLC1A2	Island
cg19781472	chr12	56883202	GLS2	S_Shore
cg27014608	chr16	4166952	ADCY9	Island
cg22934516	chr11	35413951	SLC1A2	OpenSea
cg25439807	chr18	3771151	DLGAP1	
cg11112257	chr11	88781135	GRM5	
cg10573143	chr11	70628992	SHANK2	OpenSea
cg01025883	chr16	23867088	PRKCB	
cg17818798	chr19	14228473	PRKACA	
cg24446178	chr12	100750702	SLC17A8	
cg12986110	chr19	48551504	PLA2G4C	
cg14100184	chr16	851298	GNG13	S_Shore
cg27591117	chr20	8113191	PLCB1	
cg22989942	chr20	57426950	GNAS	
cg13574337	chr16	4016720	ADCY9	Island
cg02740128	chr17	7123860	DLG4	
cg09257092	chr12	26986805	ITPR2	S_Shore
cg14907788	chr19	2555976	GNG7	Island
cg25399541	chr7	45622395	ADCY1	OpenSea
cg06401532	chr16	24220008	PRKCB	
cg08942894	chr15	83563792	HOMER2	
cg06293172	chr2	25045211	ADCY3	OpenSea
cg22335074	chr11	70733258	SHANK2	OpenSea
cg06772874	chr1	110090953	GNAI3	N_Shore
cg26968025	chr17	64519943	PRKCA	OpenSea
cg11637718	chr16	4029254	ADCY9	Island
cg01903557	chr3	179169602	GNB4	S_Shore

cg03510435	chr12	14094558	GRIN2B	OpenSea
cg07838205	chr1	110091179	GNAI3	Island
cg01140008	chr6	34002114	GRM4	N_Shore
cg03679394	chr11	70516997	SHANK2	OpenSea
cg02409125	chr7	126889555	GRM8	N_Shore
cg13804196	chr9	71628906	PRKACG	Island
cg26645082	chr11	70563264	SHANK2	N_Shore
cg02520816	chr16	4056403	ADCY9	OpenSea
cg19548470	chr18	3880510	DLGAP1	S_Shore
cg23815646	chr8	131961143	ADCY8	OpenSea
cg02615582	chr19	49939549	SLC17A7	N_Shore
cg10815152	chr6	102098727	GRIK2	
cg16560679	chr7	100276684	GNB2	S_Shelf
cg03511974	chr1	53568259	SLC1A7	OpenSea
cg08263099	chr19	54410160	PRKCG	Island
cg02660823	chr19	54410305	PRKCG	N_Shore
cg14022022	chr9	140055728	GRIN1	
cg01286319	chr19	2695343	GNG7	OpenSea
cg16358215	chr11	70455662	SHANK2	
cg22405973	chr22	22222028	MAPK1	
cg02591871	chr19	14228565	PRKACA	Island
cg21615915	chr6	102295679	GRIK2	
cg26568075	chr1	1718809	GNB1	OpenSea
cg14121185	chr17	64488849	PRKCA	OpenSea
cg20779373	chr1	37428969	GRIK3	OpenSea
cg09209803	chr6	33588932	ITPR3	Island
cg04338055	chr19	1000955	GRIN3B	Island
cg05577548	chr11	70666748	SHANK2	Island
cg02780849	chr1	235814163	GNG4	
cg11895615	chr12	2224518	CACNA1C	
cg04747226	chr11	105481319	GRIA4	
cg01697794	chr17	7117125	DLG4	
cg01943657	chr4	102268799	PPP3CA	
cg01900555	chr12	6948846	GNB3	
cg01192061	chr11	70368264	SHANK2	
cg05297437	chr20	57471672	GNAS	
cg02748316	chr3	50273710	GNAI2	
cg16728539	chr12	2451169	CACNA1C	
cg23425324	chr12	26986193	ITPR2	Island
cg25193077	chr1	235812109	GNG4	
cg13213810	chr22	51158720	SHANK3	Island
cg17702518	chr7	100271260	GNB2	Island
cg10583180	chr6	101851354	GRIK2	
cg01962496	chr5	78809740	HOMER1	Island
cg19367232	chr2	68478649	PPP3R1	N_Shore
cg07391392	chr5	7826900	ADCY2	OpenSea
cg07546293	chr16	851255	GNG13	S_Shore
cg16737409	chr20	57428366	GNAS	

cg03551401	chr8	132051228	ADCY8	N_Shore
cg09066361	chr7	126890254	GRM8	N_Shore
cg10768900	chr11	70557881	SHANK2	OpenSea
cg03830585	chr3	4536777	ITPR1	
cg02993882	chr16	4043463	ADCY9	OpenSea
cg16993684	chr20	57466131	GNAS	
cg25350198	chr1	84971850	SPATA1	
cg22304522	chr8	131914037	ADCY8	OpenSea
cg02799411	chr3	4794061	ITPR1	
cg06693667	chr20	57426570	GNAS	
cg06170425	chr16	4164087	ADCY9	N_Shore
cg03866831	chr16	9849427	GRIN2A	
cg12965344	chr19	48898160	GRIN2D	
cg16862315	chr17	7123138	DLG4	
cg00202454	chr15	42371886	PLA2G4D	S_Shore
cg05514043	chr9	140040822	GRIN1	
cg09103960	chr16	56225504	LOC283856	
cg10243075	chr19	13615439	CACNA1A	
cg08290212	chr11	62473659	BSCL2	
cg08587534	chr20	57427503	GNAS	
cg26739691	chr7	45637270	ADCY1	OpenSea
cg15591578	chr19	51219167	SHANK1	N_Shore
cg18870258	chr20	9460935	PLCB4	
cg10168763	chr16	4166767	ADCY9	Island
cg22741626	chr20	57463265	GNAS	
cg09053902	chr16	4034298	ADCY9	OpenSea
cg19815589	chr11	70709062	SHANK2	OpenSea
cg00980784	chr17	47287577	ABI3	
cg21151432	chr2	25142229	ADCY3	N_Shore
cg14583606	chr9	4490315	SLC1A1	Island
cg17540496	chr3	53845930	CACNA1D	
cg05648629	chr16	4162203	ADCY9	N_Shelf
cg00426976	chr22	51140977	SHANK3	N_Shore
cg11023668	chr2	25095040	ADCY3	OpenSea
cg16102063	chr8	22298240	PPP3CC	Island
cg25308079	chr20	57463763	GNAS	
cg16835502	chr17	4710020	PLD2	Island
cg10599507	chr6	33653337	ITPR3	N_Shelf
cg23249369	chr20	57426759	GNAS	
cg05340495	chr14	52327368	GNG2	
cg25960479	chr11	88243569	GRM5	
cg14960282	chr1	37321669	GRIK3	OpenSea
cg05309239	chr20	57427017	GNAS	
cg25314445	chr1	1718835	GNB1	OpenSea
cg08240335	chr3	50273314	GNAI2	Island
cg00646241	chr11	70563878	SHANK2	Island
cg03344105	chr20	57426131	GNASAS	
cg03211327	chr15	52470919	GNB5	

cg03768297	chr15	52441108	GNB5	
cg16108726	chr11	70781009	SHANK2	OpenSea
cg13714844	chr9	114422486	GNG10	
cg04675204	chr16	10179771	GRIN2A	
cg04086239	chr16	24067174	PRKCB	
cg03962451	chr2	191754464	GLS	OpenSea
cg19365406	chr9	140043007	GRIN1	
cg07982896	chr19	13365938	CACNA1A	
cg10002103	chr12	46766730	SLC38A2	S_Shore
cg11070176	chr11	70489806	SHANK2	
cg19161850	chr22	22222040	MAPK1	
cg18375707	chr11	64034959	PLCB3	Island
cg17902551	chr12	2801061	CACNA1C	
cg05147077	chr5	36606601	SLC1A3	
cg03015368	chr16	10065222	GRIN2A	
cg18997188	chr20	57463270	GNAS	
cg26767990	chr20	57463615	GNAS	
cg13701180	chr19	2513436	GNG7	Island
cg02392737	chr16	4136367	ADCY9	OpenSea
cg16904585	chr16	10276119	GRIN2A	
cg00296378	chr12	49177153	ADCY6	
cg14560133	chr19	51199453	SHANK1	S_Shore
cg02775369	chr16	56316221	GNAO1	
cg17377463	chr19	48908557	GRIN2D	Island
cg02261541	chr16	4050315	ADCY9	OpenSea
cg09906922	chr19	14203252	PRKACA	
cg04106389	chr17	7117241	DLG4	
cg08894891	chr19	19040364	HOMER3	
cg19013391	chr3	123166774	ADCY5	Island
cg06622135	chr11	70474416	SHANK2	
cg17221095	chr7	45717651	ADCY1	OpenSea
cg05312962	chr19	2576254	GNG7	N_Shelf
cg20789595	chr3	123063477	ADCY5	OpenSea
cg24788034	chr19	2588241	GNG7	N_Shore
cg08161922	chr12	2163608	CACNA1C	
cg04498349	chr16	10274317	GRIN2A	
cg24867180	chr15	42120426	JMJD7-PLA2G4B	
cg17047106	chr8	131961479	ADCY8	OpenSea
cg05824594	chr12	2734503	CACNA1C	
cg16864295	chr3	171463753	PLD1	
cg24204556	chr22	22222030	MAPK1	
cg00672228	chr17	7123130	DLG4	
cg26632831	chr11	70935863	SHANK2	OpenSea
cg14847975	chr12	26986502	ITPR2	S_Shore
cg04507426	chr16	56229180	DKFZP434H168	
cg16418734	chr1	1720537	GNB1	OpenSea
cg17839611	chr17	47286802	ABI3	
cg24868926	chr1	182360594	GLUL	

cg08626004	chr19	2513687	GNG7	Island
cg20401058	chr20	57426240	GNAS	
cg05100017	chr6	34102222	GRM4	OpenSea
cg01047778	chr11	70584252	SHANK2	OpenSea
cg05684300	chr4	102267366	PPP3CA	
