# Supplementary material for: Differential DNA Methylation of the Serotonin Receptor Signaling and Glutamatergic Synapse Pathways in Adult Twins Born Preterm
Source: Genes (Basel). 2026 Jun 10;17(6):683. doi: 10.3390/genes17060683 (PMC13299586; doi:10.3390/genes17060683)
Supplement: Supplementary file 1 [file genes-17-00683-s001.zip › Supplementary Table S2 serotonin.pdf]

CpG	Chromosome	Position	Gene	CpG_Island_Context
cg27549720	chr1	19992167	HTR6	
cg06291867	chr10	92617162	HTR7	
cg23764129	chr11	113846017	HTR3A	
cg18023598	chr1	19992504	HTR6	Island
cg07664198	chr7	136553882	CHRM2	
cg01281175	chr5	175109114	HRH2	
cg00183186	chr6	78174065	HTR1B	Island
cg00903099	chr7	154862441	HTR5A	N_Shore
cg11615755	chr5	63257867	HTR1A	Island
cg19500607	chr5	148034319	HTR4	
cg24845274	chr7	136555697	CHRM2	
cg22059812	chr1	19992564	HTR6	Island
cg17805202	chr1	239937440	CHRM3	OpenSea
cg09386376	chr11	638939	DRD4	Island
cg12103152	chr6	78173200	HTR1B	Island
cg07102705	chr5	148033896	HTR4	
cg05717871	chr11	638507	DRD4	Island
cg07833420	chr6	87647147	HTR1E	
cg06531741	chr11	113775450	HTR3B	OpenSea
cg04427003	chr5	63257499	HTR1A	Island
cg08186362	chr20	60794868	HRH3	Island
cg08323651	chr7	136553855	CHRM2	
cg03909863	chr11	638404	DRD4	Island
cg24575234	chr7	136553884	CHRM2	
cg00840960	chr5	148034030	HTR4	
cg16280141	chr5	63257753	HTR1A	Island
cg17220584	chr5	148017424	HTR4	
cg24228819	chr7	136553868	CHRM2	
cg21566860	chr3	11242797	HRH1	
cg01257383	chr11	46408399	CHRM4	S_Shore
cg09640960	chr20	60794676	HRH3	Island
cg09863441	chr6	87647129	HTR1E	
cg14433983	chr11	636460	DRD4	Island
cg22471401	chr3	183824717	HTR3E	OpenSea
cg18019017	chr6	78173408	HTR1B	Island
cg03138127	chr2	231989146	PSMD1	
cg12825070	chr5	148033708	HTR4	
cg02266732	chr5	63257710	HTR1A	Island
cg25632105	chr7	136553728	CHRM2	
cg13077519	chr6	78174007	HTR1B	Island
cg05919907	chr5	175105350	HRH2	OpenSea
cg11298960	chr7	154862548	HTR5A	
cg03986968	chr15	34330894	AVEN	
cg00120810	chr3	11195317	HRH1	
cg12198176	chr22	23415316	RTDR1	
cg27615388	chr5	63257092	HTR1A	Island
cg10979181	chr7	136586606	CHRM2	

cg08614481	chr6	78173250	HTR1B	Island
cg00454577	chr6	87646972	HTR1E	N_Shore
cg23247337	chr3	11178285	HRH1	OpenSea
cg07212818	chr11	638076	DRD4	Island
cg08208133	chr11	113848161	HTR3A	
cg02389195	chr5	175088015	HRH2	S_Shelf
cg06825142	chr11	637170	DRD4	Island
cg05756489	chr10	92616870	HTR7	
cg05650628	chr11	62677384	CHRM1	OpenSea
cg14632899	chr11	62678618	CHRM1	OpenSea
cg21127286	chr7	136555154	CHRM2	
cg11702866	chr3	11267098	HRH1	
cg19503977	chr6	78173287	HTR1B	Island
cg04842426	chr7	136555777	CHRM2	
cg13102079	chr7	136554731	CHRM2	
cg20277670	chr5	175110375	HRH2	
cg20847733	chr7	136554160	CHRM2	
cg15602074	chr6	78173720	HTR1B	Island
cg24121172	chr20	60796414	HRH3	S_Shore
cg19764436	chr22	23413260	RTDR1	
cg03440850	chr11	46407440	CHRM4	Island
cg13450708	chr7	154862157	HTR5A	N_Shore
cg11438011	chr5	148033882	HTR4	
cg13256912	chr3	11211081	HRH1	
cg15662768	chr20	60795818	HRH3	Island
cg02150536	chr3	11302179	HRH1	
cg06969845	chr5	175084250	HRH2	N_Shore
cg14199144	chr11	113852043	HTR3A	
cg07043494	chr3	11293681	HRH1	
cg16929739	chr3	11178593	HRH1	OpenSea
cg02866106	chr7	136553110	CHRM2	
cg06961323	chr11	113775900	HTR3B	OpenSea
cg24607283	chr3	11302249	HRH1	
cg26321066	chr3	183769987	HTR3C	OpenSea
cg15368905	chr6	78172337	HTR1B	Island
cg00556112	chr11	637173	DRD4	Island
cg27579609	chr1	20003062	HTR6	N_Shelf
cg02701826	chr19	15903794	OR10H5	OpenSea
cg02928916	chr6	87647154	HTR1E	
cg17176676	chr22	23441402	RTDR1	
cg25058023	chr7	154863176	HTR5A	Island
cg07826387	chr1	240072501	CHRM3	OpenSea
cg10454514	chr7	136553778	CHRM2	
cg21286526	chr6	87646986	HTR1E	N_Shore
cg06020661	chr13	47472138	HTR2A	
cg20102280	chr13	47470793	HTR2A	
cg07318372	chr1	240071102	CHRM3	OpenSea
cg14458903	chr3	11203475	HRH1	

cg00902763	chr3	183750690	HTR3D	
cg16109381	chr15	34339800	CHRM5	OpenSea
cg04694812	chr5	63257554	HTR1A	Island
cg07162608	chr1	239828159	CHRM3	OpenSea
cg05157516	chr5	175085577	HRH2	Island
cg25763788	chr6	78172950	HTR1B	Island
cg09714615	chr5	148033068	HTR4	
cg12449682	chr5	175111543	HRH2	
cg10538202	chr7	154863338	HTR5A	S_Shore
cg01991150	chr11	46407677	CHRM4	S_Shore
cg01616529	chr11	638424	DRD4	Island
cg27092248	chr3	11178798	HRH1	
cg04434491	chr19	16058640	OR10H4	OpenSea
cg02960016	chr3	11192067	HRH1	N_Shelf
cg09662616	chr1	240071263	CHRM3	OpenSea
cg01620540	chr13	47472064	HTR2A	
cg08722720	chr15	34331557	AVEN	
cg02508664	chr6	87646738	HTR1E	N_Shore
cg00195561	chr11	46408584	CHRM4	S_Shore
cg21633143	chr7	154862021	HTR5A	Island
cg18593668	chr22	23411986	RTDR1	
cg01192538	chr13	47472050	HTR2A	
cg15092168	chr5	63257873	HTR1A	Island
cg04278702	chr6	87647399	HTR1E	
cg09623773	chr7	154863381	HTR5A	S_Shore
cg25368284	chr22	23438430	RTDR1	
cg03855291	chr11	639423	DRD4	Island
cg04200192	chr3	183749414	HTR3D	
cg12440040	chr6	87725662	HTR1E	OpenSea
cg01751188	chr22	23412225	RTDR1	
cg02236913	chr1	20005598	HTR6	Island
cg26043322	chr1	159507162	OR10J5	OpenSea
cg13593758	chr3	11178365	HRH1	OpenSea
cg12089079	chr13	47470350	HTR2A	
cg12583095	chr10	92618141	HTR7	
cg07963181	chr3	11195902	HRH1	
cg24539937	chr7	136633191	CHRM2	
cg06299284	chr11	636659	DRD4	Island
cg09297468	chr6	87647376	HTR1E	
cg12793238	chr2	231977049	HTR2B	
cg27447053	chr20	60795465	HRH3	Island
cg03024742	chr7	154863244	HTR5A	Island
cg04799838	chr5	63256926	HTR1A	Island
cg24134767	chr11	113845638	HTR3A	
cg03472798	chr6	87646930	HTR1E	N_Shore
cg26393112	chr11	46408262	CHRM4	S_Shore
cg11811391	chr1	23520083	HTR1D	OpenSea
cg25388738	chr1	240071723	CHRM3	OpenSea

cg15919431	chr5	147862506	HTR4	
cg08831077	chr3	11178745	HRH1	OpenSea
cg07630532	chr20	60795459	HRH3	Island
cg06251978	chr1	159507078	OR10J5	OpenSea
cg15068527	chr3	183817134	HTR3E	OpenSea
cg23757489	chr7	154862139	HTR5A	N_Shore
cg05327864	chr7	136554352	CHRM2	
cg11158819	chr3	183817853	HTR3E	OpenSea
cg04315863	chr15	34330588	AVEN	
cg12816057	chr5	148034206	HTR4	
cg09798090	chr13	47472140	HTR2A	
cg01459748	chr11	113817020	HTR3B	OpenSea
cg10644575	chr6	87725675	HTR1E	OpenSea
cg26253500	chr7	136641740	CHRM2	
cg01004457	chr19	15851571	OR10H3	OpenSea
cg05596267	chr15	34331198	CHRM5	
cg18200810	chr13	47472200	HTR2A	
cg07839533	chr5	63257885	HTR1A	Island
cg19630629	chr7	136556193	CHRM2	
cg04291946	chr20	60791310	HRH3	Island
cg22442841	chr6	87646804	HTR1E	N_Shore
cg15835825	chr7	154862030	HTR5A	Island
cg05942508	chr11	113846922	HTR3A	
cg27527345	chr1	159505015	OR10J5	OpenSea
cg21960184	chr11	113804386	HTR3B	OpenSea
cg00365524	chr1	19992771	HTR6	Island
cg05506446	chr11	46409501	CHRM4	N_Shore
cg18412730	chr22	23435458	RTDR1	
cg24101459	chr19	15919798	OR10H1	S_Shore
cg10323433	chr13	47471562	HTR2A	
cg14059288	chr13	47468240	HTR2A	
cg27068143	chr13	47471264	HTR2A	
cg16921789	chr3	88031773	HTR1F	
cg26333242	chr1	240072456	CHRM3	OpenSea
cg27530352	chr3	11294188	HRH1	
cg01953456	chr3	183817976	HTR3E	
cg20991421	chr6	87646740	HTR1E	N_Shore
cg18708329	chr20	60795362	HRH3	Island
cg13530039	chr11	62689557	CHRM1	N_Shore
cg10685228	chr3	183750284	HTR3D	
cg24714094	chr22	23467005	GNAZ	
cg21200229	chr7	136588030	CHRM2	
cg07116919	chr7	136558341	CHRM2	
cg04042861	chr2	231989824	HTR2B	
cg18236734	chr3	183817931	HTR3E	OpenSea
cg12974545	chr3	11198695	HRH1	
cg22614355	chr1	19991237	HTR6	Island
cg05551003	chr3	11267072	HRH1	

cg14944166	chr7	136686832	CHRM2	
cg27022535	chr20	60794588	HRH3	Island
cg22368476	chr11	123814163	OR6T1	OpenSea
cg13666507	chr5	63257941	HTR1A	S_Shore
cg08726248	chr11	637032	DRD4	Island
cg19045531	chr19	15919022	OR10H1	S_Shore
cg06804815	chr22	23438116	RTDR1	
cg17405853	chr5	175084085	HRH2	N_Shore
cg26864526	chr3	11178064	HRH1	OpenSea
cg20967585	chr7	154862524	HTR5A	N_Shore
cg15668767	chr11	46407019	CHRM4	Island
cg11131902	chr5	175084710	HRH2	N_Shore
cg15861585	chr11	637038	DRD4	Island
cg22812013	chr5	147830713	HTR4	
cg22471517	chr7	136553682	CHRM2	
cg17850597	chr19	15917833	OR10H1	
cg10418044	chr7	136553170	CHRM2	
cg07915206	chr15	34260555	AVEN	
cg04493143	chr5	147862421	HTR4	
cg00147248	chr7	136568546	CHRM2	
cg00987015	chr11	62688751	CHRM1	
cg21330960	chr22	23415915	RTDR1	
cg02440199	chr7	136691229	CHRM2	
cg12068949	chr1	159506033	OR10J5	OpenSea
cg17645664	chr3	183750429	HTR3D	
cg18243460	chr1	20005511	HTR6	Island
cg27075786	chr22	23438059	RTDR1	
cg14345676	chr5	175109098	HRH2	
cg12580770	chr7	154861569	HTR5A	N_Shore
cg16738940	chr10	92575878	HTR7	
cg20887241	chr1	23522636	HTR1D	OpenSea
cg12418071	chr19	15919836	OR10H1	S_Shore
cg11335335	chr11	637885	DRD4	Island
cg12528649	chr11	46407116	CHRM4	Island
cg00078348	chr11	113845487	HTR3A	
cg24397241	chr3	11227410	HRH1	
cg20178075	chr11	113860607	HTR3A	
cg02052721	chr1	23518539	HTR1D	
cg03657040	chr5	175083981	HRH2	N_Shore
cg00310588	chr3	183770589	HTR3C	OpenSea
cg11990309	chr6	87647644	HTR1E	Island
cg15207662	chr5	175108315	HRH2	
cg05122082	chr14	20710905	OR11H4	OpenSea
cg12598837	chr11	113845788	HTR3A	
cg04752263	chr20	60791717	HRH3	Island
cg18371750	chr5	175112799	HRH2	OpenSea
cg14483391	chr3	183749227	HTR3D	
cg17647537	chr11	113778957	HTR3B	OpenSea

cg06160669	chr1	240070975	CHRM3	OpenSea
cg05506829	chr13	47472349	HTR2A	
cg06477056	chr5	175110609	HRH2	
cg26920451	chr15	34260956	CHRM5	
cg16873130	chr7	136586950	CHRM2	
cg02250787	chr13	47470989	HTR2A	
cg16029939	chr11	640328	DRD4	Island
cg00967901	chr10	92617915	HTR7	
cg01331196	chr3	11287078	HRH1	
cg24137472	chr14	20710881	OR11H4	OpenSea
cg02579332	chr7	154875967	HTR5A	OpenSea
cg25599573	chr5	175108429	HRH2	
cg17200850	chr5	175107097	HRH2	
cg12296860	chr11	46409428	CHRM4	N_Shore
cg06718003	chr19	15851771	OR10H3	OpenSea
cg22347705	chr22	23438507	RTDR1	
cg00170438	chr20	60792389	HRH3	N_Shore
cg07075299	chr13	47472360	HTR2A	
cg15108640	chr1	240071966	CHRM3	OpenSea
cg26135506	chr10	92617562	HTR7	
cg01406506	chr5	147938667	HTR4	
cg17578539	chr5	175104957	HRH2	OpenSea
cg13069918	chr1	20005744	HTR6	Island
cg09362722	chr7	136626256	CHRM2	
cg01586609	chr11	113846937	HTR3A	
cg00378234	chr19	15904416	OR10H5	OpenSea
cg03056854	chr1	20005414	HTR6	N_Shore
cg10772974	chr15	34332433	AVEN	
cg10605520	chr20	60796141	HRH3	S_Shore
cg27051089	chr6	87653726	HTR1E	OpenSea
cg25150440	chr7	136553088	CHRM2	
cg03321592	chr1	19991676	HTR6	Island
cg12749468	chr3	183755507	HTR3D	
cg18190847	chr3	11195751	HRH1	
cg23300659	chr7	136553822	CHRM2	
cg00363114	chr11	113844663	HTR3A	
cg22863118	chr7	136701166	CHRM2	
cg01920563	chr7	136648231	CHRM2	
cg27090784	chr5	147862681	HTR4	
cg15888097	chr6	87646462	HTR1E	N_Shore
cg19116351	chr3	88031048	HTR1F	OpenSea
cg17637877	chr11	113779788	HTR3B	OpenSea
cg00973677	chr7	136553595	CHRM2	
cg17723143	chr5	148033473	HTR4	
cg05888433	chr14	20711344	OR11H4	OpenSea
cg08258494	chr1	239987344	CHRM3	OpenSea
cg11553153	chr15	34348521	CHRM5	OpenSea
cg01791421	chr1	19996240	HTR6	S_Shelf

cg00307530	chr5	147834517	HTR4	
cg17571559	chr3	11267525	HRH1	
cg24875857	chr3	183817230	HTR3E	OpenSea
cg17564844	chr22	23413784	RTDR1	
cg01468656	chr1	19991678	HTR6	Island
cg05680531	chr7	136553327	CHRM2	
cg17405012	chr7	136553263	CHRM2	
cg06457736	chr3	11178683	HRH1	OpenSea
cg07648740	chr7	154864630	HTR5A	S_Shore
cg24682621	chr1	239918125	CHRM3	OpenSea
cg03360907	chr11	62688748	CHRM1	
cg03737442	chr3	11177295	HRH1	OpenSea
cg05762326	chr10	92591168	HTR7	
cg13690703	chr3	88030644	HTR1F	OpenSea
cg00308665	chr13	47469654	HTR2A	
cg12452364	chr20	60795156	HRH3	
cg20678835	chr1	23521332	HTR1D	OpenSea
cg26332534	chr10	92618063	HTR7	
cg12513379	chr19	15838397	OR10H2	N_Shore
cg08372315	chr11	113844382	HTR3A	
cg16543009	chr15	34331514	AVEN	
cg23881368	chr13	47472343	HTR2A	
cg00576550	chr3	183749212	HTR3D	
cg17483297	chr5	175084743	HRH2	N_Shore
cg00783712	chr1	239974513	CHRM3	OpenSea
cg26153642	chr3	183818368	HTR3E	OpenSea
cg12639324	chr10	92617735	HTR7	
cg19699807	chr19	16060211	OR10H4	OpenSea
cg01625621	chr15	34260433	AVEN	
cg27230009	chr3	11241399	HRH1	
cg24661173	chr7	154866132	HTR5A	S_Shelf
cg01616732	chr20	60795457	HRH3	Island
cg25271892	chr11	62690462	CHRM1	N_Shore
cg09863950	chr1	19990768	HTR6	N_Shore
cg10650018	chr10	92616759	HTR7	
cg02527199	chr5	175085245	HRH2	Island
cg11773243	chr7	136613718	CHRM2	
cg18853490	chr1	239882840	CHRM3	OpenSea
cg17537380	chr6	87649596	HTR1E	S_Shore
cg11514288	chr13	47471197	HTR2A	
cg02517524	chr22	23412814	RTDR1	
cg08622198	chr1	239979505	CHRM3	OpenSea
cg23343875	chr19	16058370	OR10H4	OpenSea
cg10842339	chr1	240071807	CHRM3	OpenSea
cg16188532	chr13	47471090	HTR2A	
cg11666515	chr19	15919834	OR10H1	S_Shore
cg09666573	chr3	11267627	HRH1	
cg21232620	chr6	78172192	HTR1B	N_Shore

cg14841965	chr11	123814849	OR6T1	OpenSea
cg18859248	chr20	60791502	HRH3	Island
cg15625631	chr11	123814972	OR6T1	OpenSea
cg23424273	chr6	78173227	HTR1B	Island
cg25276126	chr1	20005715	HTR6	Island
cg21536328	chr1	23522665	HTR1D	OpenSea
cg01614101	chr11	113778771	HTR3B	OpenSea
cg09133032	chr11	640094	DRD4	Island
cg01274715	chr10	92618033	HTR7	
cg26724798	chr11	113844828	HTR3A	
cg00456868	chr15	34331390	AVEN	
cg09575258	chr5	175084718	HRH2	N_Shore
cg22806527	chr5	175087093	HRH2	S_Shore
cg22075328	chr22	23412381	RTDR1	
cg25843439	chr19	15852574	OR10H3	OpenSea
cg02762115	chr11	640446	DRD4	Island
cg06096336	chr2	231989800	PSMD1	
cg01808284	chr5	148031958	HTR4	
cg18271969	chr3	183771499	HTR3C	OpenSea
cg03448301	chr11	62678778	CHRM1	OpenSea
cg23720528	chr10	92501798	HTR7	
cg04607131	chr1	19990783	HTR6	N_Shore
cg13245440	chr1	19990624	HTR6	N_Shore
cg09361691	chr13	47471169	HTR2A	
cg16126186	chr18	22053986	HRH4	
cg15484742	chr1	23519884	HTR1D	OpenSea
cg08417719	chr11	123814620	OR6T1	OpenSea
cg07579109	chr22	23437461	RTDR1	
cg00023024	chr1	23521272	HTR1D	OpenSea
cg10876621	chr10	92565207	HTR7	
cg16057587	chr7	136698566	CHRM2	
cg15015426	chr1	159506559	OR10J5	OpenSea
cg10953410	chr11	113846918	HTR3A	
cg02027079	chr13	47471705	HTR2A	
cg10276834	chr7	136575360	CHRM2	
cg14986832	chr22	23412408	RTDR1	
cg11247289	chr1	19991707	HTR6	Island
cg01296705	chr5	175108269	HRH2	
cg27143370	chr20	60795923	HRH3	Island
cg01812577	chr11	62689122	CHRM1	N_Shore
cg17660833	chr3	11267020	HRH1	
cg06476131	chr13	47471052	HTR2A	
cg11788586	chr7	136606294	CHRM2	
cg27551227	chr7	154877217	HTR5A	OpenSea
cg08872493	chr1	23521417	HTR1D	OpenSea
cg12243453	chr11	113775400	HTR3B	OpenSea
cg12775613	chr3	88040034	HTR1F	OpenSea
