# Supplementary material for: Differential DNA Methylation of the Serotonin Receptor Signaling and Glutamatergic Synapse Pathways in Adult Twins Born Preterm
Source: Genes (Basel). 2026 Jun 10;17(6):683. doi: 10.3390/genes17060683 (PMC13299586; doi:10.3390/genes17060683)
Supplement: Supplementary file 1 [file genes-17-00683-s001.zip › Supplementary Table S3_glutamate_young.pdf]

CpG	logFC	t	P.Value	adj.P.Val	chr	pos
cg10529401	0.121101	4.630591	5.54E-06	0.010542	chr1	53587046
cg22291265	0.267729	4.536261	8.44E-06	0.01181	chr19	51182808
cg05112254	0.13171	4.454497	1.21E-05	0.013098	chr12	2224410
cg08098950	-0.2156	-4.29392	2.41E-05	0.01536	chr16	4033226
cg00582971	-0.45391	-4.16135	4.19E-05	0.01769	chr5	178422128
cg06247406	-0.16216	-4.11997	4.97E-05	0.018755	chr6	101846791
cg26351229	-0.15809	-4.1178	5.01E-05	0.018755	chr8	132053780
cg00439658	-0.20034	-4.07664	5.93E-05	0.019433	chr17	72848669
cg11439695	0.113051	4.076241	5.94E-05	0.019433	chr12	2561024
cg08475096	-0.17573	-4.04816	6.65E-05	0.019923	chr4	158143750
cg07456682	0.11542	4.012583	7.68E-05	0.021102	chr5	178414540
cg22762091	-0.16117	-3.99168	8.34E-05	0.02149	chr8	132052843
cg01281157	-0.199	-3.96105	9.43E-05	0.022246	chr5	178422260
cg04138502	-0.22508	-3.92225	0.00011	0.023432	chr3	123167522
cg07628416	-0.27296	-3.84613	0.000148	0.025304	chr16	4033297
cg25909396	0.13849	3.830611	0.000157	0.025824	chr17	64300729
cg11308643	-0.1473	-3.82126	0.000163	0.026163	chr11	105480788
cg01637841	0.110674	3.820814	0.000163	0.026165	chr3	7610381
cg12124094	-0.15593	-3.81894	0.000164	0.026263	chr17	64433744
cg01577933	-0.26241	-3.81285	0.000168	0.026478	chr1	37498025
cg24301620	-0.15615	-3.79582	0.00018	0.026832	chr6	101846872
cg09108394	0.135833	3.777008	0.000193	0.027284	chr16	23850106
cg10591607	-0.16758	-3.76288	0.000204	0.027822	chr6	101846916
cg25649039	0.187746	3.728558	0.000232	0.029178	chr17	64335475
cg13515021	-0.16302	-3.72694	0.000234	0.029276	chr12	49177075
cg01942127	-0.15941	-3.72335	0.000237	0.029355	chr3	53529259
cg15021670	0.118592	3.715068	0.000244	0.02968	chr15	42386726
cg07365960	-0.27136	-3.71337	0.000246	0.029736	chr17	72848535
cg13483026	-0.18216	-3.6923	0.000266	0.030678	chr3	123167973
cg15425280	-0.12029	-3.69063	0.000268	0.030678	chr4	158141492
cg24213507	-0.20201	-3.67532	0.000284	0.031354	chr3	6902689
cg17605476	-0.15793	-3.62793	0.000338	0.033074	chr4	158143917
cg14010550	-0.42866	-3.61685	0.000352	0.033679	chr19	1009642
cg14287235	-0.21515	-3.606	0.000367	0.034087	chr14	24804339
cg11844965	0.121986	3.594341	0.000383	0.034662	chr19	42510093
cg14082127	-0.15618	-3.58658	0.000394	0.035073	chr6	101847349
cg15519474	0.138411	3.583837	0.000398	0.035202	chr17	64300902
cg14859460	-0.14892	-3.57072	0.000417	0.035879	chr5	178422244
cg03451029	-0.2236	-3.55942	0.000435	0.036494	chr7	79764387
cg00082310	-0.16324	-3.5382	0.00047	0.037488	chr3	171528573
cg01250212	0.142403	3.524419	0.000494	0.038098	chr19	2650755
cg04837533	-0.17792	-3.52415	0.000494	0.038108	chr12	26986864
cg21187352	-0.14234	-3.51479	0.000511	0.038587	chr3	6903327
cg26332560	-0.18955	-3.488	0.000563	0.039875	chr8	132052887
cg15335139	-0.1802	-3.47254	0.000595	0.040492	chr3	50242325
cg02332525	-0.13445	-3.47194	0.000597	0.040529	chr3	6903153
cg16812352	0.101127	3.433408	0.000684	0.042517	chr16	4049855

cg08292023	-0.13031	-3.40893	0.000746	0.044036	chr11	88242253
cg07100700	0.100275	3.405711	0.000754	0.044293	chr6	34059787
cg17627654	-0.21142	-3.39261	0.00079	0.044946	chr11	70508410
cg08379637	-0.10318	-3.37918	0.000828	0.045673	chr21	31311387
cg01622416	0.168989	3.377123	0.000834	0.045802	chr21	31310508
cg27182923	0.078217	3.376632	0.000835	0.045823	chr3	123129387
cg04711050	-0.14221	-3.3661	0.000867	0.046382	chr9	4490757
cg15066837	0.151288	3.363581	0.000874	0.046513	chr7	79775261
cg20172500	-0.13903	-3.35913	0.000888	0.046727	chr3	6902432
cg22490420	0.098318	3.354307	0.000903	0.046974	chr15	42434040
cg24342051	-0.13254	-3.34997	0.000917	0.047259	chr3	53527884
cg11640185	0.089553	3.342743	0.00094	0.047647	chr19	42509801
cg12934382	-0.28286	-3.33137	0.000978	0.048427	chr3	51741135
cg27428129	0.100324	3.32296	0.001007	0.049054	chr6	34051095
cg26430597	0.079699	3.322383	0.001009	0.049086	chr1	182354699
cg05115424	-0.11313	-3.31945	0.001019	0.049299	chr17	64787379
cg19646112	-0.14281	-3.31071	0.00105	0.049815	chr14	24804342
cg19657814	-0.19616	-3.29718	0.0011	0.050615	chr19	47137444
cg20326410	0.093312	3.295991	0.001104	0.050716	chr1	53600821
cg02140020	0.101902	3.294245	0.001111	0.050751	chr12	2613914
cg03663215	-0.17593	-3.28926	0.00113	0.051164	chr5	7396491
cg25856632	0.105803	3.278807	0.001171	0.05173	chr17	7111551
cg09147777	-0.12704	-3.26086	0.001245	0.052848	chr11	105480771
cg19237476	0.082308	3.254592	0.001272	0.053236	chr11	70501603
cg23475371	-0.1248	-3.24593	0.00131	0.053877	chr21	31311842
cg02110273	0.089667	3.241969	0.001328	0.054127	chr17	64727684
cg01245966	-0.11733	-3.23608	0.001354	0.054543	chr8	132053823
cg27199820	-0.27378	-3.22545	0.001404	0.055208	chr3	6903019
cg22541254	-0.13423	-3.22492	0.001406	0.055259	chr6	101846779
cg16415870	-0.14529	-3.21993	0.00143	0.055562	chr3	6904261
cg22597733	-0.15711	-3.2166	0.001446	0.055863	chr4	158142891
cg02928699	-0.14154	-3.2161	0.001449	0.055893	chr11	88241946
cg01737026	-0.12244	-3.19812	0.001539	0.057218	chr6	101847656
cg14214834	0.08318	3.179831	0.001635	0.058765	chr3	123139305
cg13757263	0.117261	3.175877	0.001657	0.059097	chr3	53807593
cg02504690	0.087304	3.175251	0.001661	0.059141	chr11	70319385
cg03499570	0.089856	3.174588	0.001664	0.059181	chr11	70424529
cg06099971	0.067454	3.160058	0.001747	0.060232	chr15	42386816
cg24863175	0.180907	3.154971	0.001776	0.060695	chr1	186840433
cg15228639	0.087107	3.154915	0.001777	0.0607	chr15	42289939
cg22231400	-0.18636	-3.14889	0.001812	0.061277	chr19	49935823
cg01352090	-0.10041	-3.13361	0.001906	0.062724	chr16	4103533
cg08550394	-0.10731	-3.12114	0.001986	0.063625	chr16	4013659
cg21816539	-0.13915	-3.11506	0.002026	0.064228	chr21	31312328
cg17450585	0.089398	3.110777	0.002054	0.064601	chr6	34059718
cg09434500	-0.28135	-3.09781	0.002143	0.065693	chr19	42502897
cg13861180	0.10784	3.09302	0.002177	0.066181	chr9	140060268
cg20640499	-0.12425	-3.08724	0.002219	0.066711	chr6	101850881

cg03120091	-0.16775	-3.08465	0.002237	0.066875	chr8	132052779
cg23676682	-0.11931	-3.08134	0.002262	0.067158	chr11	105480792
cg07950000	-0.11354	-3.07546	0.002305	0.06772	chr21	31312333
cg02194717	0.118612	3.063046	0.0024	0.068647	chr11	70415188
cg25942860	0.087481	3.061264	0.002414	0.06874	chr5	178410055
cg03149432	-0.12461	-3.04349	0.002556	0.070126	chr1	37498721
cg02155655	0.06069	3.041316	0.002574	0.070352	chr1	53566481
cg15993383	-0.22762	-3.03725	0.002608	0.070745	chr3	123167507
cg01596520	0.088433	3.036847	0.002611	0.070771	chr19	14225029
cg13373757	0.091975	3.036658	0.002613	0.070774	chr12	2263558
cg23894980	0.079332	3.036092	0.002617	0.070818	chr11	70534718
cg20787196	-0.12875	-3.02929	0.002675	0.071384	chr12	26987031
cg18193094	-0.14961	-3.02883	0.002679	0.071417	chr6	101846905
cg03764381	-0.1395	-3.01603	0.002791	0.072414	chr16	10275410
cg04907257	-0.13956	-3.00924	0.002852	0.0731	chr5	7395318
cg03966406	-0.09951	-3.00371	0.002903	0.073509	chr12	26985412
cg11836949	0.252975	3.000444	0.002933	0.073809	chr1	1812359
cg19809667	-0.12415	-2.99882	0.002949	0.073982	chr19	54401945
cg14462402	0.14553	2.996605	0.002969	0.074277	chr11	120678447
cg18760587	-0.11484	-2.97682	0.003162	0.076087	chr7	79764888
cg17199007	-0.10815	-2.97016	0.003229	0.076685	chr6	146349527
cg01722994	-0.09214	-2.96888	0.003242	0.076813	chr16	10277317
cg19914607	-0.12375	-2.96881	0.003243	0.076818	chr3	50242505
cg23970331	0.094128	2.967442	0.003257	0.076965	chr6	33656237
cg13286510	-0.12256	-2.96281	0.003305	0.077434	chr7	126893007
cg07060551	-0.18127	-2.95684	0.003367	0.078057	chr19	51198381
cg02515133	0.120055	2.955848	0.003378	0.078145	chr7	86415687
cg22851944	-0.11307	-2.94619	0.003482	0.079164	chr6	101847388
cg04695635	0.082321	2.936181	0.003593	0.080295	chr19	42510823
cg16761581	-0.14465	-2.92039	0.003774	0.082061	chr14	24803807
cg21806090	0.206617	2.919611	0.003783	0.082122	chr12	2198034
cg03923850	0.092641	2.915097	0.003837	0.082625	chr12	2372169
cg19606462	0.148746	2.911899	0.003875	0.083001	chr5	7827964
cg26316946	-0.14437	-2.9089	0.003912	0.083294	chr6	101846967
cg14872036	0.106736	2.907014	0.003935	0.083467	chr3	123049031
cg15562780	-0.10751	-2.90612	0.003945	0.083598	chr11	35441311
cg05346491	0.077862	2.900911	0.00401	0.084182	chr19	48917104
cg05031016	-0.12357	-2.89856	0.004039	0.084409	chr14	24804153
cg07312654	-0.11503	-2.89395	0.004097	0.084769	chr8	132053773
cg19008133	0.086037	2.883955	0.004226	0.085823	chr3	123124015
cg24634422	-0.11535	-2.88348	0.004232	0.085862	chr11	35441593
cg19507068	-0.37444	-2.88136	0.004259	0.086018	chr7	79764176
cg20626645	-0.1787	-2.8729	0.004372	0.086869	chr3	53528846
cg15705536	0.147027	2.8716	0.004389	0.087025	chr5	7825292
cg06722633	-0.1246	-2.86958	0.004417	0.087281	chr1	37499309
cg09300795	0.146706	2.869136	0.004423	0.08733	chr16	4042428
cg13537240	0.078932	2.86418	0.004491	0.087904	chr12	2761549
cg09096555	-0.16042	-2.86347	0.0045	0.088007	chr17	72848358

cg12350325	-0.25656	-2.85592	0.004606	0.088816	chr12	2800909
cg03508063	0.086669	2.853324	0.004642	0.089067	chr17	7124385
cg24755189	0.083751	2.851464	0.004669	0.089304	chr11	62475373
cg19196684	0.081733	2.85111	0.004674	0.089343	chr1	53608037
cg03437186	-0.10291	-2.84561	0.004753	0.089978	chr7	45614848
cg00076497	-0.12023	-2.84392	0.004778	0.090157	chr7	126891621
cg00834536	-0.12427	-2.84243	0.004799	0.090338	chr16	4013537
cg27369641	0.086187	2.840143	0.004833	0.090633	chr7	100274361
cg07699277	0.09403	2.835697	0.004899	0.0911	chr6	34004226
cg05942459	-0.19481	-2.83422	0.004921	0.09133	chr6	101846805
cg02837591	-0.12362	-2.83363	0.00493	0.091367	chr6	101850261
cg10793758	0.08632	2.831416	0.004963	0.091573	chr22	51133417
cg26536401	0.074859	2.831132	0.004967	0.091615	chr12	6956432
cg06829391	0.091708	2.824927	0.005062	0.092254	chr16	9857151
cg27092975	0.070545	2.822246	0.005103	0.092528	chr11	70805455
cg24849633	0.055341	2.817697	0.005174	0.093075	chr22	51142900
cg00664406	-0.15556	-2.79656	0.005515	0.095516	chr3	51740875
cg08958294	-0.17128	-2.79522	0.005537	0.095692	chr6	146350131
cg03503785	0.102468	2.794556	0.005549	0.09575	chr16	23962572
cg17567700	-0.11113	-2.78917	0.005639	0.096421	chr22	51112218
cg19707326	0.079334	2.787302	0.005671	0.096569	chr14	24787611
cg02309655	0.090585	2.786858	0.005678	0.096631	chr19	2588629
cg23566401	-0.11552	-2.78283	0.005747	0.09712	chr17	7120484
cg09432792	0.254132	2.782226	0.005758	0.097151	chr16	56352311
cg12228229	0.083123	2.780542	0.005787	0.097384	chr17	7122261
cg03991309	0.107026	2.776871	0.005851	0.097801	chr1	68237761
cg03562531	0.073963	2.776692	0.005854	0.097812	chr3	53764604
cg26393354	0.093083	2.7738	0.005905	0.098099	chr11	70713937
cg23942984	0.157879	2.76491	0.006064	0.099163	chr12	14103087
cg04583232	-0.08143	-2.76448	0.006072	0.099188	chr11	22362874
cg04149773	0.066702	2.760701	0.006141	0.099719	chr12	49179923
cg23519329	0.101933	2.76039	0.006146	0.099761	chr16	4166914
cg22989419	0.12383	2.758598	0.006179	0.100012	chr20	9340396
cg06466348	0.078326	2.755492	0.006237	0.100477	chr16	50337922
cg23797439	-0.11359	-2.75226	0.006297	0.100795	chr20	8113355
cg26564874	0.090432	2.751825	0.006305	0.100829	chr5	178416134
cg23321702	0.112198	2.750578	0.006328	0.100971	chr6	34031060
cg02203881	0.074387	2.749387	0.006351	0.101117	chr15	42386909
cg00553487	0.105327	2.74748	0.006387	0.101327	chr19	42570406
cg06193383	-0.11039	-2.74394	0.006455	0.101823	chr16	10275767
cg25727569	0.068199	2.739328	0.006543	0.102357	chr3	53845287
cg09461286	-0.09975	-2.73868	0.006556	0.102427	chr16	10276081
cg11479156	0.179395	2.721498	0.006897	0.104362	chr11	70672388
cg05488168	0.104317	2.721495	0.006897	0.104362	chr19	13400637
cg06328100	0.090766	2.710664	0.007121	0.105933	chr6	33638806
cg14111697	-0.06663	-2.70392	0.007263	0.106747	chr9	80462928
cg13536060	-0.11876	-2.70159	0.007313	0.107078	chr19	51189671
cg00808175	0.076859	2.692339	0.007514	0.10836	chr12	6949119

cg25702790	-0.11248	-2.69042	0.007556	0.108609	chr7	79765394
cg23159970	-0.85932	-2.68844	0.0076	0.108946	chr12	2690385
cg00518386	-0.13787	-2.68654	0.007642	0.109224	chr16	10276984
cg22891619	-0.18009	-2.68162	0.007752	0.109904	chr17	72839038
cg03132806	0.102136	2.673731	0.007933	0.111104	chr5	178414179
cg27555529	-0.14353	-2.67329	0.007943	0.111149	chr19	13617518
cg03091752	-0.13565	-2.67021	0.008014	0.111593	chr19	51221605
cg10806318	0.113733	2.669358	0.008034	0.11176	chr11	70374297
cg26746936	-0.30601	-2.6689	0.008045	0.111872	chr19	42503392
cg07232612	-0.08323	-2.66658	0.008099	0.112162	chr7	93551012
cg25852492	0.093719	2.665749	0.008119	0.11228	chr15	42140150
cg10286380	-0.10531	-2.66245	0.008197	0.112713	chr19	51171847
cg13510813	0.079931	2.661873	0.00821	0.112798	chr19	42571339
cg15603568	-0.12345	-2.66166	0.008215	0.112818	chr11	105481283
cg25001544	0.193784	2.657064	0.008325	0.113407	chr6	34073788
cg14263118	0.067668	2.655513	0.008363	0.113587	chr20	57463787
cg11544138	0.07551	2.652726	0.008431	0.114065	chr19	1003455
cg15651980	0.094481	2.651631	0.008457	0.114205	chr19	48903304
cg13555101	-0.10386	-2.65106	0.008471	0.114317	chr9	4490751
cg27178677	0.109744	2.651016	0.008472	0.114326	chr20	8834803
cg18799510	-0.12193	-2.6447	0.008628	0.115122	chr9	104499700
cg27073262	0.160157	2.644507	0.008633	0.115158	chr7	86493792
cg08997253	-0.13659	-2.63987	0.008749	0.115866	chr9	104500729
cg04527363	-0.10762	-2.63482	0.008877	0.11666	chr3	6902337
cg04336164	0.111366	2.634064	0.008896	0.116815	chr2	68478630
cg04396791	-0.28223	-2.63033	0.008992	0.117365	chr11	70508180
cg09305491	0.098061	2.628614	0.009037	0.117587	chr16	24151191
cg10409919	-0.15118	-2.62813	0.009049	0.117643	chr3	53530016
cg14093720	0.074644	2.62784	0.009057	0.117693	chr18	3712400
cg14019146	0.126797	2.625577	0.009116	0.118025	chr3	50243930
cg13384396	-0.10048	-2.62334	0.009175	0.118252	chr3	123167677
cg27333271	0.164739	2.622212	0.009204	0.118466	chr3	7268498
cg16086007	-0.11221	-2.62124	0.00923	0.118655	chr17	72855588
cg08955995	-0.1953	-2.61859	0.0093	0.119066	chr19	42503412
cg08310216	-0.08874	-2.61856	0.009301	0.119067	chr7	100271217
cg01663725	-0.10914	-2.61811	0.009313	0.119121	chr12	14133829
cg10904109	-0.15739	-2.61505	0.009395	0.119664	chr6	146755494
cg21217024	-0.11982	-2.61442	0.009412	0.119718	chr11	105481406
cg20132775	0.144509	2.614301	0.009415	0.119743	chr3	142444202
cg20459126	-0.09523	-2.61191	0.009479	0.120142	chr3	142443247
cg18842187	0.0535	2.604232	0.009689	0.121316	chr6	33647826
cg02807849	0.075195	2.603073	0.009721	0.12157	chr19	48908102
cg20882260	0.075774	2.602075	0.009749	0.121767	chr12	2374427
cg25744767	-0.28272	-2.60044	0.009794	0.121958	chr7	79764178
cg25148589	-0.12968	-2.59941	0.009823	0.122182	chr4	158141936
cg03403991	0.074023	2.594504	0.009961	0.122826	chr22	51167187
cg16359985	0.078786	2.59107	0.010059	0.123326	chr7	100276087
cg09106984	0.081369	2.586282	0.010196	0.123952	chr6	34004360

cg22491927	-0.21201	-2.58202	0.01032	0.124618	chr19	13617091
cg13878010	-0.09677	-2.57949	0.010394	0.124994	chr3	123167276
cg15828915	0.150775	2.573817	0.010562	0.125977	chr12	26801163
cg21563683	-0.08684	-2.57272	0.010595	0.126136	chr12	46767928
cg26780231	0.204817	2.572689	0.010596	0.126136	chr17	64468338
cg00426968	-0.10124	-2.57121	0.01064	0.126396	chr19	47138284
cg14926715	0.0655	2.571139	0.010642	0.126417	chr11	70318919
cg14753385	0.073308	2.568116	0.010733	0.126869	chr11	70476422
cg09354294	0.066621	2.551745	0.011239	0.12971	chr1	68188118
cg19032532	0.111097	2.547825	0.011363	0.130281	chr19	2547067
cg17483510	-0.12363	-2.54659	0.011402	0.130486	chr3	179168677
cg19063061	-0.20083	-2.54559	0.011434	0.130663	chr19	49935893
cg14483383	0.083269	2.544268	0.011477	0.130881	chr11	64022763
cg19965023	0.073696	2.541526	0.011565	0.131379	chr17	72838366
cg04453050	-0.1039	-2.53848	0.011664	0.131821	chr3	51740896
cg02551234	0.091504	2.536462	0.01173	0.132129	chr11	64023126
cg24275501	0.0777	2.534925	0.01178	0.132432	chr12	2198070
cg18391758	-0.09071	-2.53365	0.011822	0.132644	chr16	10274963
cg24611631	-0.10028	-2.52824	0.012002	0.133546	chr9	4490288
cg20018057	0.064296	2.52812	0.012006	0.133564	chr20	57465139
cg25407736	0.15169	2.526741	0.012052	0.133751	chr1	68296179
cg06421614	-0.08731	-2.5236	0.012158	0.134364	chr17	7121116
cg14530764	0.065234	2.522315	0.012201	0.134578	chr3	123124018
cg12377578	-0.07684	-2.52148	0.01223	0.134722	chr17	72856181
cg18356448	0.15343	2.52065	0.012258	0.134829	chr18	3881547
cg13203394	-0.07602	-2.5201	0.012277	0.134883	chr12	26951217
cg03856723	-0.08553	-2.51866	0.012326	0.135093	chr19	14229466
cg20871277	0.07967	2.51631	0.012407	0.135515	chr6	33656548
cg10262891	0.073639	2.514323	0.012475	0.135788	chr19	48904928
cg15805568	-0.10522	-2.51305	0.012519	0.135994	chr19	51199000
cg07309764	-0.12862	-2.51046	0.012609	0.136345	chr7	79763914
cg14724613	-0.08645	-2.50905	0.012659	0.136574	chr7	86273429
cg02303571	-0.09237	-2.50853	0.012677	0.136671	chr5	36606769
cg12682032	0.068322	2.504383	0.012823	0.137305	chr15	83617937
cg04537738	0.070373	2.503738	0.012846	0.137405	chr22	51143999
cg01207684	0.182448	2.501083	0.012941	0.137894	chr16	4103167
cg03193168	0.079599	2.498253	0.013042	0.138347	chr22	51159995
cg01780685	0.067741	2.496675	0.013099	0.138625	chr17	7099875
cg24607686	0.125131	2.49491	0.013163	0.138979	chr2	191827930
cg06954761	-0.10917	-2.48563	0.013504	0.140596	chr6	33601863
cg04023483	-0.10646	-2.48473	0.013537	0.140713	chr3	6904134
cg14036830	-0.43831	-2.4784	0.013774	0.141754	chr19	42503207
cg22689690	-0.11062	-2.47735	0.013814	0.141889	chr12	49183468
cg18175690	0.10524	2.46506	0.014287	0.14379	chr15	40580770
cg21734356	0.074955	2.463794	0.014336	0.143972	chr18	3498854
cg12191293	-0.08269	-2.45937	0.01451	0.144756	chr12	56882314
cg07716032	0.087975	2.458606	0.01454	0.144831	chr17	7122846
cg09408768	-0.1073	-2.45717	0.014598	0.145123	chr2	155555053

cg13570585	-0.09557	-2.45696	0.014606	0.145135	chr20	8113573
cg22198397	0.069331	2.456892	0.014608	0.145141	chr19	15067457
cg27644513	0.109594	2.453264	0.014754	0.14569	chr15	42281679
cg17540575	0.061405	2.445673	0.015061	0.14694	chr19	42504627
cg10648542	0.075098	2.445624	0.015063	0.14694	chr5	178416050
cg13448720	0.079439	2.44427	0.015119	0.147222	chr9	140052246
cg03153115	0.073468	2.443591	0.015146	0.147323	chr19	2604559
cg12709244	-0.21036	-2.44211	0.015208	0.147566	chr17	7123282
cg00699993	-0.20715	-2.44074	0.015264	0.147782	chr4	158141570
cg17401938	0.100329	2.438888	0.015341	0.148123	chr12	2228442
cg18707858	0.077006	2.434594	0.01552	0.148822	chr19	13366101
cg04510788	-0.13375	-2.43344	0.015569	0.149042	chr1	37498900
cg15995075	0.087078	2.431896	0.015634	0.149342	chr3	142451487
cg11855555	-0.10578	-2.43105	0.01567	0.14952	chr1	68232134
cg10123654	0.094899	2.429718	0.015726	0.149801	chr16	4162541
cg16341159	-0.07847	-2.42954	0.015734	0.149801	chr17	7121370
cg03070741	0.085736	2.421702	0.01607	0.151214	chr19	2650727
cg23183497	-0.08284	-2.42002	0.016143	0.151493	chr7	86273718
cg09965996	-0.08316	-2.4195	0.016165	0.151566	chr16	56390429
cg12887832	0.07881	2.415855	0.016325	0.152296	chr11	70805627
cg17754876	-0.09429	-2.41443	0.016388	0.152532	chr11	35441260
cg12778476	-0.13152	-2.41172	0.016507	0.152998	chr11	22359345
cg17871403	0.076778	2.409168	0.016621	0.153554	chr5	7827115
cg12496211	0.092831	2.408821	0.016637	0.153647	chr12	2193060
cg16696270	-0.12573	-2.40656	0.016738	0.154087	chr8	132052934
cg20790998	0.077303	2.405346	0.016792	0.154269	chr1	68290436
cg24159247	-0.07121	-2.40161	0.016961	0.154934	chr3	4575483
cg25693099	0.073109	2.400021	0.017034	0.155227	chr18	3879303
cg26555126	0.072001	2.399921	0.017038	0.155242	chr6	33998729
cg05716556	0.055342	2.396608	0.01719	0.155927	chr17	47287410
cg16378117	0.069122	2.393961	0.017312	0.156476	chr16	9857804
cg05616819	0.131462	2.393483	0.017334	0.156569	chr16	24231485
cg01975093	0.093866	2.389755	0.017507	0.157277	chr11	62474759
cg20771332	0.08135	2.386005	0.017683	0.157882	chr11	70332620
cg10614021	-0.08629	-2.38429	0.017764	0.15818	chr12	14134486
cg18872881	0.12182	2.381144	0.017914	0.15884	chr4	102199803
cg09143713	0.072717	2.380518	0.017943	0.158958	chr20	9141615
cg04569608	-0.08379	-2.37639	0.018142	0.159661	chr11	64018309
cg22920586	0.08454	2.373946	0.01826	0.160084	chr3	171472629
cg15706539	0.067718	2.371241	0.018391	0.160606	chr11	70924914
cg07254421	-0.09054	-2.37056	0.018424	0.160744	chr5	36657993
cg04590790	0.139672	2.367846	0.018557	0.161224	chr5	7770690
cg17604429	0.086884	2.367539	0.018572	0.161263	chr5	7827133
cg10509626	-0.12762	-2.36745	0.018577	0.161272	chr11	70333993
cg16848712	-0.09091	-2.36468	0.018713	0.161832	chr12	46767747
cg03489495	-0.09765	-2.36449	0.018723	0.161869	chr6	33588875
cg14679587	-0.11478	-2.362	0.018847	0.16233	chr12	56882324
cg20073686	-0.08571	-2.36061	0.018916	0.162624	chr11	105481863

cg24539500	0.11253	2.352807	0.01931	0.16413	chr6	102115051
cg13722123	-0.11499	-2.35243	0.019329	0.164181	chr6	146350346
cg23715749	0.105594	2.350772	0.019414	0.164528	chr1	37413867
cg19228334	0.171609	2.347859	0.019563	0.165065	chr6	101851283
cg24082826	-0.08677	-2.34639	0.019639	0.165342	chr12	26985738
cg17742947	0.080476	2.34211	0.019861	0.166187	chr19	42546977
cg13891121	-0.09452	-2.33841	0.020055	0.166908	chr12	26987045
cg24620508	0.101845	2.338245	0.020064	0.166909	chr21	31310605
cg00146655	0.066201	2.334797	0.020246	0.167569	chr3	7517194
cg24868359	-0.10784	-2.33479	0.020246	0.167569	chr21	31312535
cg16803737	0.068112	2.332159	0.020386	0.168129	chr6	33592658
cg20818778	-0.0929	-2.33143	0.020426	0.168248	chr1	235814145
cg03225817	-0.18131	-2.33104	0.020446	0.168288	chr11	105481317
cg10774282	0.070885	2.328778	0.020568	0.16864	chr1	53608280
cg07906046	-0.06537	-2.32762	0.02063	0.16889	chr16	4131584
cg15331781	-0.08196	-2.32559	0.02074	0.169177	chr7	86274443
cg02693486	0.067811	2.325552	0.020742	0.169177	chr11	64030862
cg06068039	0.081364	2.323881	0.020833	0.169437	chr6	34031208
cg24643102	-0.10382	-2.32284	0.02089	0.169642	chr3	6903921
cg00392377	-0.09435	-2.32131	0.020973	0.170011	chr19	49939882
cg06844159	0.080853	2.318896	0.021106	0.170416	chr11	70374355
cg27630678	0.066749	2.316705	0.021226	0.170831	chr11	70565201
cg19755318	-0.09875	-2.31042	0.021577	0.171941	chr3	50243323
cg27335600	-0.0791	-2.30925	0.021642	0.172221	chr3	53528857
cg09465746	-0.14864	-2.30859	0.021679	0.172362	chr3	6904386
cg27027803	0.063699	2.304223	0.021927	0.173091	chr20	57464742
cg05410012	-0.08812	-2.29992	0.022173	0.173823	chr17	72857095
cg17921248	-0.09889	-2.29599	0.0224	0.174661	chr17	64298993
cg12389770	-0.09599	-2.29573	0.022415	0.174689	chr6	101847706
cg17987968	0.076494	2.295272	0.022441	0.174739	chr5	152869882
cg23734973	-0.09209	-2.29126	0.022676	0.175506	chr4	158141449
cg24445167	0.072924	2.290635	0.022712	0.175655	chr12	2383231
cg23310850	-0.1113	-2.28907	0.022804	0.176039	chr19	19051337
cg27181295	0.069027	2.288974	0.02281	0.176048	chr19	2511475
cg01132471	0.078783	2.287981	0.022868	0.176216	chr1	53556482
cg25397191	0.07582	2.287497	0.022897	0.176318	chr19	14224992
cg17214089	0.076864	2.27884	0.023414	0.178038	chr1	182354912
cg11300838	0.062686	2.27883	0.023414	0.178038	chr19	19049950
cg03972076	0.066537	2.273065	0.023764	0.179163	chr11	64023183
cg25557995	0.091541	2.27204	0.023827	0.179385	chr12	2761091
cg00303541	-0.15547	-2.27045	0.023924	0.179707	chr3	51741280
cg15436476	0.074193	2.267569	0.024102	0.180376	chr19	2626283
cg20227471	0.069028	2.266837	0.024147	0.180515	chr2	25065550
cg20684528	-0.09557	-2.26594	0.024202	0.180699	chr12	14133667
cg11284196	-0.07122	-2.2642	0.024311	0.181045	chr19	51190047
cg15174564	-0.24353	-2.26143	0.024483	0.181564	chr11	120856801
cg13709913	0.064669	2.261107	0.024504	0.181604	chr9	104334427
cg10271186	0.071407	2.26003	0.024572	0.18188	chr11	70908897

cg02882755	0.079915	2.257385	0.024738	0.182464	chr6	34100963
cg14468634	0.122285	2.256545	0.024791	0.18261	chr5	78758952
cg03777288	0.068753	2.256049	0.024823	0.182712	chr12	13717033
cg17298751	-0.10716	-2.2559	0.024832	0.182725	chr11	22363370
cg25217317	-0.08811	-2.25492	0.024894	0.182918	chr1	235811994
cg01962826	0.220044	2.254446	0.024925	0.183051	chr6	34100967
cg21496785	0.083797	2.252212	0.025067	0.183462	chr5	178420865
cg14301531	0.067226	2.251281	0.025127	0.183689	chr12	2226907
cg02774856	-0.07812	-2.25023	0.025194	0.183865	chr19	19052293
cg16222802	0.078095	2.247761	0.025353	0.184463	chr3	50295474
cg12265829	-0.08617	-2.24739	0.025377	0.184527	chr14	24804022
cg06371583	0.064354	2.241755	0.025744	0.185735	chr19	2581343
cg02471325	0.11017	2.237968	0.025993	0.186467	chr15	42290555
cg01806181	0.080756	2.237172	0.026046	0.186648	chr19	1007843
cg08429705	0.645768	2.23587	0.026132	0.18696	chr19	2583601
cg05659265	-0.08703	-2.23442	0.026228	0.187257	chr16	56225846
cg19234705	0.066398	2.234091	0.02625	0.187323	chr19	19042181
cg05736079	-0.24816	-2.2318	0.026403	0.187809	chr9	140063205
cg10943398	0.085696	2.228404	0.026631	0.188459	chr11	70319250
cg09963080	0.096117	2.22413	0.02692	0.1893	chr16	4017270
cg03100801	0.122467	2.22383	0.026941	0.189367	chr20	9075962
cg26875958	0.068729	2.223031	0.026995	0.18955	chr6	146751590
cg01793368	0.062658	2.21867	0.027294	0.190441	chr11	64022905
cg09146232	0.075854	2.215613	0.027506	0.19112	chr17	64672129
cg01348055	0.065289	2.214497	0.027583	0.191392	chr16	10272788
cg22632947	-0.06692	-2.21337	0.027662	0.191604	chr17	64787784
cg17799599	-0.13348	-2.20704	0.028106	0.19305	chr17	64787605
cg25988118	0.058099	2.206064	0.028175	0.193263	chr6	34101785
cg09327847	0.067908	2.204505	0.028285	0.193577	chr16	24087793
cg04884798	0.057296	2.203408	0.028363	0.193794	chr14	24791720
cg04209460	0.085811	2.200038	0.028604	0.194428	chr17	4711018
cg09662638	0.073527	2.198887	0.028687	0.194689	chr3	53795946
cg05850280	-0.10353	-2.19783	0.028763	0.194883	chr9	140056489
cg13583454	0.133227	2.197696	0.028772	0.194923	chr5	153038077
cg10785385	0.063439	2.196163	0.028883	0.195282	chr22	51114364
cg17767099	-0.10482	-2.19384	0.029051	0.195823	chr19	1009048
cg07735790	0.082575	2.190236	0.029315	0.196643	chr6	34101545
cg21006600	0.107758	2.190086	0.029326	0.196671	chr12	2342206
cg02484455	0.066254	2.184738	0.02972	0.19775	chr11	70559534
cg23559689	-0.0989	-2.17956	0.030106	0.198798	chr11	105481292
cg09640070	0.074496	2.178351	0.030197	0.199028	chr12	26876374
cg00110790	0.071122	2.17147	0.030718	0.200664	chr6	33655814
cg24454829	-0.08565	-2.1708	0.030769	0.200783	chr11	22363053
cg05484458	0.067083	2.161208	0.031509	0.202872	chr12	6949260
cg21895324	0.060509	2.161096	0.031518	0.202887	chr11	35385435
cg17168836	0.097653	2.159878	0.031613	0.203161	chr1	68256161
cg09598225	-0.10212	-2.15771	0.031783	0.203619	chr20	57466839
cg05509359	0.108247	2.153992	0.032076	0.204499	chr11	70432884

cg22187630	-0.09606	-2.15361	0.032106	0.204568	chr19	13616871
cg21323244	0.064628	2.152389	0.032203	0.204848	chr11	70415439
cg13724160	-0.15661	-2.14949	0.032435	0.205529	chr9	104500958
cg21245981	-0.08161	-2.14924	0.032455	0.205546	chr5	36607390
cg16999602	0.06205	2.147683	0.032579	0.205909	chr1	53608163
cg19274368	0.068935	2.14719	0.032619	0.206022	chr11	70331491
cg24874003	0.072881	2.146222	0.032697	0.206263	chr19	2602614
cg01081636	0.059726	2.143949	0.03288	0.206774	chr6	33994263
cg08236022	-0.07522	-2.13257	0.033812	0.209545	chr7	93551014
cg15884992	0.059565	2.131177	0.033927	0.209865	chr6	34028192
cg12198934	0.067825	2.129903	0.034033	0.210173	chr17	64378035
cg26626089	-0.10013	-2.12674	0.034297	0.210847	chr19	54385865
cg08586541	-0.1622	-2.12641	0.034326	0.210933	chr19	51198888
cg26381514	0.095265	2.122856	0.034625	0.211721	chr12	26963489
cg09973502	-0.08724	-2.12285	0.034625	0.211721	chr12	46766012
cg26350373	0.065841	2.11991	0.034875	0.212284	chr15	42449015
cg02569086	-0.08488	-2.1198	0.034884	0.212284	chr12	26985672
cg13907146	-0.0856	-2.11918	0.034937	0.212418	chr3	50243565
cg08258650	-0.08108	-2.11822	0.035019	0.21265	chr11	35441900
cg10546626	0.067508	2.116284	0.035185	0.213181	chr20	57424521
cg21201396	0.101989	2.115208	0.035277	0.2134	chr11	70665271
cg07642043	-0.13522	-2.11415	0.035368	0.213665	chr16	10276674
cg22941646	-0.07133	-2.11327	0.035443	0.213882	chr1	235814339
cg01427575	-0.12195	-2.11306	0.035462	0.213931	chr19	51171712
cg01542384	-0.07508	-2.11211	0.035544	0.214144	chr3	50284305
cg22953407	0.055896	2.109326	0.035785	0.214782	chr5	178408081
cg04158792	0.062168	2.109084	0.035807	0.21484	chr19	2514622
cg19915762	0.055565	2.108966	0.035817	0.214854	chr11	64023086
cg16684939	-0.06923	-2.10798	0.035903	0.215061	chr7	100272223
cg20490197	0.064137	2.101125	0.036505	0.216681	chr6	34000298
cg20875807	-0.06688	-2.09571	0.036987	0.218174	chr15	83620951
cg02640558	-0.08811	-2.09489	0.03706	0.218392	chr8	22299141
cg05460776	0.066233	2.086209	0.037846	0.220416	chr16	4031231
cg15164708	-0.0773	-2.08147	0.03828	0.2216	chr19	49936274
cg03578926	-0.14264	-2.08092	0.038331	0.221711	chr11	70508032
cg09407429	-0.0588	-2.07795	0.038605	0.222358	chr3	4534383
cg10013716	-0.09061	-2.07783	0.038617	0.222376	chr3	179168760
cg11833293	0.074021	2.070055	0.039345	0.224264	chr11	70557519
cg02399044	-0.09368	-2.06891	0.039453	0.224557	chr12	2500229
cg26562691	0.068705	2.068907	0.039453	0.224557	chr16	23850404
cg20342105	0.064837	2.067558	0.039581	0.224921	chr11	62474910
cg03760316	-0.07582	-2.06541	0.039785	0.225541	chr18	3594197
cg04364463	-0.0556	-2.06522	0.039804	0.225595	chr1	37498270
cg05068686	0.078188	2.065128	0.039812	0.225623	chr11	70419186
cg13896105	0.047159	2.064735	0.039849	0.2257	chr12	2304473
cg15338449	-0.08275	-2.06178	0.040132	0.226482	chr15	83620910
cg09649610	-0.09085	-2.0612	0.040188	0.226593	chr1	235814039
cg06047881	0.059934	2.060284	0.040276	0.226783	chr20	57465132

cg12502079	0.060126	2.058522	0.040446	0.22721	chr22	51169028
cg03485674	-0.05875	-2.05839	0.040459	0.227231	chr16	50347895
cg03029664	0.064138	2.057244	0.040569	0.227526	chr17	72840306
cg24428099	0.063867	2.056925	0.0406	0.227598	chr2	25065702
cg16177440	0.060695	2.055388	0.040749	0.227979	chr7	100275304
cg13936125	-0.06995	-2.05462	0.040824	0.228199	chr16	56225599
cg00140112	0.060748	2.054587	0.040827	0.228199	chr18	3879595
cg00683984	-0.12086	-2.05133	0.041146	0.229043	chr7	45615337
cg26640467	-0.07722	-2.04879	0.041395	0.229707	chr7	126893304
cg01291761	0.086383	2.048434	0.04143	0.229796	chr12	14017080
cg14291291	0.081973	2.046398	0.041631	0.230313	chr6	33656083
cg24454695	-0.06332	-2.04416	0.041854	0.230829	chr1	235814326
cg04743945	-0.07673	-2.04401	0.041869	0.230882	chr7	86273058
cg02380914	0.06071	2.043071	0.041962	0.231146	chr22	51143114
cg20837354	0.071701	2.040779	0.042191	0.231739	chr12	2398146
cg15008401	-0.07843	-2.03985	0.042284	0.23196	chr4	102267974
cg05926269	0.063729	2.038923	0.042377	0.232189	chr20	57463906
cg05725666	0.056197	2.038394	0.04243	0.232316	chr12	2224644
cg07678592	0.061541	2.037824	0.042488	0.232487	chr12	49178406
cg00150025	-0.08689	-2.03346	0.042929	0.23346	chr15	42448079
cg16135716	-0.16869	-2.02639	0.043653	0.23519	chr12	14133887
cg10341242	-0.05801	-2.02479	0.043818	0.235516	chr16	50347849
cg08218799	0.055476	2.024624	0.043835	0.235557	chr14	24804930
cg14869721	0.065926	2.023212	0.043981	0.235917	chr2	25065924
cg13763339	-0.10328	-2.02099	0.044213	0.236412	chr11	70516627
cg02585344	-0.10186	-2.02051	0.044263	0.236546	chr16	10276092
cg21858376	-0.06691	-2.01969	0.044348	0.236671	chr3	4534791
cg26268742	0.0801	2.016533	0.044679	0.237386	chr19	48563560
cg02011392	-0.08413	-2.01194	0.045163	0.23869	chr6	101847541
cg13401531	0.077015	2.011748	0.045184	0.238721	chr11	70333281
cg24764979	-0.07838	-2.01154	0.045206	0.238774	chr16	10276600
cg07417708	-0.06662	-2.01136	0.045226	0.238815	chr5	78809348
cg17960347	0.067597	2.010314	0.045337	0.239104	chr12	2457373
cg22175856	0.079495	2.009277	0.045447	0.239334	chr19	15084302
cg12581769	0.050636	2.008027	0.045581	0.239656	chr19	13412999
cg23369234	0.068014	2.006124	0.045785	0.240108	chr12	2511478
cg14061491	0.171256	2.006108	0.045786	0.240108	chr9	80433462
cg10590857	-0.10511	-2.00242	0.046184	0.241055	chr5	7397021
cg12647801	0.072088	2.001904	0.046239	0.241184	chr11	64028732
cg10362475	-0.13582	-2.00135	0.0463	0.241345	chr11	70507825
cg20955817	0.056714	2.001334	0.046301	0.241345	chr11	70936560
cg25839745	0.075053	1.999971	0.046449	0.24172	chr4	101969341
cg02224372	0.092411	1.996826	0.046792	0.242514	chr11	70858695
cg20877313	-0.0797	-1.99627	0.046852	0.242681	chr12	56881753
cg14101380	0.069352	1.991208	0.047409	0.243868	chr11	70718575
cg07366462	0.052393	1.990229	0.047518	0.244077	chr3	123162899
cg10106561	0.060443	1.988824	0.047673	0.244451	chr2	25050913
cg05793288	0.061147	1.988136	0.04775	0.244612	chr15	42375586

cg27279652	-0.08432	-1.98665	0.047915	0.244993	chr12	26986506
cg19200285	-0.07439	-1.98647	0.047935	0.245025	chr12	2800755
cg14351692	0.073633	1.986414	0.047942	0.245042	chr12	13716374
cg00226831	0.084443	1.985235	0.048073	0.245309	chr15	42371511
cg04894216	0.122592	1.98325	0.048296	0.245876	chr7	86377879
cg14696064	0.061319	1.983015	0.048322	0.245921	chr3	123010055
cg22639787	0.050694	1.98273	0.048354	0.245979	chr20	57464973
cg07813421	-0.08275	-1.98098	0.048552	0.246374	chr17	7123626
cg18011401	-0.10707	-1.9799	0.048674	0.24671	chr19	13617366
cg02218200	0.06184	1.979438	0.048725	0.246807	chr22	51135138
cg18827756	0.053163	1.97774	0.048918	0.247299	chr15	42130735
cg17006443	0.068724	1.974547	0.049281	0.248078	chr11	70628938
cg04505435	0.147425	1.974054	0.049338	0.248241	chr11	70672511
cg06736148	0.059264	1.97397	0.049347	0.248258	chr15	52416833
cg05846851	0.070345	1.968776	0.049944	0.249527	chr16	10172054
cg14537332	-0.19158	-1.96869	0.049954	0.249531	chr11	70508113
cg23766996	0.10371	1.965911	0.050276	0.250296	chr7	86272023
cg03645007	0.060067	1.965566	0.050316	0.250371	chr3	50255295
cg15334006	0.057929	1.964105	0.050486	0.250809	chr15	42449916
cg27222147	0.060312	1.963281	0.050582	0.250972	chr12	2224755
cg14829063	0.062318	1.960207	0.050942	0.251747	chr11	70731587
cg15134033	-0.08375	-1.95861	0.05113	0.252154	chr16	10274415
cg26213368	0.055556	1.95616	0.05142	0.252794	chr11	62474978
cg13180375	0.082003	1.955565	0.05149	0.2529	chr8	22298119
cg11169463	0.062335	1.954866	0.051573	0.253102	chr6	33653411
cg16753846	0.064716	1.953026	0.051792	0.253557	chr11	70318894
cg05282459	0.054023	1.952621	0.05184	0.253669	chr22	51117157
cg06628693	-0.11485	-1.95151	0.051973	0.253936	chr1	84543156
cg24693368	-0.07457	-1.951	0.052034	0.254079	chr2	155554844
cg04270835	-0.0791	-1.94967	0.052192	0.254412	chr11	22359188
cg00350942	-0.08597	-1.94953	0.052209	0.254438	chr9	140034073
cg25960567	-0.11149	-1.94735	0.052471	0.254993	chr12	26985181
cg21899500	-0.09555	-1.94396	0.05288	0.255783	chr3	51740850
cg19471040	0.055503	1.937452	0.053675	0.257588	chr6	34031142
cg01980361	0.061613	1.937277	0.053696	0.257635	chr3	53843939
cg22518097	0.115552	1.934998	0.053976	0.258227	chr4	101948183
cg20151098	0.07325	1.934779	0.054003	0.258278	chr6	33995991
cg26160180	0.196855	1.932637	0.054268	0.25874	chr1	1822883
cg21725265	-0.07947	-1.92937	0.054674	0.259655	chr19	19051201
cg18558423	0.062034	1.928257	0.054813	0.259923	chr6	33653506
cg17721618	0.049214	1.927895	0.054858	0.26001	chr15	42376692
cg05060704	-0.0878	-1.92718	0.054948	0.260158	chr3	50275694
cg23832825	0.054809	1.926133	0.055079	0.260429	chr11	70842453
cg12089094	0.056948	1.926038	0.055091	0.26046	chr3	171463962
cg03827772	0.054138	1.925757	0.055126	0.260537	chr11	70912450
cg14397813	0.074584	1.925036	0.055217	0.260749	chr9	80522508
cg24497686	0.07552	1.922981	0.055475	0.261331	chr1	53606574
cg16168199	-0.0662	-1.92182	0.055622	0.261549	chr17	7123990

cg15014684	0.055201	1.921564	0.055654	0.26158	chr17	7093301
cg27368776	0.130723	1.92135	0.055681	0.261638	chr11	105793986
cg00341980	-0.07192	-1.91745	0.056177	0.262656	chr1	84544220
cg03654598	0.077149	1.917411	0.056182	0.262667	chr19	51202141
cg16142824	0.082997	1.917009	0.056233	0.262766	chr5	178413721
cg10425005	-0.07813	-1.9166	0.056286	0.262848	chr16	10133433
cg27371466	0.054892	1.9134	0.056695	0.263766	chr6	34003640
cg25134567	0.052753	1.912039	0.05687	0.264125	chr17	64782369
cg23068476	0.068698	1.909696	0.057173	0.264806	chr12	2613703
cg00898486	0.055844	1.907597	0.057445	0.265393	chr6	34006557
cg07190947	0.062787	1.906241	0.057621	0.265772	chr11	70349808
cg18920858	0.088929	1.905813	0.057677	0.26588	chr17	64617748
cg27484541	0.090766	1.905774	0.057682	0.265899	chr20	57461542
cg08875948	-0.07479	-1.90527	0.057747	0.266078	chr6	146755900
cg24098927	-0.07175	-1.90458	0.057838	0.266252	chr7	86273180
cg05082609	0.111946	1.904198	0.057888	0.266337	chr11	105692831
cg03412431	0.06755	1.903996	0.057914	0.266372	chr6	34023418
cg11871050	0.077786	1.903935	0.057922	0.266387	chr15	52477794
cg08461840	-0.05786	-1.90237	0.058127	0.266777	chr19	2620967
cg11444428	0.078793	1.902052	0.058169	0.266848	chr16	850802
cg12013817	0.073214	1.90189	0.05819	0.266863	chr6	34008384
cg22900607	0.132413	1.901484	0.058244	0.266955	chr19	2546938
cg08478447	-0.0882	-1.90117	0.058285	0.267043	chr3	53528849
cg16512895	0.077623	1.900389	0.058388	0.267245	chr19	13410117
cg24891846	0.226024	1.89344	0.059309	0.269129	chr19	13319523
cg06310285	0.064173	1.891598	0.059555	0.26962	chr3	53796066
cg10024799	0.062325	1.890994	0.059636	0.269785	chr12	2641381
cg26419941	-0.08842	-1.89052	0.059699	0.269908	chr12	49183133
cg07855933	0.063003	1.89013	0.059752	0.270064	chr19	13349725
cg16079774	-0.09414	-1.88927	0.059867	0.270329	chr11	22364498
cg06419562	-0.07065	-1.88888	0.05992	0.270435	chr16	24148747
cg07824914	-0.06731	-1.88847	0.059975	0.270516	chr20	57465815
cg07192048	-0.12293	-1.88684	0.060195	0.27102	chr11	70560211
cg17020834	-0.07728	-1.88503	0.06044	0.271459	chr5	152870258
cg09428623	-0.06734	-1.88486	0.060462	0.271495	chr2	68480128
cg00444360	0.086575	1.881458	0.060925	0.272495	chr11	88270612
cg08170375	0.073111	1.880373	0.061073	0.2728	chr3	50247180
cg20741386	0.056511	1.879201	0.061234	0.273148	chr1	53556189
cg26282150	-0.13617	-1.87861	0.061316	0.273322	chr1	110091625
cg21072025	-0.07049	-1.87651	0.061603	0.273923	chr19	47137863
cg17107112	0.064593	1.874685	0.061855	0.274471	chr6	34021546
cg24363298	0.06996	1.869812	0.062532	0.275849	chr3	50242671
cg12150066	-0.07034	-1.86932	0.0626	0.275998	chr1	1823305
cg07533824	0.071659	1.868965	0.06265	0.276112	chr19	13363864
cg12754421	-0.0809	-1.86851	0.062713	0.276263	chr11	105480790
cg22971402	0.07678	1.868221	0.062754	0.276343	chr6	33993822
cg00287829	-0.07319	-1.86596	0.063071	0.276973	chr16	10276375
cg22125912	0.095	1.864136	0.063327	0.2775	chr3	171428263

cg26914334	0.057195	1.863925	0.063357	0.277567	chr11	120561236
cg22454005	0.066862	1.863394	0.063432	0.277665	chr19	54392916
cg04955246	0.04007	1.861568	0.06369	0.278129	chr17	64720790
cg20801637	0.071328	1.860941	0.063779	0.278306	chr1	1795408
cg19694519	-0.05425	-1.86031	0.063867	0.278441	chr8	22389206
cg03412547	0.076506	1.859402	0.063997	0.278654	chr16	4057728
cg07633435	0.062367	1.858568	0.064116	0.278897	chr5	152869009
cg19033906	0.050279	1.856383	0.064427	0.279496	chr3	53532972
cg12012319	0.060751	1.856268	0.064444	0.279519	chr7	126675528
cg04929165	0.05285	1.855952	0.064489	0.279607	chr1	186807235
cg25961618	0.063125	1.855388	0.06457	0.279763	chr11	35360531
cg11803392	0.067924	1.852482	0.064987	0.280612	chr17	64712432
cg01814344	0.068596	1.848967	0.065494	0.2816	chr5	178413313
cg09134640	0.053528	1.847746	0.065672	0.282007	chr1	37337877
cg14597908	0.039389	1.847352	0.065729	0.282124	chr20	57414960
cg08037774	0.052705	1.847232	0.065746	0.282154	chr1	53608962
cg09050775	0.070064	1.847124	0.065762	0.282195	chr12	46762708
cg26472511	0.067427	1.846957	0.065786	0.282246	chr16	4034240
cg04521626	-0.05156	-1.84326	0.066326	0.28343	chr17	4714200
cg00586732	0.068803	1.842484	0.066439	0.283673	chr3	7755472
cg04658038	0.055268	1.842121	0.066493	0.283772	chr17	64800166
cg12544392	0.037206	1.838643	0.067005	0.284698	chr19	42544587
cg17642145	-0.10827	-1.83844	0.067035	0.284769	chr8	22298578
cg25000623	-0.07301	-1.83812	0.067082	0.28483	chr17	72848918
cg03078593	0.069108	1.837585	0.067161	0.28495	chr12	26789311
cg14235271	0.078322	1.835314	0.067497	0.285626	chr20	57462812
cg27541048	0.060372	1.834299	0.067649	0.28595	chr12	6954816
cg07284407	0.046005	1.830299	0.068246	0.28711	chr20	57429858
cg19942459	-0.14564	-1.8266	0.068803	0.288167	chr11	70508110
cg08809418	-0.07822	-1.82601	0.068892	0.288332	chr12	56881865
cg02035425	-0.07564	-1.82425	0.069157	0.28885	chr9	140062632
cg00701890	0.043736	1.824225	0.069161	0.28885	chr16	4102293
cg21844450	-0.07176	-1.82378	0.069229	0.288965	chr20	8112956
cg19385628	-0.08079	-1.82312	0.069328	0.289156	chr3	6904640
cg10956480	-0.1109	-1.82234	0.069448	0.289406	chr12	2338945
cg06872721	0.057745	1.821592	0.069562	0.289595	chr19	19040258
cg06758670	-0.06664	-1.81854	0.070028	0.290394	chr16	10276383
cg24801123	-0.08931	-1.81832	0.070061	0.290435	chr7	45615503
cg02327001	0.117529	1.817163	0.070239	0.290764	chr4	101972762
cg14653281	-0.10076	-1.81639	0.070357	0.290988	chr9	104500954
cg03896542	0.058242	1.815171	0.070545	0.291326	chr16	56378687
cg02937055	0.098964	1.814631	0.070628	0.291472	chr3	171489625
cg04440551	0.078516	1.814225	0.070691	0.291572	chr2	25051151
cg25803139	0.062613	1.813294	0.070835	0.291831	chr19	2539626
cg04119977	0.067896	1.810596	0.071253	0.292675	chr5	7826972
cg17509967	-0.09546	-1.81012	0.071327	0.292827	chr19	13617094
cg12933359	0.058449	1.809659	0.071398	0.292989	chr7	86416314
cg11306735	0.082597	1.808544	0.071572	0.293316	chr19	49943812

cg15704369	-0.06445	-1.80786	0.071679	0.293504	chr1	84543558
cg13135654	0.056137	1.80689	0.07183	0.293771	chr11	70842116
cg19622911	-0.07164	-1.80588	0.071989	0.293997	chr18	3771570
cg14919164	0.101551	1.804742	0.072167	0.294271	chr7	45729156
cg09031790	0.056	1.802352	0.072543	0.294941	chr16	24129948
cg21518089	-0.06697	-1.80177	0.072635	0.295116	chr11	22362708
cg00161247	0.061892	1.801756	0.072637	0.295116	chr9	140060986
cg08685096	-0.08126	-1.79869	0.073122	0.295974	chr21	31312643
cg20950146	0.062728	1.794747	0.07375	0.297122	chr5	7827524
cg26801014	-0.08062	-1.7943	0.073821	0.297271	chr11	88241769
cg20315739	0.058857	1.79069	0.074401	0.298368	chr15	42303032
cg09436713	-0.05931	-1.78968	0.074563	0.298621	chr12	2323135
cg19621460	-0.06541	-1.78864	0.074731	0.298867	chr19	14225945
cg26985666	-0.08659	-1.78787	0.074855	0.299119	chr11	35441088
cg19727439	-0.1451	-1.78715	0.074972	0.299369	chr1	37500508
cg03047070	0.048253	1.783461	0.075572	0.300413	chr12	2787827
cg07636145	0.099246	1.783171	0.075619	0.300519	chr16	56228188
cg06432462	-0.08555	-1.78266	0.075703	0.300705	chr9	80646879
cg03650282	0.073994	1.782146	0.075786	0.300873	chr17	64451448
cg15527678	0.048142	1.781307	0.075924	0.301104	chr12	49171898
cg03324851	0.049541	1.781096	0.075958	0.301158	chr7	100274414
cg16468914	-0.18371	-1.78099	0.075976	0.301176	chr3	50242735
cg22620614	0.06676	1.780326	0.076084	0.301357	chr11	70794709
cg11025960	0.060498	1.776249	0.076755	0.302649	chr3	51749188
cg02306526	0.054923	1.774442	0.077054	0.303216	chr12	2202821
cg12691534	-0.05621	-1.77352	0.077207	0.303517	chr3	50275394
cg15329866	0.070793	1.772881	0.077313	0.303669	chr3	171455826
cg13856573	0.051152	1.77251	0.077374	0.303797	chr11	62475078
cg11046772	0.060041	1.77042	0.077723	0.304399	chr12	2353479
cg02066343	0.059069	1.768637	0.07802	0.304956	chr6	33590458
cg20103018	0.052272	1.767206	0.07826	0.305405	chr6	33996522
cg15122327	0.094631	1.765162	0.078604	0.306067	chr20	57435146
cg00036258	0.063628	1.763463	0.07889	0.306602	chr16	4029218
cg21931938	-0.0564	-1.76188	0.079157	0.307036	chr15	40600493
cg04576607	0.060281	1.759225	0.079609	0.307774	chr1	235787279
cg04496615	-0.07683	-1.75869	0.0797	0.307922	chr2	68479620
cg15245951	-0.06346	-1.75765	0.079877	0.3082	chr3	50283471
cg23475045	0.051374	1.757258	0.079944	0.308332	chr3	7248510
cg26334023	0.081465	1.756496	0.080074	0.308552	chr17	47287492
cg01091261	0.08826	1.756459	0.08008	0.308565	chr16	4029363
cg05340882	-0.13179	-1.75529	0.080281	0.308938	chr19	2543750
cg06112910	0.089405	1.755068	0.080319	0.309012	chr6	33995939
cg02295678	0.0811	1.754908	0.080346	0.309053	chr17	7123415
cg13298384	0.055511	1.753967	0.080507	0.309247	chr19	42546648
cg04525943	-0.06843	-1.75201	0.080843	0.309877	chr19	2579529
cg05362860	0.066332	1.750282	0.081142	0.310356	chr16	50320692
cg08325885	0.068607	1.749426	0.08129	0.310584	chr8	22297108
cg23232299	0.096979	1.742833	0.082438	0.312589	chr1	68212170

cg03425609	0.047918	1.741245	0.082716	0.313107	chr12	6952374
cg22849059	-0.09162	-1.74073	0.082806	0.31329	chr12	56882693
cg00587834	-0.0937	-1.74033	0.082877	0.313405	chr3	51749959
cg16128363	0.051021	1.740251	0.082891	0.313426	chr18	3880558
cg09448677	0.150289	1.737399	0.083393	0.314261	chr11	70672740
cg02738298	0.041062	1.737045	0.083456	0.314386	chr3	51749852
cg23911372	0.09422	1.736451	0.083561	0.314563	chr19	42546731
cg27066052	0.054603	1.736319	0.083584	0.31459	chr16	4015761
cg08289346	0.048308	1.735259	0.083772	0.314953	chr6	34089350
cg15852446	0.117699	1.733997	0.083996	0.31538	chr15	40583422
cg26229990	0.105564	1.732635	0.084239	0.3158	chr14	24801301
cg07340423	-0.06657	-1.73245	0.084271	0.315869	chr15	52472383
cg07823688	0.116841	1.731495	0.084442	0.316096	chr16	56310091
cg18851100	0.060146	1.730026	0.084704	0.316663	chr22	51158550
cg22967080	-0.05017	-1.72583	0.085459	0.317993	chr14	52333365
cg02105211	0.065873	1.724425	0.085712	0.318534	chr3	4625188
cg16777106	0.061661	1.723851	0.085815	0.318699	chr4	158281194
cg02026498	0.083115	1.721772	0.086192	0.319341	chr19	47139338
cg08576827	0.108181	1.721732	0.086199	0.319346	chr20	9075493
cg04926767	0.051625	1.72068	0.08639	0.319663	chr11	62476194
cg09190579	-0.12895	-1.7198	0.08655	0.31995	chr7	79763888
cg04016326	-0.10134	-1.71915	0.086669	0.320163	chr12	14132940
cg04348872	0.057394	1.718518	0.086784	0.320323	chr2	25141696
cg26621408	0.060783	1.715785	0.087284	0.321127	chr7	100275863
cg06996175	0.055561	1.715356	0.087363	0.321243	chr19	2546877
cg11281320	0.05007	1.712239	0.087937	0.322099	chr11	120553251
cg16677144	-0.05785	-1.71112	0.088144	0.322398	chr1	1790217
cg01538522	0.046136	1.709527	0.088438	0.322853	chr20	57463974
cg20646491	0.069943	1.70768	0.088781	0.323431	chr5	36608769
cg16312514	0.086878	1.707611	0.088794	0.323456	chr11	70650521
cg07217499	0.056245	1.707112	0.088887	0.323644	chr12	2416339
cg07287255	-0.07637	-1.70617	0.089062	0.323962	chr16	56374688
cg03861217	0.066499	1.704923	0.089295	0.324309	chr2	155652401
cg22798925	0.033924	1.704068	0.089455	0.324586	chr20	57464129
cg04763994	0.049837	1.70147	0.089942	0.325372	chr19	42506259
cg12046677	0.052756	1.701205	0.089991	0.325461	chr12	2360663
cg08599266	-0.0722	-1.70001	0.090215	0.3258	chr2	25142473
cg00866976	-0.13589	-1.69637	0.090904	0.326902	chr16	56224782
cg25885280	-0.12361	-1.69559	0.091052	0.327101	chr11	70760166
cg08480458	-0.07002	-1.69401	0.091353	0.327554	chr3	53529655
cg01231108	-0.08136	-1.69363	0.091425	0.327695	chr2	155556016
cg27418217	-0.04663	-1.69186	0.091762	0.328216	chr15	83518427
cg20110535	0.04287	1.690599	0.092003	0.328576	chr19	14225647
cg26726141	0.070503	1.690084	0.092102	0.328713	chr17	64612159
cg03300177	0.08723	1.688623	0.092382	0.329225	chr16	56390811
cg07036561	0.047096	1.686229	0.092842	0.329925	chr15	42118869
cg18247436	-0.05862	-1.68436	0.093203	0.330576	chr11	120856646
cg04062190	0.06202	1.68433	0.093209	0.33058	chr7	86413438

cg23159236	0.054417	1.684125	0.093249	0.330656	chr20	57464002
cg10668781	0.047826	1.68314	0.093439	0.330987	chr12	2307325
cg23153707	-0.0615	-1.68312	0.093443	0.330987	chr17	7121040
cg03723730	0.043309	1.682668	0.093531	0.331138	chr6	34031694
cg01364969	0.04953	1.681175	0.093821	0.331612	chr16	56389029
cg03319638	0.046793	1.678814	0.094281	0.332302	chr15	42387193
cg24276988	0.092778	1.678296	0.094382	0.332496	chr20	57463106
cg10804438	0.056908	1.677895	0.09446	0.33262	chr3	51747196
cg11971789	0.04932	1.675979	0.094835	0.333228	chr19	42545623
cg13327846	-0.06315	-1.67532	0.094965	0.333423	chr15	52472389
cg02598335	0.054854	1.675259	0.094976	0.333439	chr7	86337918
cg07176385	0.105053	1.675186	0.094991	0.33345	chr5	7397756
cg06512271	-0.0593	-1.67392	0.09524	0.333891	chr5	7394927
cg06828043	0.057618	1.67262	0.095496	0.33432	chr11	70398862
cg10453337	0.067229	1.672029	0.095612	0.334492	chr5	7502014
cg09830455	0.096953	1.671745	0.095668	0.334552	chr11	70886197
cg20090108	-0.15805	-1.67116	0.095784	0.334748	chr11	88242488
cg21213853	-0.13515	-1.66996	0.096022	0.335123	chr3	51741473
cg16623098	0.261031	1.669802	0.096053	0.335143	chr16	56374383
cg15417641	0.120054	1.668414	0.096328	0.335576	chr3	53700141
cg16543027	-0.06216	-1.6666	0.096688	0.336197	chr15	40599680
cg00147943	0.061546	1.66352	0.097304	0.337095	chr1	68225713
cg09927287	0.128216	1.659745	0.098062	0.338328	chr15	42447989
cg10698424	-0.06203	-1.65866	0.09828	0.338672	chr9	114423570
cg24250393	-0.06987	-1.65744	0.098527	0.338981	chr16	23846838
cg22804770	0.046306	1.65586	0.098847	0.339483	chr12	2786316
cg27179693	-0.05256	-1.65359	0.099307	0.34026	chr11	120530818
cg17826344	-0.07731	-1.652	0.099631	0.340755	chr19	51169660
cg20008140	0.079508	1.650786	0.09988	0.341116	chr20	57463455
cg08471319	0.071925	1.650588	0.09992	0.341191	chr2	25141735
cg06439941	0.077107	1.64905	0.100235	0.341712	chr7	93550756
cg00369202	0.056373	1.648741	0.100299	0.341797	chr6	33989844
cg23501962	-0.07132	-1.64858	0.100332	0.341845	chr11	35440252
cg09655520	0.040274	1.647594	0.100534	0.342095	chr17	64786064
cg04993286	0.068507	1.647081	0.10064	0.342228	chr16	4027595
cg22374237	-0.06706	-1.64488	0.101093	0.342948	chr7	126891197
cg14654306	-0.07009	-1.64292	0.101499	0.34356	chr1	186798107
cg15674997	-0.06068	-1.64198	0.101692	0.343896	chr5	178421314
cg17800870	0.114566	1.640749	0.101949	0.344373	chr1	182362757
cg00110769	0.079285	1.640537	0.101993	0.344459	chr1	110122089
cg13647052	-0.05525	-1.64053	0.101995	0.344459	chr12	2800382
cg08535918	0.045968	1.639273	0.102256	0.344935	chr16	56256748
cg22758916	0.0484	1.638447	0.102428	0.345229	chr15	42303292
cg12913957	-0.07764	-1.63742	0.102642	0.34558	chr6	33589131
cg11797430	0.047243	1.635938	0.102953	0.346041	chr12	2761364
cg21146273	0.079655	1.635588	0.103026	0.346118	chr5	152869193
cg27074174	-0.06825	-1.63513	0.103121	0.346227	chr6	101847318
cg24043604	0.049294	1.633651	0.103432	0.346704	chr12	2613948

cg00521993	0.059944	1.633343	0.103497	0.346857	chr10	75199411
cg03109047	-0.06807	-1.62988	0.104228	0.347889	chr9	140032686
cg10410142	-0.12497	-1.62895	0.104425	0.348235	chr4	158141542
cg05029189	-0.07905	-1.62686	0.104869	0.348918	chr3	123168386
cg06025216	0.050557	1.626179	0.105013	0.34914	chr16	4164891
cg18845797	0.060634	1.624393	0.105394	0.34964	chr5	7794275
cg00160359	0.048658	1.623965	0.105485	0.349779	chr12	49178192
cg03938110	0.069321	1.623098	0.10567	0.350086	chr21	31120485
cg03527683	0.06028	1.622589	0.105779	0.350285	chr7	126752816
cg00515755	0.070214	1.622192	0.105864	0.350383	chr19	1005248
cg16701848	0.052342	1.614722	0.107474	0.352727	chr19	2588479
cg02441543	0.100668	1.614531	0.107515	0.352806	chr22	51157530
cg14870792	-0.07102	-1.61417	0.107594	0.352902	chr12	2163532
cg16069986	0.07392	1.613687	0.107698	0.353028	chr11	70650456
cg20439283	0.047917	1.613367	0.107768	0.353139	chr5	178411249
cg08458678	0.044783	1.613268	0.107789	0.353171	chr11	70565177
cg16564940	-0.06831	-1.61097	0.10829	0.353902	chr17	7117310
cg17329110	0.121186	1.610211	0.108455	0.354179	chr11	70708812
cg05666036	0.125784	1.604	0.109818	0.356055	chr6	101848236
cg18411150	-0.06454	-1.6025	0.110148	0.356452	chr19	51172144
cg00317626	0.06274	1.600251	0.110647	0.357167	chr1	53600741
cg05432017	-0.05345	-1.60001	0.110701	0.357252	chr15	42119684
cg16261581	0.077622	1.598939	0.110938	0.35754	chr1	84972327
cg04002822	0.067374	1.598539	0.111027	0.357686	chr12	2483094
cg06728579	0.130213	1.59813	0.111118	0.357833	chr16	56224901
cg23165500	0.04589	1.59783	0.111185	0.357942	chr5	152949095
cg23409374	-0.09842	-1.5974	0.11128	0.358126	chr19	49934742
cg27431037	0.097103	1.597251	0.111314	0.358165	chr12	2330387
cg23580000	-0.05077	-1.59614	0.111562	0.358546	chr16	50322156
cg26863600	-0.04827	-1.59437	0.111957	0.359201	chr19	2616921
cg14643330	-0.05434	-1.59273	0.112325	0.359827	chr3	4534051
cg25185429	0.039926	1.59254	0.112368	0.359909	chr3	4739461
cg19256368	0.048407	1.590885	0.11274	0.360494	chr15	83619037
cg08288223	0.074238	1.590756	0.112769	0.360518	chr11	70563131
cg13343565	0.138646	1.590588	0.112807	0.360568	chr17	4710032
cg01833890	0.029667	1.590122	0.112912	0.360695	chr12	2564063
cg22147917	0.053978	1.588982	0.113169	0.361041	chr6	33656031
cg27012424	0.076159	1.586744	0.113676	0.361755	chr18	3773224
cg15056189	0.050537	1.584899	0.114094	0.362457	chr12	49176428
cg09772382	0.024591	1.584819	0.114113	0.362488	chr20	57463775
cg00020720	-0.04926	-1.58428	0.114236	0.362675	chr1	84972482
cg06517489	-0.05356	-1.58395	0.11431	0.362811	chr11	22359333
cg10371483	0.062122	1.581395	0.114893	0.363632	chr16	4152045
cg24902435	0.051624	1.580586	0.115078	0.36392	chr12	2790095
cg13370485	0.043941	1.580471	0.115104	0.363964	chr12	2762840
cg20809470	-0.05966	-1.57889	0.115467	0.364447	chr11	22364837
cg19930620	0.071782	1.57805	0.115659	0.364724	chr3	7340148
cg05876496	0.052509	1.576952	0.115912	0.365055	chr6	33638550

cg14728235	0.029979	1.574581	0.116459	0.365752	chr20	57415177
cg26912314	0.05269	1.572519	0.116936	0.366527	chr16	56291781
cg15728692	0.05649	1.570929	0.117305	0.367046	chr22	51135736
cg01866630	-0.0608	-1.56844	0.117885	0.367844	chr6	33601722
cg21269738	0.040852	1.568407	0.117892	0.367856	chr12	2356703
cg15486374	-0.0539	-1.5648	0.118736	0.369052	chr15	83621710
cg07166235	-0.06013	-1.56374	0.118985	0.369359	chr12	49183018
cg24287125	-0.05855	-1.56329	0.11909	0.369492	chr12	2692308
cg01118752	0.100936	1.562137	0.119362	0.369833	chr4	102264326
cg20557935	0.04043	1.560074	0.119848	0.370585	chr15	52484322
cg13599596	0.07072	1.557678	0.120415	0.371286	chr9	4541807
cg24719827	-0.0521	-1.55592	0.120833	0.371748	chr1	110091174
cg22600443	0.069255	1.5536	0.121386	0.372552	chr15	42388240
cg22746789	-0.05674	-1.55325	0.121469	0.372671	chr19	2579221
cg24944109	-0.11441	-1.55304	0.12152	0.372738	chr11	35440136
cg22960869	0.085522	1.552556	0.121635	0.372923	chr17	47287521
cg22060073	-0.08856	-1.55217	0.121727	0.373062	chr8	132052942
cg05492714	0.070459	1.551773	0.121822	0.37321	chr9	140042286
cg21370856	-0.05908	-1.55128	0.121941	0.373349	chr16	23848003
cg01895482	0.049953	1.550516	0.122124	0.373547	chr19	2556145
cg00788521	0.06175	1.55035	0.122163	0.373599	chr12	2229269
cg03821543	0.041427	1.549705	0.122318	0.373793	chr20	57463925
cg19747632	0.044741	1.549541	0.122358	0.373823	chr15	52472703
cg06996976	0.040959	1.547952	0.12274	0.374426	chr17	64576105
cg04029168	-0.05578	-1.54657	0.123073	0.374907	chr16	23963538
cg03466124	-0.06361	-1.54577	0.123267	0.375202	chr3	179168156
cg26459372	-0.08524	-1.54565	0.123295	0.37526	chr7	45613676
cg02218260	0.058752	1.545152	0.123415	0.3754	chr3	51742878
cg07386190	-0.06069	-1.54505	0.123441	0.375426	chr1	182361453
cg09067029	0.053593	1.541847	0.124216	0.376649	chr1	186954259
cg00390253	0.050229	1.540871	0.124454	0.377012	chr3	50241300
cg24603235	0.091418	1.539324	0.124831	0.377521	chr6	33588219
cg03173525	-0.0566	-1.5391	0.124886	0.37757	chr7	100273221
cg07178968	0.041027	1.538557	0.125018	0.377741	chr15	42130662
cg17658854	0.043576	1.536524	0.125516	0.378365	chr20	57462798
cg23494413	0.048408	1.534898	0.125914	0.379009	chr12	6954534
cg04156464	-0.08105	-1.53467	0.125969	0.379057	chr16	56224504
cg20582984	0.041259	1.533595	0.126235	0.379454	chr20	57417233
cg23202253	0.070324	1.533443	0.126272	0.379484	chr12	26902211
cg03885818	0.084562	1.533075	0.126363	0.379568	chr12	2299830
cg07091154	0.048687	1.53169	0.126704	0.380015	chr11	70562728
cg10037905	-0.09095	-1.52923	0.127314	0.380843	chr12	26986999
cg10738479	-0.05354	-1.52682	0.12791	0.381773	chr19	1000105
cg15425921	0.078696	1.526617	0.127961	0.381866	chr19	2643085
cg26337841	0.051796	1.52652	0.127986	0.381898	chr1	37329331
cg20917920	0.046676	1.526033	0.128107	0.382038	chr11	70416238
cg25976563	-0.04818	-1.52578	0.12817	0.382112	chr3	179169592
cg05806233	-0.05612	-1.52445	0.128502	0.382605	chr7	79763748

cg10748817	0.052869	1.524356	0.128525	0.382634	chr20	57465175
cg17106653	-0.06669	-1.52338	0.128767	0.383025	chr19	48897279
cg17125585	0.049053	1.522724	0.128933	0.383294	chr1	68176471
cg17006204	0.059408	1.52234	0.129029	0.38342	chr11	70448210
cg02624701	-0.12575	-1.52181	0.129161	0.383587	chr19	49937176
cg13802605	0.045013	1.521552	0.129226	0.383669	chr9	4495359
cg07121488	0.041551	1.521174	0.129321	0.383784	chr15	40581105
cg16480969	0.041567	1.519182	0.129821	0.384402	chr6	33996203
cg07267600	0.046836	1.518757	0.129928	0.384541	chr12	2750053
cg03773989	-0.04838	-1.51867	0.129951	0.384598	chr10	75255862
cg10159951	-0.05764	-1.51827	0.130052	0.38474	chr11	35441881
cg05248742	-0.06746	-1.51663	0.130466	0.385322	chr16	24142386
cg02984142	0.133291	1.516074	0.130605	0.385529	chr1	84971681
cg09991710	0.042875	1.515383	0.13078	0.385749	chr1	37472559
cg24319825	-0.14636	-1.51446	0.131015	0.386091	chr3	4534939
cg12467435	0.04763	1.509418	0.132296	0.387834	chr17	64651126
cg18389339	0.093941	1.506603	0.133016	0.388811	chr18	3730593
cg03973705	0.041219	1.505785	0.133226	0.389112	chr16	24174850
cg16307325	0.05762	1.5057	0.133248	0.389155	chr12	2339235
cg09554596	0.061534	1.505116	0.133398	0.389384	chr19	1004620
cg04933990	-0.08663	-1.50478	0.133484	0.389528	chr16	10133501
cg17652507	0.057089	1.503356	0.133851	0.39002	chr20	57463653
cg07080031	0.151767	1.502687	0.134023	0.39023	chr11	70675170
cg05414613	0.058368	1.50102	0.134454	0.390842	chr1	53558470
cg00336149	0.076877	1.500863	0.134494	0.390879	chr3	53700195
cg03371918	-0.06419	-1.49581	0.135806	0.392691	chr17	64297988
cg00534626	0.08917	1.495685	0.135839	0.392749	chr16	9864730
cg13883984	-0.05489	-1.49455	0.136135	0.393143	chr15	83621779
cg01103812	0.094423	1.494398	0.136174	0.393198	chr12	26986269
cg25638611	-0.13293	-1.49254	0.136659	0.393779	chr11	70508420
cg23398700	-0.05827	-1.4907	0.137142	0.394384	chr5	78808194
cg02602411	0.045432	1.490116	0.137296	0.394613	chr9	104357177
cg16899036	0.04018	1.486241	0.138317	0.395884	chr19	19052705
cg07502066	-0.1241	-1.48395	0.138924	0.396691	chr1	110091086
cg05065846	0.04421	1.483642	0.139005	0.396785	chr7	126885001
cg05166022	-0.06177	-1.4832	0.139122	0.396965	chr12	2800471
cg15827003	0.10907	1.479613	0.140077	0.398245	chr1	1822912
ch.5.240336F	-0.15906	-1.47855	0.14036	0.398631	chr5	7757969
cg01007458	0.044684	1.478252	0.140441	0.398722	chr19	15084527
cg01355739	0.039455	1.476972	0.140783	0.399186	chr20	57416888
cg13523713	-0.0732	-1.4764	0.140937	0.399385	chr19	48614733
cg02624051	0.096156	1.476385	0.140941	0.399386	chr15	42371635
cg27433516	0.050867	1.476014	0.14104	0.399533	chr1	37266621
cg08982381	-0.05603	-1.47594	0.141062	0.399562	chr9	80647290
cg02640306	-0.05923	-1.47532	0.141226	0.399772	chr2	191745287
cg06614951	-0.0558	-1.47398	0.141588	0.400155	chr19	14229385
cg01686093	0.042001	1.471525	0.142249	0.400968	chr11	70491582
cg19343464	-0.06064	-1.47102	0.142385	0.401169	chr11	105481509

cg11692123	-0.04738	-1.47045	0.142539	0.401332	chr11	70935949
cg14123942	-0.10804	-1.4693	0.142851	0.401739	chr9	104500322
cg24753760	-0.07168	-1.46816	0.143161	0.402152	chr6	101846767
cg09530407	0.059628	1.468141	0.143165	0.402157	chr11	22359486
cg12054318	0.03335	1.468019	0.143198	0.402208	chr20	57414529
cg06783533	0.056466	1.466449	0.143625	0.402768	chr16	56388908
cg19088553	0.088721	1.465756	0.143813	0.403023	chr6	101901884
cg20920827	0.06215	1.465717	0.143824	0.403038	chr11	70858475
cg09558195	-0.0812	-1.4652	0.143966	0.403201	chr6	146350585
cg07587653	0.047762	1.462909	0.14459	0.404025	chr11	70338480
cg02794451	-0.0545	-1.46204	0.144829	0.404327	chr12	2800446
cg10474377	0.051217	1.461638	0.144938	0.40446	chr15	42131658
cg08747970	0.047102	1.461086	0.14509	0.404608	chr11	70666452
cg13823003	-0.04143	-1.45793	0.145956	0.405809	chr17	72856049
cg11996914	0.022704	1.457504	0.146074	0.405977	chr20	57414578
cg02808075	-0.08762	-1.45568	0.146579	0.406641	chr3	4534881
cg12452386	0.042363	1.454856	0.146806	0.406924	chr15	42302500
cg02725014	-0.05775	-1.45419	0.146992	0.407133	chr5	78809520
cg01111718	0.048711	1.453587	0.147157	0.407383	chr11	105762374
cg13588054	0.061241	1.451849	0.14764	0.408032	chr7	126892578
cg20492121	-0.05471	-1.45159	0.147712	0.408114	chr1	182361258
cg03832839	-0.08191	-1.44831	0.148626	0.409249	chr19	54401967
cg06952307	-0.05376	-1.44804	0.148702	0.40931	chr17	47287974
cg09890339	0.04609	1.447803	0.148768	0.409381	chr12	2734150
cg05632631	0.045775	1.447772	0.148776	0.409386	chr16	24099614
cg26534489	-0.05421	-1.44597	0.14928	0.410047	chr20	57427495
cg06085579	0.053066	1.445035	0.149543	0.410397	chr3	171509822
cg13830799	0.061072	1.444676	0.149644	0.410496	chr6	146750462
cg15464481	0.051339	1.442795	0.150173	0.411191	chr3	123151962
cg04269530	-0.07192	-1.44252	0.15025	0.411281	chr19	48919401
cg01141838	0.035914	1.439216	0.151183	0.412439	chr11	62474744
cg11706780	0.041913	1.436646	0.151912	0.413452	chr15	42289766
cg21809160	0.0487	1.435118	0.152346	0.414023	chr20	57428309
cg01617139	-0.07784	-1.43412	0.152631	0.414417	chr19	2543861
cg20748533	-0.0704	-1.4337	0.152752	0.414531	chr19	51189975
cg21102121	-0.08772	-1.43163	0.153341	0.415362	chr19	54401295
cg07408989	0.039229	1.428823	0.154146	0.416367	chr17	7116792
cg02736560	0.037229	1.428049	0.154369	0.416611	chr1	68257754
cg17839232	0.086559	1.427369	0.154564	0.416834	chr5	36686601
cg10623198	0.035788	1.423408	0.155708	0.418403	chr12	6949114
cg00050312	-0.05015	-1.42298	0.155832	0.418542	chr17	64299065
cg20782596	0.045293	1.42198	0.156122	0.418859	chr20	57462978
cg13624528	0.101613	1.420477	0.156558	0.419449	chr6	101846409
cg09551072	0.031475	1.419414	0.156867	0.419934	chr1	53567678
cg18295203	-0.05243	-1.41854	0.157121	0.42024	chr3	171528273
cg08445323	0.06662	1.415909	0.15789	0.421172	chr16	4015030
cg09529437	-0.04107	-1.41587	0.157902	0.42118	chr16	24136792
cg03616148	0.088574	1.414391	0.158335	0.42168	chr16	9911529

cg16919771	-0.04202	-1.41199	0.159041	0.42261	chr7	126892599
cg07688749	-0.03839	-1.41165	0.159141	0.422737	chr12	46767132
cg23696752	-0.08473	-1.41043	0.159499	0.423222	chr6	146349312
cg23491599	-0.09227	-1.40811	0.160183	0.42398	chr2	155554688
cg11802781	0.058602	1.408095	0.160189	0.423985	chr11	70370534
cg12276123	-0.07993	-1.408	0.160216	0.424015	chr2	155555157
cg26011633	0.048654	1.407709	0.160303	0.424105	chr5	178411667
cg26952925	0.127696	1.4044	0.161285	0.425402	chr16	4166391
cg26379672	-0.04535	-1.40139	0.162181	0.426374	chr3	4534954
cg15802396	-0.06018	-1.4012	0.16224	0.426465	chr17	7108305
cg13861294	0.064257	1.40043	0.162469	0.426698	chr12	100750473
cg05794931	-0.0804	-1.39892	0.16292	0.427306	chr11	70493931
cg01716959	0.048498	1.398301	0.163106	0.427523	chr11	70515714
cg20645074	-0.06533	-1.39821	0.163133	0.427559	chr12	49182479
cg14526297	-0.05741	-1.39681	0.163554	0.42806	chr6	146349488
cg17789138	-0.05816	-1.39678	0.163563	0.428074	chr19	49936880
cg13971030	0.126009	1.396778	0.163563	0.428074	chr11	35366721
cg14767950	-0.05167	-1.39654	0.163633	0.428139	chr19	49939773
cg14158769	0.118603	1.396484	0.163652	0.428157	chr7	126698156
cg18950779	0.056417	1.396279	0.163713	0.428233	chr17	64685057
cg21797718	0.074987	1.395835	0.163847	0.428387	chr6	34024416
cg17665552	0.083517	1.394665	0.164199	0.428826	chr11	70455599
cg10577016	-0.08409	-1.39382	0.164454	0.429134	chr9	114423680
cg25047001	-0.04922	-1.39376	0.164473	0.429151	chr16	10277017
cg19242688	0.098245	1.393597	0.164521	0.42921	chr19	47139391
cg08997444	0.033601	1.392611	0.164819	0.429578	chr20	57464970
cg21392385	-0.04105	-1.39217	0.164951	0.429732	chr15	52428589
cg22692013	-0.04904	-1.39096	0.165319	0.430181	chr12	2354618
cg22133366	-0.06251	-1.39086	0.16535	0.430236	chr11	70385327
cg22065976	-0.04685	-1.39039	0.16549	0.430427	chr6	33589061
cg13411554	0.145076	1.390141	0.165567	0.430526	chr3	53700276
cg27629673	0.043834	1.390043	0.165597	0.430579	chr5	7462856
cg25762078	0.045041	1.387791	0.166281	0.431411	chr1	53554504
cg00267746	0.038092	1.385121	0.167095	0.432335	chr20	57463984
cg13591723	-0.04685	-1.382	0.168051	0.433404	chr12	46765135
cg18318307	0.04387	1.380429	0.168533	0.433963	chr11	70368307
cg08464513	0.049753	1.378459	0.169139	0.434715	chr16	30136024
cg01041222	-0.06479	-1.37559	0.170024	0.435738	chr4	158142863
cg11820929	0.069773	1.371573	0.171272	0.437005	chr4	102258631
cg27369423	-0.04707	-1.37117	0.171397	0.437175	chr16	56228901
cg26904140	-0.06324	-1.36605	0.172995	0.439171	chr19	2703086
cg11803871	0.043702	1.364822	0.173382	0.439657	chr3	123039831
cg12076692	0.070693	1.363741	0.173722	0.440097	chr1	1718852
cg15916804	-0.04566	-1.36368	0.173742	0.440114	chr10	75255500
cg10259111	0.050091	1.360593	0.174714	0.441271	chr14	52429547
cg25904372	0.044123	1.359053	0.175201	0.441855	chr11	70628970
cg11021321	-0.06595	-1.35635	0.176059	0.442868	chr20	57471660
cg21188533	0.120562	1.355674	0.176272	0.443131	chr3	53700263

cg02071600	-0.05938	-1.3549	0.176517	0.443385	chr5	78808852
cg14851284	0.072316	1.354006	0.176804	0.443725	chr11	70713732
cg21885159	-0.04988	-1.35233	0.177337	0.444308	chr19	2645923
cg24995240	-0.07207	-1.35159	0.177574	0.444634	chr1	84971124
cg12159995	0.051714	1.350282	0.177993	0.44515	chr11	70398671
cg25451120	0.041523	1.349961	0.178096	0.445295	chr17	47287444
cg22983529	-0.05271	-1.34991	0.178113	0.445321	chr7	93551132
cg09858208	-0.06142	-1.34977	0.178157	0.445354	chr3	50283852
cg10272968	-0.04616	-1.34962	0.178206	0.445423	chr19	2611105
cg15639581	-0.04254	-1.34914	0.178359	0.445575	chr19	13318873
cg08525508	0.032104	1.347735	0.17881	0.446091	chr19	48920297
cg04127894	0.044131	1.34753	0.178876	0.446148	chr15	40594732
cg26503038	0.046084	1.345066	0.179669	0.447056	chr19	2699659
cg01864982	-0.05465	-1.3443	0.179916	0.447346	chr19	54409631
cg03242834	0.055729	1.344275	0.179925	0.447358	chr19	13317326
cg08111863	0.043236	1.342473	0.180507	0.448089	chr11	70882036
cg05884705	-0.05456	-1.34206	0.18064	0.448255	chr15	40600099
cg15083678	0.037454	1.340445	0.181165	0.448886	chr12	2724200
cg23055735	0.042057	1.339728	0.181397	0.449139	chr11	70557464
cg22522961	0.069881	1.339105	0.1816	0.449365	chr1	68288817
cg22598669	-0.07707	-1.33829	0.181865	0.449668	chr19	51186978
cg18384228	0.054678	1.337136	0.182241	0.450108	chr14	24789158
cg26672104	-0.04375	-1.3366	0.182415	0.450275	chr14	52327433
cg04988514	-0.10669	-1.33141	0.184115	0.452222	chr19	48947560
cg19120580	0.035029	1.331073	0.184225	0.452348	chr12	49173414
cg13355041	-0.05164	-1.32991	0.184609	0.452787	chr18	3593715
cg08897844	0.075154	1.329353	0.184791	0.452996	chr5	36606102
cg27073113	-0.04969	-1.32822	0.185163	0.453408	chr16	56228744
cg21633134	-0.05413	-1.32779	0.185305	0.453527	chr5	7816399
cg16739976	0.058489	1.327492	0.185405	0.453633	chr6	34101401
cg20984065	0.040698	1.325611	0.186027	0.454352	chr3	51745321
cg16163543	0.06481	1.323838	0.186614	0.455113	chr11	70449219
cg04761722	-0.08309	-1.32344	0.186747	0.455258	chr16	10274315
cg07571637	-0.0699	-1.32294	0.186913	0.455469	chr22	51143260
cg10486865	0.083092	1.322429	0.187082	0.455677	chr17	64355892
cg10116505	-0.05277	-1.3212	0.187491	0.456233	chr16	10274064
cg09157251	-0.25915	-1.32027	0.187802	0.45656	chr11	70733251
cg11669839	0.02356	1.319956	0.187905	0.456697	chr20	57426322
cg02274728	0.038152	1.318829	0.188281	0.457185	chr20	57414407
cg10891888	-0.05323	-1.3166	0.189027	0.458017	chr12	46765615
cg26361533	0.04782	1.316245	0.189146	0.458098	chr12	2445561
cg23725394	-0.05504	-1.3137	0.19	0.459102	chr2	25057656
cg22569496	0.118636	1.31366	0.190014	0.459126	chr19	13409671
cg23464041	-0.03184	-1.31139	0.19078	0.46002	chr6	33663938
cg19946638	0.062778	1.311085	0.190881	0.460096	chr11	70859647
cg01449218	-0.04424	-1.31099	0.190912	0.460133	chr7	86273083
cg10302550	-0.07069	-1.31043	0.191103	0.460389	chr20	57427821
cg24723883	0.033866	1.309775	0.191323	0.460702	chr19	2608495

cg02521996	-0.08428	-1.30929	0.191487	0.460875	chr16	30134825
cg09286797	-0.06255	-1.30915	0.191534	0.460924	chr20	8116999
cg11758458	0.038239	1.309025	0.191577	0.460974	chr17	64575021
cg20699497	-0.05861	-1.30859	0.191723	0.461122	chr19	49944642
cg21216562	-0.07107	-1.30416	0.193228	0.462817	chr9	140045508
cg19636672	0.059331	1.303908	0.193314	0.462906	chr12	56881121
cg16671069	-0.07798	-1.30052	0.19447	0.464241	chr6	146350969
cg12566890	0.034129	1.29868	0.1951	0.464964	chr5	7494531
cg01354782	0.048931	1.298473	0.195171	0.465061	chr12	26522758
cg04903912	0.043664	1.297841	0.195388	0.465296	chr11	70374289
cg10707626	0.044856	1.29763	0.19546	0.465389	chr3	51747098
cg00070899	0.108078	1.29697	0.195687	0.465593	chr6	34024479
cg20579012	0.031444	1.295762	0.196102	0.466063	chr12	2803823
cg12667048	0.053481	1.295329	0.196251	0.466262	chr11	70644526
cg00043510	-0.06493	-1.29487	0.19641	0.46646	chr1	235814134
cg25652859	-0.06826	-1.29388	0.19675	0.466815	chr20	57427412
cg15878555	-0.05324	-1.29262	0.197188	0.467252	chr11	62473962
cg03547757	0.045781	1.291341	0.197628	0.467806	chr20	57425515
cg14212966	0.05285	1.290476	0.197928	0.468126	chr1	53558392
cg10851168	0.04321	1.289079	0.198412	0.468673	chr11	70317508
cg08886546	-0.04543	-1.28768	0.198898	0.469189	chr19	19051335
cg22885821	-0.05167	-1.28749	0.198966	0.469275	chr20	57465921
cg09726240	0.079961	1.287477	0.198969	0.469275	chr11	70672878
cg07687951	0.063183	1.287181	0.199072	0.469411	chr11	70666560
cg20213508	0.04613	1.287014	0.19913	0.469485	chr20	57463325
cg15442907	-0.04643	-1.28693	0.199159	0.469506	chr12	2800463
cg00997853	-0.04936	-1.28643	0.199335	0.469703	chr12	26844579
cg09659734	-0.04923	-1.28537	0.199703	0.470183	chr16	56227065
cg24008901	0.073959	1.28433	0.200066	0.470557	chr12	13715497
cg00601648	0.053572	1.283973	0.200191	0.470683	chr8	131974587
cg27661264	-0.06264	-1.28261	0.200668	0.471118	chr20	57427738
cg12116020	-0.06495	-1.2809	0.201267	0.471802	chr19	14228622
cg21625881	0.02861	1.280409	0.201439	0.471939	chr20	57430313
cg24033471	-0.02486	-1.28	0.201582	0.472135	chr12	2735579
cg06194010	0.045998	1.279216	0.201858	0.472417	chr12	2493567
cg11480627	0.106535	1.279044	0.201918	0.472484	chr11	70672876
cg07746960	0.066222	1.27793	0.20231	0.472956	chr19	42546662
cg09555879	0.036763	1.276485	0.20282	0.473568	chr19	42569956
cg00765653	0.02035	1.276041	0.202976	0.473785	chr20	57415144
cg09320113	-0.04616	-1.27573	0.203087	0.473901	chr1	186799481
cg25338454	-0.03938	-1.27455	0.203503	0.474308	chr12	26900022
cg14648237	0.031062	1.273268	0.203957	0.474845	chr17	64422393
cg20936920	0.041464	1.272014	0.204402	0.475366	chr3	7782210
cg24824840	0.050126	1.270771	0.204843	0.475857	chr19	51219975
cg03399271	-0.04916	-1.27053	0.204929	0.475921	chr16	56228385
cg08166863	-0.02844	-1.27028	0.205018	0.476004	chr20	57426391
cg04473078	-0.09776	-1.26996	0.205133	0.476126	chr16	4165886
cg17222829	-0.04334	-1.26933	0.205357	0.476337	chr11	70433293

cg11830694	-0.05168	-1.26924	0.205388	0.47637	chr12	2289797
cg24086869	-0.04437	-1.26905	0.205454	0.476437	chr15	52471581
cg21350778	0.058873	1.26888	0.205516	0.476488	chr17	64297603
cg09742895	0.043227	1.266057	0.206523	0.477663	chr11	105781302
cg04803128	-0.04386	-1.26363	0.207392	0.478562	chr7	100273280
cg14396328	0.077916	1.262245	0.207889	0.479166	chr7	45761429
cg10482356	0.034616	1.261891	0.208016	0.479309	chr16	56328421
cg25521963	0.062624	1.260955	0.208353	0.479641	chr17	7123553
cg14208013	0.048972	1.26069	0.208448	0.479726	chr20	57485765
cg06932616	-0.07766	-1.25721	0.209705	0.481195	chr19	48908335
cg24009995	-0.06247	-1.25618	0.210077	0.481644	chr14	52434702
cg07700514	-0.10872	-1.25576	0.21023	0.48184	chr16	56228467
cg04446870	0.037054	1.255373	0.210368	0.482025	chr17	64440273
cg03306374	0.097853	1.254727	0.210602	0.48225	chr16	23847325
cg16620233	0.035388	1.25429	0.210761	0.48243	chr3	53781107
cg01331810	0.067845	1.252594	0.211377	0.483201	chr7	86414302
cg13914083	-0.06699	-1.2523	0.211483	0.483321	chr4	158141526
cg09017174	-0.05047	-1.25152	0.211768	0.483609	chr11	35440525
cg26613742	0.021034	1.251412	0.211807	0.483643	chr19	14225000
cg11953334	-0.04364	-1.24999	0.212325	0.484239	chr19	48897863
cg08505135	0.032345	1.249006	0.212684	0.484582	chr19	19045615
cg11692021	0.042774	1.249006	0.212684	0.484582	chr12	6949472
cg08021727	0.042002	1.246513	0.213596	0.485604	chr16	4056879
cg07218663	-0.07821	-1.24502	0.214142	0.486216	chr6	146350618
cg02240622	0.024588	1.244119	0.214474	0.486532	chr15	40601467
cg05279172	0.046151	1.243609	0.214662	0.486729	chr17	7113697
cg17846122	0.048784	1.243161	0.214827	0.4869	chr17	64351144
cg12544243	0.050164	1.24279	0.214963	0.487021	chr19	1005641
cg02954212	-0.05348	-1.24267	0.215006	0.487057	chr16	56226350
cg10820904	0.047595	1.242449	0.215089	0.487133	chr17	64412745
cg19794207	-0.04599	-1.24124	0.215536	0.487571	chr9	140040231
cg12240358	0.057624	1.240997	0.215624	0.487654	chr15	83619523
cg11906607	0.037457	1.239959	0.216007	0.488052	chr19	2524064
cg23956071	0.085272	1.239145	0.216308	0.488366	chr17	4710044
cg16400825	-0.04961	-1.23816	0.216672	0.488767	chr6	33589418
cg10671676	0.041276	1.237675	0.216852	0.488932	chr5	178413560
cg14090916	-0.06413	-1.23505	0.217827	0.489983	chr9	140044904
cg20822365	-0.09388	-1.23498	0.217853	0.490019	chr15	83621694
cg25423647	0.03683	1.234566	0.218006	0.490196	chr3	51746723
cg03264550	-0.08399	-1.2339	0.218254	0.490447	chr20	57465448
cg10503359	-0.07983	-1.23368	0.218334	0.490519	chr3	142468059
cg21636577	0.029752	1.233104	0.21855	0.490756	chr17	7121881
cg05185634	0.023623	1.233093	0.218554	0.490761	chr14	24804750
cg16600501	-0.07118	-1.2326	0.218738	0.490929	chr19	15083842
cg07926858	0.06325	1.232474	0.218784	0.490978	chr10	75252761
cg17726655	0.038684	1.231523	0.219139	0.4913	chr19	42509860
cg04260676	-0.03461	-1.23148	0.219156	0.491312	chr1	1774322
cg02328440	-0.04321	-1.23036	0.219574	0.491734	chr1	235813839

cg11155924	0.064253	1.229831	0.219771	0.491896	chr11	70449258
cg06874426	0.051759	1.229745	0.219803	0.49191	chr17	47287526
cg09152120	0.053424	1.225493	0.221398	0.493549	chr16	4016669
cg13016048	0.056939	1.224954	0.2216	0.49376	chr9	140054056
cg07024458	-0.04584	-1.22462	0.221727	0.493902	chr16	56390600
cg18046365	0.052252	1.22426	0.221862	0.494038	chr1	53608367
cg06864895	-0.04444	-1.22385	0.222015	0.494189	chr12	46767683
cg02288564	-0.1011	-1.22082	0.223161	0.495364	chr19	49934404
cg16601231	0.086087	1.218565	0.224014	0.496306	chr19	13367946
cg23740474	0.037078	1.218383	0.224083	0.496376	chr11	70455140
cg14992273	0.043027	1.218305	0.224112	0.496402	chr1	37337827
cg27318000	0.07824	1.216422	0.224827	0.497146	chr18	3845667
cg27643147	-0.0602	-1.21575	0.225082	0.497449	chr11	70517294
cg02953559	0.036754	1.212426	0.22635	0.498668	chr3	123164964
cg23613253	0.029026	1.21221	0.226433	0.498752	chr11	70440347
cg09997760	-0.04469	-1.20825	0.227948	0.500332	chr3	179169556
cg05161074	0.042345	1.204792	0.22928	0.501738	chr15	42289885
cg23965720	-0.04133	-1.20456	0.229369	0.501805	chr1	235805770
cg24151995	-0.05849	-1.20432	0.229461	0.5019	chr11	22364293
cg00383081	0.046285	1.203198	0.229895	0.502285	chr1	182362088
cg19324023	0.036035	1.202559	0.230142	0.502543	chr22	51170003
cg21830821	-0.05842	-1.20249	0.230167	0.502561	chr15	52475339
cg13000134	-0.04613	-1.19999	0.231137	0.503676	chr19	48896922
cg24339704	0.028034	1.199594	0.23129	0.503809	chr19	2529022
cg16009558	-0.06978	-1.19899	0.231522	0.50408	chr6	101846707
cg08038054	0.054222	1.198471	0.231726	0.504306	chr7	93550781
cg17119568	-0.06197	-1.19841	0.231751	0.504327	chr19	14229237
cg14203613	0.048924	1.195028	0.233066	0.505503	chr3	4714928
cg02991464	0.041088	1.194799	0.233155	0.505601	chr12	2788732
cg25193885	0.038695	1.193315	0.233735	0.506205	chr11	70328867
cg22290117	0.04229	1.1933	0.233741	0.506205	chr20	57427173
cg24092939	0.06521	1.192808	0.233933	0.506404	chr12	49181056
cg12664560	-0.11874	-1.19152	0.234435	0.50699	chr15	83621517
cg17159473	-0.04105	-1.19041	0.234871	0.507407	chr7	126893890
cg05397010	-0.06393	-1.1883	0.235702	0.508251	chr15	42448259
cg20772037	0.034748	1.187808	0.235895	0.508469	chr6	34067242
cg11435239	0.05204	1.186871	0.236263	0.508784	chr19	51220297
cg07451034	0.041553	1.186309	0.236485	0.509012	chr17	47283774
cg25534294	-0.0729	-1.186	0.236608	0.509113	chr2	155554931
cg08374499	0.067384	1.185689	0.236729	0.509214	chr19	49941241
cg08460548	-0.05622	-1.18561	0.23676	0.509244	chr19	49944817
cg13916928	0.040745	1.185188	0.236927	0.509413	chr11	62474735
cg12981270	0.040022	1.185047	0.236983	0.509462	chr19	42509946
cg19293162	0.035448	1.183907	0.237433	0.50994	chr19	2513302
cg05722993	0.036407	1.183297	0.237674	0.51018	chr1	1727796
cg16253976	0.061964	1.180876	0.238633	0.51121	chr5	7686199
cg11601336	0.027515	1.180713	0.238698	0.511259	chr19	19053012
cg03016097	-0.0384	-1.17951	0.239174	0.511843	chr15	83621726

cg26266429	0.026243	1.178465	0.239591	0.51225	chr12	49174651
cg19853565	0.039938	1.177997	0.239777	0.512431	chr19	2540907
cg22459924	-0.02987	-1.17747	0.239989	0.51262	chr19	2607850
cg01399255	0.081667	1.172756	0.24187	0.514618	chr7	100271274
cg09853822	0.039085	1.170616	0.242728	0.515562	chr17	4712456
cg13523557	-0.05951	-1.17015	0.242917	0.515763	chr7	45613725
cg05546044	0.049846	1.169674	0.243106	0.515979	chr22	22222597
cg06692957	-0.0456	-1.16807	0.243752	0.516647	chr9	80647629
cg24067803	0.032558	1.168012	0.243775	0.516662	chr11	70653378
cg09641955	-0.03569	-1.16597	0.244598	0.517534	chr12	2716844
cg08028452	0.045856	1.165688	0.244712	0.517623	chr3	53545444
cg18522549	0.082692	1.165191	0.244913	0.517807	chr11	62473861
cg18761756	0.044834	1.164528	0.245181	0.518086	chr18	3732002
cg08104845	0.055372	1.162607	0.245958	0.518865	chr1	182356337
cg25894071	0.074835	1.159098	0.247383	0.520272	chr4	101953935
cg08505222	0.044576	1.158751	0.247525	0.520367	chr22	51139277
cg02929073	-0.04862	-1.15667	0.248372	0.521238	chr7	126892249
cg11801011	0.046184	1.155418	0.248884	0.52174	chr19	51220537
cg26235243	0.03368	1.154308	0.249337	0.522161	chr6	33656836
cg00050938	0.046354	1.15243	0.250107	0.522894	chr3	50296100
cg21028562	0.027831	1.151417	0.250523	0.523351	chr3	50287909
cg02380983	0.042231	1.150821	0.250767	0.523611	chr19	48568071
cg05960039	0.025661	1.150256	0.250999	0.523846	chr20	57465123
cg05848509	-0.04886	-1.14985	0.251167	0.524027	chr17	72857354
cg10917153	0.027429	1.148623	0.251671	0.524553	chr15	42448786
cg14792155	0.035312	1.148322	0.251795	0.524687	chr15	42289618
cg26322763	0.036465	1.147677	0.252061	0.524955	chr19	48914444
cg10139742	0.047316	1.147257	0.252234	0.525153	chr16	56352151
cg25475999	0.057576	1.146764	0.252438	0.525362	chr11	35282046
cg20759084	-0.058	-1.14455	0.253352	0.52621	chr5	7395875
cg23374892	-0.10232	-1.14242	0.254236	0.527101	chr19	51165845
cg15279308	-0.04206	-1.14238	0.254251	0.527106	chr12	2800500
cg06185738	-0.04704	-1.14183	0.254481	0.527296	chr11	22359868
cg22860367	0.028899	1.141429	0.254646	0.527474	chr20	57426538
cg10083824	0.052432	1.140164	0.255172	0.528047	chr6	34102147
cg19892433	0.047094	1.139819	0.255315	0.528189	chr11	70331861
cg06646622	0.053943	1.139672	0.255376	0.528254	chr5	78766902
cg16774375	0.027816	1.139541	0.255431	0.528306	chr16	4102333
cg13533759	0.03524	1.136804	0.256572	0.529459	chr9	114431056
cg13065504	0.039938	1.136691	0.256619	0.529499	chr15	42448234
cg04251662	-0.04123	-1.13634	0.256764	0.529598	chr3	4535075
cg10206594	0.064641	1.13592	0.256941	0.529735	chr1	37314665
cg08931917	0.048657	1.135494	0.257119	0.529915	chr12	14109569
cg27529848	-0.04233	-1.13544	0.257142	0.529939	chr15	52472330
cg07482508	-0.0526	-1.13536	0.257176	0.529996	chr5	36606981
cg00986191	0.047669	1.134269	0.257632	0.530424	chr5	178419655
cg26601922	-0.04176	-1.13187	0.258635	0.531381	chr3	50243174
cg21635870	-0.07241	-1.13102	0.258996	0.531701	chr6	101847058

cg12372477	-0.04189	-1.13054	0.259196	0.531912	chr20	57465915
cg15680973	0.066605	1.129776	0.259517	0.532271	chr5	7420177
cg16040341	0.039417	1.128456	0.260073	0.532804	chr15	83544284
cg00671386	0.048372	1.127489	0.260481	0.533232	chr16	851834
cg23854103	-0.09734	-1.127	0.260687	0.533429	chr19	2543602
cg04498418	-0.05033	-1.12645	0.260921	0.533685	chr6	101850540
cg02232377	-0.05813	-1.12585	0.261172	0.533922	chr7	86297084
cg14957718	-0.08449	-1.12359	0.26213	0.534888	chr3	50243260
cg26454299	-0.04866	-1.12349	0.262172	0.534934	chr4	102268957
cg06849501	0.038208	1.123138	0.262321	0.535062	chr11	70458964
cg26764244	0.065782	1.121952	0.262824	0.535613	chr1	68299511
cg10324572	0.034785	1.121592	0.262977	0.535749	chr3	51749334
cg20490175	0.03216	1.12048	0.263449	0.536203	chr12	2797745
cg00720707	0.03941	1.120405	0.263481	0.536233	chr16	4016845
cg27277859	-0.06708	-1.11838	0.264343	0.537021	chr19	51165632
cg05765440	0.038709	1.116038	0.265343	0.538059	chr1	68252343
cg03104569	-0.05616	-1.11557	0.265544	0.538254	chr19	13615864
cg15219163	0.047277	1.115297	0.26566	0.538375	chr11	70842128
cg03100024	0.039832	1.113396	0.266473	0.539106	chr19	42571402
cg00350503	0.052066	1.112719	0.266764	0.539398	chr19	13613614
cg06147822	-0.06884	-1.11169	0.267207	0.539881	chr20	57466905
cg01246398	0.03373	1.110388	0.267765	0.54044	chr3	123165872
cg02500883	0.039169	1.108699	0.268491	0.541086	chr9	104356619
cg25983380	-0.09286	-1.10764	0.268949	0.541573	chr20	57465439
cg15218096	0.056926	1.107345	0.269075	0.541688	chr11	70858342
cg27299660	-0.04824	-1.10727	0.269108	0.541698	chr3	171527797
cg12312205	-0.03196	-1.10723	0.269124	0.541715	chr18	3594173
cg00943909	-0.05676	-1.1039	0.270562	0.543028	chr20	57427942
cg14731698	0.064932	1.103436	0.270765	0.543242	chr11	120738292
cg13353325	0.078965	1.103226	0.270856	0.543342	chr20	57485837
cg16890681	-0.04601	-1.10273	0.27107	0.543475	chr2	68479269
cg22134372	0.047474	1.101487	0.271611	0.543993	chr15	52455211
cg21462934	0.048496	1.100003	0.272256	0.544611	chr19	2622858
cg25751482	0.055824	1.099876	0.272311	0.544648	chr17	64301327
cg12008034	0.033762	1.098846	0.272759	0.545064	chr6	33996580
cg22088263	0.03802	1.096649	0.273717	0.545976	chr3	51746830
cg25210134	-0.04713	-1.09664	0.273722	0.545976	chr15	40600265
cg03043696	0.034766	1.09542	0.274254	0.54655	chr1	1820656
cg19217955	-0.03467	-1.09486	0.274499	0.546733	chr17	7123994
cg10011623	0.035538	1.094192	0.274792	0.547025	chr20	57463527
cg13740815	-0.05199	-1.09322	0.275217	0.547415	chr19	51170356
cg02892153	-0.04532	-1.09216	0.275684	0.547911	chr18	3593461
cg10347199	-0.07256	-1.09069	0.276329	0.548481	chr17	7123561
cg14337339	-0.04519	-1.09025	0.276523	0.548666	chr3	53529481
cg20484832	0.052134	1.08913	0.277013	0.549081	chr20	9075810
cg07851738	0.038894	1.086877	0.278007	0.550027	chr8	131896788
cg24000444	-0.06519	-1.08671	0.27808	0.550084	chr18	3771452
cg21673873	0.042913	1.08276	0.279827	0.551705	chr11	70511688

cg19251850	0.046146	1.082178	0.280085	0.551946	chr3	171428254
cg26129110	0.035499	1.077921	0.281978	0.553793	chr5	7619983
cg15109207	-0.07657	-1.07684	0.28246	0.554258	chr19	48614773
cg19244300	0.03262	1.075413	0.283096	0.554874	chr1	110113304
cg00732970	-0.02911	-1.07478	0.283379	0.555159	chr20	57414162
cg14791525	-0.09046	-1.07474	0.283398	0.555177	chr11	70732224
cg16850687	-0.05368	-1.07464	0.283442	0.55521	chr18	3594398
cg01157070	0.069293	1.074008	0.283725	0.555522	chr16	56228511
cg13416129	0.051395	1.071256	0.284958	0.556599	chr9	140037808
cg08698835	0.048516	1.071167	0.284998	0.55664	chr16	56279276
cg22076160	-0.0688	-1.07092	0.285107	0.556772	chr1	235805690
cg01748573	0.034131	1.070272	0.2854	0.557025	chr20	57463530
cg13054613	0.035855	1.069598	0.285702	0.557314	chr3	7742036
cg27552287	0.06588	1.066528	0.287084	0.558585	chr19	54385396
cg08644463	-0.04421	-1.06443	0.288033	0.559485	chr1	110106962
cg22484822	0.040815	1.063264	0.288559	0.560025	chr6	33996111
cg01090161	-0.03102	-1.06309	0.288637	0.560088	chr6	34032747
cg07713849	-0.02338	-1.06294	0.288708	0.560163	chr6	33624841
cg16986624	-0.06415	-1.06277	0.288782	0.560219	chr15	52471717
cg01865825	-0.04192	-1.06272	0.288808	0.560237	chr16	850240
cg11118235	-0.03137	-1.06104	0.289566	0.560932	chr3	50284010
cg14594362	-0.04792	-1.0601	0.289993	0.561301	chr19	48947631
cg26212328	0.077232	1.058416	0.290759	0.561963	chr12	46767665
cg11676382	0.044236	1.057837	0.291022	0.56222	chr22	51141829
cg06181697	0.036731	1.057581	0.291139	0.562333	chr3	50294603
cg10283969	0.02965	1.055876	0.291916	0.563042	chr11	70666639
cg17641631	-0.04567	-1.05564	0.292024	0.563108	chr3	4535021
cg19047292	-0.06497	-1.05504	0.292299	0.563346	chr16	56228442
cg06173536	-0.04338	-1.05405	0.292749	0.563804	chr1	235814462
cg09505516	-0.04468	-1.05306	0.293201	0.564255	chr2	25110296
cg05255330	0.031885	1.052095	0.293644	0.56463	chr18	3498963
cg22531801	-0.0642	-1.05153	0.293901	0.56491	chr1	235806070
cg15114328	-0.03708	-1.05121	0.294051	0.565078	chr1	182361557
cg14788049	-0.05007	-1.05053	0.29436	0.565333	chr11	64018549
cg08897759	0.058242	1.050124	0.294548	0.565504	chr5	178415944
cg12452300	0.020614	1.04973	0.294729	0.565657	chr3	51751788
cg10181414	0.069637	1.049296	0.294928	0.565826	chr19	2546598
cg16548911	-0.03994	-1.04868	0.295211	0.56607	chr16	50347766
cg03199239	0.076314	1.048625	0.295236	0.566094	chr19	2588553
cg02910037	0.030672	1.04853	0.29528	0.566128	chr16	4102366
cg06898306	0.057615	1.045101	0.296859	0.567657	chr6	33996673
cg10306450	-0.04385	-1.04338	0.297655	0.568394	chr12	56882479
cg26635576	0.043258	1.043283	0.297698	0.568433	chr11	35275997
cg12732284	0.071656	1.042909	0.297871	0.568601	chr3	171320284
cg27644733	0.042905	1.041498	0.298524	0.569201	chr16	9857216
cg11414276	0.028768	1.038061	0.300118	0.570645	chr12	2166831
cg10699496	-0.03659	-1.03439	0.301828	0.57227	chr3	123005576
cg21422400	0.038019	1.034223	0.301906	0.572346	chr1	1747243

cg20528838	-0.05245	-1.03181	0.303034	0.57333	chr20	57427730
cg15212295	-0.0336	-1.03171	0.30308	0.573361	chr17	64710687
cg06940168	0.05804	1.02747	0.305067	0.575236	chr17	64370665
cg14399447	-0.03288	-1.02555	0.305972	0.576005	chr19	48613950
cg07986199	0.038626	1.024398	0.306513	0.576503	chr12	2743038
cg09654471	0.041201	1.021549	0.307858	0.577538	chr3	4624132
cg25418001	0.065221	1.021421	0.307918	0.577603	chr7	79780310
cg17952046	0.041962	1.021174	0.308035	0.577707	chr1	37379413
cg07561162	-0.03579	-1.01923	0.308955	0.578476	chr16	10174417
cg21971807	-0.03642	-1.01836	0.309366	0.578838	chr20	57471654
cg04576491	0.037622	1.017724	0.309669	0.579115	chr19	1005427
cg06441398	0.035374	1.017552	0.309751	0.579213	chr11	70317455
cg12727358	0.051924	1.015452	0.310749	0.580208	chr3	53839610
cg20495738	0.040721	1.013811	0.31153	0.580964	chr12	2338399
cg13980113	-0.04242	-1.01178	0.3125	0.581846	chr5	36607333
cg14583825	-0.0369	-1.01096	0.312889	0.582152	chr19	54393040
cg24266105	-0.0695	-1.01023	0.31324	0.582516	chr6	146351044
cg00498360	0.031255	1.005944	0.315293	0.584422	chr17	64504304
cg14967731	0.027687	1.004656	0.315912	0.58499	chr3	4748984
cg11727252	0.024454	1.004373	0.316048	0.585084	chr3	51752143
cg17566735	-0.02948	-1.00376	0.316342	0.585335	chr3	171527257
cg27501686	-0.06086	-1.00255	0.316923	0.585895	chr12	2184874
cg11221524	0.039071	1.000969	0.317688	0.586613	chr1	84969091
cg08884490	0.026676	0.999591	0.318353	0.58725	chr22	51160651
cg23522194	-0.1341	-0.99813	0.319058	0.587856	chr19	48565189
cg11921270	-0.03998	-0.99803	0.31911	0.58791	chr19	19051154
cg24155399	0.04463	0.996622	0.31979	0.588564	chr1	235781578
cg27112585	0.036775	0.996171	0.320009	0.588787	chr11	70378117
cg05765011	0.034069	0.994741	0.320703	0.589301	chr16	4103225
cg08407014	0.050007	0.994051	0.321038	0.589596	chr19	2541104
cg13759674	-0.12113	-0.99307	0.321516	0.590029	chr9	140051205
cg27451362	0.082457	0.992995	0.321552	0.590059	chr6	101846650
cg27363558	-0.04222	-0.99274	0.321678	0.590141	chr17	7108792
cg14149552	-0.03664	-0.99218	0.321947	0.590304	chr9	80642464
cg01520586	-0.03451	-0.99126	0.322397	0.590604	chr3	50280404
cg08411235	0.025477	0.990584	0.322726	0.590907	chr11	35297026
cg24090202	0.042606	0.988828	0.323583	0.591717	chr19	54410030
cg06541349	0.027923	0.988316	0.323833	0.591962	chr16	4015096
cg02840199	0.039295	0.987672	0.324148	0.592241	chr16	4165257
cg02757172	0.068072	0.982689	0.326591	0.594405	chr4	158141076
cg01661235	-0.03014	-0.98167	0.327094	0.594869	chr16	23988974
cg18619398	0.020066	0.979424	0.328198	0.59576	chr20	57416506
cg19653589	-0.04103	-0.97707	0.329361	0.596794	chr19	2614177
cg09611472	0.071574	0.976668	0.329559	0.596974	chr16	850371
cg15067127	0.041312	0.974624	0.330571	0.597895	chr1	182352978
cg04367107	0.049269	0.971226	0.332256	0.599442	chr11	88238963
cg12102973	0.045527	0.970483	0.332626	0.59974	chr19	13320368
cg08860136	0.024749	0.968816	0.333456	0.600463	chr17	7111414

cg02760164	0.053583	0.966582	0.33457	0.60134	chr15	42371967
cg05366189	0.046137	0.965058	0.335331	0.601926	chr19	13397419
cg15815156	-0.05401	-0.96478	0.335469	0.602083	chr15	52472201
cg17402889	0.030795	0.964264	0.335728	0.602347	chr12	2527099
cg25456593	0.09099	0.962857	0.336433	0.602994	chr11	70672858
cg10533538	-0.04815	-0.96245	0.336636	0.603161	chr16	4166864
cg23542572	0.026267	0.962282	0.336721	0.603204	chr17	64780054
cg26320601	-0.0363	-0.95836	0.338691	0.605008	chr12	26986803
cg02754494	-0.03632	-0.95816	0.33879	0.605082	chr5	78810199
cg24882525	0.030418	0.957982	0.338881	0.605182	chr11	70565226
cg16850173	-0.06764	-0.95734	0.339203	0.60548	chr9	140033611
cg19577617	-0.03559	-0.95615	0.339806	0.605898	chr2	191745301
cg01509809	0.028714	0.955085	0.340341	0.606373	chr17	47284233
cg16833551	-0.05004	-0.95239	0.341705	0.607531	chr20	57427237
cg08619378	-0.02782	-0.95219	0.341806	0.607608	chr7	45616358
cg09284949	-0.06508	-0.95197	0.341916	0.607676	chr19	51190179
cg05607461	-0.03823	-0.94987	0.342981	0.608592	chr2	191745416
cg01700524	0.043704	0.947581	0.344143	0.609685	chr14	24803014
cg22901212	0.049439	0.943517	0.346213	0.611533	chr19	1003348
cg19999705	0.031807	0.942694	0.346633	0.611819	chr6	34026903
cg21142456	-0.0429	-0.9424	0.346782	0.611926	chr15	42376744
cg08001559	0.034165	0.940136	0.347941	0.612823	chr14	52326905
cg27272547	-0.03897	-0.93824	0.348915	0.613642	chr19	13347337
cg12535596	-0.03718	-0.93403	0.351077	0.615528	chr1	182360697
cg16829998	-0.04176	-0.93298	0.351617	0.616019	chr19	49944964
cg13371705	0.026495	0.932661	0.351782	0.616127	chr12	2452955
cg26345619	0.034226	0.931321	0.352473	0.616712	chr12	2602405
cg19700341	-0.05032	-0.92929	0.353522	0.617606	chr17	72857076
cg26343183	-0.03974	-0.9291	0.35362	0.617663	chr5	153008721
cg09576209	0.043812	0.928436	0.353964	0.617961	chr12	2339614
cg07064537	0.02448	0.92736	0.354522	0.618478	chr3	171515212
cg08578734	-0.03963	-0.92596	0.355247	0.619016	chr5	152870490
cg25562925	0.019508	0.925681	0.355392	0.619125	chr2	25110049
cg04202736	0.060947	0.923308	0.356625	0.620263	chr15	42371721
cg17867333	-0.05405	-0.92236	0.357117	0.620726	chr5	178423163
cg14011070	-0.05974	-0.92054	0.358066	0.621523	chr1	235811811
cg03716942	-0.05295	-0.92024	0.358222	0.621637	chr1	1821981
cg20408693	-0.03573	-0.91917	0.358781	0.622102	chr12	46767289
cg25869295	0.031115	0.914733	0.361103	0.624102	chr11	70433086
cg19206040	-0.0698	-0.91454	0.361206	0.624153	chr1	37500441
cg18574254	0.067664	0.914131	0.361418	0.624365	chr7	126889015
cg20518994	0.032865	0.912526	0.36226	0.62503	chr2	25141532
cg00348762	-0.04611	-0.91208	0.362495	0.62524	chr16	56228114
cg05511872	-0.06189	-0.91148	0.362808	0.625491	chr11	70824260
cg22798121	0.031328	0.911224	0.362945	0.62563	chr5	152896564
cg01130792	-0.05159	-0.91045	0.363351	0.626005	chr22	51112683
cg11335969	0.030465	0.910095	0.363539	0.62617	chr22	22118304
cg27271486	-0.06407	-0.90995	0.363614	0.626241	chr16	56227690

cg15372689	-0.04456	-0.90886	0.364191	0.626729	chr7	93550986
cg14638988	0.042669	0.908727	0.364259	0.626768	chr11	62476544
cg22363670	-0.03599	-0.90833	0.364466	0.626938	chr7	86273169
cg21779904	0.024764	0.908015	0.364635	0.627085	chr20	57425157
cg20019489	0.048913	0.907438	0.364939	0.62733	chr20	57414351
cg12281620	0.040037	0.905581	0.36592	0.628115	chr19	2525290
cg05456713	0.033862	0.903738	0.366895	0.628888	chr19	2513356
cg01397507	-0.03575	-0.90183	0.367906	0.629781	chr11	70440395
cg20306837	-0.04374	-0.90103	0.368329	0.630135	chr11	105481988
cg00377653	-0.05751	-0.90052	0.368602	0.630322	chr12	56882535
cg08572336	-0.09883	-0.90022	0.36876	0.630475	chr19	51165404
cg16448399	0.045096	0.899167	0.36932	0.631	chr18	3880076
cg07959068	0.025314	0.899054	0.36938	0.631032	chr16	24057138
cg14116756	0.077615	0.897571	0.37017	0.631678	chr10	75255721
cg25733708	-0.0362	-0.89618	0.370908	0.63229	chr19	54393220
cg14241370	-0.05324	-0.89527	0.371396	0.632626	chr9	104499850
cg03731464	0.030457	0.894589	0.371759	0.632924	chr12	2801158
cg04742605	0.025852	0.892088	0.373096	0.634013	chr11	70385511
cg10487659	0.033469	0.891094	0.373628	0.634465	chr16	4152081
cg19542445	0.028972	0.891033	0.373661	0.634487	chr12	2596220
cg15389472	-0.0464	-0.88858	0.374974	0.635466	chr1	182361528
cg17155859	0.026598	0.886142	0.376286	0.636567	chr11	64026541
cg03321319	0.044397	0.885847	0.376444	0.636688	chr22	22222323
cg07114886	0.021924	0.884318	0.377267	0.637315	chr3	51745946
cg04190002	0.02776	0.883547	0.377683	0.637685	chr22	51113604
cg17024257	-0.02852	-0.8832	0.377872	0.63785	chr3	171528758
cg18589960	-0.04024	-0.88269	0.378146	0.638047	chr19	51220392
cg15418783	0.031012	0.880973	0.379072	0.638825	chr11	70557693
cg17260383	-0.02484	-0.88076	0.37919	0.638911	chr8	22298246
cg07268119	0.026728	0.878191	0.380577	0.640074	chr7	45717573
cg06137273	0.064377	0.877396	0.381008	0.640402	chr3	6905031
cg24904943	0.047753	0.87734	0.381039	0.640415	chr5	7781077
cg04518808	-0.04187	-0.87688	0.381285	0.640615	chr19	54384822
cg02540833	0.040343	0.875525	0.382023	0.641123	chr11	70562363
cg07601741	-0.11069	-0.87508	0.382266	0.641307	chr5	153160425
cg02274788	0.031168	0.873658	0.383037	0.641887	chr1	68232457
cg13592780	-0.05087	-0.87364	0.383047	0.641889	chr3	123010034
cg24671939	-0.0338	-0.87325	0.38326	0.642058	chr18	3593798
cg04378167	-0.05347	-0.87255	0.383639	0.642345	chr19	49944943
cg25592107	-0.04537	-0.87224	0.383809	0.642526	chr19	19042864
cg21650436	0.02013	0.871697	0.384105	0.642736	chr12	49162444
cg25196508	0.035955	0.871051	0.384457	0.643047	chr12	49181554
cg07947033	-0.04518	-0.8709	0.384539	0.643121	chr20	57426545
cg16098545	-0.03786	-0.87027	0.384883	0.643378	chr12	26492312
cg01450274	0.057376	0.870224	0.384908	0.643404	chr3	171396325
cg08066673	0.026081	0.869378	0.38537	0.643753	chr14	52325747
cg09787442	-0.03626	-0.86934	0.385388	0.64377	chr21	31119294
cg04005969	-0.03374	-0.86914	0.3855	0.643846	chr19	51171247

cg15877314	0.034895	0.868753	0.385711	0.644026	chr3	50273895
cg03014008	0.025699	0.867375	0.386464	0.644594	chr20	57463767
cg20998200	0.035856	0.866878	0.386736	0.644791	chr11	22370099
cg07559526	0.031141	0.866818	0.386768	0.644814	chr16	4164735
cg07960450	-0.05531	-0.86677	0.386794	0.644834	chr7	45614300
cg10336707	0.061037	0.866275	0.387065	0.644993	chr11	62476526
cg11435826	-0.02455	-0.86541	0.38754	0.645347	chr12	2792111
cg14851700	0.051894	0.864751	0.3879	0.645685	chr1	182362230
cg24037166	0.042938	0.863909	0.388362	0.645973	chr10	75255724
cg08515427	0.027665	0.860991	0.389964	0.64728	chr16	848997
cg26983544	0.038704	0.860149	0.390427	0.647618	chr5	78784024
cg06192619	0.031076	0.859817	0.39061	0.647772	chr19	15083616
cg02557189	-0.03032	-0.8595	0.390785	0.647904	chr7	100273384
cg22562461	0.04367	0.85949	0.39079	0.64791	chr7	86274769
cg14350337	0.042089	0.856152	0.39263	0.649435	chr9	140060064
cg14815005	0.087756	0.855471	0.393006	0.649735	chr22	22222162
cg23427362	0.040069	0.851343	0.395291	0.651642	chr12	2335272
cg03058660	-0.06618	-0.85127	0.395332	0.651683	chr19	48919198
cg25121007	0.033386	0.850396	0.395817	0.652059	chr7	126854672
cg00269140	0.060412	0.850328	0.395854	0.652094	chr7	86389542
cg24946911	0.046363	0.850314	0.395862	0.6521	chr12	2788654
cg02879453	-0.03293	-0.85005	0.396007	0.652219	chr16	50321818
cg20170028	-0.05574	-0.8498	0.396147	0.652342	chr11	70917283
cg24218925	-0.02634	-0.84919	0.396486	0.652616	chr19	2578938
cg09150064	-0.05019	-0.84537	0.39861	0.654301	chr11	22364185
cg06489744	0.036214	0.844625	0.399026	0.65464	chr11	70557772
cg05902503	0.025987	0.842563	0.400177	0.655597	chr16	30133175
cg02525785	-0.03372	-0.84255	0.400184	0.655597	chr17	7117684
cg08033640	0.027526	0.842169	0.400397	0.655747	chr11	70419197
cg27184649	0.027135	0.838311	0.402556	0.657304	chr11	70935963
cg17752088	-0.04411	-0.8373	0.403121	0.657739	chr5	78810367
cg03330642	0.018729	0.836851	0.403375	0.657873	chr5	178411124
cg10797197	0.039627	0.83629	0.40369	0.65808	chr20	57444000
cg15350840	0.042915	0.835014	0.404407	0.658602	chr11	70475709
cg23281712	0.040942	0.834237	0.404844	0.658986	chr2	25143391
cg21114126	-0.0521	-0.83422	0.404853	0.658998	chr4	102267189
cg22986870	0.027378	0.832447	0.405851	0.659886	chr7	126765549
cg01638185	0.038527	0.832176	0.406004	0.660002	chr17	64530027
cg23698058	0.043405	0.831474	0.406399	0.660298	chr1	84544097
cg10331829	0.02996	0.831443	0.406417	0.660322	chr11	35343789
cg00792185	0.031998	0.830261	0.407084	0.660807	chr16	9855960
cg04603130	-0.03248	-0.83011	0.40717	0.660837	chr19	2550027
cg18846074	0.059341	0.829182	0.407693	0.661229	chr14	24801073
cg09437522	-0.03613	-0.82777	0.408493	0.661859	chr20	57431202
cg16143105	0.057285	0.82697	0.408944	0.662216	chr5	78689278
cg04398695	-0.06199	-0.8267	0.409094	0.662343	chr16	850713
cg01386883	-0.02687	-0.82636	0.409289	0.66245	chr3	53529144
cg11116429	-0.04017	-0.82577	0.409624	0.662701	chr5	36607417

cg04903916	0.025519	0.82463	0.410269	0.663272	chr6	33638413
cg27107076	0.054089	0.822451	0.411506	0.664244	chr2	25050844
cg20288565	-0.06474	-0.82244	0.411514	0.664244	chr1	235805403
cg04835297	-0.04691	-0.82196	0.411783	0.664431	chr3	142443257
cg08204867	-0.04271	-0.82194	0.411798	0.664446	chr16	10208426
cg20979061	-0.03784	-0.82168	0.411944	0.664556	chr19	49939949
cg03871526	0.058844	0.81956	0.413149	0.665512	chr3	142447949
cg26495109	-0.03144	-0.81888	0.413534	0.665815	chr16	56225952
cg10639428	0.020229	0.817207	0.414491	0.666559	chr2	25138879
cg24432193	0.047966	0.816834	0.414703	0.666696	chr6	102062733
cg04085699	0.024366	0.816336	0.414987	0.666911	chr11	64026024
cg05339056	0.042428	0.815855	0.415262	0.667128	chr7	86391162
cg14150378	0.034314	0.815836	0.415273	0.667136	chr9	80335274
cg19589727	-0.03768	-0.81526	0.415603	0.667406	chr20	57427762
cg24194077	-0.02798	-0.8139	0.416382	0.667938	chr19	54386020
cg00116766	0.036777	0.812679	0.417078	0.668441	chr16	9857614
cg06490627	-0.04151	-0.81254	0.417156	0.668484	chr16	50344198
cg27351813	-0.03214	-0.81221	0.417348	0.668641	chr19	51197056
cg15160746	0.033697	0.810606	0.418266	0.669318	chr12	2743239
cg14351882	0.052875	0.810568	0.418288	0.669334	chr9	140061878
cg08969344	0.029271	0.80994	0.418648	0.669627	chr6	34031597
cg10801143	0.028502	0.809387	0.418966	0.669831	chr11	88245465
cg03011594	-0.04304	-0.80939	0.418966	0.669831	chr16	56370697
cg10371523	0.022063	0.80697	0.420354	0.670879	chr11	70395513
cg16218964	0.058992	0.804652	0.421689	0.671971	chr11	62473680
cg01010868	-0.07221	-0.80245	0.422959	0.673062	chr19	14228654
cg19151292	0.021951	0.801266	0.423643	0.673611	chr6	33653502
cg12128893	0.021767	0.800957	0.423822	0.673761	chr10	75255807
cg18618964	0.030076	0.79943	0.424705	0.674477	chr2	68480222
cg23460210	-0.04053	-0.79841	0.425294	0.674912	chr19	54385404
cg26010751	0.024656	0.798282	0.425369	0.674985	chr3	51742513
cg21130255	0.029695	0.798142	0.425451	0.675057	chr11	70368676
cg25144574	0.022687	0.797827	0.425633	0.675192	chr5	36655019
cg08364956	0.035462	0.797708	0.425702	0.675241	chr5	178407122
cg26826325	0.023869	0.797574	0.42578	0.675303	chr11	70459053
cg01014438	0.045383	0.795592	0.426929	0.676168	chr12	2762689
cg06200857	-0.05293	-0.79515	0.427184	0.676314	chr20	57426420
cg13651483	0.043425	0.795089	0.427221	0.676343	chr19	48566587
cg03010274	-0.02555	-0.79439	0.427629	0.6766	chr20	57427274
cg26389955	0.02856	0.793623	0.428073	0.676931	chr17	64640562
cg04404381	0.031879	0.792889	0.4285	0.677228	chr11	70563580
cg21599324	-0.01833	-0.7924	0.428782	0.677415	chr6	34074282
cg26674800	0.031508	0.792325	0.428828	0.677462	chr17	7108653
cg23913904	0.03657	0.789833	0.43028	0.678455	chr11	70590130
cg13139998	0.019115	0.789785	0.430308	0.678477	chr3	51740201
cg06015218	-0.03444	-0.78929	0.430595	0.678712	chr6	146350434
cg16730369	-0.0293	-0.78906	0.430732	0.678813	chr11	120823575
cg06739873	0.028834	0.788644	0.430973	0.678999	chr19	19040289

cg10692302	-0.04988	-0.7874	0.431697	0.679588	chr3	51747227
cg03478199	-0.04098	-0.7873	0.431756	0.679626	chr6	146348913
cg17755730	0.024614	0.783953	0.433717	0.681136	chr3	50285392
cg13844463	0.033356	0.78231	0.43468	0.681809	chr11	35436672
cg07212894	-0.03073	-0.78214	0.434779	0.681877	chr3	50243021
cg03882437	-0.03968	-0.78184	0.434956	0.682015	chr9	140051729
cg26452915	-0.02444	-0.78077	0.435584	0.682465	chr20	57486076
cg10887945	0.028033	0.780454	0.435769	0.682609	chr3	4805396
cg09405076	0.022238	0.77944	0.436365	0.683091	chr11	70477139
cg23077606	0.023348	0.778821	0.436729	0.683337	chr11	70540206
cg12996903	-0.02595	-0.77798	0.437226	0.68371	chr3	50275575
cg19640589	-0.04812	-0.77764	0.437423	0.683859	chr20	57427973
cg10144604	-0.03396	-0.77669	0.437986	0.684221	chr20	57465599
cg19325477	0.038776	0.775945	0.438423	0.684523	chr17	64688496
cg16862319	-0.04064	-0.77522	0.438847	0.684853	chr12	13899195
cg11357538	0.01668	0.774265	0.439414	0.685318	chr20	57463397
cg22242216	-0.04861	-0.77425	0.439422	0.685323	chr19	48947578
cg06952422	0.025272	0.773598	0.439808	0.685638	chr6	33647685
cg22956310	0.040459	0.772126	0.440678	0.6864	chr2	25142878
cg08123425	-0.03865	-0.77187	0.440827	0.686517	chr12	13939517
cg17540499	0.047938	0.771429	0.44109	0.686679	chr7	126698451
cg11317158	0.036494	0.771028	0.441327	0.686849	chr19	49938204
cg18166990	0.024652	0.770514	0.441631	0.687081	chr11	70515565
cg13329789	0.018914	0.769689	0.44212	0.687406	chr7	45757644
cg24867458	-0.0289	-0.76954	0.44221	0.687451	chr1	110090677
cg14414124	0.027831	0.767467	0.443437	0.688425	chr2	68465373
cg25673737	0.03473	0.766915	0.443765	0.688695	chr4	101966414
cg00951869	-0.01723	-0.76657	0.443971	0.688864	chr14	24805349
cg10468484	0.019365	0.766549	0.443982	0.688872	chr20	57431303
cg18162783	-0.02665	-0.76631	0.444123	0.688981	chr1	1795891
cg16475558	0.046762	0.765376	0.444679	0.689384	chr16	56388945
cg19270265	0.049124	0.765096	0.444845	0.689459	chr7	126275080
cg16446012	0.029592	0.764998	0.444903	0.689509	chr5	78772259
cg00318899	-0.02476	-0.76459	0.445144	0.689686	chr3	50284137
cg12536809	0.025079	0.762325	0.446495	0.690733	chr17	72852514
cg00991400	-0.0315	-0.76197	0.446707	0.690885	chr7	126883137
cg25976932	0.023157	0.761315	0.447096	0.691163	chr3	123138958
cg23753795	0.054581	0.760955	0.447311	0.691288	chr6	146531540
cg12771777	0.046321	0.758429	0.448819	0.692446	chr11	70692038
cg04509024	0.023825	0.757512	0.449367	0.692906	chr1	37270540
cg11856810	-0.04357	-0.75705	0.449643	0.693118	chr2	155554961
cg12363682	0.036603	0.756825	0.449778	0.693224	chr5	178420690
cg17334845	0.015921	0.755019	0.450859	0.694004	chr20	57463572
cg26060003	0.028097	0.754572	0.451127	0.694176	chr1	53558511
cg03182218	0.033051	0.754239	0.451326	0.694356	chr17	7100221
cg01637551	-0.04602	-0.7537	0.451652	0.694624	chr12	2161661
cg00417823	-0.02332	-0.75093	0.453315	0.695972	chr16	56330268
cg06163629	0.019346	0.750225	0.453736	0.696258	chr20	57414884

cg24183324	-0.02979	-0.75014	0.453787	0.696304	chr11	35440062
cg03606258	0.123161	0.750063	0.453833	0.696329	chr20	57426935
cg16693012	0.023066	0.750057	0.453837	0.696329	chr1	68283821
cg09868882	0.023278	0.748647	0.454685	0.697023	chr7	126883640
cg11841246	0.04154	0.745024	0.456869	0.698777	chr15	52414751
cg14298577	0.025181	0.741211	0.459173	0.700542	chr7	100272703
cg09710790	0.035739	0.740027	0.459891	0.701094	chr3	4534905
cg13641156	-0.03482	-0.73883	0.460617	0.701634	chr11	70515650
cg12201698	0.022816	0.738457	0.460842	0.701789	chr11	70634975
cg08834938	0.018546	0.735962	0.462357	0.702929	chr16	4136053
cg00631706	0.025828	0.735171	0.462838	0.70325	chr5	78751239
cg06979118	-0.02449	-0.73275	0.464313	0.704361	chr11	70601971
cg25367568	-0.04453	-0.72952	0.46628	0.705852	chr20	57428437
cg18457944	-0.0287	-0.72751	0.46751	0.706704	chr3	171428279
cg21156276	-0.03854	-0.72671	0.467998	0.707131	chr9	4491917
cg09635994	0.033012	0.721575	0.471145	0.709235	chr22	51171263
cg04476846	0.025938	0.720702	0.471682	0.709617	chr5	78791415
cg03162045	0.027748	0.720614	0.471735	0.709648	chr3	142443933
cg16106068	0.019373	0.720337	0.471906	0.709775	chr14	24792081
cg15620385	0.034356	0.72033	0.47191	0.709777	chr12	26986279
cg00925020	-0.03351	-0.71945	0.472449	0.710182	chr19	2543877
cg18080819	0.028516	0.718382	0.473109	0.710637	chr11	70505972
cg16909293	0.027417	0.718251	0.473189	0.710695	chr1	68171537
cg14694901	0.041969	0.71764	0.473565	0.710987	chr10	75255186
cg26201811	0.058359	0.717411	0.473706	0.711063	chr22	51111714
cg25381331	0.040508	0.716995	0.473962	0.711242	chr1	53556414
cg08486432	0.022041	0.716612	0.474198	0.711426	chr6	33598003
cg08712808	0.041204	0.716269	0.474409	0.711563	chr3	7342929
cg19128261	0.01982	0.716179	0.474465	0.711607	chr3	53839251
cg10276272	0.027813	0.714848	0.475286	0.712167	chr16	10271822
cg10613332	0.031464	0.713231	0.476284	0.71296	chr19	13368845
cg15765694	-0.02223	-0.71275	0.476584	0.713147	chr9	104358101
cg26332715	-0.06585	-0.71126	0.477505	0.713839	chr2	191745502
cg09179079	-0.04432	-0.71106	0.477628	0.713947	chr6	146348690
cg13078421	-0.02669	-0.71068	0.477863	0.714125	chr19	2624622
cg23315601	0.027493	0.707473	0.479849	0.715677	chr11	120590134
cg18287522	0.038041	0.707407	0.47989	0.715701	chr3	179115547
cg10161743	0.029412	0.706351	0.480545	0.71616	chr19	48917816
cg09950871	0.022353	0.704358	0.481783	0.717121	chr16	4029235
cg11010575	0.024862	0.703387	0.482387	0.717491	chr15	42129464
cg11539664	0.032356	0.703207	0.482499	0.717532	chr6	33604664
cg27642181	0.025373	0.702013	0.483242	0.71802	chr20	57413694
cg05626242	0.027753	0.701927	0.483295	0.71806	chr10	75255789
cg17961101	-0.03989	-0.70082	0.483988	0.71857	chr22	22222050
cg11814875	-0.035	-0.69929	0.484941	0.719233	chr19	2611237
cg02902102	0.033338	0.698481	0.485444	0.719589	chr19	48902290
cg24030173	-0.01642	-0.69794	0.485785	0.71982	chr19	48922140
cg11422541	0.038885	0.69756	0.486019	0.719995	chr12	49182586

cg18117347	-0.02807	-0.69746	0.486079	0.720037	chr19	51196754
cg01944370	0.019894	0.696969	0.486388	0.720239	chr3	50286969
cg01937808	-0.02578	-0.69663	0.486598	0.720383	chr16	4025588
cg01174786	0.020244	0.696155	0.486897	0.720601	chr16	4027541
cg01017090	-0.01912	-0.69605	0.486965	0.720654	chr20	57427046
cg18160880	0.022147	0.695724	0.487167	0.720813	chr20	57463903
cg07526227	0.028516	0.694616	0.48786	0.721297	chr19	51168305
cg07121340	0.024839	0.693555	0.488524	0.721783	chr12	2173987
cg10998242	0.023579	0.692232	0.489353	0.722306	chr3	53530847
cg08505076	-0.02568	-0.69132	0.489928	0.722702	chr17	7122063
cg12760563	-0.03069	-0.69006	0.490716	0.723209	chr18	3594396
cg05329317	-0.0194	-0.69003	0.490736	0.723226	chr16	30126595
cg04733951	-0.02431	-0.68806	0.491974	0.724171	chr17	64545218
cg15522719	-0.03238	-0.68648	0.492964	0.724862	chr2	191745248
cg25316569	0.036532	0.685648	0.493491	0.725273	chr11	120530952
cg11659796	0.021099	0.684601	0.49415	0.725765	chr19	2560038
cg07547788	0.023063	0.683228	0.495016	0.726363	chr7	93534693
cg21250721	-0.0525	-0.68241	0.495531	0.726717	chr9	140056227
cg18414579	0.025744	0.682212	0.495657	0.726812	chr4	102266325
cg12650926	-0.02921	-0.68177	0.495934	0.727037	chr6	33589118
cg14116052	-0.03108	-0.68124	0.496273	0.727299	chr5	7596454
cg05921947	0.02972	0.681198	0.496298	0.727319	chr5	7715579
cg27176392	0.028702	0.680976	0.496438	0.727438	chr19	2652259
cg02607130	0.029706	0.680897	0.496487	0.727468	chr19	1008643
cg16379885	-0.02059	-0.67941	0.497426	0.728076	chr1	37500369
cg01104717	-0.03084	-0.67829	0.498138	0.728553	chr3	53545550
cg22834542	0.031376	0.677971	0.498338	0.728658	chr11	88288530
cg06930757	-0.02211	-0.67787	0.498402	0.728694	chr19	51216389
cg25804443	0.037919	0.67549	0.49991	0.729761	chr18	3875823
cg02433656	0.025838	0.675402	0.499966	0.729804	chr16	56322654
cg16920001	0.027636	0.674468	0.500558	0.730281	chr15	42448760
cg27586581	-0.02989	-0.67424	0.500706	0.730363	chr19	19051157
cg00800141	-0.03183	-0.67422	0.500717	0.730367	chr16	4014161
cg26199552	0.020467	0.672349	0.501905	0.731199	chr11	70368372
cg21340148	-0.02698	-0.67062	0.503003	0.731959	chr19	2702986
cg03654504	0.027031	0.669607	0.503649	0.732444	chr1	37495105
cg01728682	0.038901	0.669439	0.503756	0.732511	chr2	25057480
cg24825722	0.018951	0.667836	0.504777	0.73311	chr17	7121848
cg13939602	0.031144	0.667103	0.505244	0.733389	chr12	2467410
cg01823958	0.037681	0.66668	0.505514	0.733575	chr1	53557455
cg01419479	-0.04124	-0.66631	0.505751	0.733685	chr1	182360822
cg21375506	0.026234	0.666028	0.50593	0.733777	chr17	64470354
cg02637414	0.02002	0.665602	0.506202	0.733927	chr19	13613091
cg15122716	0.024658	0.665417	0.50632	0.734017	chr9	140050568
cg19015708	-0.02759	-0.66519	0.506467	0.734118	chr2	68418292
cg04994975	0.039784	0.663915	0.507279	0.734716	chr1	1795945
cg04279973	-0.02228	-0.66289	0.507937	0.735221	chr16	23846968
cg23686556	0.052515	0.66249	0.508191	0.735386	chr8	131961316

cg27019717	-0.02276	-0.66221	0.508367	0.735527	chr14	52351055
cg27304369	-0.03809	-0.66153	0.508802	0.735891	chr20	57427483
cg27279809	-0.03385	-0.66149	0.508834	0.735901	chr12	49183212
cg26496204	-0.01901	-0.66146	0.508853	0.73591	chr20	57427210
cg20910008	-0.02151	-0.66077	0.509291	0.736209	chr8	22301378
cg19558628	-0.02483	-0.65956	0.510069	0.736772	chr14	24801616
cg07841877	-0.02275	-0.65939	0.510179	0.736825	chr1	182359858
cg17641710	0.014738	0.657831	0.511176	0.737476	chr3	50279038
cg20716703	0.023633	0.656735	0.51188	0.737869	chr19	13397750
cg18854735	-0.03006	-0.65633	0.51214	0.738067	chr1	1822972
cg21793437	0.022259	0.653266	0.51411	0.739442	chr12	2734591
cg26999577	0.025822	0.651272	0.515394	0.740199	chr12	2340831
cg23264278	0.035984	0.649392	0.516606	0.740939	chr19	19051482
cg17038626	0.030844	0.648652	0.517084	0.741298	chr12	2435931
cg20740024	0.024503	0.644909	0.519503	0.742948	chr11	35303612
cg17867243	0.0494	0.644835	0.519551	0.74298	chr15	42371653
cg23524436	-0.01856	-0.64449	0.519775	0.743136	chr12	2161437
cg24603152	0.051432	0.64166	0.521608	0.744429	chr1	84543539
cg19742341	-0.02427	-0.64123	0.521889	0.744661	chr11	70385301
cg17500055	-0.03714	-0.64117	0.521926	0.744675	chr1	235805560
cg17483139	-0.02566	-0.63985	0.522782	0.74532	chr1	1822401
cg19356346	0.027416	0.637844	0.524086	0.746274	chr12	100749441
cg09169283	-0.02628	-0.63466	0.52616	0.747578	chr17	72856452
cg04142017	-0.0463	-0.63335	0.527013	0.748119	chr1	53558596
cg22188571	0.021274	0.632096	0.527829	0.748656	chr6	34074955
cg09001143	0.025954	0.631144	0.528451	0.749138	chr16	9857475
cg03641740	0.042051	0.631061	0.528505	0.749168	chr4	102087758
cg13729891	-0.02298	-0.63012	0.529117	0.749579	chr17	7108468
cg25766748	-0.03645	-0.63003	0.529179	0.749621	chr19	13614882
cg00380835	0.04537	0.628968	0.529872	0.750071	chr19	51165752
cg12321149	-0.03107	-0.62895	0.529884	0.750071	chr20	57427426
cg07748847	-0.03561	-0.62782	0.530624	0.750578	chr12	14124857
cg08987995	0.023099	0.626019	0.531802	0.751341	chr3	4788106
cg06456864	0.031237	0.625725	0.531994	0.751476	chr6	34101399
cg21988465	0.018658	0.625324	0.532257	0.751655	chr20	57429277
cg10961484	-0.02398	-0.62441	0.532857	0.752078	chr11	120530860
cg04103918	0.020477	0.624308	0.532923	0.752125	chr17	4726687
cg10126715	-0.01582	-0.62287	0.533868	0.752802	chr1	37500195
cg16875863	-0.04337	-0.62143	0.534814	0.753395	chr19	48947572
cg24203465	-0.01564	-0.62081	0.535221	0.753635	chr20	57425986
cg10031793	0.018904	0.620585	0.535367	0.753753	chr12	2734257
cg24791862	-0.01954	-0.62031	0.535546	0.753842	chr7	126893237
cg00014104	0.023532	0.620088	0.535694	0.753974	chr22	22220367
cg26560871	-0.03012	-0.619	0.536407	0.754429	chr6	146348616
cg15844381	-0.04351	-0.61872	0.536593	0.754569	chr19	14228577
cg19572362	-0.01748	-0.61868	0.536621	0.75458	chr1	110091012
cg19466160	-0.03116	-0.61803	0.537045	0.754861	chr17	7117160
cg24794531	-0.02281	-0.61689	0.537797	0.755332	chr3	142443837

cg24384034	0.024438	0.615881	0.538463	0.755789	chr11	70858463
cg23638849	0.024339	0.613413	0.540091	0.756916	chr11	70477184
cg09262171	-0.02205	-0.61339	0.540107	0.756924	chr16	4140361
cg08193910	-0.02185	-0.61295	0.540396	0.757174	chr19	54410103
cg27083019	-0.03379	-0.61061	0.541941	0.758227	chr19	49945958
cg19296354	0.017809	0.609171	0.542895	0.758875	chr20	57415697
cg07028661	-0.02929	-0.60783	0.543784	0.759433	chr21	31312905
cg00536080	0.030649	0.607138	0.544241	0.759721	chr19	13409931
cg09447435	0.02257	0.606299	0.544797	0.760108	chr6	101854480
cg00791406	0.028212	0.605887	0.54507	0.760306	chr12	14118880
cg17414107	-0.03669	-0.60539	0.545402	0.760502	chr20	57427830
cg14395444	-0.02952	-0.6044	0.546057	0.7609	chr12	2800182
cg18689402	0.104745	0.603878	0.546403	0.761133	chr3	4630986
cg02771299	0.01898	0.60352	0.546641	0.761337	chr6	33609240
cg10901633	-0.01531	-0.60344	0.546696	0.761386	chr3	4814459
cg12216470	0.038159	0.601325	0.5481	0.762297	chr19	51200481
cg00056257	0.035227	0.600341	0.548754	0.762772	chr3	6901652
cg27552955	-0.04182	-0.59828	0.550126	0.763697	chr3	142442915
cg04865531	0.021001	0.597621	0.550566	0.763969	chr22	51159147
cg00610508	-0.03656	-0.59737	0.550732	0.764079	chr16	4013337
cg24678505	-0.03547	-0.59449	0.552655	0.765378	chr12	56882429
cg16200584	-0.02123	-0.59422	0.552833	0.765528	chr11	62473981
cg14090219	0.021093	0.593163	0.553541	0.765998	chr19	13347085
cg23666299	0.032516	0.59314	0.553556	0.765999	chr6	102326919
cg19512268	-0.03051	-0.59309	0.553593	0.766025	chr6	146348901
cg07067241	-0.02846	-0.59244	0.554023	0.766293	chr3	142442711
cg05745748	0.0277	0.591718	0.554507	0.766526	chr6	33996013
cg01708648	0.018082	0.591548	0.554621	0.766609	chr11	64023044
cg16281600	0.025035	0.59114	0.554893	0.766807	chr5	152869431
cg08805338	-0.02314	-0.59082	0.555107	0.766903	chr10	75255936
cg23054925	-0.02936	-0.59032	0.555441	0.767109	chr1	84972704
cg03490157	0.040964	0.590302	0.555454	0.767114	chr3	6906061
cg03389717	-0.0488	-0.59026	0.555485	0.767123	chr6	33601269
cg01273580	-0.03792	-0.58824	0.556833	0.768077	chr19	49946108
cg05120716	0.015474	0.588197	0.556864	0.768101	chr16	23881993
cg17191518	0.023274	0.586927	0.557716	0.768644	chr15	52468850
cg14232870	0.03539	0.584931	0.559055	0.769466	chr11	70458782
cg12211091	0.027649	0.582355	0.560786	0.770599	chr19	2512999
cg18801906	0.025268	0.580895	0.561769	0.771308	chr11	70584165
cg07790747	-0.02326	-0.57817	0.563602	0.77243	chr16	56266223
cg27262796	-0.01581	-0.57734	0.564163	0.772783	chr20	57426858
cg17969540	-0.03893	-0.57636	0.564823	0.773246	chr19	48908179
cg14910395	0.0136	0.576225	0.564916	0.773308	chr19	48941986
cg22364668	0.041612	0.57584	0.565177	0.773476	chr19	49944826
cg27340723	0.035574	0.574307	0.566212	0.774158	chr16	4061608
cg14574037	-0.02341	-0.5733	0.566894	0.774645	chr2	155553986
cg04798490	0.020217	0.570233	0.568967	0.776008	chr11	70517045
cg11244758	0.013195	0.567418	0.570875	0.77723	chr20	57463900

cg06869158	0.013025	0.566764	0.571319	0.777511	chr19	48900058
cg05119467	0.018304	0.565899	0.571906	0.777879	chr1	1765440
cg11480267	0.033334	0.565458	0.572206	0.77811	chr20	57463503
cg26362197	0.041577	0.564433	0.572902	0.7785	chr19	48948005
cg20698170	-0.02716	-0.5641	0.573127	0.778638	chr15	42120362
cg17696044	0.026177	0.562806	0.574008	0.779259	chr11	70449316
cg16204066	0.030034	0.56044	0.575618	0.780314	chr6	33638922
cg23868250	0.024355	0.559965	0.575942	0.780528	chr11	120764552
cg03837903	0.035611	0.559344	0.576365	0.780821	chr20	57464000
cg17069533	0.013406	0.558223	0.577129	0.781332	chr17	64657833
cg14564778	-0.03026	-0.55729	0.577764	0.781798	chr20	57427556
cg13756965	-0.03772	-0.5558	0.578782	0.782419	chr11	70917458
cg07774177	0.023968	0.554032	0.57999	0.783205	chr15	42387287
cg18151275	0.022734	0.551405	0.581787	0.784263	chr12	56873616
cg17868128	0.024702	0.550322	0.582528	0.784718	chr17	64383859
cg03989758	0.029103	0.549585	0.583033	0.785023	chr5	36662950
cg16567056	-0.01875	-0.5474	0.584532	0.785933	chr15	40599985
cg04153604	-0.01413	-0.54628	0.5853	0.786411	chr17	7099823
cg20326682	0.014364	0.546246	0.585323	0.786426	chr17	64345488
cg04077795	-0.02407	-0.54546	0.585859	0.786791	chr16	9857925
cg11067712	0.020514	0.545147	0.586078	0.786948	chr11	105850644
cg14420982	0.018991	0.541386	0.588663	0.788568	chr9	80647609
cg23119809	-0.01788	-0.53968	0.589835	0.789245	chr17	72839750
cg04084618	-0.02947	-0.53951	0.589955	0.789334	chr5	36607065
cg06532779	0.027434	0.539163	0.590194	0.789474	chr19	1003622
cg07751222	-0.03068	-0.53834	0.590759	0.789867	chr4	102268245
cg08861556	0.0306	0.535646	0.592619	0.790953	chr16	850614
cg22857947	0.027524	0.535047	0.593033	0.791205	chr19	14225039
cg15623249	-0.02575	-0.53494	0.593105	0.791254	chr19	48896947
cg24481035	-0.0182	-0.53485	0.593172	0.791286	chr19	54388422
cg25423752	0.034446	0.534478	0.593426	0.791431	chr5	178422415
cg23143233	0.022645	0.531241	0.595664	0.792816	chr20	57465864
cg19885037	-0.01512	-0.53065	0.596071	0.793054	chr3	4762242
cg03357547	-0.02934	-0.53025	0.59635	0.793232	chr19	51165207
cg07843390	-0.01521	-0.52914	0.597119	0.7937	chr19	2541015
cg25228562	-0.01928	-0.52795	0.597946	0.794244	chr17	64718121
cg17300307	-0.01796	-0.52704	0.598574	0.794618	chr2	191744975
cg15128801	0.024108	0.525814	0.599425	0.795115	chr1	68202053
cg25326570	-0.02527	-0.52501	0.599983	0.795408	chr20	57426757
cg02620388	0.018226	0.525003	0.599988	0.795409	chr7	100271115
cg00091960	-0.01871	-0.52447	0.60036	0.795658	chr7	126829514
cg11407328	-0.01537	-0.52346	0.601062	0.796061	chr17	64759926
cg11836212	0.028492	0.522988	0.601388	0.796276	chr1	37447865
cg17885091	-0.0374	-0.5226	0.601654	0.796481	chr1	68299057
cg26279745	-0.0345	-0.52134	0.602535	0.797015	chr14	24801970
cg09722397	-0.01803	-0.52036	0.603216	0.79742	chr17	72855943
cg13715127	-0.01795	-0.52031	0.603252	0.797426	chr17	72856825
cg08779207	0.021302	0.519516	0.603803	0.79773	chr15	40586496

cg18423469	0.017577	0.518615	0.604431	0.798136	chr18	3726858
cg06922606	-0.01818	-0.51833	0.604628	0.798277	chr16	10274632
cg13344206	0.021907	0.517906	0.604924	0.79844	chr6	34051016
cg09239744	0.027346	0.517801	0.604998	0.798474	chr16	10276580
cg16707895	0.02688	0.517301	0.605346	0.798658	chr19	13394116
cg15114105	-0.04536	-0.51649	0.605915	0.79896	chr19	49944820
cg08074971	0.028105	0.516187	0.606123	0.799054	chr16	850562
cg14746605	-0.02215	-0.51592	0.60631	0.799158	chr11	35374336
cg08870587	-0.02753	-0.51543	0.60665	0.799384	chr11	70455278
cg18352793	0.011854	0.513956	0.60768	0.800096	chr22	22210625
cg11985287	0.01456	0.513034	0.608324	0.800525	chr19	2556805
cg15814923	-0.04318	-0.5124	0.608769	0.800805	chr19	14228610
cg14285012	0.021515	0.511488	0.609404	0.801258	chr9	104357967
cg06065549	-0.0201	-0.51148	0.609411	0.801258	chr20	57427443
cg20259256	-0.0256	-0.51118	0.609618	0.801388	chr3	142443266
cg15160445	-0.02612	-0.50872	0.611341	0.802454	chr20	57426749
cg14898177	-0.01689	-0.50802	0.611831	0.802778	chr12	2486634
cg12190341	0.010113	0.507136	0.61245	0.803185	chr17	72838819
cg20152382	0.01625	0.506686	0.612766	0.803329	chr17	64783099
cg12413242	-0.01059	-0.50661	0.612821	0.803359	chr12	2292890
cg06525750	-0.01953	-0.50428	0.614453	0.804348	chr2	25141152
cg27109030	-0.02198	-0.50372	0.614849	0.804502	chr19	2702898
cg03945800	-0.02306	-0.5027	0.61556	0.804951	chr16	4165515
cg15985106	0.013706	0.502023	0.616038	0.80525	chr12	6954791
cg23982607	-0.03979	-0.50124	0.616587	0.805578	chr1	1823379
cg24131262	0.015042	0.501093	0.616691	0.805631	chr3	4549756
cg24889366	0.02382	0.501009	0.61675	0.805671	chr16	850646
cg05693127	0.016759	0.500931	0.616805	0.805709	chr6	33643684
cg06500096	0.020044	0.49957	0.617762	0.806253	chr19	13405582
cg00940140	0.031889	0.497849	0.618973	0.806909	chr20	57480494
cg04677683	-0.02435	-0.49705	0.619536	0.807267	chr20	57426743
cg26204322	-0.04959	-0.49703	0.619547	0.807267	chr11	64018687
cg08994082	0.022333	0.496821	0.619697	0.807372	chr19	2525459
cg24617313	-0.07455	-0.49602	0.620263	0.807687	chr20	57427146
cg01347776	0.017272	0.493244	0.622219	0.808837	chr3	179121836
cg24036292	-0.01569	-0.49297	0.622415	0.808951	chr11	70416269
cg23816431	0.022348	0.492248	0.622922	0.809259	chr3	171524969
cg02084729	-0.01893	-0.4922	0.622956	0.809266	chr6	33589691
cg27567416	0.020731	0.489147	0.625112	0.810521	chr16	4117281
cg15355952	0.030318	0.48761	0.6262	0.811279	chr5	36662829
cg00652727	-0.02589	-0.48553	0.627673	0.812201	chr1	235812198
cg13851211	-0.01383	-0.48466	0.628287	0.812597	chr16	50321678
cg01344243	-0.02759	-0.48346	0.629138	0.813209	chr16	9855280
cg11661914	-0.04079	-0.48314	0.629363	0.813342	chr12	49180849
cg08111661	0.016304	0.482758	0.629637	0.813527	chr12	2223540
cg11227541	-0.02059	-0.48241	0.629884	0.813684	chr1	84972317
cg22023664	0.010185	0.480824	0.631009	0.814438	chr19	2619608
cg06324048	-0.02169	-0.48074	0.631069	0.814476	chr20	57427103

cg05558390	0.013987	0.48046	0.631268	0.8146	chr20	57415377
cg05795849	-0.02052	-0.47965	0.631843	0.814963	chr3	4794082
cg19696388	0.014972	0.479027	0.632285	0.815217	chr19	19042720
cg11293016	0.023289	0.4764	0.634154	0.816365	chr19	54402647
cg24676071	-0.03264	-0.47507	0.635098	0.816897	chr7	45613410
cg02374107	-0.01316	-0.47471	0.635354	0.817034	chr16	4163907
cg18982286	-0.0248	-0.47388	0.635949	0.817362	chr22	51136325
cg03303857	-0.02006	-0.47309	0.636508	0.817726	chr11	120619307
cg06716686	-0.01635	-0.47287	0.636665	0.817843	chr3	4535154
cg26811638	-0.02479	-0.47199	0.637293	0.81825	chr20	57427493
cg24226238	0.014095	0.469091	0.639362	0.81961	chr11	120530774
cg07964163	-0.01697	-0.46723	0.640695	0.820433	chr20	57413417
cg00578437	0.014854	0.466881	0.640941	0.820558	chr22	22217249
cg09604333	0.007718	0.466131	0.641476	0.820871	chr20	57465125
cg04266169	-0.01705	-0.46596	0.641596	0.820941	chr1	1822862
cg18554395	0.017968	0.465535	0.641903	0.821102	chr19	2555791
cg05627987	0.020167	0.464212	0.642849	0.82171	chr19	51220286
cg20098420	0.01449	0.463077	0.643662	0.822173	chr22	51155589
cg26752663	0.011044	0.462208	0.644284	0.82253	chr2	25142016
cg03626208	-0.01295	-0.46133	0.644912	0.822854	chr12	2443169
cg07582829	0.01979	0.461276	0.644951	0.822875	chr19	54408440
cg17174980	-0.02257	-0.46034	0.645624	0.823318	chr12	14109514
cg08091561	-0.03136	-0.4588	0.64673	0.824007	chr20	57426425
cg11207372	0.01837	0.457885	0.647383	0.824385	chr11	70385365
cg01833923	-0.00798	-0.45754	0.647632	0.824589	chr17	47286719
cg16399393	-0.01516	-0.45628	0.648533	0.825235	chr11	70368768
cg13631572	-0.02039	-0.45556	0.649055	0.825497	chr14	24803903
cg08370077	0.020019	0.454237	0.650003	0.826063	chr16	851288
cg16916688	0.022346	0.452943	0.650933	0.826596	chr6	34101441
cg15908975	0.029553	0.451383	0.652056	0.827233	chr7	126698829
cg20788479	-0.01591	-0.45132	0.652099	0.827245	chr3	179169536
cg10622236	-0.01379	-0.45111	0.652249	0.827314	chr15	42120082
cg25229306	0.010804	0.448658	0.654018	0.828419	chr20	57426374
cg03630683	-0.01752	-0.44825	0.654311	0.828614	chr3	4534997
cg25594486	-0.03679	-0.44794	0.654536	0.828762	chr19	51165441
cg12345953	0.021142	0.445771	0.6561	0.829578	chr4	102207435
cg02858053	-0.0243	-0.44558	0.656237	0.829624	chr1	84971927
cg02371119	0.017518	0.444861	0.656757	0.829895	chr3	171527346
cg12079381	0.025075	0.442479	0.658477	0.830844	chr21	31310920
cg07938763	0.01611	0.442052	0.658786	0.830981	chr19	2516966
cg06887224	-0.01281	-0.44159	0.659123	0.831146	chr5	7399037
cg17841572	-0.01011	-0.44149	0.659194	0.831186	chr20	57426368
cg24778538	-0.01418	-0.44043	0.659959	0.831671	chr12	14131975
cg11658986	0.012697	0.440024	0.660252	0.831851	chr12	49177605
cg24146288	-0.02108	-0.43975	0.66045	0.831941	chr15	42302342
cg12463346	-0.03212	-0.43952	0.66062	0.832042	chr4	102268854
cg03263685	-0.01827	-0.43938	0.66072	0.832084	chr2	68480160
cg13885159	0.016166	0.43906	0.66095	0.832182	chr11	62473858

cg17298543	-0.01734	-0.43897	0.661017	0.832223	chr7	79802016
cg06868991	0.015375	0.437039	0.662413	0.833122	chr11	70774323
cg15333818	0.018516	0.436715	0.662648	0.833235	chr12	46766724
cg06465194	-0.02048	-0.43661	0.662724	0.833291	chr11	120531034
cg01935096	0.020371	0.436343	0.662917	0.833385	chr11	70391215
cg21032008	-0.01405	-0.4343	0.664399	0.83427	chr3	7724717
cg12743970	0.015571	0.430779	0.666953	0.835856	chr17	64536520
cg08966293	0.016252	0.430126	0.667428	0.836151	chr16	30134858
cg17509220	-0.028	-0.42886	0.668346	0.836688	chr19	13617012
cg21725954	-0.02988	-0.42816	0.668856	0.836981	chr6	146348890
cg04708601	0.01534	0.427912	0.669037	0.837066	chr6	101880078
cg09845015	0.011277	0.427305	0.669479	0.837316	chr11	70458994
cg21938532	-0.01226	-0.42635	0.670173	0.837684	chr20	57426931
cg02847220	0.020485	0.425412	0.670856	0.838111	chr3	171523157
cg22827707	0.01432	0.423015	0.672603	0.839122	chr6	34100899
cg24661595	-0.01632	-0.42253	0.672952	0.839317	chr11	70456848
cg07008386	-0.01719	-0.42085	0.674181	0.840005	chr20	8113630
cg12446629	-0.01997	-0.42035	0.674548	0.840193	chr8	132052044
cg22323942	-0.0377	-0.41754	0.676599	0.841401	chr19	2543655
cg27122965	0.016817	0.415069	0.678403	0.842492	chr11	70882053
cg13948330	0.011915	0.415017	0.67844	0.842506	chr11	70559064
cg23848889	-0.01676	-0.41489	0.678533	0.842579	chr11	70455304
cg20325479	0.021602	0.413102	0.679841	0.843368	chr7	100271106
cg14792912	0.013854	0.410768	0.68155	0.844393	chr3	51742779
cg20152891	-0.02493	-0.41052	0.681734	0.844516	chr19	49944506
cg06044900	-0.0197	-0.40966	0.68236	0.844904	chr20	57467811
cg01360067	0.019833	0.407873	0.683672	0.845635	chr10	75256027
cg12451177	-0.01997	-0.40698	0.684328	0.845952	chr22	22222048
cg14120436	-0.01725	-0.40492	0.685835	0.846745	chr15	52483498
cg18949315	0.013352	0.404856	0.685886	0.846757	chr20	57418015
cg04908625	-0.02281	-0.40457	0.686093	0.846884	chr3	123166882
cg04107939	-0.01099	-0.40338	0.686972	0.847379	chr3	171520494
cg26226650	0.009723	0.403216	0.687091	0.847435	chr3	50276265
cg03601797	0.014636	0.402656	0.687502	0.847677	chr2	155556321
cg10241462	0.01396	0.400713	0.688931	0.848501	chr2	191746790
cg04017672	-0.01402	-0.39999	0.689466	0.848737	chr1	182359056
cg16377872	-0.0139	-0.39878	0.690355	0.849191	chr19	15084823
cg20231694	-0.01866	-0.39846	0.690589	0.849342	chr11	70691944
cg13824515	-0.0231	-0.39818	0.690795	0.849491	chr9	140047122
cg05087008	0.01584	0.398088	0.690862	0.849515	chr11	105483680
cg05035616	-0.01364	-0.39603	0.692381	0.850397	chr16	56374523
cg11343713	0.008514	0.395426	0.692823	0.85066	chr7	45749313
cg06840723	0.018183	0.39437	0.693601	0.851068	chr15	52484492
cg22990158	-0.03785	-0.39353	0.69422	0.851473	chr14	24802150
cg01255513	0.017648	0.392324	0.695111	0.852094	chr19	13365923
cg26670249	-0.01121	-0.39188	0.695438	0.8523	chr19	14223997
cg20240931	0.016974	0.390394	0.696536	0.852982	chr19	2613894
cg12043631	0.011912	0.388181	0.698171	0.853877	chr11	70643814

cg23465427	-0.01038	-0.3879	0.698382	0.854023	chr17	7111429
cg26424956	0.013802	0.38748	0.698689	0.854175	chr6	34101526
cg19006947	0.019447	0.387372	0.698769	0.854214	chr11	64034861
cg08266286	0.011145	0.387027	0.699024	0.85429	chr2	25141901
cg09444818	0.018676	0.384963	0.700551	0.855102	chr4	102097226
cg23325230	-0.01792	-0.38479	0.700683	0.855159	chr7	100272578
cg07783800	-0.01621	-0.38459	0.700829	0.855226	chr1	235803662
cg13300911	-0.01328	-0.38344	0.701676	0.85569	chr15	42119951
cg01846046	-0.02014	-0.38277	0.702176	0.855921	chr11	64034019
cg03503758	-0.01403	-0.38035	0.703966	0.856841	chr19	2614104
cg24737505	-0.01572	-0.38015	0.704117	0.85688	chr11	70564116
cg25963822	-0.01476	-0.37897	0.70499	0.857354	chr7	100270831
cg03697708	-0.02025	-0.37722	0.706288	0.858076	chr19	13617549
cg26265279	0.015786	0.37705	0.706416	0.858147	chr3	53566044
cg14099468	0.024383	0.376085	0.707133	0.858461	chr1	235814814
cg08076125	0.024752	0.375664	0.707445	0.858607	chr6	146350230
cg14943539	-0.0165	-0.37422	0.708515	0.859262	chr20	57420942
cg14295482	-0.01235	-0.37404	0.708653	0.859354	chr19	2555717
cg25781595	-0.01512	-0.37373	0.708884	0.859508	chr19	48918712
cg18571531	0.010637	0.37328	0.709216	0.859681	chr11	70477192
cg13399816	0.015987	0.373233	0.709252	0.859711	chr1	68299468
cg02664157	-0.02395	-0.37225	0.709984	0.860108	chr19	2702877
cg02423534	-0.00861	-0.37167	0.710414	0.860328	chr12	49160180
cg25335435	-0.02917	-0.37103	0.710893	0.860596	chr11	22399705
cg23484981	0.049807	0.370966	0.710937	0.86062	chr20	57426626
cg06344265	0.060968	0.370448	0.711323	0.860828	chr11	120530973
cg11264539	0.009465	0.370238	0.711479	0.860878	chr11	62474940
cg06836849	-0.01592	-0.37021	0.711499	0.860888	chr12	100751051
cg14843888	0.014838	0.369714	0.711869	0.861094	chr3	53530247
cg16582156	0.028831	0.368708	0.712618	0.861454	chr1	110091224
cg23332732	-0.01861	-0.36797	0.71317	0.861782	chr12	26986274
cg25556008	-0.01618	-0.36783	0.713269	0.861831	chr19	2525384
cg20668321	-0.01188	-0.36745	0.713551	0.861976	chr5	153192843
cg14317712	-0.01595	-0.36678	0.714058	0.862199	chr9	140034900
cg25316853	0.019745	0.366748	0.714078	0.862211	chr5	36606347
cg21810373	-0.01746	-0.36663	0.714166	0.862249	chr11	70917159
cg00536924	-0.01302	-0.36533	0.715138	0.862779	chr7	93551004
cg21330323	-0.00485	-0.36391	0.716195	0.863347	chr20	57414596
cg20915897	-0.01167	-0.36166	0.717877	0.864259	chr7	45717588
cg13469748	0.020084	0.361339	0.718113	0.864397	chr1	84971910
cg02063520	0.017444	0.361185	0.718228	0.864438	chr6	102516590
cg03335128	0.015922	0.360246	0.718929	0.864909	chr1	1731415
cg02046017	0.013363	0.357492	0.720987	0.866127	chr11	70707406
cg15093766	0.014716	0.356267	0.721904	0.866698	chr17	64408569
cg03140521	-0.01452	-0.35549	0.722487	0.866945	chr1	68299388
cg05313261	-0.03933	-0.35473	0.723052	0.867237	chr16	30134350
cg03836615	0.007335	0.354632	0.723127	0.867272	chr3	4856096
cg18229071	0.007819	0.35426	0.723405	0.867405	chr19	2695245

cg17151604	0.013622	0.35418	0.723465	0.867435	chr10	75197928
cg17202839	0.013409	0.353198	0.7242	0.867856	chr17	64685036
cg09902254	0.015626	0.352622	0.724632	0.868036	chr11	70858237
cg15631127	-0.00519	-0.3522	0.724945	0.868246	chr20	57426580
cg08481112	-0.02201	-0.35111	0.725766	0.868742	chr19	2544100
cg00233948	0.012716	0.350126	0.726502	0.869116	chr5	36619356
cg06111374	0.014537	0.34973	0.726799	0.869256	chr12	14109584
cg06171406	0.017555	0.34912	0.727256	0.869488	chr16	4050400
cg24220046	0.014284	0.347972	0.728117	0.869985	chr19	51171640
cg06223539	-0.02844	-0.34641	0.72929	0.870629	chr11	70517374
cg14975881	0.012064	0.346148	0.729486	0.87073	chr19	54389945
cg07237830	0.010134	0.345258	0.730154	0.871071	chr11	62474725
cg23055496	-0.01615	-0.34516	0.730226	0.8711	chr3	6906371
cg14167033	-0.0873	-0.34489	0.730427	0.871224	chr11	70424559
cg08462108	0.011499	0.34431	0.730867	0.871432	chr11	70680470
cg02098786	0.022581	0.344239	0.730919	0.871466	chr14	24801794
cg04421162	-0.01479	-0.34419	0.730956	0.871474	chr19	54406293
cg26648054	-0.0194	-0.34364	0.731367	0.871703	chr17	7117995
cg22294773	0.009568	0.342973	0.73187	0.871959	chr19	2606054
cg11264635	0.013281	0.342921	0.73191	0.87198	chr19	15083868
cg26739975	-0.02379	-0.34133	0.733105	0.872627	chr4	158144318
cg09336589	0.01444	0.341191	0.733211	0.872697	chr17	7107939
cg25090051	0.009365	0.340944	0.733396	0.872801	chr20	57414059
cg01588464	0.016085	0.340609	0.733648	0.87295	chr1	182360063
cg10504751	-0.02651	-0.34035	0.733842	0.873025	chr16	56390830
cg15129608	-0.0143	-0.34022	0.733941	0.873065	chr19	54393153
cg01895374	0.01209	0.339626	0.734388	0.87336	chr17	64536954
cg06697294	0.031293	0.339604	0.734404	0.873367	chr19	54385412
cg09980522	-0.01692	-0.33919	0.734719	0.873557	chr11	105481802
cg23982812	-0.02251	-0.33594	0.73716	0.874924	chr12	14004950
cg16175911	0.01307	0.335663	0.737371	0.875029	chr12	26985133
cg15812599	0.011564	0.334768	0.738045	0.875392	chr11	70849065
cg01817393	0.029523	0.333751	0.738812	0.875811	chr20	57427642
cg20830447	-0.01578	-0.33327	0.739177	0.875987	chr12	46764929
cg12198334	0.025875	0.332403	0.739829	0.876352	chr11	70692032
cg07105596	0.026078	0.331661	0.740388	0.876668	chr20	57427472
cg23399933	0.022543	0.331376	0.740603	0.876784	chr4	102112217
cg09340615	0.01055	0.329692	0.741874	0.877496	chr16	4021030
cg14418176	0.010031	0.329081	0.742335	0.877725	chr2	25050403
cg17838127	0.010907	0.328981	0.742411	0.877769	chr8	22298935
cg24081764	-0.01949	-0.32755	0.743494	0.878361	chr19	54402116
cg13650938	0.01583	0.326802	0.744057	0.878725	chr19	2579075
cg09885502	0.123355	0.326721	0.744118	0.878755	chr20	57463991
cg13707945	0.016478	0.326513	0.744275	0.87887	chr3	4714992
cg13559773	-0.01391	-0.32609	0.744594	0.87907	chr19	48562267
cg21409965	0.017322	0.325894	0.744743	0.879125	chr1	37283904
cg03217795	0.020581	0.32534	0.745162	0.879322	chr16	23847556
cg21429394	-0.01594	-0.32531	0.745187	0.879342	chr12	100750899

cg15972294	0.014273	0.32505	0.745381	0.879478	chr3	50273096		
cg25900614	0.023124	0.324905	0.745491	0.879532	chr7	126079083		
cg24039816	-0.01361	-0.32377	0.746352	0.879999	chr19	51220098		
cg12863967	0.01386	0.323564	0.746505	0.880089	chr7	93534920		
cg22218695	0.010931	0.323481	0.746568	0.880137	chr7	126446519		
cg02090654	0.016631	0.323399	0.74663	0.880175	chr7	126698344		
cg07793724	0.026793	0.323033	0.746906	0.880287	chr1	53609371		
cg01729401	0.016532	0.321381	0.748156	0.880959	chr1	1750560		
cg06088782	0.01161	0.320016	0.74919	0.881552	chr11	70563839		
cg18935491	-0.00794	-0.31837	0.750435	0.882296	chr20	57425979		
cg04583195	0.012432	0.317912	0.750784	0.882489	chr3	179165115		
cg05800983	-0.01463	-0.3174	0.75117	0.882711	chr6	34102530		
cg24591182	0.031148	0.317297	0.751249	0.882749	chr11	64019217		
cg02676523	-0.01174	-0.31712	0.751384	0.882787	chr16	4027674		
cg21233003	-0.01778	-0.31634	0.751974	0.883102	chr9	140057464		
cg02415992	0.013156	0.316126	0.752137	0.883175	chr16	10102278		
cg04132853	-0.00935	-0.31513	0.752891	0.883619	chr20	57414039		
cg01187464	0.011547	0.314434	0.753421	0.883929	chr16	50351302		
cg04153722	-0.01442	-0.31323	0.754336	0.884484	chr17	64783041		
cg04779428	0.015745	0.31307	0.754456	0.88453	chr20	57463355		
cg12568707	0.012121	0.31228	0.755055	0.884825	chr19	19042904		
cg15692593	-0.01554	-0.31186	0.755373	0.885006	chr6	101993140		
cg12282391	-0.01264	-0.31134	0.75577	0.885218	chr12	2162491	NM_00112	NM_00112
cg18225409	0.011203	0.311138	0.755922	0.885287	chr11	70713375		
cg13680388	0.01077	0.310584	0.756343	0.88553	chr20	57471844		
cg06563300	-0.01717	-0.31015	0.756672	0.88568	chr12	100750811		
cg18150383	0.016454	0.308311	0.75807	0.886502	chr19	49933217		
cg21997766	0.013709	0.306821	0.759203	0.887085	chr17	72846113		
cg09822192	0.019602	0.304158	0.761229	0.888172	chr14	24801191		
cg16848624	-0.02052	-0.30339	0.761814	0.888522	chr7	45614290		
cg20721022	-0.01724	-0.30335	0.761844	0.88854	chr19	54386355		
cg25322847	-0.01004	-0.30153	0.763226	0.889337	chr7	45617892		
cg24171047	-0.01495	-0.30095	0.763669	0.889593	chr17	64765921		
cg00041368	0.009636	0.300849	0.763748	0.889647	chr18	3879131		
cg13231680	0.00981	0.298118	0.765829	0.89079	chr3	7693146		
cg08992229	-0.01756	-0.29622	0.767278	0.891551	chr7	126866923		
cg21116900	-0.01247	-0.29607	0.767394	0.891584	chr12	100750760		
cg03969219	-0.01234	-0.2956	0.767754	0.891779	chr19	2611456		
cg18753811	0.015832	0.295487	0.767837	0.891824	chr12	2162232		
cg14637685	-0.00986	-0.29526	0.768011	0.891896	chr12	2411116		
cg09248655	-0.01342	-0.29338	0.769444	0.892585	chr19	48897955		
cg25556841	0.010839	0.291902	0.770573	0.893233	chr19	2511263		
cg12872693	0.014831	0.291009	0.771255	0.893625	chr3	179168798		
cg18399935	-0.01101	-0.29019	0.771881	0.893937	chr3	6906994		
cg08134671	0.017616	0.287796	0.773711	0.894886	chr19	2542837		
cg12246156	-0.0101	-0.28723	0.774142	0.895115	chr17	64522604		
cg13396607	-0.00997	-0.28555	0.775429	0.89577	chr6	102055059		
cg04169369	0.009879	0.28554	0.775437	0.895771	chr16	4053199		

cg14416930	0.01058	0.28471	0.776072	0.896064	chr17	64498178
cg16395366	-0.027	-0.2839	0.776692	0.896416	chr1	53558245
cg27491190	-0.00872	-0.28051	0.779291	0.897844	chr12	46767943
cg12204773	-0.01313	-0.27993	0.779736	0.898104	chr17	7123253
cg25293328	-0.01601	-0.27908	0.780383	0.898353	chr19	2611690
cg22090419	-0.01356	-0.27812	0.781119	0.898753	chr3	53844172
cg21157507	-0.00853	-0.27789	0.781298	0.898863	chr11	70830058
cg02647408	-0.01343	-0.27769	0.781451	0.898939	chr11	88241594
cg04696980	0.009192	0.277657	0.781476	0.898949	chr19	2586206
cg13934625	0.013819	0.277348	0.781713	0.899079	chr15	52472770
cg17354190	0.01387	0.27681	0.782126	0.899287	chr17	72856064
cg01871907	-0.01399	-0.2763	0.782516	0.899471	chr19	2703055
cg01035815	-0.01362	-0.27602	0.782735	0.899598	chr6	33600828
cg21961771	-0.0134	-0.27583	0.782874	0.899672	chr12	100750652
cg24640697	0.007138	0.275231	0.783338	0.899955	chr1	84970057
cg05780228	0.01281	0.275112	0.783429	0.899996	chr11	70713608
cg13052954	0.013743	0.274106	0.784201	0.900396	chr1	37467416
cg02959759	0.01884	0.273961	0.784313	0.900443	chr12	2801584
cg27356165	0.012511	0.273742	0.784481	0.900508	chr19	2613933
cg16669395	0.01587	0.27327	0.784843	0.900715	chr16	10208417
cg16182691	-0.00763	-0.27215	0.785703	0.901203	chr7	79762956
cg14111579	0.008706	0.27161	0.786118	0.901406	chr19	48614090
cg26314755	0.008839	0.269758	0.787541	0.902085	chr19	42550298
cg24190415	-0.02048	-0.26951	0.787729	0.902194	chr11	35441012
cg21970929	0.010438	0.269399	0.787817	0.902261	chr5	36608598
cg20000940	0.008186	0.269023	0.788106	0.902428	chr14	52327486
cg14176797	0.006303	0.268384	0.788597	0.90272	chr20	57426801
cg03657031	0.009028	0.268104	0.788812	0.90287	chr19	54385215
cg24058407	-0.00915	-0.26781	0.789041	0.902961	chr20	57428282
cg00007326	-0.01035	-0.26741	0.789346	0.903129	chr19	13366046
cg23323297	0.01197	0.26441	0.791655	0.904254	chr19	51195418
cg04987335	-0.01119	-0.26414	0.791861	0.904348	chr11	35288779
cg09241929	-0.01915	-0.26311	0.792658	0.904814	chr20	57465560
cg22749173	-0.00812	-0.26165	0.793781	0.905443	chr19	2614039
cg26968767	0.009228	0.256723	0.797577	0.907559	chr17	72843650
cg27270541	0.014686	0.255493	0.798526	0.908107	chr19	48614177
cg16279290	0.007855	0.25395	0.799717	0.908721	chr11	70368624
cg06485596	-0.00644	-0.25326	0.800252	0.908972	chr16	24112658
cg25419928	0.005272	0.250672	0.802247	0.910049	chr6	33656793
cg11706467	-0.01357	-0.25003	0.802743	0.91028	chr2	155554707
cg06986989	0.008374	0.2461	0.805782	0.911849	chr1	235802839
cg16361867	0.011197	0.245857	0.80597	0.911944	chr11	120581355
cg11663780	-0.00818	-0.245	0.806635	0.912317	chr19	1001892
cg01242196	0.006646	0.242259	0.808753	0.913392	chr6	33990181
cg23123694	0.011379	0.241717	0.809173	0.913583	chr12	46766543
cg07456314	-0.01085	-0.23986	0.810613	0.914363	chr17	4708968
cg23808301	-0.00881	-0.23925	0.811082	0.914587	chr17	4710015
cg04533189	0.009258	0.239151	0.81116	0.914649	chr17	64298763

cg09583957	-0.0138	-0.23854	0.811632	0.914896	chr20	57428315
cg16644457	0.012104	0.234366	0.814868	0.916507	chr11	22359480
cg13762474	0.013473	0.23335	0.815656	0.916955	chr15	42371808
cg21024916	0.011199	0.232775	0.816103	0.917175	chr3	4535815
cg11265916	-0.00645	-0.23268	0.816177	0.917188	chr22	22221056
cg25983305	-0.01191	-0.23099	0.817484	0.917909	chr8	22298586
cg01749530	-0.00914	-0.23074	0.817685	0.918021	chr10	75255289
cg04455869	0.008775	0.230237	0.818072	0.918284	chr5	7663853
cg18224653	-0.0058	-0.22902	0.819015	0.918793	chr20	57426979
cg19592829	0.004584	0.228659	0.819298	0.918943	chr20	57426215
cg11464074	0.008289	0.226519	0.82096	0.919764	chr7	126417126
cg15863841	-0.00749	-0.22532	0.821889	0.920225	chr3	171430173
cg18411237	0.011007	0.220969	0.825274	0.921931	chr11	70653173
cg08848088	-0.00717	-0.22005	0.82599	0.922258	chr1	235714526
cg04586622	0.009034	0.219772	0.826205	0.922348	chr2	25135609
cg00848945	0.014387	0.218252	0.827388	0.922881	chr12	2800919
cg11647651	0.013334	0.217791	0.827747	0.923042	chr17	4710373
cg08901242	-0.01186	-0.21734	0.828102	0.923179	chr19	15083667
cg15131024	0.012102	0.21649	0.828761	0.923479	chr11	70338408
cg00495303	0.026304	0.216433	0.828805	0.923501	chr18	3771110
cg05944877	0.007741	0.215821	0.829281	0.923761	chr16	24197863
cg26429499	0.011248	0.215718	0.829361	0.923761	chr11	70563792
cg15154232	-0.01276	-0.21483	0.830054	0.92412	chr19	48615306
cg24591824	0.007993	0.213114	0.83139	0.924825	chr12	2762732
cg27173374	0.010571	0.212793	0.83164	0.924933	chr14	52413159
cg05564552	-0.00705	-0.21195	0.832294	0.925254	chr15	42120091
cg14616584	0.008999	0.211608	0.832564	0.925403	chr1	37388124
cg16815991	-0.01145	-0.2115	0.832644	0.925451	chr12	14133129
cg17959824	0.008191	0.211175	0.832901	0.925559	chr11	70391706
cg04037585	0.007387	0.210767	0.833219	0.925656	chr16	56231292
cg09433558	0.010051	0.210474	0.833448	0.925802	chr3	171412917
cg16685860	0.017445	0.208571	0.834931	0.926533	chr17	4710619
cg23019936	-0.01296	-0.20845	0.835025	0.926573	chr12	13903266
cg13280108	-0.00893	-0.20774	0.83558	0.926806	chr11	70398751
cg13070193	-0.00736	-0.2068	0.836317	0.927222	chr7	45613752
cg13878641	-0.00778	-0.2058	0.837096	0.927604	chr1	110090951
cg04019914	-0.00916	-0.20531	0.837472	0.927792	chr20	57463357
cg17895496	-0.00642	-0.20477	0.837897	0.928015	chr15	42449716
cg17074573	0.007083	0.2047	0.837953	0.928026	chr22	51165537
cg04355871	-0.00812	-0.2042	0.83834	0.928212	chr11	64022825
cg14995148	-0.00636	-0.20285	0.839398	0.928717	chr5	36683916
cg24646457	-0.00778	-0.20247	0.839693	0.928835	chr15	42360292
cg14003231	-0.00724	-0.2005	0.84123	0.929538	chr6	33640908
cg10538151	-0.01219	-0.20035	0.841348	0.929571	chr9	140033364
cg18379295	0.006004	0.200105	0.841541	0.929679	chr14	52326155
cg15144016	0.009992	0.199341	0.842137	0.930001	chr3	51749782
cg08413366	0.008137	0.198156	0.843063	0.930359	chr10	75255930
cg23475725	0.006765	0.197963	0.843215	0.93045	chr12	2734205

cg02946850	0.008842	0.195701	0.844983	0.931307	chr7	126882944
cg06210447	-0.00946	-0.19568	0.844998	0.931307	chr11	70601842
cg19796640	-0.00693	-0.19517	0.845396	0.931493	chr17	72848197
cg27068206	-0.00755	-0.19344	0.846749	0.932107	chr11	70559053
cg08021532	0.0169	0.19209	0.847808	0.932591	chr16	50321878
cg08436756	0.008937	0.191934	0.84793	0.932621	chr11	70781118
cg13455717	-0.00892	-0.19162	0.848178	0.932686	chr1	235814365
cg14331853	0.008314	0.189857	0.849556	0.933409	chr9	140054850
cg09403559	0.005721	0.187032	0.851768	0.934525	chr16	56334857
cg18420143	-0.00653	-0.18517	0.853228	0.935287	chr17	7123125
cg04122657	-0.01034	-0.18475	0.853559	0.935423	chr16	4014295
cg04898487	0.007926	0.184402	0.853829	0.93553	chr16	10272607
cg03282345	0.008792	0.183538	0.854506	0.935854	chr19	49934577
cg13148511	0.010286	0.183519	0.854521	0.935862	chr17	4710380
cg20091384	0.006503	0.180596	0.856813	0.936834	chr19	2700927
cg03315058	-0.00985	-0.18034	0.857014	0.936977	chr11	62476542
cg09947844	0.006372	0.178707	0.858295	0.937552	chr16	4163819
cg16124935	-0.00996	-0.17518	0.861065	0.938763	chr11	70559616
cg01332711	-0.00605	-0.17394	0.862039	0.939235	chr15	42120681
cg14082123	0.006029	0.1739	0.862067	0.939237	chr15	42367977
cg17986880	0.00669	0.172964	0.862802	0.93961	chr7	79848290
cg21163960	0.013534	0.172949	0.862814	0.93962	chr11	35441777
cg19781472	-0.01006	-0.17065	0.864619	0.940463	chr12	56883202
cg27014608	-0.00885	-0.16984	0.865258	0.940772	chr16	4166952
cg22934516	-0.00954	-0.16943	0.865577	0.940947	chr11	35413951
cg25439807	0.018174	0.169077	0.865855	0.941059	chr18	3771151
cg11112257	-0.00726	-0.16857	0.866251	0.941257	chr11	88781135
cg10573143	-0.00621	-0.16814	0.866596	0.941422	chr11	70628992
cg01025883	0.006571	0.168019	0.866687	0.941456	chr16	23867088
cg17818798	-0.00609	-0.1677	0.86694	0.941604	chr19	14228473
cg24446178	-0.00749	-0.16732	0.867236	0.941724	chr12	100750702
cg12986110	0.0062	0.165893	0.868359	0.942247	chr19	48551504
cg14100184	-0.00729	-0.16566	0.868541	0.942357	chr16	851298
cg27591117	-0.00769	-0.16532	0.868806	0.942453	chr20	8113191
cg22989942	-0.00352	-0.16501	0.86905	0.942512	chr20	57426950
cg13574337	0.008497	0.163732	0.870057	0.942948	chr16	4016720
cg02740128	0.005572	0.163608	0.870155	0.943005	chr17	7123860
cg09257092	-0.00906	-0.16337	0.870343	0.943118	chr12	26986805
cg14907788	-0.00523	-0.16291	0.870703	0.943216	chr19	2555976
cg25399541	-0.00805	-0.16287	0.870734	0.943226	chr7	45622395
cg06401532	0.006076	0.162508	0.871021	0.94334	chr16	24220008
cg08942894	-0.00535	-0.16096	0.87224	0.943946	chr15	83563792
cg06293172	0.006967	0.159249	0.873585	0.944724	chr2	25045211
cg22335074	-0.00733	-0.15914	0.873667	0.94477	chr11	70733258
cg06772874	-0.00591	-0.15864	0.874063	0.944974	chr1	110090953
cg26968025	-0.00362	-0.15437	0.877426	0.946539	chr17	64519943
cg11637718	-0.00418	-0.15416	0.87759	0.946637	chr16	4029254
cg01903557	-0.00456	-0.15349	0.878119	0.946891	chr3	179169602

cg03510435	0.007834	0.153235	0.878321	0.946989	chr12	14094558
cg07838205	-0.01108	-0.15256	0.878852	0.947258	chr1	110091179
cg01140008	0.006331	0.150337	0.880605	0.948188	chr6	34002114
cg03679394	-0.00706	-0.14942	0.881325	0.948517	chr11	70516997
cg02409125	0.008014	0.147839	0.882574	0.949058	chr7	126889555
cg13804196	-0.00588	-0.14743	0.882895	0.949202	chr9	71628906
cg26645082	0.007796	0.147366	0.882947	0.949221	chr11	70563264
cg02520816	-0.00847	-0.14714	0.883124	0.949296	chr16	4056403
cg19548470	0.004225	0.145877	0.884122	0.94979	chr18	3880510
cg23815646	0.010203	0.142784	0.886561	0.950866	chr8	131961143
cg02615582	0.00591	0.141857	0.887293	0.951202	chr19	49939549
cg10815152	0.008096	0.139182	0.889404	0.952186	chr6	102098727
cg16560679	-0.00408	-0.13903	0.889522	0.952233	chr7	100276684
cg03511974	-0.00633	-0.13737	0.890831	0.952748	chr1	53568259
cg08263099	0.013629	0.137293	0.890896	0.952764	chr19	54410160
cg02660823	0.009049	0.136682	0.891379	0.952986	chr19	54410305
cg14022022	0.006751	0.135405	0.892387	0.953452	chr9	140055728
cg01286319	0.004859	0.135059	0.89266	0.95359	chr19	2695343
cg16358215	-0.011	-0.13476	0.892899	0.953677	chr11	70455662
cg22405973	0.004989	0.130122	0.896561	0.955389	chr22	22222028
cg02591871	0.01018	0.126758	0.899221	0.956574	chr19	14228565
cg21615915	0.006783	0.126501	0.899424	0.95667	chr6	102295679
cg26568075	0.004171	0.126098	0.899743	0.956825	chr1	1718809
cg14121185	0.004661	0.125058	0.900565	0.957207	chr17	64488849
cg20779373	-0.00617	-0.11944	0.905011	0.959174	chr1	37428969
cg09209803	0.005558	0.117974	0.906171	0.959717	chr6	33588932
cg04338055	0.004912	0.115806	0.907887	0.960483	chr19	1000955
cg05577548	-0.00753	-0.11546	0.908159	0.960596	chr11	70666748
cg02780849	-0.0046	-0.11523	0.908343	0.960661	chr1	235814163
cg11895615	-0.00472	-0.11387	0.909424	0.961114	chr12	2224518
cg04747226	-0.01213	-0.11167	0.91116	0.961871	chr11	105481319
cg01697794	0.005393	0.111389	0.911386	0.961972	chr17	7117125
cg01943657	-0.0074	-0.11074	0.911904	0.962262	chr4	102268799
cg01900555	0.002545	0.110611	0.912002	0.962305	chr12	6948846
cg01192061	-0.00335	-0.11051	0.912081	0.962327	chr11	70368264
cg05297437	0.004459	0.107955	0.914107	0.963227	chr20	57471672
cg02748316	-0.00886	-0.10552	0.916039	0.964032	chr3	50273710
cg16728539	0.003329	0.104515	0.916834	0.964428	chr12	2451169
cg23425324	0.003443	0.104311	0.916996	0.964491	chr12	26986193
cg25193077	-0.00546	-0.10213	0.918724	0.96529	chr1	235812109
cg13213810	0.004587	0.101071	0.919565	0.965674	chr22	51158720
cg17702518	-0.00337	-0.10056	0.919966	0.965853	chr7	100271260
cg10583180	0.005218	0.098224	0.921823	0.966737	chr6	101851354
cg01962496	0.004686	0.098078	0.921939	0.966778	chr5	78809740
cg19367232	0.00525	0.091401	0.927238	0.969063	chr2	68478649
cg07391392	0.003777	0.090444	0.927997	0.969389	chr5	7826900
cg07546293	0.004244	0.090332	0.928087	0.969394	chr16	851255
cg16737409	-0.00424	-0.09006	0.928303	0.969497	chr20	57428366

cg03551401	0.005999	0.087484	0.930348	0.970393	chr8	132051228
cg09066361	-0.00739	-0.08602	0.931509	0.970866	chr7	126890254
cg10768900	0.00367	0.085404	0.932	0.97109	chr11	70557881
cg03830585	-0.00452	-0.08509	0.932248	0.971204	chr3	4536777
cg02993882	-0.00207	-0.08383	0.933249	0.971686	chr16	4043463
cg16993684	0.008539	0.083223	0.933733	0.971915	chr20	57466131
cg25350198	-0.00426	-0.08024	0.936105	0.973115	chr1	84971850
cg22304522	-0.00338	-0.07951	0.936684	0.973363	chr8	131914037
cg02799411	-0.0023	-0.07869	0.937334	0.973666	chr3	4794061
cg06693667	-0.00259	-0.07847	0.937506	0.973769	chr20	57426570
cg06170425	0.002384	0.077927	0.937941	0.973937	chr16	4164087
cg03866831	-0.00514	-0.07742	0.938346	0.974119	chr16	9849427
cg12965344	0.005777	0.077389	0.938368	0.974129	chr19	48898160
cg16862315	0.004329	0.077177	0.938537	0.974209	chr17	7123138
cg00202454	0.006524	0.075939	0.939521	0.974604	chr15	42371886
cg05514043	0.005196	0.075548	0.939831	0.974706	chr9	140040822
cg09103960	-0.00264	-0.07412	0.940964	0.97521	chr16	56225504
cg10243075	0.004	0.073275	0.941638	0.97549	chr19	13615439
cg08290212	-0.00408	-0.07302	0.941841	0.975589	chr11	62473659
cg08587534	-0.00264	-0.07253	0.942232	0.975744	chr20	57427503
cg26739691	0.002477	0.072167	0.94252	0.97589	chr7	45637270
cg15591578	-0.00267	-0.07209	0.942578	0.975912	chr19	51219167
cg18870258	-0.00464	-0.07159	0.942982	0.976073	chr20	9460935
cg10168763	0.004424	0.070126	0.944142	0.976553	chr16	4166767
cg22741626	-0.00295	-0.06984	0.944367	0.976609	chr20	57463265
cg09053902	0.004757	0.069288	0.944809	0.976805	chr16	4034298
cg19815589	-0.0034	-0.06808	0.945771	0.977234	chr11	70709062
cg00980784	0.002241	0.0673	0.94639	0.9775	chr17	47287577
cg21151432	0.002477	0.066455	0.947062	0.977798	chr2	25142229
cg14583606	0.004919	0.06528	0.947996	0.978133	chr9	4490315
cg17540496	0.002215	0.063701	0.949253	0.978642	chr3	53845930
cg05648629	-0.00339	-0.06359	0.94934	0.97869	chr16	4162203
cg00426976	0.001925	0.062418	0.950274	0.97911	chr22	51140977
cg11023668	0.004305	0.06042	0.951863	0.97984	chr2	25095040
cg16102063	0.003393	0.060258	0.951992	0.979867	chr8	22298240
cg25308079	0.001074	0.060081	0.952133	0.979898	chr20	57463763
cg16835502	-0.00236	-0.05977	0.952379	0.980022	chr17	4710020
cg10599507	0.001868	0.059091	0.952921	0.980268	chr6	33653337
cg23249369	0.00242	0.058906	0.953068	0.980353	chr20	57426759
cg05340495	-0.00172	-0.05763	0.95408	0.98078	chr14	52327368
cg25960479	-0.00182	-0.05692	0.954647	0.980993	chr11	88243569
cg14960282	0.002181	0.055348	0.9559	0.981591	chr1	37321669
cg05309239	-0.00128	-0.05403	0.956946	0.982049	chr20	57427017
cg25314445	0.002018	0.053349	0.957491	0.982284	chr1	1718835
cg08240335	-0.00397	-0.05329	0.957539	0.9823	chr3	50273314
cg00646241	0.002504	0.051349	0.959083	0.982988	chr11	70563878
cg03344105	-0.00106	-0.04954	0.960523	0.98357	chr20	57426131
cg03211327	-0.00162	-0.04855	0.961312	0.983951	chr15	52470919

cg03768297	-0.00175	-0.0484	0.961434	0.983992	chr15	52441108
cg16108726	-0.00246	-0.0465	0.962945	0.984625	chr11	70781009
cg13714844	-0.00177	-0.04601	0.963338	0.98482	chr9	114422486
cg04675204	0.001808	0.044567	0.964483	0.985229	chr16	10179771
cg04086239	-0.0016	-0.0435	0.965335	0.985628	chr16	24067174
cg03962451	-0.00161	-0.04339	0.965423	0.985663	chr2	191754464
cg19365406	0.002088	0.042332	0.966264	0.985981	chr9	140043007
cg07982896	-0.00235	-0.04159	0.966855	0.986272	chr19	13365938
cg10002103	-0.00218	-0.04097	0.967346	0.986459	chr12	46766730
cg11070176	0.001136	0.040762	0.967514	0.986545	chr11	70489806
cg19161850	-0.00364	-0.04017	0.967988	0.986731	chr22	22222040
cg18375707	0.001732	0.039954	0.968158	0.986804	chr11	64034959
cg17902551	-0.00122	-0.03928	0.968697	0.987034	chr12	2801061
cg05147077	0.001607	0.037612	0.970023	0.987614	chr5	36606601
cg03015368	0.00149	0.035357	0.971819	0.98846	chr16	10065222
cg18997188	-0.00123	-0.03438	0.972599	0.988716	chr20	57463270
cg26767990	-0.00067	-0.03341	0.973374	0.989082	chr20	57463615
cg13701180	0.001249	0.03307	0.973642	0.989232	chr19	2513436
cg02392737	-0.00117	-0.03119	0.975138	0.989955	chr16	4136367
cg16904585	0.001208	0.028441	0.97733	0.990933	chr16	10276119
cg00296378	-0.00105	-0.02791	0.97775	0.991097	chr12	49177153
cg14560133	0.001051	0.0275	0.97808	0.991215	chr19	51199453
cg02775369	0.001148	0.027352	0.978198	0.991267	chr16	56316221
cg17377463	0.000743	0.027005	0.978475	0.991334	chr19	48908557
cg02261541	0.001039	0.026141	0.979163	0.991594	chr16	4050315
cg09906922	-0.00101	-0.02573	0.979492	0.991703	chr19	14203252
cg04106389	0.00083	0.025049	0.980034	0.991919	chr17	7117241
cg08894891	-0.00125	-0.0249	0.980153	0.991925	chr19	19040364
cg19013391	0.000901	0.023496	0.981271	0.992395	chr3	123166774
cg06622135	0.00076	0.021482	0.982876	0.993019	chr11	70474416
cg17221095	0.000502	0.021422	0.982924	0.993039	chr7	45717651
cg05312962	-0.00058	-0.02061	0.983569	0.993311	chr19	2576254
cg20789595	-0.00066	-0.02057	0.983605	0.993311	chr3	123063477
cg24788034	-0.00077	-0.01916	0.984728	0.993774	chr19	2588241
cg08161922	0.0012	0.017821	0.985794	0.994196	chr12	2163608
cg04498349	0.000981	0.016974	0.986469	0.994415	chr16	10274317
cg24867180	-0.00082	-0.01682	0.986591	0.994444	chr15	42120426
cg17047106	-0.00081	-0.01435	0.988564	0.995246	chr8	131961479
cg05824594	-0.00032	-0.01361	0.989149	0.995486	chr12	2734503
cg16864295	0.000523	0.012958	0.98967	0.995688	chr3	171463753
cg24204556	-0.00085	-0.01255	0.989999	0.995849	chr22	22222030
cg00672228	-0.00043	-0.01225	0.990233	0.995946	chr17	7123130
cg26632831	0.000425	0.011747	0.990636	0.996117	chr11	70935863
cg14847975	0.000858	0.011681	0.990688	0.996124	chr12	26986502
cg04507426	0.001104	0.011641	0.99072	0.996125	chr16	56229180
cg16418734	0.000383	0.011327	0.990971	0.996173	chr1	1720537
cg17839611	0.000264	0.010375	0.991729	0.996489	chr17	47286802
cg24868926	-0.00065	-0.01021	0.991861	0.996534	chr1	182360594

cg08626004	0.000515	0.007898	0.993704	0.997278	chr19	2513687
cg20401058	-0.00017	-0.00618	0.995073	0.997882	chr20	57426240
cg05100017	-0.00026	-0.00552	0.995598	0.998079	chr6	34102222
cg01047778	-0.00012	-0.00424	0.996621	0.998511	chr11	70584252
cg05684300	2.55E-05	0.000737	0.999413	0.999716	chr4	102267366

NM_00112	NM_00112	NM_00112	NM_00112	NM_00112	NM_00112	NM_00112	NM_00112	5'UTR	5'UTR

5'UTR 1stExon 1stExon 1stExon 5'UTR 5'UTR 1stExon 1stExon 1stExon 1stExon

1stExon	5'UTR	5'UTR	1stExon	1stExon	1stExon	1stExon	5'UTR	5'UTR	5'UTR

5'UTR 1stExon 1stExon 5'UTR 1stExon 5'UTR 5'UTR 1stExon 1stExon 1stExon

5'UTR	5'UTR	1stExon	5'UTR	1stExon	5'UTR	5'UTR	5'UTR	5'UTR	5'UTR
