# Supplementary material for: Differential DNA Methylation of the Serotonin Receptor Signaling and Glutamatergic Synapse Pathways in Adult Twins Born Preterm
Source: Genes (Basel). 2026 Jun 10;17(6):683. doi: 10.3390/genes17060683 (PMC13299586; doi:10.3390/genes17060683)
Supplement: Supplementary file 1 [file genes-17-00683-s001.zip › Supplementary Table S4_glutamate_old.pdf]

CpG	logFC	t	P.Value	adj.P.Val	chr	pos
cg02928699	0.187429	6.17203	2.30E-09	1.09E-05	chr11	88241946
cg08379637	0.123709	5.822958	1.55E-08	3.41E-05	chr21	31311387
cg07060551	0.23928	5.613966	4.68E-08	6.77E-05	chr19	51198381
cg21806090	-0.2696	-5.46761	9.95E-08	0.000109	chr12	2198034
cg17605476	0.163151	5.323942	2.06E-07	0.000165	chr4	158143917
cg07365960	0.271547	5.268123	2.72E-07	0.00019	chr17	72848535
cg26801014	0.162514	5.215732	3.52E-07	0.000222	chr11	88241769
cg19727439	0.289929	5.143788	5.01E-07	0.000271	chr1	37500508
cg01737026	0.138259	5.139354	5.12E-07	0.000273	chr6	101847656
cg25148589	0.177	5.073467	7.04E-07	0.000333	chr4	158141936
cg00439658	0.176157	5.038834	8.32E-07	0.00037	chr17	72848669
cg08955995	0.262046	5.019173	9.14E-07	0.000393	chr19	42503412
cg26746936	0.398053	4.951943	1.26E-06	0.000479	chr19	42503392
cg05942459	0.238816	4.947742	1.28E-06	0.000484	chr6	101846805
cg19206040	0.260061	4.909846	1.53E-06	0.000541	chr1	37500441
cg09649610	0.150633	4.894403	1.65E-06	0.000568	chr1	235814039
cg25744767	0.366725	4.802933	2.53E-06	0.000728	chr7	79764178
cg07642043	0.213758	4.77541	2.87E-06	0.000781	chr16	10276674
cg26351229	0.130595	4.757988	3.11E-06	0.000816	chr8	132053780
cg15519474	-0.12971	-4.72775	3.57E-06	0.000879	chr17	64300902
cg19507068	0.431313	4.706637	3.93E-06	0.000933	chr7	79764176
cg27262796	-0.08947	-4.69677	4.11E-06	0.000956	chr20	57426858
cg14036830	0.582554	4.686955	4.30E-06	0.000978	chr19	42503207
cg25885280	0.238333	4.677367	4.49E-06	0.001009	chr11	70760166
cg09434500	0.296997	4.621072	5.78E-06	0.001186	chr19	42502897
cg04908625	0.181375	4.619228	5.83E-06	0.001192	chr3	123166882
cg22762091	0.132658	4.591621	6.60E-06	0.001294	chr8	132052843
cg08958294	0.198473	4.58884	6.68E-06	0.001305	chr6	146350131
cg05346491	-0.08685	-4.57823	7.00E-06	0.001341	chr19	48917104
cg20818778	0.128094	4.571542	7.21E-06	0.001372	chr1	235814145
cg01942127	0.137322	4.491321	1.03E-05	0.001699	chr3	53529259
cg10591607	0.141698	4.450398	1.23E-05	0.001903	chr6	101846916
cg13483026	0.156381	4.436908	1.30E-05	0.001988	chr3	123167973
cg27199820	0.266369	4.415043	1.43E-05	0.002106	chr3	6903019
cg17168836	-0.13962	-4.38527	1.63E-05	0.00229	chr1	68256161
cg22187630	0.136685	4.349663	1.90E-05	0.002531	chr19	13616871
cg00582971	0.338311	4.306977	2.28E-05	0.002824	chr5	178422128
cg22541254	0.126984	4.284177	2.51E-05	0.002977	chr6	101846779
cg17298751	0.143259	4.272873	2.63E-05	0.003072	chr11	22363370
cg11308643	0.117994	4.267157	2.69E-05	0.003117	chr11	105480788
cg10785385	-0.08634	-4.23433	3.09E-05	0.003391	chr22	51114364
cg24868359	0.138108	4.231461	3.13E-05	0.003418	chr21	31312535
cg17199007	0.107552	4.151258	4.37E-05	0.004197	chr6	146349527
cg17799599	0.175601	4.10591	5.26E-05	0.004728	chr17	64787605
cg01817393	-0.25335	-4.0824	5.79E-05	0.005019	chr20	57427642
cg00553487	-0.10972	-4.02398	7.33E-05	0.005877	chr19	42570406
cg16415870	0.129712	4.022647	7.37E-05	0.00589	chr3	6904261

cg08475096	0.125407	4.00102	8.04E-05	0.00624	chr4	158143750
cg23460210	-0.14248	-3.99233	8.32E-05	0.00639	chr19	54385404
cg08586541	0.21284	3.939384	0.000103	0.007323	chr19	51198888
cg10904109	0.168176	3.928794	0.000107	0.00751	chr6	146755494
cg26316946	0.138392	3.908643	0.000116	0.007873	chr6	101846967
cg25909396	-0.10183	-3.90711	0.000117	0.007908	chr17	64300729
cg15425280	0.091393	3.895598	0.000122	0.008142	chr4	158141492
cg20342105	-0.08646	-3.89134	0.000124	0.008219	chr11	62474910
cg03058660	0.212675	3.885106	0.000127	0.00834	chr19	48919198
cg24753760	0.133827	3.882763	0.000128	0.008397	chr6	101846767
cg24146288	-0.13012	-3.85686	0.000142	0.009008	chr15	42302342
cg23332732	-0.13692	-3.84759	0.000147	0.009223	chr12	26986274
cg03923850	-0.08715	-3.84036	0.000151	0.009396	chr12	2372169
cg14082127	0.120201	3.837145	0.000153	0.009477	chr6	101847349
cg20684528	0.114701	3.829942	0.000158	0.009617	chr12	14133667
cg12934382	0.232374	3.8147	0.000167	0.009979	chr3	51741135
cg04743945	0.100554	3.775751	0.000194	0.010894	chr7	86273058
cg17627654	0.168858	3.772099	0.000197	0.011007	chr11	70508410
cg20231694	0.124262	3.766219	0.000201	0.011134	chr11	70691944
cg06421614	0.09265	3.760169	0.000206	0.011298	chr17	7121116
cg26459372	0.146491	3.755187	0.00021	0.011437	chr7	45613676
cg19237476	-0.06808	-3.75183	0.000213	0.011523	chr11	70501603
cg19809667	0.111077	3.750011	0.000214	0.011562	chr19	54401945
cg23715749	-0.11967	-3.74531	0.000218	0.011688	chr1	37413867
cg12463346	-0.19212	-3.72896	0.000232	0.012156	chr4	102268854
cg14919164	-0.14732	-3.69274	0.000266	0.013187	chr7	45729156
cg01207684	-0.19138	-3.68085	0.000278	0.013601	chr16	4103167
cg20325479	-0.13567	-3.67833	0.00028	0.013678	chr7	100271106
cg22860367	-0.06574	-3.67355	0.000285	0.013823	chr20	57426538
cg22491927	0.214303	3.658207	0.000302	0.014364	chr19	13617091
cg03451029	0.164503	3.632166	0.000333	0.015255	chr7	79764387
cg22231400	0.153945	3.624374	0.000343	0.01554	chr19	49935823
cg02332525	0.100995	3.620625	0.000348	0.015701	chr3	6903153
cg21187352	0.10549	3.614118	0.000356	0.015906	chr3	6903327
cg24213507	0.143327	3.610956	0.00036	0.016037	chr3	6902689
cg02071600	0.111293	3.585655	0.000395	0.017054	chr5	78808852
cg07950000	0.094734	3.576062	0.000409	0.017476	chr21	31312333
cg00699993	0.21634	3.573491	0.000413	0.01758	chr4	158141570
cg24676071	0.17314	3.567746	0.000422	0.017817	chr7	45613410
cg05309239	-0.0596	-3.55786	0.000438	0.018231	chr20	57427017
cg22597733	0.124391	3.542408	0.000463	0.018909	chr4	158142891
cg05488168	-0.0963	-3.51078	0.000519	0.020323	chr19	13400637
cg04837533	0.12791	3.510426	0.00052	0.020346	chr12	26986864
cg21938532	-0.07122	-3.50519	0.000529	0.020613	chr20	57426931
cg07561162	0.086929	3.496951	0.000545	0.021019	chr16	10174417
cg00348762	0.124885	3.490557	0.000558	0.021328	chr16	56228114
cg01663725	0.102833	3.447621	0.000651	0.023471	chr12	14133829
cg00392377	0.09976	3.438653	0.000672	0.023987	chr19	49939882

cg23123694	-0.11441	-3.43657	0.000677	0.024121	chr12	46766543
cg14859460	0.103352	3.428415	0.000696	0.024567	chr5	178422244
cg20877313	0.09724	3.419811	0.000718	0.025012	chr12	56881753
cg19385628	0.107789	3.419352	0.000719	0.025028	chr3	6904640
cg25638611	0.2162	3.418851	0.00072	0.025056	chr11	70508420
cg21816539	0.109476	3.407223	0.00075	0.025732	chr21	31312328
cg27304369	-0.1387	-3.40255	0.000763	0.026	chr20	57427483
cg00350942	0.106844	3.402478	0.000763	0.026003	chr9	140034073
cg15603568	0.112752	3.394206	0.000786	0.026477	chr11	105481283
cg22849059	0.126788	3.38678	0.000806	0.026921	chr12	56882693
cg05031016	0.103596	3.384873	0.000812	0.027032	chr14	24804153
cg09147777	0.094943	3.381565	0.000821	0.027184	chr11	105480771
cg19063061	0.189841	3.362065	0.000879	0.028398	chr19	49935893
cg26332560	0.131833	3.356732	0.000895	0.028767	chr8	132052887
cg09465746	0.15415	3.351377	0.000912	0.029097	chr3	6904386
cg19512268	0.12169	3.339318	0.000951	0.029899	chr6	146348901
cg07309764	0.122198	3.332281	0.000975	0.030345	chr7	79763914
cg17509967	0.124933	3.32673	0.000994	0.030664	chr19	13617094
cg07100700	-0.06947	-3.2643	0.001231	0.035059	chr6	34059787
cg07166235	0.089276	3.262973	0.001236	0.035138	chr12	49183018
cg04510788	0.128271	3.260242	0.001248	0.035341	chr1	37498900
cg10341242	0.06658	3.256424	0.001264	0.035692	chr16	50347849
cg06247406	0.093449	3.255456	0.001268	0.035773	chr6	101846791
cg14396328	-0.1427	-3.25332	0.001277	0.035953	chr7	45761429
cg04583232	0.068654	3.245433	0.001312	0.036613	chr11	22362874
cg12281620	-0.10167	-3.24014	0.001336	0.037017	chr19	2525290
cg09106984	-0.07303	-3.23744	0.001348	0.037203	chr6	34004360
cg16548911	0.087427	3.232511	0.001371	0.037586	chr16	50347766
cg27178677	-0.09583	-3.22633	0.0014	0.038081	chr20	8834803
cg14560133	0.087183	3.218741	0.001436	0.038649	chr19	51199453
cg10083824	-0.10459	-3.20038	0.001527	0.040193	chr6	34102147
cg08292023	0.088026	3.183243	0.001617	0.041651	chr11	88242253
cg11435826	0.063886	3.171348	0.001682	0.04263	chr12	2792111
cg06722633	0.098937	3.167225	0.001706	0.04301	chr1	37499309
cg01281157	0.115653	3.159902	0.001747	0.043718	chr5	178422260
cg06439941	-0.10469	-3.14102	0.00186	0.045538	chr7	93550756
cg24693368	0.085272	3.123046	0.001973	0.047228	chr2	155554844
cg12778476	0.122031	3.121946	0.001981	0.047334	chr11	22359345
cg19755318	0.095553	3.121475	0.001984	0.047383	chr3	50243323
cg10590857	0.116676	3.110181	0.002058	0.048433	chr5	7397021
cg17742947	-0.07657	-3.11015	0.002059	0.048433	chr19	42546977
cg13701180	0.083266	3.105568	0.00209	0.048906	chr19	2513436
cg17867243	0.168286	3.092486	0.002181	0.050256	chr15	42371653
cg22374237	0.089893	3.091708	0.002186	0.05034	chr7	126891197
cg12211091	-0.10379	-3.07727	0.002292	0.051857	chr19	2512999
cg19343464	0.089982	3.062747	0.002402	0.053395	chr11	105481509
cg07312654	0.087601	3.059187	0.00243	0.053713	chr8	132053773
cg12754421	0.094512	3.055467	0.002459	0.05409	chr11	105480790

cg14208013	-0.08441	-3.05159	0.00249	0.054503	chr20	57485765
cg18391758	0.078342	3.047321	0.002525	0.054992	chr16	10274963
cg00664406	0.121846	3.043051	0.00256	0.055467	chr3	51740875
cg20172500	0.091063	3.038446	0.002598	0.055934	chr3	6902432
cg10583180	0.114367	3.031202	0.002659	0.056804	chr6	101851354
cg00518386	0.111674	3.025635	0.002707	0.05745	chr16	10276984
cg18193094	0.107843	3.025385	0.002709	0.057488	chr6	101846905
cg11284196	0.068002	3.016218	0.00279	0.058519	chr19	51190047
cg15639581	0.067582	3.00743	0.002869	0.05953	chr19	13318873
cg27335600	0.07373	3.001764	0.002921	0.060236	chr3	53528857
cg06324048	-0.09617	-2.99869	0.00295	0.060596	chr20	57427103
cg15228639	-0.05984	-2.99865	0.00295	0.060596	chr15	42289939
cg09408768	0.093576	2.984133	0.003089	0.062286	chr2	155555053
cg16677144	0.07194	2.978846	0.003141	0.06291	chr1	1790217
cg03485674	0.060767	2.973395	0.003196	0.063568	chr16	50347895
cg23475371	0.082693	2.971985	0.00321	0.063769	chr21	31311842
cg04509024	-0.06646	-2.97011	0.003229	0.064031	chr1	37270540
cg17642145	-0.12501	-2.96921	0.003239	0.064142	chr8	22298578
cg16850173	0.148616	2.954591	0.003391	0.065922	chr9	140033611
cg13213810	0.0952	2.950626	0.003434	0.066362	chr22	51158720
cg20787196	0.090515	2.948795	0.003454	0.066601	chr12	26987031
cg14724613	0.072987	2.947339	0.003469	0.066789	chr7	86273429
cg04955246	-0.0453	-2.94242	0.003523	0.067488	chr17	64720790
cg20090108	0.198739	2.9416	0.003532	0.067557	chr11	88242488
cg07417708	0.069687	2.938443	0.003567	0.067948	chr5	78809348
cg06922606	0.073278	2.937277	0.00358	0.068064	chr16	10274632
cg14082123	0.072367	2.935446	0.003601	0.068305	chr15	42367977
cg04396791	0.226204	2.929895	0.003664	0.069072	chr11	70508180
cg18761756	-0.08029	-2.9261	0.003708	0.069501	chr18	3732002
cg08997253	0.108825	2.922531	0.003749	0.069986	chr9	104500729
cg22941646	0.070331	2.907291	0.003931	0.072048	chr1	235814339
cg15129608	0.086787	2.901691	0.004	0.072849	chr19	54393153
cg03663215	0.112438	2.901424	0.004003	0.07288	chr5	7396491
cg03091752	0.106089	2.900413	0.004016	0.073048	chr19	51221605
cg06065549	-0.08109	-2.90009	0.00402	0.073094	chr20	57427443
cg07192048	0.135135	2.898239	0.004043	0.073352	chr11	70560211
cg08870587	0.109886	2.890815	0.004137	0.074398	chr11	70455278
cg26337841	-0.07	-2.88839	0.004168	0.074755	chr1	37329331
cg27418217	0.056681	2.87604	0.00433	0.076655	chr15	83518427
cg25556841	-0.07579	-2.86825	0.004435	0.077733	chr19	2511263
cg12265829	0.078756	2.861569	0.004527	0.078691	chr14	24804022
cg20645074	0.094996	2.847567	0.004725	0.080758	chr12	49182479
cg22891619	0.137629	2.844314	0.004772	0.081201	chr17	72839038
cg15338449	0.081827	2.843888	0.004778	0.081272	chr15	83620910
cg24778538	-0.06509	-2.83941	0.004844	0.081932	chr12	14131975
cg26811638	-0.10615	-2.83868	0.004855	0.082057	chr20	57427493
cg15331781	0.071777	2.834936	0.00491	0.082647	chr7	86274443
cg21779904	-0.05502	-2.8301	0.004983	0.083453	chr20	57425157

cg24301620	0.084877	2.828499	0.005007	0.083726	chr6	101846872
cg27092975	-0.05077	-2.81421	0.005229	0.085846	chr11	70805455
cg17867333	0.11749	2.81248	0.005256	0.086145	chr5	178423163
cg20915897	0.064018	2.785195	0.005707	0.090496	chr7	45717588
cg14653281	0.110394	2.778981	0.005814	0.091471	chr9	104500954
cg17450585	-0.05768	-2.77168	0.005943	0.092637	chr6	34059718
cg11844965	-0.06848	-2.77159	0.005944	0.092641	chr19	42510093
cg02591871	-0.15732	-2.75025	0.006335	0.096377	chr19	14228565
cg03764381	0.091882	2.745589	0.006423	0.097182	chr16	10275410
cg13914083	0.104516	2.735146	0.006625	0.099098	chr4	158141526
cg23559689	0.088687	2.720855	0.00691	0.101726	chr11	105481292
cg13861294	-0.08916	-2.71804	0.006968	0.102178	chr12	100750473
cg10806318	-0.08345	-2.71522	0.007026	0.102711	chr11	70374297
cg21217024	0.089475	2.707686	0.007183	0.104187	chr11	105481406
cg06828043	-0.06672	-2.70476	0.007245	0.104755	chr11	70398862
cg07254421	0.074257	2.702031	0.007303	0.105245	chr5	36657993
cg08480458	0.079908	2.699457	0.007359	0.105727	chr3	53529655
cg14123942	0.141962	2.699273	0.007363	0.10575	chr9	104500322
cg11306735	-0.08779	-2.68185	0.007747	0.108912	chr19	49943812
cg24646457	0.073243	2.675218	0.007898	0.110049	chr15	42360292
cg12496211	-0.07419	-2.67383	0.00793	0.110303	chr12	2193060
cg08038054	-0.08637	-2.67177	0.007978	0.110689	chr7	93550781
cg18522549	-0.1354	-2.67049	0.008008	0.110907	chr11	62473861
cg08236022	0.06767	2.67045	0.008009	0.110916	chr7	93551014
cg02647408	0.091958	2.66784	0.008069	0.111409	chr11	88241594
cg22989942	-0.04032	-2.64861	0.008531	0.115105	chr20	57426950
cg10748817	-0.06572	-2.647	0.008571	0.115485	chr20	57465175
cg14350337	-0.09277	-2.64335	0.008662	0.116247	chr9	140060064
cg10546626	-0.06057	-2.64269	0.008678	0.116382	chr20	57424521
cg05794931	0.108619	2.641889	0.008698	0.116554	chr11	70493931
cg04002822	0.079514	2.634158	0.008894	0.118088	chr12	2483094
cg27271486	0.132338	2.632427	0.008938	0.11836	chr16	56227690
cg00601648	-0.07853	-2.63209	0.008947	0.118423	chr8	131974587
cg23019936	0.116569	2.628511	0.009039	0.119115	chr12	13903266
cg04907257	0.08828	2.628212	0.009047	0.119158	chr5	7395318
cg24319825	0.181373	2.621487	0.009223	0.120563	chr3	4534939
cg09190579	0.140641	2.616813	0.009347	0.121455	chr7	79763888
cg18224653	-0.04721	-2.61322	0.009444	0.122255	chr20	57426979
cg20439283	-0.05555	-2.61043	0.009519	0.122874	chr5	178411249
cg16920001	0.076181	2.604747	0.009675	0.124049	chr15	42448760
cg02232377	0.095957	2.599808	0.009812	0.125129	chr7	86297084
cg16644457	0.095511	2.59247	0.010019	0.126789	chr11	22359480
cg02615582	-0.07702	-2.59154	0.010045	0.126987	chr19	49939549
cg16761581	0.092529	2.580559	0.010363	0.129245	chr14	24803807
cg08587534	-0.0668	-2.57349	0.010572	0.130726	chr20	57427503
cg07105596	-0.14425	-2.57119	0.01064	0.131238	chr20	57427472
cg12102973	0.086191	2.571147	0.010642	0.131244	chr19	13320368
cg17868128	-0.0822	-2.56548	0.010813	0.132361	chr17	64383859

cg20640499	0.074828	2.56353	0.010873	0.13276	chr6	101850881
cg04498418	0.081579	2.553187	0.011193	0.13506	chr6	101850540
cg26472511	-0.06674	-2.54669	0.011399	0.136626	chr16	4034240
cg10123654	-0.0714	-2.53569	0.011755	0.139195	chr16	4162541
cg07190947	-0.05995	-2.53424	0.011803	0.139509	chr11	70349808
cg24098927	0.068543	2.533328	0.011833	0.139703	chr7	86273180
cg04269530	0.090311	2.528805	0.011983	0.140696	chr19	48919401
cg05793288	-0.05588	-2.52837	0.011998	0.140793	chr15	42375586
cg20875807	0.057833	2.519621	0.012293	0.142758	chr15	83620951
cg05397010	0.096879	2.516794	0.01239	0.143435	chr15	42448259
cg03437186	0.065863	2.516143	0.012412	0.143603	chr7	45614848
cg24037166	-0.08922	-2.51157	0.012571	0.14468	chr10	75255724
cg02984142	-0.15817	-2.51041	0.012611	0.144948	chr1	84971681
cg04209460	-0.0704	-2.5076	0.01271	0.145575	chr17	4711018
cg16833551	-0.09397	-2.50168	0.012919	0.147019	chr20	57427237
cg23310850	0.08761	2.501344	0.012931	0.147088	chr19	19051337
cg13724160	0.131112	2.500791	0.012951	0.147207	chr9	104500958
cg16135716	0.149532	2.49847	0.013034	0.147788	chr12	14133887
cg15674997	0.066071	2.492619	0.013246	0.149109	chr5	178421314
cg01103812	-0.11286	-2.49229	0.013258	0.149173	chr12	26986269
cg02780849	0.070923	2.488971	0.01338	0.149991	chr1	235814163
cg14526297	0.073131	2.483737	0.013574	0.151204	chr6	146349488
cg00287829	0.069932	2.482142	0.013634	0.151489	chr16	10276375
cg13861180	-0.06242	-2.46627	0.01424	0.155368	chr9	140060268
cg25534294	0.1084	2.463823	0.014335	0.155979	chr2	155554931
cg02837591	0.077689	2.459529	0.014504	0.156961	chr6	101850261
cg19657814	0.106297	2.455757	0.014654	0.157965	chr19	47137444
cg22758916	-0.05196	-2.45147	0.014826	0.159078	chr15	42303292
cg05627987	0.075935	2.44648	0.015028	0.160296	chr19	51220286
cg00050938	0.070315	2.442013	0.015211	0.161458	chr3	50296100
cg21245981	0.066715	2.440235	0.015285	0.161932	chr5	36607390
cg03330642	0.039056	2.440178	0.015287	0.161942	chr5	178411124
cg09096555	0.098848	2.435687	0.015474	0.163093	chr17	72848358
cg10409919	0.10125	2.435132	0.015498	0.163199	chr3	53530016
cg03357547	0.096291	2.435106	0.015499	0.163199	chr19	51165207
cg16815991	0.094084	2.433716	0.015557	0.163543	chr12	14133129
cg11921270	0.06975	2.433392	0.015571	0.163616	chr19	19051154
cg16696270	0.091423	2.425416	0.01591	0.165689	chr8	132052934
cg05659265	0.067826	2.416726	0.016287	0.167799	chr16	56225846
cg23165500	-0.04976	-2.41438	0.01639	0.168447	chr5	152949095
cg03321319	-0.08653	-2.41361	0.016424	0.168633	chr22	22222323
cg04202736	0.11388	2.411405	0.016521	0.169226	chr15	42371721
cg22956310	-0.0903	-2.40989	0.016589	0.169627	chr2	25142878
cg15620385	-0.08211	-2.40758	0.016692	0.170263	chr12	26986279
cg09641955	0.052766	2.406797	0.016727	0.170446	chr12	2716844
cg19293162	-0.0516	-2.40639	0.016745	0.170528	chr19	2513302
cg00296378	0.064845	2.403294	0.016885	0.171334	chr12	49177153
cg19700341	0.09277	2.394394	0.017292	0.173621	chr17	72857076

cg05780228	0.079583	2.39181	0.017411	0.174288	chr11	70713608
cg09659734	0.065501	2.386687	0.017651	0.175572	chr16	56227065
cg17826344	0.080177	2.386336	0.017668	0.175682	chr19	51169660
cg23077606	-0.05109	-2.38219	0.017864	0.1767	chr11	70540206
cg04521626	0.047877	2.381245	0.017909	0.176962	chr17	4714200
cg01509809	-0.05124	-2.3812	0.017911	0.176971	chr17	47284233
cg00980784	0.056597	2.37855	0.018037	0.177641	chr17	47287577
cg21793437	0.057813	2.372481	0.018331	0.179255	chr12	2734591
cg24454829	0.067418	2.3716	0.018373	0.179503	chr11	22363053
cg12377578	0.052188	2.369974	0.018453	0.179931	chr17	72856181
cg02807849	-0.04946	-2.36777	0.018561	0.180569	chr19	48908102
cg18799510	0.078938	2.367152	0.018591	0.180743	chr9	104499700
cg14291291	-0.06822	-2.36564	0.018666	0.181081	chr6	33656083
cg08436756	0.078294	2.352444	0.019328	0.184661	chr11	70781118
cg09662638	-0.05654	-2.34566	0.019677	0.1866	chr3	53795946
cg14121185	-0.06236	-2.34089	0.019925	0.187937	chr17	64488849
cg01962826	0.164503	2.337222	0.020118	0.189009	chr6	34100967
cg07601741	-0.2112	-2.33271	0.020357	0.190113	chr5	153160425
cg19640589	-0.10315	-2.32965	0.020521	0.190962	chr20	57427973
cg25418001	-0.10638	-2.32632	0.0207	0.191962	chr7	79780310
cg26229990	-0.10149	-2.31799	0.021155	0.194356	chr14	24801301
cg27222147	-0.05122	-2.3163	0.021249	0.19475	chr12	2224755
cg10262891	-0.04902	-2.31549	0.021294	0.194973	chr19	48904928
cg00116766	-0.07499	-2.31507	0.021317	0.195073	chr16	9857614
cg12682032	-0.04564	-2.31455	0.021346	0.1952	chr15	83617937
cg16468914	0.171475	2.312406	0.021465	0.195818	chr3	50242735
cg06223539	0.135754	2.312244	0.021474	0.195854	chr11	70517374
cg18707858	-0.0528	-2.31084	0.021553	0.196222	chr19	13366101
cg03149432	0.068702	2.309816	0.02161	0.196505	chr1	37498721
cg08218799	-0.04557	-2.30929	0.02164	0.196665	chr14	24804930
cg25556008	-0.07261	-2.30887	0.021664	0.196753	chr19	2525384
cg27369423	0.056826	2.307686	0.02173	0.197032	chr16	56228901
cg02693486	-0.04856	-2.30756	0.021738	0.197066	chr11	64030862
cg16919771	0.049239	2.30578	0.021838	0.197627	chr7	126892599
cg14287235	0.100468	2.30333	0.021978	0.198396	chr14	24804339
cg26452915	0.051594	2.30267	0.022015	0.198591	chr20	57486076
cg09305491	-0.06178	-2.28848	0.022839	0.202461	chr16	24151191
cg11439695	-0.0466	-2.28429	0.023087	0.203735	chr12	2561024
cg13344206	0.069124	2.284129	0.023097	0.203767	chr6	34051016
cg04336164	-0.06983	-2.28205	0.023221	0.204291	chr2	68478630
cg01871907	0.082519	2.278208	0.023452	0.205477	chr19	2703055
cg25733708	0.065886	2.277892	0.023471	0.205549	chr19	54393220
cg09262171	0.058396	2.270105	0.023945	0.20799	chr16	4140361
cg15333818	-0.06869	-2.26451	0.024291	0.209572	chr12	46766724
cg16009558	0.094343	2.2607	0.024529	0.210784	chr6	101846707
cg08875948	0.063853	2.259757	0.024589	0.211051	chr6	146755900
cg20998200	-0.0669	-2.25818	0.024688	0.211504	chr11	22370099
cg26419941	0.075952	2.256306	0.024807	0.212064	chr12	49183133

cg14574037	0.065931	2.255879	0.024834	0.212166	chr2	155553986
cg03882437	0.081846	2.251938	0.025085	0.213472	chr9	140051729
cg02140020	-0.05066	-2.24721	0.025389	0.214759	chr12	2613914
cg14285012	-0.0676	-2.24563	0.025491	0.215181	chr9	104357967
cg24539500	-0.07753	-2.24447	0.025567	0.215518	chr6	102115051
cg01245966	0.059265	2.244408	0.025571	0.215528	chr8	132053823
cg21895324	-0.04532	-2.24429	0.025579	0.215554	chr11	35385435
cg23522194	0.21584	2.24203	0.025726	0.216195	chr19	48565189
cg14679587	0.078593	2.239048	0.025922	0.216981	chr12	56882324
cg03578926	0.110577	2.238137	0.025982	0.217241	chr11	70508032
cg12887832	-0.05276	-2.23804	0.025988	0.217271	chr11	70805627
cg13740815	0.076291	2.237608	0.026017	0.2174	chr19	51170356
cg03945800	0.073407	2.236144	0.026114	0.217855	chr16	4165515
cg03645007	-0.04919	-2.23501	0.026189	0.218211	chr3	50255295
cg27182923	-0.03776	-2.23412	0.026248	0.218489	chr3	123129387
cg11116429	0.077605	2.227114	0.026718	0.220488	chr5	36607417
cg13759674	0.194542	2.225554	0.026824	0.220967	chr9	140051205
cg19636672	-0.07238	-2.21671	0.02743	0.223678	chr12	56881121
cg04453050	0.065539	2.213122	0.027679	0.224696	chr3	51740896
cg10529401	-0.04293	-2.21251	0.027722	0.224868	chr1	53587046
cg08747970	-0.05113	-2.20808	0.028032	0.226141	chr11	70666452
cg06869158	-0.03632	-2.20701	0.028108	0.226462	chr19	48900058
cg00834536	0.069849	2.201869	0.028473	0.228019	chr16	4013537
cg14149552	0.058277	2.20151	0.028498	0.228111	chr9	80642464
cg01427575	0.091594	2.200767	0.028552	0.228389	chr19	51171712
cg12246156	0.055329	2.198386	0.028723	0.229187	chr17	64522604
cg02946850	-0.07106	-2.19761	0.028778	0.229442	chr7	126882944
cg19646112	0.069031	2.194666	0.028991	0.230386	chr14	24804342
cg01806181	-0.05717	-2.19414	0.029029	0.230549	chr19	1007843
cg10013716	0.068955	2.193025	0.029111	0.230806	chr3	179168760
cg04695635	-0.04462	-2.1908	0.029273	0.231455	chr19	42510823
cg13907146	0.063804	2.190172	0.029319	0.231668	chr3	50243565
cg17483510	0.076794	2.185708	0.029648	0.23299	chr3	179168677
cg13756965	0.106078	2.182763	0.029866	0.23381	chr11	70917458
cg23475725	0.053322	2.1799	0.03008	0.234779	chr12	2734205
cg15815156	-0.08715	-2.17147	0.030718	0.23755	chr15	52472201
cg14960282	0.061163	2.168492	0.030945	0.238525	chr1	37321669
cg15706539	-0.0447	-2.16565	0.031164	0.239472	chr11	70924914
cg03856723	0.053182	2.164039	0.031289	0.240025	chr19	14229466
cg03601797	-0.05606	-2.15398	0.032077	0.243111	chr2	155556321
cg05765011	-0.0529	-2.15388	0.032085	0.243155	chr16	4103225
cg16739976	0.067636	2.137665	0.033392	0.248517	chr6	34101401
cg14120436	0.065087	2.133806	0.033709	0.249779	chr15	52483498
cg04156464	0.08094	2.132005	0.033859	0.250359	chr16	56224504
cg06193383	0.06214	2.129526	0.034065	0.251147	chr16	10275767
cg00202454	0.13069	2.124625	0.034475	0.252721	chr15	42371886
cg05846851	-0.05465	-2.12189	0.034707	0.253669	chr16	10172054
cg07700514	0.131835	2.120916	0.034789	0.254022	chr16	56228467

cg17483139	0.060935	2.120289	0.034843	0.254263	chr1	1822401
cg01286319	-0.05459	-2.11878	0.034971	0.254691	chr19	2695343
cg26495109	0.058306	2.11874	0.034974	0.254691	chr16	56225952
cg11855555	0.066664	2.117926	0.035044	0.254982	chr1	68232134
cg00720707	-0.05343	-2.11675	0.035145	0.255438	chr16	4016845
cg16693012	-0.04663	-2.11542	0.035259	0.25592	chr1	68283821
cg07735790	-0.05751	-2.11248	0.035512	0.256897	chr6	34101545
cg04903912	-0.05103	-2.11204	0.03555	0.257052	chr11	70374289
cg02218200	-0.04749	-2.1086	0.035849	0.258217	chr22	51135138
cg27643147	0.074945	2.108172	0.035886	0.258367	chr11	70517294
cg16312514	-0.07718	-2.10767	0.035929	0.258482	chr11	70650521
cg23696752	0.090854	2.104729	0.036187	0.259341	chr6	146349312
cg12771777	0.092113	2.10411	0.036242	0.259484	chr11	70692038
cg25451120	0.046406	2.100079	0.036598	0.2609	chr17	47287444
cg26640467	0.057057	2.098567	0.036732	0.26142	chr7	126893304
cg13878010	0.057003	2.097585	0.036819	0.261794	chr3	123167276
cg04119977	-0.05661	-2.09593	0.036967	0.262376	chr5	7826972
cg23911372	0.081553	2.087705	0.037709	0.265114	chr19	42546731
cg10998242	-0.05091	-2.08512	0.037945	0.26595	chr3	53530847
cg09583957	-0.08632	-2.08348	0.038095	0.266558	chr20	57428315
cg20979061	0.068801	2.08331	0.038111	0.266586	chr19	49939949
cg00140112	-0.04442	-2.08213	0.038219	0.267035	chr18	3879595
cg13203394	0.045436	2.080038	0.038412	0.267789	chr12	26951217
cg09405076	-0.04255	-2.08003	0.038413	0.267789	chr11	70477139
cg04023483	0.064447	2.077802	0.03862	0.26861	chr3	6904134
cg04103918	-0.04882	-2.07671	0.038721	0.268933	chr17	4726687
cg18753811	-0.07969	-2.07641	0.038749	0.269031	chr12	2162232
cg14957718	0.111822	2.071658	0.039194	0.270538	chr3	50243260
cg06954761	0.065655	2.06483	0.03984	0.272998	chr6	33601863
cg18842187	-0.03072	-2.06313	0.040002	0.273523	chr6	33647826
cg07748847	0.083821	2.061978	0.040113	0.27392	chr12	14124857
cg20315739	-0.04878	-2.06019	0.040285	0.274608	chr15	42303032
cg00587834	0.079591	2.052798	0.041002	0.277171	chr3	51749959
cg00007326	-0.05689	-2.05122	0.041157	0.277745	chr19	13366046
cg17885091	0.104921	2.045872	0.041683	0.279606	chr1	68299057
cg25381331	-0.08273	-2.04224	0.042045	0.280881	chr1	53556414
cg24849633	-0.02913	-2.04182	0.042086	0.280996	chr22	51142900
cg22689690	0.065982	2.040742	0.042195	0.28136	chr12	49183468
cg00426968	0.05806	2.034771	0.042796	0.283401	chr19	47138284
cg07793724	-0.1207	-2.03086	0.043194	0.28485	chr1	53609371
cg05736079	-0.16314	-2.03031	0.043251	0.285059	chr9	140063205
cg14788049	0.069455	2.029892	0.043293	0.285238	chr11	64018549
cg12452386	-0.04248	-2.02867	0.043418	0.2857	chr15	42302500
cg14241370	0.086376	2.024444	0.043854	0.287329	chr9	104499850
cg13523713	-0.07209	-2.02154	0.044155	0.288417	chr19	48614733
cg04077795	-0.0639	-2.01979	0.044338	0.289061	chr16	9857925
cg20790998	-0.04692	-2.01702	0.044628	0.289994	chr1	68290436
cg08894891	0.072595	2.015434	0.044794	0.290488	chr19	19040364

cg01352090	0.047047	2.014629	0.044879	0.290818	chr16	4103533
cg17952046	0.059281	2.009593	0.045413	0.292547	chr1	37379413
cg01577933	0.101572	2.009216	0.045454	0.292661	chr1	37498025
cg09635994	0.065854	2.006928	0.045698	0.293522	chr22	51171263
cg26430597	-0.03515	-2.00624	0.045772	0.293792	chr1	182354699
cg23815646	0.102678	2.005097	0.045895	0.294167	chr8	131961143
cg00792185	0.055359	2.002074	0.046221	0.295313	chr16	9855960
cg18760587	0.056157	2.000593	0.046381	0.295973	chr7	79764888
cg01980361	-0.0458	-1.99617	0.046863	0.297615	chr3	53843939
cg10613332	-0.0631	-1.99425	0.047074	0.298275	chr19	13368845
cg00848945	-0.09419	-1.99367	0.047137	0.298502	chr12	2800919
cg04527363	0.058985	1.991039	0.047428	0.299495	chr3	6902337
cg23734973	0.057844	1.990046	0.047538	0.299808	chr4	158141449
cg10468484	-0.03607	-1.98983	0.047562	0.299879	chr20	57431303
cg21392385	0.042157	1.988351	0.047726	0.300399	chr15	52428589
cg08550394	0.049626	1.980382	0.048619	0.30314	chr16	4013659
cg19234705	-0.04249	-1.97744	0.048951	0.304262	chr19	19042181
cg24634422	0.057463	1.975688	0.049151	0.304927	chr11	35441593
cg05824594	0.033277	1.974406	0.049297	0.305393	chr12	2734503
cg01017090	-0.03891	-1.97421	0.04932	0.305422	chr20	57427046
cg03100024	-0.05073	-1.97413	0.049329	0.305428	chr19	42571402
cg23264278	0.07837	1.971778	0.049598	0.306318	chr19	19051482
cg24643102	0.063707	1.970084	0.049793	0.306965	chr3	6903921
cg23816431	0.064098	1.968926	0.049927	0.307261	chr3	171524969
cg15765694	0.043832	1.958471	0.051147	0.311188	chr9	104358101
cg10901633	0.035645	1.957963	0.051206	0.311422	chr3	4814459
cg25217317	0.055235	1.954636	0.0516	0.312499	chr1	235811994
cg10917153	0.033535	1.954343	0.051635	0.312585	chr15	42448786
cg01397507	0.055602	1.953711	0.05171	0.312833	chr11	70440395
cg02380914	-0.04191	-1.95317	0.051774	0.313042	chr22	51143114
cg10425005	0.057436	1.952919	0.051804	0.313126	chr16	10133433
cg01014438	-0.07994	-1.95276	0.051824	0.313178	chr12	2762689
cg13384396	0.054122	1.94771	0.052428	0.314969	chr3	123167677
cg11836949	-0.11925	-1.94264	0.053042	0.316882	chr1	1812359
cg24339704	0.032552	1.937905	0.053619	0.318625	chr19	2529022
cg23153707	0.050983	1.936564	0.053784	0.319117	chr17	7121040
cg10410142	0.106942	1.935513	0.053913	0.319489	chr4	158141542
cg05366189	-0.06644	-1.93541	0.053925	0.319528	chr19	13397419
cg08861556	-0.07923	-1.93353	0.054158	0.320243	chr16	850614
cg21072025	0.052363	1.932274	0.054313	0.320744	chr19	47137863
cg08021532	-0.12154	-1.92669	0.055009	0.323026	chr16	50321878
cg21844450	0.054318	1.914192	0.056593	0.327405	chr20	8112956
cg18920858	-0.0644	-1.91271	0.056784	0.327923	chr17	64617748
cg23797439	0.057302	1.911105	0.056991	0.328533	chr20	8113355
cg01231108	0.066068	1.908405	0.05734	0.329346	chr2	155556016
cg13411554	-0.14327	-1.90815	0.057373	0.329438	chr3	53700276
cg06836849	0.058838	1.907741	0.057426	0.329623	chr12	100751051
cg03010274	-0.04404	-1.90702	0.057519	0.329882	chr20	57427274

cg03140521	0.055783	1.904606	0.057834	0.33076	chr1	68299388
cg23566401	0.057355	1.901251	0.058274	0.332164	chr17	7120484
cg01542384	0.048837	1.901018	0.058305	0.332209	chr3	50284305
cg04711050	0.058686	1.899836	0.05846	0.332622	chr9	4490757
cg25316569	-0.07263	-1.89935	0.058524	0.332769	chr11	120530952
cg04016326	0.080648	1.898127	0.058686	0.333145	chr12	14132940
cg17006443	-0.0476	-1.89426	0.059199	0.33454	chr11	70628938
cg18982286	0.071021	1.891615	0.059552	0.335649	chr22	51136325
cg15852446	0.092555	1.89151	0.059567	0.335693	chr15	40583422
cg05329317	0.038132	1.889118	0.059888	0.336758	chr16	30126595
cg13878641	-0.05123	-1.88898	0.059907	0.336814	chr1	110090951
cg05806233	0.050032	1.887447	0.060113	0.337328	chr7	79763748
cg16399393	0.044976	1.88743	0.060115	0.337333	chr11	70368768
cg02771299	0.042539	1.884837	0.060466	0.338361	chr6	33609240
cg13510813	-0.04109	-1.88475	0.060477	0.338391	chr19	42571339
cg13515021	0.060385	1.879754	0.061158	0.340337	chr12	49177075
cg17641710	0.030204	1.878139	0.061379	0.340873	chr3	50279038
cg07064537	-0.03561	-1.87793	0.061409	0.340953	chr3	171515212
cg03836615	0.027794	1.872984	0.062091	0.342918	chr3	4856096
cg13523557	0.068478	1.872784	0.062118	0.342994	chr7	45613725
cg19589727	-0.06206	-1.86983	0.062529	0.344263	chr20	57427762
cg26555126	-0.04059	-1.86751	0.062853	0.345184	chr6	33998729
cg12647801	0.048565	1.867251	0.062889	0.345248	chr11	64028732
cg08066673	-0.04019	-1.86536	0.063154	0.345837	chr14	52325747
cg15827003	-0.09862	-1.85803	0.064192	0.348633	chr1	1822912
cg21635870	0.08549	1.857406	0.064281	0.348844	chr6	101847058
cg01081636	-0.03739	-1.85609	0.064469	0.34932	chr6	33994263
cg06844159	-0.0468	-1.85371	0.06481	0.350241	chr11	70374355
cg04378167	-0.08144	-1.85004	0.06534	0.351847	chr19	49944943
cg17020834	0.054686	1.848115	0.065618	0.352707	chr5	152870258
cg04270835	0.054107	1.846959	0.065786	0.353107	chr11	22359188
cg15164708	0.04949	1.843807	0.066246	0.354246	chr19	49936274
cg13971030	-0.11958	-1.84149	0.066585	0.355075	chr11	35366721
cg20917920	-0.04055	-1.84072	0.066698	0.355326	chr11	70416238
cg08170375	-0.05165	-1.8405	0.066731	0.355451	chr3	50247180
cg13455717	0.06139	1.838783	0.066984	0.356177	chr1	235814365
cg26983544	-0.05933	-1.83572	0.067438	0.357434	chr5	78784024
cg13722123	0.064946	1.834377	0.067637	0.357914	chr6	146350346
cg09248655	0.060165	1.832487	0.067919	0.358715	chr19	48897955
cg01780685	-0.03604	-1.8316	0.068051	0.358978	chr17	7099875
cg10671676	-0.04392	-1.83083	0.068166	0.359251	chr5	178413560
cg19621460	0.048281	1.830102	0.068275	0.359475	chr19	14225945
cg03508063	-0.04041	-1.82846	0.068522	0.360143	chr17	7124385
cg16098545	0.05712	1.827766	0.068626	0.360443	chr12	26492312
cg05248742	0.058465	1.824692	0.069091	0.361592	chr16	24142386
cg07232612	0.041368	1.824641	0.069098	0.361613	chr7	93551012
cg09530407	0.053275	1.821424	0.069587	0.362837	chr11	22359486
cg15486374	-0.04513	-1.81845	0.070041	0.364066	chr15	83621710

cg19942459	0.104619	1.818329	0.07006	0.364089	chr11	70508110
cg13448720	-0.04278	-1.81589	0.070434	0.365098	chr9	140052246
cg17401938	-0.05415	-1.8157	0.070464	0.365143	chr12	2228442
cg17754876	0.051377	1.815242	0.070534	0.365256	chr11	35441260
cg06328100	-0.04414	-1.81394	0.070735	0.365818	chr6	33638806
cg02879453	0.050364	1.809857	0.071368	0.367566	chr16	50321818
cg06500096	-0.0521	-1.80889	0.071518	0.367824	chr19	13405582
cg00380835	-0.09368	-1.80872	0.071545	0.367827	chr19	51165752
cg04988514	0.104179	1.80627	0.071927	0.368946	chr19	48947560
cg10286380	0.051843	1.804461	0.072211	0.369616	chr19	51171847
cg01864982	0.052752	1.802594	0.072505	0.370427	chr19	54409631
cg15355952	-0.0804	-1.80145	0.072685	0.37087	chr5	36662829
cg10614021	0.047241	1.801238	0.072719	0.370926	chr12	14134486
cg26226650	0.031176	1.801167	0.07273	0.370958	chr3	50276265
cg04476846	-0.04652	-1.79975	0.072954	0.371547	chr5	78791415
cg05185634	-0.02477	-1.7971	0.073374	0.372542	chr14	24804750
cg08805338	-0.05049	-1.79563	0.073609	0.37308	chr10	75255936
cg22851944	0.050181	1.795105	0.073693	0.373309	chr6	101847388
cg14351692	-0.04806	-1.79427	0.073827	0.373621	chr12	13716374
cg20771332	-0.04427	-1.79162	0.07425	0.374646	chr11	70332620
cg15522719	-0.06057	-1.78777	0.074872	0.37613	chr2	191745248
cg01344243	0.073037	1.782889	0.075665	0.378209	chr16	9855280
cg11758458	0.037451	1.781054	0.075965	0.378904	chr17	64575021
cg27629673	-0.0404	-1.77932	0.076249	0.379665	chr5	7462856
cg23281712	-0.06261	-1.77538	0.076899	0.381099	chr2	25143391
cg04158792	-0.03781	-1.77358	0.077196	0.381828	chr19	2514622
cg12389770	0.05365	1.77161	0.077524	0.382685	chr6	101847706
cg24266105	0.087547	1.770059	0.077783	0.383208	chr6	146351044
cg02736560	-0.03323	-1.7695	0.077875	0.383363	chr1	68257754
cg02760164	0.07047	1.768367	0.078066	0.383867	chr15	42371967
cg14301531	-0.03823	-1.76832	0.078073	0.38388	chr12	2226907
cg21885159	0.046957	1.768224	0.078089	0.383895	chr19	2645923
cg23666299	-0.06949	-1.76506	0.07862	0.385197	chr6	102326919
cg11118235	0.037505	1.76381	0.078832	0.38563	chr3	50284010
cg24891846	0.151939	1.762463	0.079059	0.386143	chr19	13319523
cg16069986	-0.05815	-1.76073	0.079353	0.386934	chr11	70650456
cg01331810	0.068563	1.75885	0.079672	0.387506	chr7	86414302
cg14537332	0.12362	1.758085	0.079803	0.38777	chr11	70508113
cg11023668	0.089709	1.754164	0.080473	0.38949	chr2	25095040
cg08515427	0.040505	1.753958	0.080509	0.389569	chr16	848997
cg21006600	-0.06245	-1.7538	0.080535	0.389612	chr12	2342206
cg11010575	-0.04448	-1.75179	0.080881	0.390448	chr15	42129464
cg20668321	-0.04065	-1.75142	0.080946	0.390613	chr5	153192843
cg18420143	-0.04431	-1.74925	0.081321	0.391364	chr17	7123125
cg11658986	0.036135	1.743952	0.082242	0.393471	chr12	49177605
cg15014684	-0.03619	-1.74374	0.082279	0.393537	chr17	7093301
cg14116756	-0.10825	-1.74161	0.082651	0.394359	chr10	75255721
cg09902254	0.0553	1.738101	0.083269	0.395704	chr11	70858237

cg10362475	0.085	1.732753	0.084217	0.397768	chr11	70507825
cg02303571	0.046216	1.729515	0.084796	0.399138	chr5	36606769
cg26968025	0.029018	1.725697	0.085482	0.400711	chr17	64519943
cg20240931	-0.05374	-1.72131	0.086275	0.402479	chr19	2613894
cg20772037	-0.03618	-1.7185	0.086788	0.403639	chr6	34067242
cg12198934	-0.03954	-1.7157	0.0873	0.404623	chr17	64378035
cg16777106	-0.04426	-1.71475	0.087474	0.405081	chr4	158281194
cg01132471	-0.04272	-1.71246	0.087896	0.406053	chr1	53556482
cg14992273	-0.04349	-1.71087	0.08819	0.406711	chr1	37337827
cg24764979	0.048172	1.709891	0.088371	0.407062	chr16	10276600
cg22798121	-0.04227	-1.70989	0.088371	0.407062	chr5	152896564
cg03989758	-0.06497	-1.70809	0.088704	0.407735	chr5	36662950
cg14093720	-0.03517	-1.704	0.089466	0.40919	chr18	3712400
cg05339056	0.063672	1.703347	0.089589	0.409423	chr7	86391162
cg11640185	-0.03339	-1.7031	0.089636	0.409496	chr19	42509801
cg13353325	0.087538	1.699788	0.090258	0.410789	chr20	57485837
cg25751482	-0.06202	-1.69838	0.090523	0.411373	chr17	64301327
cg17658854	0.034721	1.698047	0.090587	0.41148	chr20	57462798
cg14019146	-0.05957	-1.69773	0.090646	0.411555	chr3	50243930
cg14967731	0.033644	1.697181	0.09075	0.411763	chr3	4748984
cg08161922	0.081992	1.696435	0.090892	0.412043	chr12	2163608
cg05511872	0.082692	1.69378	0.091396	0.413054	chr11	70824260
cg24151995	0.059169	1.692575	0.091625	0.41353	chr11	22364293
cg23868250	0.052879	1.692332	0.091672	0.413624	chr11	120764552
cg04440551	0.052882	1.692077	0.09172	0.413678	chr2	25051151
cg04586622	-0.04992	-1.69107	0.091913	0.414098	chr2	25135609
cg17191518	-0.04813	-1.6893	0.092251	0.414931	chr15	52468850
cg04929165	-0.03474	-1.68872	0.092362	0.415173	chr1	186807235
cg14003231	0.04376	1.686837	0.092725	0.415997	chr6	33640908
cg25988118	-0.0321	-1.68281	0.093504	0.417646	chr6	34101785
cg21725265	0.050081	1.682412	0.09358	0.41778	chr19	19051201
cg14815005	-0.12378	-1.67829	0.094383	0.419406	chr22	22222162
cg02775369	0.050577	1.678273	0.094386	0.419406	chr16	56316221
cg27340723	0.074497	1.673851	0.095253	0.421356	chr16	4061608
cg07716032	-0.04346	-1.67381	0.095261	0.421356	chr17	7122846
cg05410012	0.04644	1.67252	0.095515	0.421942	chr17	72857095
cg14212966	-0.0493	-1.67143	0.095731	0.422313	chr1	53558392
cg20152891	0.07286	1.670017	0.09601	0.422863	chr19	49944506
cg23427362	0.056464	1.668458	0.096319	0.423528	chr12	2335272
cg20484832	0.057342	1.664686	0.09707	0.425002	chr20	9075810
cg14010550	0.144722	1.662793	0.097449	0.425639	chr19	1009642
cg10482356	0.03283	1.661779	0.097653	0.426081	chr16	56328421
cg11895615	0.049434	1.660299	0.09795	0.426606	chr12	2224518
cg25649039	-0.06148	-1.66024	0.097962	0.426606	chr17	64335475
cg18287522	0.064119	1.658851	0.098242	0.427197	chr3	179115547
cg15425921	-0.06164	-1.65823	0.098368	0.427537	chr19	2643085
cg11801011	-0.0477	-1.65765	0.098485	0.427799	chr19	51220537
cg04569608	0.042348	1.656055	0.098807	0.428567	chr11	64018309

cg13555101	0.04713	1.654774	0.099067	0.429092	chr9	4490751
cg25000623	0.047475	1.654438	0.099135	0.429294	chr17	72848918
cg03830585	-0.06308	-1.6539	0.099244	0.429523	chr3	4536777
cg17125585	-0.03841	-1.65326	0.099375	0.429794	chr1	68176471
cg20910008	0.038684	1.653184	0.099391	0.429822	chr8	22301378
cg03016097	0.038735	1.652412	0.099548	0.430063	chr15	83621726
cg07456314	0.053703	1.652373	0.099556	0.430077	chr17	4708968
cg24445167	-0.0381	-1.65114	0.099808	0.430604	chr12	2383231
cg15417641	-0.08555	-1.64733	0.100588	0.43206	chr3	53700141
cg00521993	0.043583	1.64577	0.100909	0.432851	chr10	75199411
cg22090419	0.057616	1.645421	0.100981	0.433009	chr3	53844172
cg02540833	0.054278	1.637809	0.102561	0.436172	chr11	70562363
cg14090219	-0.04184	-1.63698	0.102735	0.436556	chr19	13347085
cg16916688	-0.05791	-1.63369	0.103424	0.437903	chr6	34101441
cg23580000	0.037475	1.632905	0.103589	0.438223	chr16	50322156
cg21899500	0.05794	1.630407	0.104116	0.439381	chr3	51740850
cg23068476	-0.04233	-1.62783	0.104662	0.440406	chr12	2613703
cg21201396	-0.0568	-1.62738	0.104757	0.440603	chr11	70665271
cg15418783	-0.04116	-1.62547	0.105164	0.441517	chr11	70557693
cg20528838	-0.05945	-1.62516	0.105229	0.441662	chr20	57427730
cg17839611	0.029673	1.624666	0.105335	0.441872	chr17	47286802
cg06840723	0.053819	1.624597	0.10535	0.441899	chr15	52484492
cg02624051	0.076172	1.622061	0.105892	0.442894	chr15	42371635
cg20518994	-0.04194	-1.61854	0.106649	0.444419	chr2	25141532
cg04149773	-0.02846	-1.61841	0.106677	0.444494	chr12	49179923
cg11264635	-0.04501	-1.61758	0.106856	0.44486	chr19	15083868
cg16753846	-0.0387	-1.61487	0.107441	0.445992	chr11	70318894
cg23464041	0.028238	1.614007	0.107629	0.446364	chr6	33663938
cg26496204	-0.03333	-1.61328	0.107787	0.446685	chr20	57427210
cg13469748	-0.06438	-1.61214	0.108035	0.447123	chr1	84971910
cg10271186	-0.03689	-1.61086	0.108314	0.447679	chr11	70908897
cg23676682	0.045539	1.610744	0.108339	0.447702	chr11	105480792
cg17298543	0.045604	1.606297	0.109312	0.449415	chr7	79802016
cg09772382	-0.01797	-1.60431	0.109749	0.450226	chr20	57463775
cg03827772	-0.03255	-1.60121	0.110433	0.451504	chr11	70912450
cg02676523	0.042607	1.601101	0.110459	0.451548	chr16	4027674
cg17540575	-0.02916	-1.60013	0.110674	0.451916	chr19	42504627
cg22323942	0.103806	1.599933	0.110718	0.451975	chr19	2543655
cg13574337	0.059606	1.598702	0.110991	0.452516	chr16	4016720
cg15335139	0.060773	1.596846	0.111404	0.453351	chr3	50242325
cg06489744	0.04924	1.596564	0.111467	0.453421	chr11	70557772
cg19242688	-0.08112	-1.59634	0.111517	0.45348	chr19	47139391
cg19006947	-0.05758	-1.59619	0.111551	0.45353	chr11	64034861
cg23491599	0.075301	1.594002	0.11204	0.454474	chr2	155554688
cg03731464	-0.03903	-1.59363	0.112123	0.454594	chr12	2801158
cg14616584	-0.04867	-1.59275	0.11232	0.454929	chr1	37388124
cg09336589	-0.04837	-1.5906	0.112803	0.455957	chr17	7107939
cg18384228	0.046872	1.59049	0.112829	0.456021	chr14	24789158

cg26536401	-0.03063	-1.59019	0.112897	0.456162	chr12	6956432
cg10774282	-0.03509	-1.58942	0.113071	0.456533	chr1	53608280
cg27333271	-0.0725	-1.58729	0.113553	0.457408	chr3	7268498
cg14150378	-0.04802	-1.58727	0.113556	0.457408	chr9	80335274
cg26199552	0.034729	1.586603	0.113707	0.457653	chr11	70368372
cg19296354	0.033335	1.585984	0.113848	0.457926	chr20	57415697
cg27014608	-0.05931	-1.58372	0.114363	0.458761	chr16	4166952
cg17221095	0.026597	1.579238	0.115387	0.460596	chr7	45717651
cg19930620	0.051826	1.578892	0.115466	0.460761	chr3	7340148
cg12012319	-0.03734	-1.57866	0.115519	0.460832	chr7	126675528
cg12345953	0.053804	1.578418	0.115575	0.460918	chr4	102207435
cg08860136	-0.02897	-1.57569	0.116202	0.462018	chr17	7111414
cg08779207	0.046438	1.575476	0.116252	0.462137	chr15	40586496
cg02110273	-0.03187	-1.575	0.116362	0.46237	chr17	64727684
cg22569496	-0.10248	-1.57466	0.11644	0.462528	chr19	13409671
cg15562780	0.042462	1.574304	0.116523	0.462703	chr11	35441311
cg15464481	-0.04038	-1.57365	0.116675	0.463003	chr3	123151962
cg03972076	-0.03339	-1.5732	0.116779	0.463219	chr11	64023183
cg16848624	0.076461	1.573062	0.11681	0.463277	chr7	45614290
cg11407328	0.033186	1.571908	0.117078	0.463691	chr17	64759926
cg14696064	-0.03516	-1.57145	0.117183	0.463872	chr3	123010055
cg01354782	-0.04263	-1.56972	0.117587	0.464559	chr12	26522758
cg00076497	0.048346	1.569557	0.117624	0.464614	chr7	126891621
cg03315058	-0.06161	-1.56949	0.11764	0.464614	chr11	62476542
cg24794531	0.041701	1.568808	0.117798	0.464931	chr3	142443837
cg21858376	0.037605	1.568687	0.117827	0.465006	chr3	4534791
cg14100184	0.049547	1.567152	0.118185	0.465653	chr16	851298
cg18457944	0.044423	1.565808	0.118499	0.466253	chr3	171428279
cg22023664	0.02384	1.565532	0.118564	0.466371	chr19	2619608
cg27451362	0.093376	1.562279	0.119328	0.467576	chr6	101846650
cg10815152	0.0653	1.562246	0.119336	0.467578	chr6	102098727
cg09576209	0.053031	1.561637	0.119479	0.467842	chr12	2339614
cg26739975	-0.07815	-1.56033	0.119788	0.468386	chr4	158144318
cg23398700	0.043985	1.559976	0.119871	0.468539	chr5	78808194
cg27270541	-0.06434	-1.55751	0.120456	0.469601	chr19	48614177
cg24159247	0.033482	1.555789	0.120864	0.470483	chr3	4575483
cg00943909	-0.05755	-1.55439	0.121196	0.47108	chr20	57427942
cg02399044	0.050953	1.554382	0.121199	0.471085	chr12	2500229
cg16620233	-0.0316	-1.55435	0.121207	0.471092	chr3	53781107
cg00390253	-0.03652	-1.55251	0.121647	0.471874	chr3	50241300
cg09558195	0.062025	1.551669	0.121847	0.472197	chr6	146350585
cg21734356	-0.03427	-1.55128	0.12194	0.472362	chr18	3498854
cg26350373	0.034894	1.551276	0.121941	0.472363	chr15	42449015
cg18150383	-0.05944	-1.54973	0.122313	0.472915	chr19	49933217
cg19999705	-0.03763	-1.54938	0.122396	0.473082	chr6	34026903
cg12535596	-0.04439	-1.54931	0.122412	0.47309	chr1	182360697
cg23848889	0.044938	1.547629	0.122817	0.473859	chr11	70455304
cg18399935	0.042133	1.544413	0.123594	0.475134	chr3	6906994

cg25001544	0.081919	1.544123	0.123664	0.475231	chr6	34073788
cg13231680	-0.0365	-1.54302	0.123932	0.475679	chr3	7693146
cg15021670	-0.03621	-1.5416	0.124278	0.476293	chr15	42386726
cg07571637	0.058701	1.541248	0.124362	0.476357	chr22	51143260
cg05850280	0.052601	1.54094	0.124437	0.476552	chr9	140056489
cg08478447	0.05168	1.540488	0.124547	0.476756	chr3	53528849
cg21615915	0.059355	1.54024	0.124608	0.476842	chr6	102295679
cg20920827	-0.0471	-1.53991	0.124689	0.476963	chr11	70858475
cg00426976	0.034061	1.537108	0.125372	0.478207	chr22	51140977
cg19885037	-0.03145	-1.53485	0.125926	0.479126	chr3	4762242
cg08310216	0.03782	1.534519	0.126007	0.479263	chr7	100271217
cg19033906	-0.03004	-1.53394	0.126151	0.47953	chr3	53532972
cg00082310	0.051961	1.533923	0.126154	0.479537	chr3	171528573
cg03335128	0.04871	1.533174	0.126338	0.479866	chr1	1731415
cg08258650	0.0425	1.533076	0.126362	0.479892	chr11	35441900
cg15884992	-0.03103	-1.53268	0.12646	0.48007	chr6	34028192
cg23965720	0.037881	1.532445	0.126518	0.480193	chr1	235805770
cg04933990	0.063647	1.532287	0.126557	0.480267	chr16	10133501
cg17334845	-0.02323	-1.53151	0.126749	0.480586	chr20	57463572
cg22290117	-0.03907	-1.53026	0.127058	0.48109	chr20	57427173
cg10573143	0.040632	1.530046	0.127111	0.481185	chr11	70628992
cg02063520	0.053066	1.528528	0.127487	0.481791	chr6	102516590
cg17047106	-0.06172	-1.52787	0.127651	0.482023	chr8	131961479
cg25521963	-0.05467	-1.52742	0.127762	0.482223	chr17	7123553
cg06441398	-0.03822	-1.52682	0.127911	0.482487	chr11	70317455
cg08886546	0.038701	1.521905	0.129138	0.48483	chr19	19051335
cg02847220	-0.05265	-1.5207	0.129439	0.485253	chr3	171523157
cg26503038	-0.03749	-1.51752	0.13024	0.486487	chr19	2699659
cg14116052	0.049778	1.516732	0.130439	0.486839	chr5	7596454
cg19471040	-0.0314	-1.51493	0.130894	0.487654	chr6	34031142
cg11601336	-0.02539	-1.5123	0.131563	0.488859	chr19	19053012
cg20557935	-0.02827	-1.511	0.131891	0.489479	chr15	52484322
cg09991710	-0.03083	-1.50971	0.132222	0.490047	chr1	37472559
cg20151098	-0.04132	-1.50879	0.132458	0.490392	chr6	33995991
cg08809418	0.046707	1.508154	0.132619	0.490611	chr12	56881865
cg03389717	-0.08961	-1.50721	0.132861	0.490995	chr6	33601269
cg07964163	0.039333	1.506261	0.133104	0.491418	chr20	57413417
cg14731698	0.06382	1.505717	0.133244	0.491606	chr11	120738292
cg00515755	-0.04703	-1.50479	0.133481	0.492051	chr19	1005248
cg03399271	0.041951	1.504159	0.133644	0.492354	chr16	56228385
cg00940140	-0.06922	-1.50281	0.13399	0.492941	chr20	57480494
cg07633435	0.036439	1.501656	0.134289	0.493395	chr5	152869009
cg13329789	-0.02653	-1.50026	0.13465	0.493962	chr7	45757644
cg09432792	-0.09972	-1.49877	0.135036	0.494561	chr16	56352311
cg17969540	0.072773	1.498139	0.1352	0.494822	chr19	48908179
cg01250212	0.044394	1.496387	0.135656	0.495572	chr19	2650755
cg08166863	0.024135	1.49513	0.135983	0.496025	chr20	57426391
cg25673737	-0.04864	-1.49281	0.136588	0.496939	chr4	101966414

cg06874426	0.04525	1.491689	0.136883	0.497407	chr17	47287526
cg07582829	0.045977	1.490266	0.137256	0.497965	chr19	54408440
cg13135654	0.033467	1.489998	0.137327	0.498087	chr11	70842116
cg14594362	0.048511	1.489951	0.137339	0.498098	chr19	48947631
cg08037774	-0.03074	-1.48987	0.137361	0.498134	chr1	53608962
cg14399447	-0.03438	-1.4891	0.137562	0.498459	chr19	48613950
cg01291761	-0.04545	-1.48849	0.137724	0.498754	chr12	14017080
cg20110535	-0.02727	-1.48835	0.137761	0.498799	chr19	14225647
cg03403991	-0.03087	-1.48775	0.13792	0.499026	chr22	51167187
cg13536060	0.047547	1.485783	0.138438	0.499748	chr19	51189671
cg03300177	-0.05542	-1.48515	0.138606	0.499994	chr16	56390811
cg08471319	0.04674	1.485089	0.138622	0.500029	chr2	25141735
cg02194717	-0.04201	-1.48503	0.138636	0.500065	chr11	70415188
cg06758670	0.039293	1.483052	0.139162	0.50096	chr16	10276383
cg25196508	-0.04396	-1.47946	0.140118	0.50241	chr12	49181554
cg00377653	-0.06799	-1.47876	0.140304	0.502728	chr12	56882535
cg24033471	0.020706	1.478409	0.140399	0.502874	chr12	2735579
cg12965344	-0.07932	-1.47811	0.14048	0.502951	chr19	48898160
cg26362197	0.078146	1.47493	0.141331	0.504413	chr19	48948005
cg22294773	0.029585	1.474859	0.14135	0.504439	chr19	2606054
cg05876496	0.03541	1.472852	0.141891	0.505231	chr6	33638550
cg03562531	-0.02858	-1.47272	0.141927	0.505282	chr3	53764604
cg05161074	-0.03729	-1.4722	0.142067	0.505467	chr15	42289885
cg12198334	0.082349	1.471317	0.142305	0.505803	chr11	70692032
cg21797718	-0.057	-1.47082	0.14244	0.506027	chr6	34024416
cg06952307	0.039382	1.470214	0.142603	0.506216	chr17	47287974
cg08931917	-0.04536	-1.46922	0.142872	0.506531	chr12	14109569
cg03499570	-0.03036	-1.46616	0.143704	0.507854	chr11	70424529
cg05546044	-0.04497	-1.46419	0.14424	0.508852	chr22	22222597
cg23982812	0.070508	1.463196	0.144512	0.509335	chr12	14004950
cg22562461	-0.05344	-1.46118	0.145063	0.510243	chr7	86274769
cg23740474	0.032022	1.45978	0.145448	0.510735	chr11	70455140
cg01661235	0.032216	1.457068	0.146195	0.511785	chr16	23988974
cg00578437	0.033327	1.45638	0.146384	0.512107	chr22	22217249
cg08033640	0.034244	1.455411	0.146652	0.512458	chr11	70419197
cg02882755	0.037347	1.454411	0.146929	0.512893	chr6	34100963
cg08021727	0.035304	1.45333	0.147229	0.513435	chr16	4056879
cg26534489	-0.0393	-1.45274	0.147392	0.513711	chr20	57427495
cg19324023	-0.03135	-1.45121	0.147817	0.514428	chr22	51170003
cg10144604	-0.04566	-1.45089	0.147905	0.514574	chr20	57465599
cg15527678	-0.02832	-1.4495	0.148294	0.515067	chr12	49171898
cg16379885	0.031596	1.448767	0.148498	0.515469	chr1	37500369
cg18375707	-0.04514	-1.44791	0.148738	0.515779	chr11	64034959
cg01242196	-0.02856	-1.44754	0.14884	0.515958	chr6	33990181
cg10037905	0.062118	1.446801	0.149048	0.516256	chr12	26986999
cg07212894	0.040889	1.446022	0.149266	0.516573	chr3	50243021
cg20721022	0.05908	1.445466	0.149422	0.516799	chr19	54386355
cg12732284	-0.07149	-1.44442	0.149715	0.517305	chr3	171320284

cg01729401	-0.05336	-1.44237	0.150292	0.518112	chr1	1750560
cg10159951	0.039508	1.441554	0.150522	0.518447	chr11	35441881
cg16564940	0.044139	1.441274	0.150601	0.518575	chr17	7117310
cg08505076	0.038504	1.440176	0.150911	0.519047	chr17	7122063
cg04583195	0.040481	1.439495	0.151104	0.519326	chr3	179165115
cg04455869	0.039408	1.43787	0.151564	0.520052	chr5	7663853
cg00898486	0.030403	1.435214	0.152319	0.521289	chr6	34006557
cg22363670	0.040898	1.433493	0.152809	0.521982	chr7	86273169
cg23970331	-0.03312	-1.43036	0.153705	0.523268	chr6	33656237
cg24428099	-0.03214	-1.42912	0.154061	0.523769	chr2	25065702
cg02026498	-0.0498	-1.42724	0.154602	0.524535	chr19	47139338
cg23832825	-0.02938	-1.42681	0.154725	0.524679	chr11	70842453
cg24454695	0.031972	1.424964	0.155258	0.52546	chr1	235814326
cg08572336	-0.11257	-1.42404	0.155526	0.525915	chr19	51165404
cg02011392	0.043113	1.423869	0.155575	0.526012	chr6	101847541
cg11841246	0.057119	1.423315	0.155735	0.526282	chr15	52414751
cg06490627	0.052301	1.421978	0.156122	0.52675	chr16	50344198
cg13300911	0.035389	1.420696	0.156494	0.527252	chr15	42119951
cg15623249	0.049178	1.419819	0.15675	0.527618	chr19	48896947
cg01273580	0.065715	1.416801	0.15763	0.528851	chr19	49946108
cg21116900	0.042897	1.416572	0.157696	0.528957	chr12	100750760
cg15066837	-0.0467	-1.41629	0.157779	0.529101	chr7	79775261
cg00226831	0.043602	1.415786	0.157926	0.529302	chr15	42371511
cg13016048	0.047441	1.415595	0.157982	0.529387	chr9	140054056
cg26424956	-0.03619	-1.41246	0.1589	0.530791	chr6	34101526
cg20716703	-0.03657	-1.41211	0.159005	0.530952	chr19	13397750
cg22188571	-0.0342	-1.41185	0.159083	0.531085	chr6	34074955
cg19965023	-0.02977	-1.4117	0.159126	0.531143	chr17	72838366
cg02757172	0.070362	1.410169	0.159576	0.531838	chr4	158141076
cg27277859	0.060952	1.410113	0.159593	0.531851	chr19	51165632
cg00631706	0.035597	1.407607	0.160333	0.532811	chr5	78751239
cg22531801	0.061824	1.405504	0.160957	0.533597	chr1	235806070
cg09067029	-0.03524	-1.40401	0.161401	0.534211	chr1	186954259
cg03962451	0.037359	1.403863	0.161445	0.534266	chr2	191754464
cg27363558	-0.043	-1.40364	0.16151	0.53434	chr17	7108792
cg12013817	-0.03907	-1.40254	0.16184	0.534803	chr6	34008384
cg08987995	-0.03724	-1.40231	0.161906	0.534912	chr3	4788106
cg11833293	-0.03632	-1.40205	0.161985	0.535046	chr11	70557519
cg24384034	-0.04003	-1.40186	0.162041	0.535125	chr11	70858463
cg14203613	0.041315	1.39977	0.162666	0.536011	chr3	4714928
cg25367568	0.061428	1.39822	0.16313	0.536759	chr20	57428437
cg10891888	0.040735	1.39672	0.163581	0.537327	chr12	46765615
cg05765440	-0.03491	-1.39632	0.163702	0.537498	chr1	68252343
cg23501962	0.043605	1.394983	0.164103	0.538036	chr11	35440252
cg14090916	-0.05223	-1.3949	0.164128	0.538048	chr9	140044904
cg02664157	-0.06447	-1.3928	0.164762	0.538819	chr19	2702877
cg21032008	-0.0324	-1.39199	0.165007	0.539186	chr3	7724717
cg03896542	-0.03228	-1.39068	0.165404	0.53965	chr16	56378687

cg03773989	0.031973	1.390034	0.165599	0.539875	chr10	75255862
cg23055735	-0.0314	-1.38643	0.166695	0.541378	chr11	70557464
cg20740024	0.03791	1.386306	0.166733	0.541407	chr11	35303612
cg27176392	0.042041	1.385774	0.166895	0.541685	chr19	2652259
cg01935096	0.046531	1.385384	0.167014	0.541827	chr11	70391215
cg25727569	-0.02513	-1.38535	0.167025	0.541836	chr3	53845287
cg17006204	0.039038	1.385311	0.167037	0.541836	chr11	70448210
cg04029168	0.036058	1.384321	0.167339	0.542295	chr16	23963538
cg20579012	-0.02423	-1.38419	0.167381	0.542348	chr12	2803823
cg21563683	0.033975	1.383575	0.167568	0.542575	chr12	46767928
cg16671069	0.059803	1.382609	0.167863	0.542978	chr6	146350969
cg16079774	0.049793	1.380883	0.168393	0.543697	chr11	22364498
cg26129110	-0.03273	-1.37919	0.168913	0.544511	chr5	7619983
cg05115424	0.034443	1.378774	0.169042	0.544715	chr17	64787379
cg14564778	-0.05384	-1.37782	0.169335	0.545138	chr20	57427556
cg06112910	0.050731	1.377273	0.169505	0.545421	chr6	33995939
cg01157070	-0.06403	-1.37715	0.169544	0.545503	chr16	56228511
cg25475999	-0.0498	-1.37603	0.169889	0.545913	chr11	35282046
cg13416129	-0.04755	-1.37527	0.170123	0.546212	chr9	140037808
cg06622135	0.035006	1.375247	0.170131	0.546212	chr11	70474416
cg15389472	0.051374	1.366014	0.173008	0.550376	chr1	182361528
cg19914607	0.041541	1.364652	0.173435	0.551007	chr3	50242505
cg26268742	-0.03926	-1.36455	0.173467	0.551058	chr19	48563560
cg19915762	-0.02603	-1.36288	0.173991	0.551774	chr11	64023086
cg15279308	-0.03616	-1.36241	0.17414	0.552002	chr12	2800500
cg20582984	-0.02647	-1.36209	0.174241	0.552157	chr20	57417233
cg11797430	-0.02842	-1.36185	0.174318	0.552264	chr12	2761364
cg01943657	-0.06545	-1.36149	0.174429	0.552428	chr4	102268799
cg16341159	0.031954	1.361485	0.174432	0.552431	chr17	7121370
cg08994082	-0.04394	-1.3585	0.175376	0.553707	chr19	2525459
cg21142456	0.04452	1.357595	0.175663	0.554111	chr15	42376744
cg02409125	0.052926	1.357312	0.175752	0.554184	chr7	126889555
cg19356346	0.041953	1.355995	0.17617	0.554661	chr12	100749441
cg09354294	-0.02575	-1.35569	0.176268	0.554787	chr1	68188118
cg15122716	-0.03614	-1.3548	0.17655	0.555278	chr9	140050568
cg10272968	0.033415	1.353893	0.17684	0.555625	chr19	2611105
cg11300838	-0.02702	-1.35313	0.177082	0.555916	chr19	19049950
cg20759084	0.049391	1.351852	0.177491	0.556371	chr5	7395875
cg18117347	0.039129	1.350387	0.17796	0.557021	chr19	51196754
cg07926858	0.04992	1.348709	0.178497	0.557713	chr10	75252761
cg19606462	-0.05025	-1.34772	0.178817	0.558109	chr5	7827964
cg18423469	-0.03278	-1.34388	0.180052	0.559824	chr18	3726858
cg08098950	0.050076	1.343796	0.180079	0.559834	chr16	4033226
cg09611472	0.070941	1.343511	0.180171	0.560036	chr16	850371
cg00444360	-0.04472	-1.34273	0.180425	0.56032	chr11	88270612
cg22175856	0.038452	1.341605	0.180788	0.560667	chr19	15084302
cg05902503	-0.02977	-1.33994	0.181328	0.561303	chr16	30133175
cg13763339	0.049565	1.338723	0.181724	0.561795	chr11	70516627

cg02858053	-0.05254	-1.33864	0.181751	0.561834	chr1	84971927
cg13762474	0.05543	1.33443	0.183125	0.563505	chr15	42371808
cg27661264	-0.04701	-1.33427	0.183177	0.563584	chr20	57427738
cg00383081	0.037015	1.334218	0.183194	0.563614	chr1	182362088
cg18872881	-0.04953	-1.33271	0.183688	0.564374	chr4	102199803
cg15442907	-0.03462	-1.33012	0.184537	0.565478	chr12	2800463
cg05848509	0.040746	1.329877	0.184618	0.565642	chr17	72857354
cg16707895	0.049731	1.329782	0.18465	0.565676	chr19	13394116
cg07688749	-0.0261	-1.32936	0.18479	0.565845	chr12	46767132
cg00336149	-0.04914	-1.32831	0.185133	0.56616	chr3	53700195
cg16567056	-0.03274	-1.328	0.185237	0.566306	chr15	40599985
cg09448677	-0.08301	-1.32694	0.185586	0.566704	chr11	70672740
cg01588464	-0.04506	-1.3261	0.185863	0.566989	chr1	182360063
cg21375506	-0.03758	-1.32518	0.186168	0.567325	chr17	64470354
cg27431037	0.058142	1.323538	0.186714	0.56798	chr12	2330387
cg23249369	-0.03904	-1.32117	0.187499	0.569088	chr20	57426759
cg25976563	0.030116	1.320282	0.187796	0.569393	chr3	179169592
cg07628416	0.069121	1.320128	0.187848	0.569458	chr16	4033297
cg17567700	0.03818	1.314134	0.189854	0.571931	chr22	51112218
cg07751222	0.053905	1.31393	0.189923	0.571992	chr4	102268245
cg09555879	0.027299	1.313705	0.189998	0.57207	chr19	42569956
cg10336707	-0.06653	-1.31073	0.191001	0.57325	chr11	62476526
cg22242216	0.059092	1.306726	0.192356	0.574824	chr19	48947578
cg05666036	0.074045	1.306555	0.192414	0.574872	chr6	101848236
cg16448399	0.047074	1.302747	0.193709	0.576562	chr18	3880076
cg07546293	0.043984	1.301142	0.194257	0.577299	chr16	851255
cg24867180	-0.04526	-1.29747	0.195515	0.578927	chr15	42120426
cg24190415	0.070931	1.297406	0.195537	0.578947	chr11	35441012
cg17604429	0.034576	1.296832	0.195734	0.579283	chr5	7827133
cg19694519	0.027366	1.296486	0.195853	0.579456	chr8	22389206
cg05745748	0.043688	1.296308	0.195914	0.579497	chr6	33996013
cg01814344	-0.0347	-1.29218	0.19734	0.581324	chr5	178413313
cg07533824	-0.03583	-1.29083	0.197805	0.581845	chr19	13363864
cg01700524	-0.04284	-1.289	0.19844	0.582731	chr14	24803014
cg23638849	-0.03681	-1.2884	0.198649	0.582942	chr11	70477184
cg22490420	-0.0277	-1.28807	0.198763	0.583085	chr15	42434040
cg10503359	0.060129	1.288012	0.198783	0.583093	chr3	142468059
cg03991309	-0.03618	-1.2874	0.198996	0.58333	chr1	68237761
cg26621408	-0.03297	-1.28689	0.199173	0.583549	chr7	100275863
cg08325885	0.036471	1.28558	0.19963	0.584194	chr8	22297108
cg00110769	-0.04491	-1.28555	0.199639	0.584211	chr1	110122089
cg27586581	0.040973	1.283486	0.200361	0.585106	chr19	19051157
cg23854103	0.079927	1.283293	0.200428	0.585209	chr19	2543602
cg18618964	-0.03475	-1.28228	0.200781	0.585658	chr2	68480222
cg21970929	-0.03574	-1.28158	0.201026	0.585887	chr5	36608598
cg06512271	0.032793	1.280173	0.201522	0.586474	chr5	7394927
cg12043631	-0.02827	-1.28004	0.201567	0.586513	chr11	70643814
cg08290212	-0.05149	-1.27941	0.20179	0.586741	chr11	62473659

cg05340495	-0.02743	-1.27874	0.202025	0.586943	chr14	52327368
cg05632631	0.029184	1.278057	0.202266	0.587176	chr16	24099614
cg00536924	0.032772	1.277808	0.202353	0.587238	chr7	93551004
cg23982607	-0.07301	-1.27763	0.202415	0.587331	chr1	1823379
cg02504690	-0.02569	-1.2762	0.202921	0.587878	chr11	70319385
cg21188533	-0.08178	-1.27385	0.20375	0.588778	chr3	53700263
cg16864295	0.036826	1.268164	0.205771	0.591196	chr3	171463753
cg23183497	0.031531	1.267168	0.206126	0.591762	chr7	86273718
cg27107076	0.060035	1.267099	0.206151	0.591785	chr2	25050844
cg05456713	-0.03421	-1.26705	0.206168	0.591804	chr19	2513356
cg14898177	0.03032	1.266502	0.206364	0.592026	chr12	2486634
cg19946638	-0.04369	-1.26414	0.20721	0.592972	chr11	70859647
cg26560871	0.044276	1.263794	0.207333	0.593106	chr6	146348616
cg24008901	-0.05244	-1.26185	0.208029	0.593968	chr12	13715497
cg17846122	-0.03566	-1.2593	0.20895	0.594909	chr17	64351144
cg12240358	-0.04215	-1.25811	0.20938	0.595323	chr15	83619523
cg06194010	0.03263	1.257367	0.209646	0.595625	chr12	2493567
cg21024916	-0.04347	-1.25541	0.210354	0.596328	chr3	4535815
cg09598225	0.043028	1.253158	0.211172	0.597237	chr20	57466839
cg10126715	0.02291	1.252603	0.211373	0.597489	chr1	37500195
cg23315601	-0.03502	-1.25111	0.211917	0.598184	chr11	120590134
cg16378117	-0.02623	-1.24999	0.212326	0.598644	chr16	9857804
cg04496615	0.03951	1.249948	0.21234	0.598657	chr2	68479620
cg20984065	-0.02768	-1.24899	0.212689	0.599089	chr3	51745321
cg19592829	0.018015	1.248317	0.212936	0.599373	chr20	57426215
cg06996976	0.023826	1.245908	0.213818	0.600515	chr17	64576105
cg20490197	-0.02758	-1.24581	0.213853	0.600535	chr6	34000298
cg06485596	0.02279	1.245412	0.214	0.600728	chr16	24112658
cg03225817	0.070366	1.245349	0.214023	0.600774	chr11	105481317
cg15114328	0.031665	1.244854	0.214205	0.600998	chr1	182361557
cg08942894	0.029794	1.244754	0.214241	0.601014	chr15	83563792
cg09906922	0.034934	1.242563	0.215047	0.601823	chr19	14203252
cg15704369	-0.03203	-1.24143	0.215465	0.602362	chr1	84543558
cg21725954	0.062358	1.241248	0.215531	0.602429	chr6	146348890
cg11871050	-0.03673	-1.24121	0.215546	0.602434	chr15	52477794
cg24030173	0.021039	1.24119	0.215552	0.602434	chr19	48922140
cg13823003	0.025473	1.240927	0.21565	0.602529	chr17	72856049
cg11021321	0.043451	1.237799	0.216806	0.603857	chr20	57471660
cg14468634	-0.04863	-1.23596	0.217488	0.604807	chr5	78758952
cg01937808	0.03291	1.234786	0.217923	0.605315	chr16	4025588
cg04761722	0.055919	1.233787	0.218295	0.6056	chr16	10274315
cg26780231	-0.07149	-1.23349	0.218407	0.605749	chr17	64468338
cg04404381	-0.03573	-1.23339	0.218443	0.605758	chr11	70563580
cg13599596	-0.04044	-1.23235	0.218829	0.606286	chr9	4541807
cg10168763	-0.05593	-1.23204	0.218945	0.606433	chr16	4166767
cg18414579	-0.03349	-1.23196	0.218974	0.606446	chr4	102266325
cg23321702	-0.03663	-1.2314	0.219185	0.606686	chr6	34031060
cg07959068	-0.02498	-1.23104	0.21932	0.606922	chr16	24057138

cg12581769	-0.0225	-1.231	0.219336	0.60695	chr19	13412999
cg03490157	0.0615	1.230664	0.21946	0.607084	chr3	6906061
cg12452300	-0.0174	-1.22849	0.220274	0.607868	chr3	51751788
cg06044900	0.042387	1.224481	0.221778	0.609362	chr20	57467811
cg19088553	-0.05331	-1.21907	0.223822	0.611641	chr6	101901884
cg22364668	-0.06343	-1.21891	0.223883	0.611722	chr19	49944826
cg00303541	0.060584	1.218349	0.224095	0.611994	chr3	51741280
cg18247436	0.030636	1.217022	0.224599	0.612608	chr11	120856646
cg14637685	0.029251	1.216976	0.224616	0.612608	chr12	2411116
cg27073113	0.032866	1.216707	0.224719	0.612712	chr16	56228744
cg10699496	0.031027	1.216494	0.2248	0.612762	chr3	123005576
cg01041222	0.04136	1.215968	0.225	0.61299	chr4	158142863
cg11479156	-0.05835	-1.21425	0.225655	0.613642	chr11	70672388
cg14654306	0.03745	1.213817	0.225819	0.613878	chr1	186798107
cg04446870	0.025857	1.213701	0.225863	0.61393	chr17	64440273
cg24276988	0.048496	1.212889	0.226173	0.614342	chr20	57463106
cg08462108	0.029134	1.211769	0.226601	0.614838	chr11	70680470
cg16862315	-0.04889	-1.21112	0.22685	0.615111	chr17	7123138
cg03324851	-0.02437	-1.21049	0.22709	0.615297	chr7	100274414
cg01090161	0.025468	1.210284	0.227169	0.615418	chr6	34032747
cg25338454	-0.02699	-1.21022	0.227192	0.615462	chr12	26900022
cg23942984	0.050335	1.208666	0.227789	0.616098	chr12	14103087
cg10011623	-0.02831	-1.20864	0.227801	0.616098	chr20	57463527
cg13070193	0.030958	1.208392	0.227895	0.616195	chr7	45613752
cg14792912	-0.02934	-1.2081	0.228008	0.616333	chr3	51742779
cg05282459	-0.02421	-1.20762	0.228191	0.61648	chr22	51117157
cg07176385	0.054764	1.207373	0.228286	0.616607	chr5	7397756
cg07678592	-0.0264	-1.20564	0.228951	0.617261	chr12	49178406
cg26334023	0.040464	1.205642	0.228952	0.617261	chr17	47287492
cg25652859	-0.04588	-1.2047	0.229317	0.617697	chr20	57427412
cg06628693	-0.05127	-1.20247	0.230176	0.618653	chr1	84543156
cg22834542	-0.04008	-1.20214	0.230304	0.618708	chr11	88288530
cg16222802	-0.03029	-1.20047	0.230949	0.619408	chr3	50295474
cg15128801	0.03956	1.198129	0.231859	0.620562	chr1	68202053
cg18854735	0.039429	1.195258	0.232976	0.621563	chr1	1822972
cg23159236	-0.02791	-1.1941	0.233428	0.622051	chr20	57464002
cg07855933	-0.02881	-1.19345	0.233682	0.622318	chr19	13349725
cg24039816	0.036102	1.192574	0.234025	0.622656	chr19	51220098
cg13148511	-0.04806	-1.19131	0.23452	0.623295	chr17	4710380
cg18689402	-0.14882	-1.19122	0.234554	0.623333	chr3	4630986
cg21157507	0.02631	1.190301	0.234915	0.623783	chr11	70830058
cg22901212	0.044948	1.189856	0.23509	0.623902	chr19	1003348
cg00495303	-0.10405	-1.18933	0.235295	0.624091	chr18	3771110
cg21971807	0.03064	1.187883	0.235865	0.624652	chr20	57471654
cg13537240	-0.02387	-1.18628	0.236498	0.625411	chr12	2761549
cg23698058	-0.04462	-1.18612	0.236558	0.625465	chr1	84544097
cg26332715	-0.07911	-1.18609	0.23657	0.625478	chr2	191745502
cg24082826	0.03187	1.185835	0.236672	0.625572	chr12	26985738

cg22971402	-0.03526	-1.1845	0.2372	0.626152	chr6	33993822
cg15114105	-0.07468	-1.18074	0.238687	0.627769	chr19	49944820
cg10302550	-0.04598	-1.18063	0.238732	0.627809	chr20	57427821
cg08698835	-0.03854	-1.17974	0.239084	0.628234	chr16	56279276
cg13651483	0.046392	1.178673	0.239508	0.628788	chr19	48566587
cg01360067	-0.04119	-1.17651	0.240371	0.629827	chr10	75256027
cg27299660	0.03698	1.176487	0.240379	0.629837	chr3	171527797
cg14870792	0.037381	1.174865	0.241026	0.630387	chr12	2163532
cg02240622	0.016757	1.174471	0.241183	0.630556	chr15	40601467
cg20936920	-0.02762	-1.17356	0.241546	0.630941	chr3	7782210
cg07121340	0.030276	1.173298	0.241653	0.631075	chr12	2173987
cg26912314	-0.02839	-1.17182	0.242244	0.631714	chr16	56291781
cg03303857	0.03574	1.170259	0.242871	0.632364	chr11	120619307
cg20408693	-0.03278	-1.16956	0.243153	0.632685	chr12	46767289
cg20646491	-0.03463	-1.1686	0.243539	0.633116	chr5	36608769
cg09654471	-0.03397	-1.16797	0.243792	0.633405	chr3	4624132
cg02754494	-0.03186	-1.16551	0.244783	0.634328	chr5	78810199
cg04733951	0.029665	1.165341	0.244852	0.634415	chr17	64545218
cg14418176	0.025531	1.163163	0.245733	0.635438	chr2	25050403
cg03654598	-0.03388	-1.16232	0.246075	0.635813	chr19	51202141
cg23232299	0.0468	1.162131	0.246151	0.635873	chr1	68212170
cg15329866	-0.03355	-1.16073	0.24672	0.63636	chr3	171455826
cg07456682	0.024655	1.158368	0.24768	0.637153	chr5	178414540
cg03832839	0.047334	1.158321	0.247699	0.637171	chr19	54401967
cg10648542	-0.02585	-1.15744	0.248058	0.637536	chr5	178416050
cg01141838	0.020846	1.156062	0.248621	0.638054	chr11	62474744
cg03861217	-0.03258	-1.15425	0.249363	0.638582	chr2	155652401
cg19466160	0.041861	1.152604	0.250036	0.639327	chr17	7117160
cg10259111	0.030581	1.150021	0.251096	0.640395	chr14	52429547
cg11281320	-0.02433	-1.14966	0.251243	0.640549	chr11	120553251
cg06344265	-0.1361	-1.14842	0.251755	0.641119	chr11	120530973
cg03466124	0.034144	1.147775	0.252021	0.641397	chr3	179168156
cg13588054	-0.03498	-1.14771	0.252049	0.641438	chr7	126892578
cg01355739	0.02215	1.147247	0.252238	0.641624	chr20	57416888
cg20152382	-0.02649	-1.14678	0.252429	0.641735	chr17	64783099
cg21269738	0.02155	1.144178	0.253507	0.642861	chr12	2356703
cg03217795	-0.05211	-1.14398	0.253589	0.642952	chr16	23847556
cg25762078	-0.02681	-1.1436	0.253746	0.643064	chr1	53554504
cg14767950	0.030527	1.142169	0.254339	0.643691	chr19	49939773
cg02274728	-0.02385	-1.14141	0.254654	0.643995	chr20	57414407
cg00147943	-0.03045	-1.13757	0.256254	0.645526	chr1	68225713
cg02637414	-0.02464	-1.13676	0.256588	0.645805	chr19	13613091
cg26875958	-0.0255	-1.13582	0.256983	0.646221	chr6	146751590
cg07217499	-0.02708	-1.13565	0.257056	0.646302	chr12	2416339
cg06829391	-0.02691	-1.13551	0.257114	0.646365	chr16	9857151
cg03100801	0.045276	1.132161	0.258515	0.647837	chr20	9075962
cg12664560	-0.08133	-1.13073	0.259114	0.648341	chr15	83621517
cg01903557	0.024198	1.130164	0.259354	0.648538	chr3	179169602

cg02035425	0.033894	1.12876	0.259945	0.649109	chr9	140062632
cg21633134	0.033201	1.127734	0.260377	0.64942	chr5	7816399
cg25856632	-0.02666	-1.12651	0.260895	0.649981	chr17	7111551
cg03768297	0.029346	1.126363	0.260956	0.650055	chr15	52441108
cg24867458	-0.03042	-1.1237	0.262082	0.651113	chr1	110090677
cg23613253	-0.01941	-1.12254	0.262575	0.651509	chr11	70440347
cg15814923	-0.06814	-1.12248	0.2626	0.651533	chr19	14228610
cg06517489	0.0274	1.120341	0.263508	0.652375	chr11	22359333
cg18997188	-0.0289	-1.12023	0.263556	0.652429	chr20	57463270
cg15631127	0.011889	1.120156	0.263587	0.652452	chr20	57426580
cg14483383	-0.02669	-1.12006	0.263626	0.652499	chr11	64022763
cg02098786	-0.0529	-1.1198	0.26374	0.652569	chr14	24801794
cg05068686	-0.03073	-1.11895	0.264101	0.652893	chr11	70419186
cg17839232	-0.049	-1.1182	0.264422	0.653244	chr5	36686601
cg02740128	-0.02739	-1.11661	0.265096	0.65383	chr17	7123860
cg13370485	-0.02245	-1.11628	0.265241	0.653961	chr12	2762840
cg24995240	0.042914	1.11418	0.266137	0.654812	chr1	84971124
cg23494413	-0.02539	-1.11338	0.266478	0.655124	chr12	6954534
cg16986624	-0.04844	-1.11243	0.266887	0.655572	chr15	52471717
cg24603152	0.064234	1.112169	0.267	0.655656	chr1	84543539
cg19548470	-0.02319	-1.11165	0.267221	0.655856	chr18	3880510
cg26314755	-0.02621	-1.11075	0.267607	0.656196	chr19	42550298
cg10851168	-0.02689	-1.11062	0.267663	0.656255	chr11	70317508
cg15908975	-0.05225	-1.10792	0.268829	0.657432	chr7	126698829
cg22983529	0.031222	1.106975	0.269235	0.657743	chr7	93551132
cg24009995	0.039737	1.106733	0.269339	0.657819	chr14	52434702
cg14530764	-0.02081	-1.1049	0.27013	0.65861	chr3	123124018
cg05800983	-0.03663	-1.10341	0.270778	0.659124	chr6	34102530
cg01722994	0.024998	1.101661	0.271535	0.659933	chr16	10277317
cg23409374	-0.04909	-1.10161	0.271557	0.659949	chr19	49934742
cg09710790	-0.03822	-1.09797	0.273142	0.661315	chr3	4534905
cg05112254	-0.02414	-1.09635	0.27385	0.662093	chr12	2224410
cg03412547	0.032677	1.096279	0.273879	0.662093	chr16	4057728
cg13934625	-0.03932	-1.09561	0.27417	0.662371	chr15	52472770
cg24801123	0.038927	1.094315	0.274738	0.662955	chr7	45615503
cg05362860	0.030002	1.093518	0.275086	0.663272	chr16	50320692
cg14011070	-0.05117	-1.09339	0.275141	0.663334	chr1	235811811
cg27368776	-0.05385	-1.09221	0.27566	0.663853	chr11	105793986
cg11659796	0.024219	1.090339	0.276482	0.664447	chr19	2560038
cg23119809	0.026014	1.089784	0.276726	0.664639	chr17	72839750
cg25439807	-0.08434	-1.08956	0.276822	0.664716	chr18	3771151
cg08289346	-0.02195	-1.08923	0.276971	0.664865	chr6	34089350
cg15334006	-0.02324	-1.08701	0.277947	0.665665	chr15	42449916
cg12159995	-0.03007	-1.08673	0.27807	0.665807	chr11	70398671
cg02929073	0.032944	1.085846	0.278462	0.666191	chr7	126892249
cg22088263	-0.02717	-1.0858	0.278482	0.666196	chr3	51746830
cg03885818	-0.04327	-1.08501	0.278832	0.666517	chr12	2299830
cg06171406	0.039262	1.084074	0.279245	0.666884	chr16	4050400

cg00036258	-0.02831	-1.08366	0.279427	0.667048	chr16	4029218
cg10371523	-0.02134	-1.08268	0.279864	0.667322	chr11	70395513
cg23425324	0.025721	1.082081	0.280128	0.667594	chr12	26986193
cg02066343	-0.02611	-1.07977	0.281153	0.668504	chr6	33590458
cg24131262	0.023349	1.079581	0.281238	0.668588	chr3	4549756
cg17755730	0.02439	1.077486	0.282171	0.66939	chr3	50285392
cg05100017	-0.0371	-1.07645	0.282633	0.669807	chr6	34102222
cg14597908	-0.01662	-1.07584	0.282907	0.670047	chr20	57414960
cg11480267	-0.04565	-1.07462	0.283452	0.67053	chr20	57463503
cg03211327	0.025796	1.074454	0.283525	0.67062	chr15	52470919
cg10533538	-0.03874	-1.07359	0.28391	0.670991	chr16	4166864
cg19270265	-0.04962	-1.07209	0.284582	0.671404	chr7	126275080
cg19542445	-0.02508	-1.06942	0.285783	0.672642	chr12	2596220
cg02520816	0.044285	1.068548	0.286174	0.673025	chr16	4056403
cg23524436	0.022155	1.067312	0.286731	0.673475	chr12	2161437
cg06849501	0.026207	1.067291	0.286741	0.673482	chr11	70458964
cg26265279	0.032182	1.067083	0.286834	0.673618	chr3	53566044
cg24874003	-0.0263	-1.06683	0.286948	0.673751	chr19	2602614
cg00991400	0.031789	1.066639	0.287035	0.673857	chr7	126883137
cg14851700	-0.04613	-1.06593	0.287356	0.674067	chr1	182362230
cg21130255	-0.02856	-1.06451	0.287994	0.674595	chr11	70368676
cg11070176	0.021354	1.064059	0.288199	0.674806	chr11	70489806
cg09108394	-0.02815	-1.06088	0.289641	0.676189	chr16	23850106
cg14643330	-0.02618	-1.06066	0.289738	0.676255	chr3	4534051
cg26645082	-0.0404	-1.06039	0.28986	0.676381	chr11	70563264
cg04537738	-0.02166	-1.05861	0.29067	0.677114	chr22	51143999
cg17987968	-0.02561	-1.05741	0.291218	0.677516	chr5	152869882
cg22291265	-0.0464	-1.05428	0.292646	0.679048	chr19	51182808
cg05626242	0.029979	1.051865	0.293749	0.679938	chr10	75255789
cg27019717	0.026039	1.051102	0.294099	0.680185	chr14	52351055
cg01833890	-0.01415	-1.04818	0.295439	0.681255	chr12	2564063
cg01833923	0.013146	1.046127	0.296386	0.682205	chr17	47286719
cg18352793	0.017368	1.044932	0.296937	0.68273	chr22	22210625
cg12321149	-0.03716	-1.04385	0.297438	0.683168	chr20	57427426
cg16279290	-0.02318	-1.04056	0.298959	0.684468	chr11	70368624
cg16307325	0.028783	1.040161	0.299144	0.684664	chr12	2339235
cg15160445	-0.03843	-1.03882	0.299765	0.685108	chr20	57426749
cg15680973	0.044188	1.03835	0.299984	0.685316	chr5	7420177
cg04138502	0.044004	1.037124	0.300554	0.685769	chr3	123167522
cg16685860	-0.06245	-1.03663	0.300782	0.685907	chr17	4710619
cg13844463	0.031823	1.035115	0.30149	0.686388	chr11	35436672
cg09973502	0.030789	1.03208	0.302907	0.68758	chr12	46766012
cg13891121	0.030326	1.031836	0.303021	0.687683	chr12	26987045
cg26060003	-0.0277	-1.0318	0.303037	0.687688	chr1	53558511
cg01622416	-0.03783	-1.02939	0.304166	0.688773	chr21	31310508
cg10031793	0.022601	1.029363	0.304179	0.688773	chr12	2734257
cg03043696	-0.02357	-1.02906	0.304323	0.688857	chr1	1820656
cg08461840	0.022624	1.02615	0.305688	0.690147	chr19	2620967

cg04421162	0.031754	1.025573	0.305959	0.690367	chr19	54406293
cg08407014	0.037224	1.025539	0.305975	0.690393	chr19	2541104
cg06371583	-0.02139	-1.02533	0.306074	0.690461	chr19	2581343
cg01111718	-0.02484	-1.02518	0.306143	0.690495	chr11	105762374
cg23725394	0.031018	1.024732	0.306355	0.690697	chr2	25057656
cg09640070	-0.02541	-1.02291	0.307216	0.691316	chr12	26876374
cg24220046	0.030248	1.022815	0.30726	0.691371	chr19	51171640
cg19013391	0.02822	1.02196	0.307663	0.691615	chr3	123166774
cg20227471	-0.02261	-1.02159	0.307839	0.691721	chr2	25065550
cg19622911	0.029332	1.020744	0.308238	0.692046	chr18	3771570
cg05684300	-0.02544	-1.02034	0.308428	0.692208	chr4	102267366
cg25308079	-0.01312	-1.01869	0.309211	0.692758	chr20	57463763
cg04525943	0.028806	1.018411	0.309343	0.692832	chr19	2579529
cg16204066	-0.03927	-1.01685	0.310085	0.69347	chr6	33638922
cg26562691	-0.02446	-1.01511	0.310912	0.69423	chr16	23850404
cg03120091	0.040405	1.01478	0.311068	0.694395	chr8	132052779
cg24678505	-0.04354	-1.01239	0.312208	0.695461	chr12	56882429
cg08429705	-0.21244	-1.01234	0.31223	0.69547	chr19	2583601
cg17509220	0.047589	1.011417	0.312672	0.695765	chr19	13617012
cg24481035	-0.02476	-1.00958	0.31355	0.69646	chr19	54388422
cg19815589	0.036282	1.0091	0.31378	0.696675	chr11	70709062
cg26212328	-0.05313	-1.00893	0.313862	0.696742	chr12	46767665
cg08626004	0.04735	1.00726	0.314661	0.697489	chr19	2513687
cg16669395	0.042107	1.006423	0.315063	0.697826	chr16	10208417
cg03551401	0.049706	1.006258	0.315142	0.697908	chr8	132051228
cg02551234	-0.02643	-1.0058	0.315364	0.698048	chr11	64023126
cg15109207	-0.05157	-1.00473	0.315877	0.698413	chr19	48614773
cg06099971	-0.01571	-1.00416	0.316149	0.698626	chr15	42386816
cg26670249	0.020702	1.004136	0.316162	0.698629	chr19	14223997
cg09327847	-0.02245	-1.00314	0.316642	0.699001	chr16	24087793
cg20699497	-0.0324	-1.00127	0.317543	0.699753	chr19	49944642
cg20098420	0.022555	1.000225	0.318047	0.700211	chr22	51155589
cg24603235	-0.04294	-0.99954	0.31838	0.700457	chr6	33588219
cg08969344	-0.02592	-0.99455	0.320798	0.70241	chr6	34031597
cg11803871	0.022969	0.992494	0.321795	0.703171	chr3	123039831
cg14638988	-0.03362	-0.99248	0.321803	0.703171	chr11	62476544
cg17107112	0.024763	0.991556	0.322252	0.703549	chr6	34021546
cg14583825	0.026095	0.990724	0.322658	0.703861	chr19	54393040
cg17300307	0.024272	0.988476	0.323755	0.704717	chr2	191744975
cg18935491	-0.01774	-0.98687	0.324539	0.705329	chr20	57425979
cg07114886	-0.01764	-0.9863	0.32482	0.705502	chr3	51745946
cg25314445	0.026866	0.986071	0.324932	0.705595	chr1	1718835
cg16899036	-0.01927	-0.98538	0.325272	0.705795	chr19	19052705
cg03199239	0.051749	0.985213	0.325352	0.705861	chr19	2588553
cg24250393	0.029993	0.982948	0.326464	0.707026	chr16	23846838
cg16040341	-0.02472	-0.98029	0.327771	0.708075	chr15	83544284
cg14214834	-0.01879	-0.97995	0.327939	0.708213	chr3	123139305
cg26361533	-0.02566	-0.97752	0.329137	0.709	chr12	2445561

cg02471325	0.034957	0.977205	0.329293	0.709104	chr15	42290555
cg17800870	-0.04934	-0.97637	0.329705	0.709376	chr1	182362757
cg04085699	0.020967	0.973875	0.330942	0.710227	chr11	64026024
cg27484541	0.033596	0.972988	0.331381	0.710558	chr20	57461542
cg02585344	0.035576	0.972708	0.331521	0.71067	chr16	10276092
cg09050775	0.026703	0.971393	0.332174	0.711118	chr12	46762708
cg21830821	0.034044	0.970208	0.332763	0.711558	chr15	52475339
cg10956480	0.042741	0.969309	0.33321	0.711957	chr12	2338945
cg04398695	0.052418	0.969203	0.333263	0.711987	chr16	850713
cg13883984	0.025665	0.966162	0.334779	0.713223	chr15	83621779
cg20213508	-0.02502	-0.96616	0.334781	0.713223	chr20	57463325
cg19558628	-0.02614	-0.96307	0.336325	0.714218	chr14	24801616
cg25047001	0.024582	0.962908	0.336407	0.714305	chr16	10277017
cg16582156	-0.05409	-0.95998	0.337875	0.715657	chr1	110091224
cg15245951	0.025089	0.959325	0.338205	0.715955	chr3	50283471
cg09963080	-0.03009	-0.95834	0.3387	0.716241	chr16	4017270
cg19047292	0.042567	0.957547	0.3391	0.716512	chr16	56228442
cg09017174	0.027875	0.95675	0.339501	0.716832	chr11	35440525
cg03319638	0.019274	0.955064	0.340352	0.717454	chr15	42387193
cg16253976	0.036115	0.952986	0.341402	0.718299	chr5	7686199
cg01975093	0.027183	0.951125	0.342344	0.718911	chr11	62474759
cg16774375	0.016744	0.94983	0.343	0.719418	chr16	4102333
cg21961771	0.033221	0.948969	0.343438	0.719744	chr12	100750652
cg12544243	-0.02761	-0.94681	0.344535	0.720606	chr19	1005641
cg17402889	-0.02181	-0.94646	0.344715	0.720766	chr12	2527099
cg25134567	-0.01891	-0.94511	0.345402	0.72127	chr17	64782369
cg02084729	0.026193	0.944761	0.345578	0.721424	chr6	33589691
cg12190341	0.01358	0.944679	0.34562	0.721464	chr17	72838819
cg01823958	-0.03847	-0.94397	0.345982	0.721743	chr1	53557455
cg13373757	-0.02089	-0.9423	0.346836	0.722393	chr12	2263558
cg17566735	-0.01998	-0.94227	0.34685	0.722394	chr3	171527257
cg18949315	-0.02239	-0.94203	0.346972	0.722477	chr20	57418015
cg25090051	-0.01863	-0.94131	0.347342	0.722666	chr20	57414059
cg08076125	0.044552	0.938261	0.348902	0.723901	chr6	146350230
cg15134033	0.02906	0.93693	0.349585	0.724345	chr16	10274415
cg01104717	0.030649	0.934936	0.35061	0.725112	chr3	53545550
cg24661595	-0.02599	-0.93372	0.351235	0.725586	chr11	70456848
cg02569086	-0.02711	-0.9322	0.352019	0.726119	chr12	26985672
cg00056257	0.039357	0.930317	0.352992	0.726826	chr3	6901652
cg01399255	-0.04678	-0.93022	0.353041	0.726862	chr7	100271274
cg11335969	-0.02246	-0.92983	0.353241	0.726986	chr22	22118304
cg14176797	-0.01572	-0.92878	0.353787	0.727353	chr20	57426801
cg22405973	-0.02564	-0.92813	0.354124	0.727633	chr22	22222028
cg26204322	0.06655	0.925445	0.355514	0.728757	chr11	64018687
cg16168199	0.023076	0.92374	0.3564	0.729364	chr17	7123990
cg26011633	0.023065	0.922953	0.35681	0.729693	chr5	178411667
cg02660823	0.044019	0.922761	0.356909	0.72976	chr19	54410305
cg12008034	-0.02047	-0.9227	0.356943	0.72979	chr6	33996580

cg16623098	-0.10443	-0.92264	0.356971	0.729802	chr16	56374383
cg03821543	-0.01784	-0.9223	0.357148	0.729942	chr20	57463925
cg13802605	0.019736	0.922124	0.357241	0.729982	chr9	4495359
cg04763994	-0.01955	-0.92168	0.357474	0.730129	chr19	42506259
cg07008386	0.027107	0.920621	0.358024	0.730578	chr20	8113630
cg07838205	-0.04819	-0.92056	0.358054	0.730604	chr1	110091179
cg10347199	-0.0442	-0.92014	0.358274	0.730833	chr17	7123561
cg02620388	-0.02303	-0.92006	0.358314	0.730869	chr7	100271115
cg17895496	-0.02074	-0.91821	0.359284	0.731719	chr15	42449716
cg00160359	-0.01988	-0.91674	0.360054	0.73229	chr12	49178192
cg13885159	-0.0243	-0.91541	0.36075	0.73291	chr11	62473858
cg13715127	0.022761	0.914978	0.360974	0.733092	chr17	72856825
cg00350503	0.030898	0.91444	0.361256	0.733347	chr19	13613614
cg17414107	-0.03995	-0.91422	0.361373	0.733478	chr20	57427830
cg08288223	-0.03087	-0.91396	0.361505	0.733597	chr11	70563131
cg08599266	0.028075	0.912752	0.362142	0.734026	chr2	25142473
cg09551072	-0.01464	-0.91275	0.362145	0.734026	chr1	53567678
cg04518808	0.031413	0.911793	0.362646	0.734424	chr19	54384822
cg13729891	0.023979	0.911744	0.362671	0.734452	chr17	7108468
cg04708601	-0.02356	-0.91161	0.36274	0.734476	chr6	101880078
cg21625881	-0.0147	-0.91023	0.363469	0.734977	chr20	57430313
cg23323297	0.029691	0.910078	0.363548	0.735049	chr19	51195418
cg26726141	-0.02749	-0.91004	0.36357	0.735065	chr17	64612159
cg05514043	0.045047	0.908935	0.36415	0.735438	chr9	140040822
cg00986191	0.027559	0.908005	0.36464	0.73585	chr5	178419655
cg07408989	0.018005	0.906832	0.365259	0.736252	chr17	7116792
cg25781595	0.026385	0.90488	0.36629	0.737183	chr19	48918712
cg25976932	-0.0198	-0.90219	0.367713	0.7383	chr3	123138958
cg08445323	0.030693	0.902128	0.367748	0.73833	chr16	4015030
cg20132775	0.036349	0.901874	0.367883	0.738418	chr3	142444202
cg20871277	-0.02076	-0.89986	0.36895	0.739168	chr6	33656548
cg11802781	0.027071	0.899572	0.369105	0.739273	chr11	70370534
cg01617139	0.035198	0.896777	0.370592	0.740421	chr19	2543861
cg22304522	-0.0275	-0.89646	0.370759	0.740537	chr8	131914037
cg16875863	0.045115	0.896395	0.370796	0.740556	chr19	48947572
cg04153604	-0.01671	-0.89607	0.370969	0.740659	chr17	7099823
cg10961484	-0.0248	-0.89551	0.371266	0.740831	chr11	120530860
cg25803139	0.022406	0.895303	0.371378	0.740946	chr19	2539626
cg04505435	-0.04845	-0.8942	0.371966	0.741376	chr11	70672511
cg04190002	0.020274	0.89419	0.371972	0.741379	chr22	51113604
cg27501686	0.039157	0.893658	0.372256	0.741629	chr12	2184874
cg08111661	-0.02171	-0.89156	0.373377	0.742492	chr12	2223540
cg14753385	-0.01855	-0.89143	0.373447	0.742521	chr11	70476422
cg26826325	0.019218	0.890118	0.374151	0.743001	chr11	70459053
cg16684939	-0.0212	-0.88879	0.374865	0.743428	chr7	100272223
cg14395444	0.031301	0.888662	0.374932	0.743489	chr12	2800182
cg18166990	-0.0205	-0.88798	0.375299	0.743752	chr11	70515565
cg03760316	0.023658	0.887674	0.375462	0.743889	chr18	3594197

cg16999602	-0.01862	-0.88731	0.375655	0.744014	chr1	53608163
cg07960450	0.040799	0.88602	0.376351	0.744465	chr7	45614300
cg03966406	-0.02145	-0.88458	0.377126	0.744967	chr12	26985412
cg06170425	0.019467	0.883045	0.377953	0.745535	chr16	4164087
cg03282345	0.030481	0.882977	0.37799	0.745547	chr19	49934577
cg04142017	0.046509	0.882296	0.378358	0.745817	chr1	53558596
cg17838127	-0.02108	-0.88209	0.378466	0.745856	chr8	22298935
cg13709913	0.018342	0.88205	0.378491	0.745877	chr9	104334427
cg00020720	0.019837	0.881405	0.378839	0.746178	chr1	84972482
cg03109047	0.026625	0.880529	0.379312	0.74652	chr9	140032686
cg00014104	-0.02409	-0.88024	0.379471	0.746589	chr22	22220367
cg05607461	-0.02554	-0.87939	0.379926	0.746857	chr2	191745416
cg24194077	-0.0218	-0.87895	0.380166	0.746978	chr19	54386020
cg10768900	0.027178	0.877698	0.380844	0.747538	chr11	70557881
cg14847975	-0.04635	-0.87528	0.382154	0.748358	chr12	26986502
cg08884490	-0.01682	-0.8729	0.383452	0.749432	chr22	51160651
cg16728539	-0.02003	-0.87256	0.383635	0.749545	chr12	2451169
cg17902551	0.019462	0.872482	0.383677	0.749574	chr12	2801061
cg15728692	-0.02269	-0.87199	0.383947	0.749774	chr22	51135736
cg20748533	0.030957	0.871638	0.384137	0.749927	chr19	51189975
cg17155859	0.018879	0.871561	0.384179	0.749962	chr11	64026541
cg01637551	-0.03837	-0.87121	0.384371	0.75008	chr12	2161661
cg03306374	-0.04905	-0.87039	0.384817	0.750374	chr16	23847325
cg14792155	-0.01932	-0.86955	0.385275	0.750708	chr15	42289618
cg06772874	-0.02333	-0.86867	0.385754	0.751086	chr1	110090953
cg10692302	0.039668	0.867987	0.386129	0.751408	chr3	51747227
cg09209803	-0.02947	-0.86782	0.38622	0.751448	chr6	33588932
cg14462402	-0.0307	-0.86381	0.388414	0.752977	chr11	120678447
cg01708648	0.019022	0.86291	0.38891	0.75328	chr11	64023044
cg14101380	-0.02177	-0.86123	0.389833	0.753905	chr11	70718575
cg24755189	-0.01849	-0.86122	0.389838	0.75391	chr11	62475373
cg02288564	0.051515	0.860974	0.389974	0.754037	chr19	49934404
cg13078421	0.023282	0.859594	0.390733	0.754634	chr19	2624622
cg24081764	0.036804	0.85829	0.391451	0.755125	chr19	54402116
cg26320601	0.02344	0.857194	0.392056	0.755569	chr12	26986803
cg24090202	-0.02665	-0.85668	0.392337	0.755753	chr19	54410030
cg12451177	0.030262	0.855342	0.393078	0.756137	chr22	22222048
cg24446178	0.027529	0.852873	0.394443	0.757027	chr12	100750702
cg14397813	-0.02395	-0.85215	0.394846	0.757382	chr9	80522508
cg05648629	0.032782	0.852128	0.394856	0.757394	chr16	4162203
cg11422541	-0.03425	-0.85178	0.395047	0.757533	chr12	49182586
cg17159473	0.021216	0.851658	0.395116	0.757583	chr7	126893890
cg25210134	0.026431	0.85164	0.395126	0.757591	chr15	40600265
cg05279172	-0.02284	-0.85159	0.395154	0.757591	chr17	7113697
cg02910037	-0.01795	-0.84997	0.396051	0.758345	chr16	4102366
cg04037585	-0.02145	-0.84909	0.39654	0.758702	chr16	56231292
cg27272547	0.02543	0.84812	0.39708	0.759093	chr19	13347337
cg20492121	-0.02303	-0.84464	0.399016	0.760397	chr1	182361258

cg07843390	0.017484	0.843592	0.399603	0.760836	chr19	2541015
cg01716959	-0.02113	-0.84229	0.400328	0.761225	chr11	70515714
cg20073686	0.022191	0.839887	0.401673	0.762119	chr11	105481863
cg27279652	0.025835	0.838752	0.402309	0.762498	chr12	26986506
cg14995148	-0.01891	-0.83757	0.40297	0.76295	chr5	36683916
cg09169283	-0.025	-0.83719	0.403186	0.763104	chr17	72856452
cg16993684	-0.0619	-0.83708	0.403245	0.763108	chr20	57466131
cg03371918	0.025939	0.835379	0.404201	0.763714	chr17	64297988
cg21810373	-0.02861	-0.83327	0.405389	0.764462	chr11	70917159
cg02328440	0.021108	0.831613	0.406321	0.76505	chr1	235813839
cg25839745	0.022636	0.83111	0.406605	0.765239	chr4	101969341
cg27181295	-0.01822	-0.83089	0.406731	0.765275	chr19	2511475
cg18870258	0.03874	0.82976	0.407366	0.765587	chr20	9460935
cg18046365	-0.02559	-0.82965	0.407431	0.765619	chr1	53608367
cg17652507	-0.02278	-0.82891	0.407848	0.76594	chr20	57463653
cg12276123	0.034	0.828167	0.408266	0.76622	chr2	155555157
cg08028452	-0.02349	-0.82657	0.409171	0.766881	chr3	53545444
cg00317626	0.023453	0.826251	0.40935	0.767034	chr1	53600741
cg04590790	0.035432	0.825367	0.409851	0.767296	chr5	7770690
cg17377463	-0.01629	-0.82188	0.411831	0.768673	chr19	48908557
cg04062190	-0.02191	-0.82139	0.412108	0.768771	chr7	86413438
cg05921947	0.025827	0.820642	0.412534	0.769039	chr5	7715579
cg15372689	0.029023	0.820077	0.412855	0.769234	chr7	93550986
cg11067712	0.022245	0.819721	0.413058	0.76942	chr11	105850644
cg15067127	-0.02508	-0.81943	0.413222	0.769529	chr1	182352978
cg15350840	0.030391	0.819425	0.413226	0.769529	chr11	70475709
cg26343183	0.025267	0.818431	0.413793	0.769932	chr5	153008721
cg03837903	-0.03755	-0.81781	0.414148	0.770191	chr20	57464000
cg13916928	0.020294	0.816961	0.414631	0.770493	chr11	62474735
cg03511974	0.027103	0.816558	0.414861	0.770648	chr1	53568259
cg08074971	0.032037	0.815991	0.415185	0.770744	chr16	850562
cg20626645	0.037091	0.815625	0.415394	0.77092	chr3	53528846
cg05693127	-0.01965	-0.81454	0.416013	0.771333	chr6	33643684
cg02155655	-0.0119	-0.81416	0.416228	0.771437	chr1	53566481
cg02090654	-0.03016	-0.81356	0.416571	0.77163	chr7	126698344
cg25121007	-0.02305	-0.81355	0.416577	0.77163	chr7	126854672
cg25397191	-0.01961	-0.81333	0.416704	0.771674	chr19	14224992
cg12502079	-0.01723	-0.81248	0.417191	0.772036	chr22	51169028
cg21496785	0.021858	0.807914	0.419811	0.773709	chr5	178420865
cg00536080	-0.02938	-0.80693	0.420379	0.774063	chr19	13409931
cg13583454	-0.0354	-0.80342	0.422399	0.775396	chr5	153038077
cg16163543	-0.02838	-0.80179	0.423339	0.776093	chr11	70449219
cg10453337	-0.02334	-0.80126	0.423649	0.776268	chr5	7502014
cg06465194	-0.02709	-0.80089	0.423863	0.776445	chr11	120531034
cg02046017	-0.02157	-0.80044	0.424118	0.776628	chr11	70707406
cg16102063	-0.03248	-0.80018	0.424272	0.776712	chr8	22298240
cg18574254	0.042735	0.799815	0.424482	0.776874	chr7	126889015
cg24640697	0.014953	0.799704	0.424546	0.776891	chr1	84970057

cg04894216	-0.03585	-0.79916	0.424863	0.777115	chr7	86377879
cg03015368	-0.02419	-0.79632	0.426507	0.7782	chr16	10065222
cg07526227	-0.02357	-0.79599	0.4267	0.778285	chr19	51168305
cg18558423	-0.01858	-0.79585	0.42678	0.778312	chr6	33653506
cg19794207	0.02127	0.794288	0.427686	0.778828	chr9	140040231
cg12467435	-0.01809	-0.79234	0.42882	0.779529	chr17	64651126
cg18571531	-0.01627	-0.79189	0.429081	0.779659	chr11	70477192
cg18389339	-0.0357	-0.79124	0.429462	0.779878	chr18	3730593
cg27351813	0.02257	0.790291	0.430013	0.780168	chr19	51197056
cg13896105	-0.0131	-0.78979	0.430307	0.780263	chr12	2304473
cg20837354	-0.02014	-0.78962	0.430405	0.780315	chr12	2398146
cg27279809	-0.02914	-0.78938	0.430543	0.780396	chr12	49183212
cg04603130	-0.02226	-0.78818	0.431247	0.780741	chr19	2550027
cg12760563	-0.02529	-0.78816	0.431254	0.780741	chr18	3594396
cg08481112	0.035593	0.787409	0.431695	0.78096	chr19	2544100
cg09150064	0.033536	0.7827	0.434451	0.782629	chr11	22364185
cg15591578	-0.02087	-0.78232	0.434676	0.782795	chr19	51219167
cg19274368	-0.01824	-0.78191	0.434913	0.782949	chr11	70331491
cg03527683	-0.02101	-0.7811	0.435388	0.783238	chr7	126752816
cg24342051	0.022702	0.780825	0.435551	0.783357	chr3	53527884
cg01748573	-0.01798	-0.78052	0.435732	0.78346	chr20	57463530
cg10324572	0.017482	0.780223	0.435905	0.783575	chr3	51749334
cg14337339	0.023337	0.779521	0.436317	0.783866	chr3	53529481
cg26767990	0.011279	0.779353	0.436416	0.783955	chr20	57463615
cg08204867	0.029219	0.779175	0.436521	0.784035	chr16	10208426
cg17721618	-0.0144	-0.77751	0.437501	0.784601	chr15	42376692
cg18295203	-0.02069	-0.77375	0.439719	0.786076	chr3	171528273
cg26389955	0.020092	0.773661	0.43977	0.786093	chr17	64640562
cg21250721	-0.0429	-0.77289	0.440224	0.786314	chr9	140056227
cg26672104	0.018291	0.772841	0.440255	0.786329	chr14	52327433
cg15863841	0.018482	0.771183	0.441235	0.78695	chr3	171430173
cg14167033	0.140679	0.770822	0.441449	0.787069	chr11	70424559
cg19796640	0.019646	0.767953	0.443149	0.788079	chr17	72848197
cg25594486	-0.04547	-0.76764	0.443336	0.788175	chr19	51165441
cg06015218	0.024168	0.767527	0.443401	0.788222	chr6	146350434
cg04987335	-0.02343	-0.76693	0.443755	0.788433	chr11	35288779
cg16446012	0.021403	0.766754	0.44386	0.788489	chr5	78772259
cg11906607	-0.01673	-0.76637	0.444091	0.788632	chr19	2524064
cg18411237	0.027513	0.76617	0.444207	0.788652	chr11	70653173
cg04019914	-0.02462	-0.76552	0.444592	0.788878	chr20	57463357
cg07841877	-0.01903	-0.76449	0.445204	0.789229	chr1	182359858
cg06563300	0.030467	0.763476	0.445809	0.789599	chr12	100750811
cg04355871	0.021826	0.761225	0.44715	0.790178	chr11	64022825
cg12709244	-0.04774	-0.76088	0.447358	0.790386	chr17	7123282
cg24944109	0.040567	0.760723	0.447449	0.790452	chr11	35440136
cg11692021	-0.01883	-0.76054	0.447561	0.790518	chr12	6949472
cg13399816	-0.02344	-0.75892	0.448526	0.791081	chr1	68299468
cg19032532	-0.0241	-0.75823	0.448938	0.791328	chr19	2547067

cg09830455	-0.03185	-0.75804	0.449053	0.791434	chr11	70886197
cg05312962	0.015386	0.757688	0.449262	0.791607	chr19	2576254
cg26282150	0.039808	0.757171	0.449571	0.791771	chr1	110091625
cg06068039	-0.01922	-0.75432	0.451278	0.792721	chr6	34031208
cg16730369	0.020148	0.751854	0.452757	0.793599	chr11	120823575
cg24275501	-0.01678	-0.75098	0.45328	0.793966	chr12	2198070
cg16904585	0.022957	0.74959	0.454118	0.794431	chr16	10276119
cg24497686	0.021298	0.747437	0.455414	0.795128	chr1	53606574
cg24904943	0.029252	0.744491	0.457191	0.796237	chr5	7781077
cg12743970	0.019405	0.744396	0.457248	0.796268	chr17	64536520
cg07547788	0.018113	0.743677	0.457683	0.796515	chr7	93534693
cg25942860	-0.01556	-0.74327	0.457926	0.796616	chr5	178410055
cg09284949	0.036663	0.742719	0.458261	0.796845	chr19	51190179
cg17174980	0.026261	0.742613	0.458325	0.79687	chr12	14109514
cg13054613	-0.01798	-0.74259	0.458339	0.79687	chr3	7742036
cg19781472	0.031551	0.74242	0.458442	0.796917	chr12	56883202
cg26010751	-0.01655	-0.74241	0.458451	0.796917	chr3	51742513
cg11293016	0.026167	0.742202	0.458574	0.796992	chr19	54402647
cg12727358	0.027332	0.740104	0.459844	0.797917	chr3	53839610
cg09885502	-0.20148	-0.7401	0.459845	0.797917	chr20	57463991
cg09157251	-0.10501	-0.73987	0.459984	0.797977	chr11	70733251
cg25316853	-0.02863	-0.73741	0.461477	0.798974	chr5	36606347
cg12124094	0.022242	0.737064	0.461687	0.799081	chr17	64433744
cg16862319	0.027833	0.735687	0.462524	0.799543	chr12	13899195
cg03777288	-0.01627	-0.7342	0.463431	0.800182	chr12	13717033
cg24737505	0.021892	0.734053	0.463518	0.800224	chr11	70564116
cg05716556	0.012307	0.731853	0.464857	0.800834	chr17	47287410
cg20955817	-0.01506	-0.73178	0.464903	0.800865	chr11	70936560
cg25293328	0.030262	0.73176	0.464914	0.800881	chr19	2611690
cg05722993	-0.01627	-0.73152	0.46506	0.800971	chr1	1727796
cg20788479	0.018602	0.731482	0.465084	0.80098	chr3	179169536
cg11676382	0.022084	0.731097	0.465318	0.801168	chr22	51141829
cg19161850	0.047779	0.73083	0.465481	0.801238	chr22	22222040
cg27027803	-0.0147	-0.73062	0.465607	0.801287	chr20	57464742
cg14791525	0.044397	0.730213	0.465857	0.801443	chr11	70732224
cg21114126	-0.0329	-0.72988	0.466059	0.801578	chr4	102267189
cg25326570	-0.02534	-0.72967	0.466189	0.801634	chr20	57426757
cg13286510	0.022081	0.729259	0.46644	0.801749	chr7	126893007
cg26235243	-0.01526	-0.72378	0.469793	0.803821	chr6	33656836
cg23369234	-0.01779	-0.72283	0.470375	0.804146	chr12	2511478
cg07783800	0.021972	0.722708	0.47045	0.804177	chr1	235803662
cg13939602	0.02431	0.721699	0.471069	0.80456	chr12	2467410
cg20698170	-0.02502	-0.72061	0.471737	0.804968	chr15	42120362
cg19653589	0.021824	0.719596	0.472361	0.80525	chr19	2614177
cg11539664	-0.02384	-0.71799	0.473349	0.805769	chr6	33604664
cg15144016	0.025931	0.717491	0.473657	0.805933	chr3	51749782
cg08685096	0.023496	0.717331	0.473755	0.805955	chr21	31312643
cg27541048	-0.01712	-0.71732	0.473762	0.805956	chr12	6954816

cg13570585	0.020309	0.716654	0.474172	0.806139	chr20	8113573
cg24591182	0.050688	0.7161	0.474514	0.806378	chr11	64019217
cg09444818	0.024941	0.712887	0.476497	0.807492	chr4	102097226
cg00341980	0.019367	0.711635	0.477271	0.807932	chr1	84544220
cg00800141	-0.02417	-0.70942	0.478643	0.808725	chr16	4014161
cg07823688	0.034634	0.708197	0.4794	0.809118	chr16	56310091
cg15802396	-0.02198	-0.70732	0.479945	0.809349	chr17	7108305
cg26999577	0.020216	0.706675	0.480344	0.809556	chr12	2340831
cg05492714	0.02323	0.706644	0.480363	0.809567	chr9	140042286
cg26648054	-0.02873	-0.70566	0.480972	0.809892	chr17	7117995
cg11661914	0.042971	0.705636	0.480989	0.809907	chr12	49180849
cg12872693	0.025912	0.705121	0.481309	0.810135	chr3	179168798
cg09286797	0.024316	0.703723	0.482178	0.810536	chr20	8116999
cg06432462	-0.02446	-0.70317	0.482522	0.810791	chr9	80646879
cg01449218	0.017148	0.702752	0.482782	0.810954	chr7	86273083
cg19015708	-0.02099	-0.70149	0.483567	0.811342	chr2	68418292
cg25185429	0.012706	0.699797	0.484623	0.811878	chr3	4739461
cg13714844	-0.01942	-0.6989	0.485183	0.812159	chr9	114422486
cg11706467	-0.02732	-0.69819	0.485623	0.812432	chr2	155554707
cg23913904	-0.02332	-0.69767	0.485948	0.812662	chr11	70590130
cg20809470	0.019091	0.697655	0.485959	0.812668	chr11	22364837
cg17960347	-0.01702	-0.69707	0.486327	0.812923	chr12	2457373
cg16086007	0.021703	0.694852	0.487712	0.813647	chr17	72855588
cg02392737	-0.01873	-0.69419	0.488126	0.813822	chr16	4136367
cg18846074	-0.03584	-0.69376	0.488395	0.813924	chr14	24801073
cg24432193	-0.02938	-0.69313	0.48879	0.814155	chr6	102062733
cg14317712	0.021741	0.693007	0.488867	0.814199	chr9	140034900
cg17871403	-0.01607	-0.69255	0.489155	0.814328	chr5	7827115
cg09241929	-0.03632	-0.69205	0.489468	0.814531	chr20	57465560
cg09787442	0.020738	0.688705	0.491567	0.815791	chr21	31119294
cg14851284	-0.02659	-0.68824	0.491859	0.81594	chr11	70713732
cg27591117	0.023073	0.687803	0.492134	0.816073	chr20	8113191
cg22967080	0.014473	0.686969	0.492659	0.816303	chr14	52333365
cg04169369	0.017136	0.686811	0.492758	0.816373	chr16	4053199
cg06986989	0.016842	0.686399	0.493017	0.816496	chr1	235802839
cg14263118	-0.01275	-0.68546	0.49361	0.81675	chr20	57463787
cg09845015	0.013046	0.685352	0.493677	0.816779	chr11	70458994
cg27356165	-0.02257	-0.6848	0.494023	0.817006	chr19	2613933
cg16475558	0.030062	0.68174	0.495955	0.817959	chr16	56388945
cg08460548	0.023365	0.681664	0.496003	0.817985	chr19	49944817
cg24218925	-0.01526	-0.68141	0.496163	0.818035	chr19	2578938
cg02959759	0.033775	0.681088	0.496367	0.818152	chr12	2801584
cg05795849	0.021013	0.680831	0.496529	0.818237	chr3	4794082
cg14829063	-0.0157	-0.68017	0.496947	0.81842	chr11	70731587
cg00043510	0.024658	0.679988	0.497062	0.818463	chr1	235814134
cg06185738	-0.02021	-0.67867	0.497895	0.818943	chr11	22359868
cg14111579	-0.01569	-0.67856	0.497968	0.818979	chr19	48614090
cg09604333	0.008103	0.678288	0.498137	0.819092	chr20	57465125

cg07482508	0.022688	0.67771	0.498503	0.819271	chr5	36606981		
cg09742895	-0.01672	-0.67718	0.498838	0.819477	chr11	105781302		
cg02295678	-0.02266	-0.67632	0.499386	0.819763	chr17	7123415		
cg08966293	0.018391	0.674854	0.500314	0.820314	chr16	30134858		
cg04364463	-0.01318	-0.6741	0.500795	0.820558	chr1	37498270		
cg16835502	-0.01922	-0.67406	0.500819	0.820564	chr17	4710020		
cg05616819	-0.02696	-0.67406	0.50082	0.820564	chr16	24231485		
cg16480969	-0.0133	-0.6715	0.502446	0.821409	chr6	33996203		
cg04747226	0.052517	0.670511	0.503073	0.821766	chr11	105481319		
cg13052954	0.02424	0.670451	0.503111	0.821792	chr1	37467416		
cg22798925	-0.00964	-0.66814	0.504585	0.82242	chr20	57464129		
cg14926715	-0.01239	-0.66687	0.50539	0.822918	chr11	70318919		
cg16543027	0.017982	0.665381	0.506343	0.82338	chr15	40599680		
cg24067803	-0.0134	-0.66493	0.506631	0.823531	chr11	70653378		
cg27068206	-0.01871	-0.6646	0.50684	0.823679	chr11	70559053		
cg10804438	0.016328	0.664365	0.506992	0.82376	chr3	51747196		
cg06739873	0.017528	0.66415	0.507129	0.823858	chr19	19040289		
cg16177440	-0.01424	-0.66395	0.507259	0.82391	chr7	100275304		
cg05340882	0.036105	0.66334	0.507647	0.824107	chr19	2543750		
cg07851738	-0.01707	-0.66011	0.509714	0.825397	chr8	131896788		
cg19707326	-0.01372	-0.65952	0.510094	0.8256	chr14	24787611		
cg04898487	0.020432	0.659193	0.510303	0.825696	chr16	10272607		
cg01007458	-0.01437	-0.65686	0.511796	0.826558	chr19	15084527		
cg21599324	-0.01097	-0.65676	0.511861	0.826578	chr6	34074282		
cg04798490	0.01677	0.655598	0.51261	0.826913	chr11	70517045		
cg08505135	0.012256	0.65452	0.513303	0.827295	chr19	19045615		
cg09822192	-0.03036	-0.6533	0.514087	0.827738	chr14	24801191		
cg08263099	-0.04668	-0.65218	0.514812	0.828103	chr19	54410160		
cg07559526	-0.01692	-0.65216	0.514823	0.828103	chr16	4164735		
cg04742605	-0.01364	-0.65168	0.515131	0.828222	chr11	70385511		
cg06147822	-0.02915	-0.65138	0.515324	0.828317	chr20	57466905		
cg18379295	0.014088	0.651134	0.515483	0.828415	chr14	52326155		
cg16803737	-0.01383	-0.65051	0.515888	0.828602	chr6	33592658		
cg14232870	0.028339	0.649195	0.516733	0.829049	chr11	70458782		
cg04993286	0.019548	0.648695	0.517056	0.829194	chr16	4027595		
cg04005969	-0.01818	-0.64861	0.517108	0.829241	chr19	51171247		
cg07340423	0.018028	0.647224	0.518006	0.829612	chr15	52472383		
cg14022022	-0.02327	-0.64716	0.518047	0.829635	chr9	140055728		
cg04153722	-0.02148	-0.64673	0.518324	0.829761	chr17	64783041		
cg10801143	-0.01637	-0.6439	0.520155	0.830638	chr11	88245465		
cg15877314	0.018671	0.643805	0.520218	0.83066	chr3	50273895		
cg08001559	-0.01689	-0.64371	0.52028	0.830707	chr14	52326905		
cg12363682	0.022424	0.642351	0.52116	0.831117	chr5	178420690		
cg22484822	0.017781	0.641086	0.52198	0.831474	chr6	33996111		
cg12282391	0.018702	0.638921	0.523385	0.832109	chr12	2162491	1stExon	1stExon
cg12089094	0.013665	0.636744	0.524801	0.832736	chr3	171463962		
cg16358215	-0.03741	-0.6354	0.525673	0.833282	chr11	70455662		
cg01348055	0.013577	0.633196	0.527112	0.834099	chr16	10272788		

cg10707626	-0.01583	-0.63303	0.527222	0.834159	chr3	51747098
cg22522961	0.023873	0.63242	0.527618	0.834354	chr1	68288817
cg05725666	-0.01264	-0.63122	0.528401	0.83481	chr12	2224644
cg27122965	0.018443	0.631041	0.528518	0.834874	chr11	70882053
cg05166022	-0.01901	-0.6307	0.528739	0.83501	chr12	2800471
cg06697294	0.041911	0.630627	0.528788	0.835011	chr19	54385412
cg02640558	-0.01924	-0.62945	0.529558	0.835414	chr8	22299141
cg13980113	0.019064	0.62941	0.529583	0.83542	chr5	36607333
cg02327001	0.02951	0.629113	0.529777	0.835506	chr4	101972762
cg11265916	-0.01248	-0.62396	0.533154	0.837164	chr22	22221056
cg09853822	-0.01505	-0.62341	0.533513	0.837357	chr17	4712456
cg19572362	0.012675	0.621676	0.53465	0.838136	chr1	110091012
cg20490175	-0.01289	-0.62159	0.534706	0.838171	chr12	2797745
cg25983380	-0.03761	-0.62085	0.535194	0.838358	chr20	57465439
cg19151292	-0.01226	-0.61961	0.536011	0.838743	chr6	33653502
cg01895374	-0.0159	-0.61936	0.536171	0.838796	chr17	64536954
cg09407429	-0.01273	-0.61889	0.536483	0.838921	chr3	4534383
cg26345619	0.016412	0.61836	0.53683	0.839102	chr12	2602405
cg06466348	-0.01282	-0.61747	0.537414	0.839382	chr16	50337922
cg16261581	-0.0217	-0.61701	0.537719	0.8395	chr1	84972327
cg22741626	0.018822	0.616902	0.53779	0.839571	chr20	57463265
cg12566890	-0.01172	-0.6166	0.537988	0.839635	chr5	7494531
cg04367107	-0.02255	-0.6153	0.538849	0.840018	chr11	88238963
cg07713849	0.009763	0.614304	0.539503	0.840361	chr6	33624841
cg00146655	-0.01263	-0.61178	0.541172	0.84099	chr3	7517194
cg05060704	0.020202	0.61093	0.541731	0.841299	chr3	50275694
cg14235271	0.018903	0.61066	0.54191	0.841368	chr20	57462812
cg16124935	-0.02504	-0.61051	0.542009	0.841425	chr11	70559616
cg19196684	-0.01278	-0.60977	0.542496	0.841652	chr1	53608037
cg21409965	-0.02336	-0.60936	0.542767	0.841784	chr1	37283904
cg15093766	0.018126	0.608374	0.543422	0.842185	chr17	64408569
cg21673873	-0.01736	-0.60608	0.544941	0.84293	chr11	70511688
cg22600443	-0.01955	-0.60545	0.545361	0.843063	chr15	42388240
cg08458678	0.01217	0.605146	0.545562	0.843141	chr11	70565177
cg08525508	-0.0104	-0.60371	0.546515	0.843562	chr19	48920297
cg01347776	0.015249	0.603553	0.546619	0.84361	chr3	179121836
cg02515133	0.017944	0.60346	0.546681	0.843633	chr7	86415687
cg12986110	0.016245	0.602661	0.547212	0.843925	chr19	48551504
cg09529437	0.012648	0.602555	0.547282	0.843971	chr16	24136792
cg10331829	0.015669	0.602242	0.54749	0.84411	chr11	35343789
cg18080819	-0.01722	-0.60102	0.548303	0.844471	chr11	70505972
cg08123425	0.021682	0.599886	0.549057	0.844831	chr12	13939517
cg10509626	-0.02353	-0.59952	0.549302	0.844943	chr11	70333993
cg13948330	-0.01241	-0.5993	0.549446	0.845049	chr11	70559064
cg11221524	0.016837	0.597067	0.550935	0.845819	chr1	84969091
cg16281600	-0.01824	-0.59672	0.55117	0.845933	chr5	152869431
cg02433656	0.016461	0.596181	0.551526	0.846116	chr16	56322654
cg09066361	0.036911	0.596065	0.551603	0.84615	chr7	126890254

cg12312205	0.012406	0.594625	0.552565	0.846645	chr18	3594173
cg21323244	-0.01292	-0.5919	0.554387	0.847359	chr11	70415439
cg17069533	-0.01025	-0.5916	0.554588	0.847465	chr17	64657833
cg07938763	-0.01549	-0.58912	0.556247	0.84826	chr19	2516966
cg17696044	-0.01977	-0.58897	0.556346	0.848289	chr11	70449316
cg24671939	-0.01646	-0.58874	0.556498	0.848342	chr18	3593798
cg18845797	-0.01592	-0.5887	0.55653	0.84835	chr5	7794275
cg12054318	-0.00968	-0.58835	0.556761	0.848467	chr20	57414529
cg07024458	0.015905	0.587568	0.557286	0.848678	chr16	56390600
cg26454299	-0.01837	-0.58688	0.557746	0.848924	chr4	102268957
cg24183324	-0.01678	-0.58515	0.558911	0.849547	chr11	35440062
cg21216562	-0.02298	-0.58306	0.560314	0.850196	chr9	140045508
cg08576827	-0.02655	-0.58299	0.560362	0.850232	chr20	9075493
cg24946911	0.02294	0.582651	0.560587	0.850315	chr12	2788654
cg22218695	-0.01416	-0.58099	0.561704	0.850927	chr7	126446519
cg22989419	-0.01905	-0.58081	0.561829	0.850966	chr20	9340396
cg16829998	0.018741	0.579746	0.562542	0.851354	chr19	49944964
cg09461286	0.015424	0.579604	0.562638	0.851388	chr16	10276081
cg00610508	0.025552	0.578528	0.563363	0.851695	chr16	4013337
cg04251662	-0.01513	-0.57677	0.564545	0.852245	chr3	4535075
cg13371705	-0.0118	-0.57487	0.565833	0.852842	chr12	2452955
cg25961618	0.014122	0.572078	0.567718	0.853817	chr11	35360531
cg25562925	0.008694	0.571067	0.568403	0.85415	chr2	25110049
cg25423647	-0.01231	-0.57075	0.568615	0.85426	chr3	51746723
cg00417823	0.012794	0.57075	0.568617	0.85426	chr16	56330268
cg09300795	0.021325	0.570138	0.569032	0.854472	chr16	4042428
cg00534626	-0.0246	-0.57003	0.569102	0.854483	chr16	9864730
cg25193077	0.021942	0.569573	0.569414	0.854651	chr1	235812109
cg23399933	0.02791	0.568777	0.569954	0.854822	chr4	102112217
cg06047881	0.01202	0.568687	0.570015	0.854873	chr20	57465132
cg15651980	-0.01474	-0.56681	0.571286	0.85546	chr19	48903304
cg25350198	0.021668	0.566092	0.571775	0.855655	chr1	84971850
cg06692957	0.015955	0.565383	0.572257	0.855917	chr9	80647629
cg09152120	-0.01782	-0.56533	0.572293	0.85592	chr16	4016669
cg08370077	0.017958	0.564769	0.572674	0.856058	chr16	851288
cg26739691	-0.01397	-0.56444	0.572894	0.856229	chr7	45637270
cg16737409	-0.01915	-0.56387	0.573287	0.856324	chr20	57428366
cg07451034	0.014264	0.563235	0.573716	0.85656	chr17	47283774
cg12981270	-0.01375	-0.56314	0.573783	0.856581	chr19	42509946
cg19256368	-0.0124	-0.56245	0.574249	0.856802	chr15	83619037
cg09179079	0.025232	0.560849	0.57534	0.857284	chr6	146348690
cg17119568	0.020945	0.56023	0.575761	0.857433	chr19	14229237
cg26764244	-0.02365	-0.55801	0.577276	0.858062	chr1	68299511
cg15083678	0.011279	0.557913	0.577341	0.858103	chr12	2724200
cg10306450	-0.01687	-0.55535	0.579088	0.858864	chr12	56882479
cg24723883	-0.01038	-0.55481	0.579458	0.85909	chr19	2608495
cg22857947	0.020583	0.554502	0.579669	0.859251	chr19	14225039
cg08464513	-0.01448	-0.55434	0.579782	0.859305	chr16	30136024

cg06210447	-0.01928	-0.55253	0.581016	0.859923	chr11	70601842
cg13851211	0.011353	0.551438	0.581764	0.860314	chr16	50321678
cg16108726	-0.02103	-0.55081	0.582197	0.860479	chr11	70781009
cg20830447	0.018789	0.5502	0.582612	0.86071	chr12	46764929
cg04779428	0.019898	0.548494	0.583781	0.861301	chr20	57463355
cg07790747	0.015883	0.546992	0.584811	0.861831	chr16	56266223
cg20782596	-0.0126	-0.54661	0.585076	0.861961	chr20	57462978
cg22065976	0.013279	0.544556	0.586483	0.862674	chr6	33589061
cg11985287	0.011128	0.543348	0.587313	0.863087	chr19	2556805
cg27428129	-0.01205	-0.54279	0.587696	0.863261	chr6	34051095
cg20288565	0.030818	0.542211	0.588095	0.86344	chr1	235805403
cg05558390	-0.01138	-0.54178	0.588391	0.863576	chr20	57415377
cg08897844	-0.02209	-0.54013	0.589527	0.864112	chr5	36606102
cg18175690	-0.0168	-0.53997	0.58964	0.864146	chr15	40580770
cg06728579	-0.03187	-0.53996	0.589643	0.864146	chr16	56224901
cg03412431	0.013886	0.539265	0.590123	0.86437	chr6	34023418
cg27567416	-0.01647	-0.53866	0.590543	0.864524	chr16	4117281
cg14843888	-0.01555	-0.53702	0.591672	0.865042	chr3	53530247
cg03606258	-0.06355	-0.5361	0.592309	0.865363	chr20	57426935
cg02640306	0.01558	0.536015	0.592365	0.865363	chr2	191745287
cg09947844	-0.01372	-0.53361	0.594027	0.866041	chr16	4163819
cg24363298	0.014478	0.533247	0.594276	0.866194	chr3	50242671
cg11244758	-0.00895	-0.53314	0.594353	0.866218	chr20	57463900
cg13343565	-0.03356	-0.53149	0.59549	0.866853	chr17	4710032
cg17540496	0.013304	0.530412	0.596238	0.867124	chr3	53845930
cg17202839	-0.0145	-0.52928	0.597019	0.867511	chr17	64685036
cg08505222	-0.01472	-0.52915	0.597115	0.867545	chr22	51139277
cg23894980	-0.01012	-0.5284	0.597635	0.867799	chr11	70534718
cg08411235	0.009799	0.527314	0.598384	0.868175	chr11	35297026
cg10206594	-0.02169	-0.52716	0.598488	0.868239	chr1	37314665
cg02954212	0.016406	0.527082	0.598545	0.868287	chr16	56226350
cg01246398	-0.01157	-0.52697	0.59862	0.868324	chr3	123165872
cg05884705	0.015479	0.526269	0.599109	0.86859	chr15	40600099
cg13355041	-0.01475	-0.52488	0.600074	0.868975	chr18	3593715
cg03641740	-0.0252	-0.52402	0.600671	0.869179	chr4	102087758
cg12413242	0.007903	0.523819	0.60081	0.869198	chr12	2292890
cg17038626	0.017955	0.523141	0.601281	0.869383	chr12	2435931
cg01637841	0.011199	0.522844	0.601488	0.869458	chr3	7610381
cg23519329	0.014109	0.522811	0.60151	0.869458	chr16	4166914
cg24036292	-0.01197	-0.52147	0.602445	0.869845	chr11	70416269
cg24791862	0.011846	0.52115	0.602666	0.869979	chr7	126893237
cg17024257	-0.01214	-0.52036	0.603214	0.870182	chr3	171528758
cg01386883	-0.01222	-0.52023	0.603306	0.870224	chr3	53529144
cg06310285	-0.01278	-0.51918	0.604036	0.870628	chr3	53796066
cg27555529	0.020318	0.518228	0.6047	0.870939	chr19	13617518
cg04498349	0.021524	0.516511	0.605896	0.871596	chr16	10274317
cg21422400	-0.01372	-0.51639	0.605983	0.871616	chr1	1747243
cg02794451	0.013927	0.516057	0.606213	0.871707	chr12	2800446

cg23475045	-0.01092	-0.5151	0.606879	0.872047	chr3	7248510
cg07813421	0.015584	0.513708	0.607853	0.87256	chr17	7123626
cg03162045	0.014234	0.512043	0.609016	0.873107	chr3	142443933
cg24863175	0.021491	0.510829	0.609865	0.873437	chr1	186840433
cg22920586	-0.01324	-0.51063	0.610004	0.873496	chr3	171472629
cg21429394	0.018009	0.509571	0.610745	0.873863	chr12	100750899
cg10243075	-0.02005	-0.50917	0.611029	0.873947	chr19	13615439
cg20000940	0.011146	0.507742	0.612026	0.874385	chr14	52327486
cg27073262	-0.02244	-0.50764	0.612098	0.874407	chr7	86493792
cg03866831	0.024244	0.506205	0.613103	0.874799	chr16	9849427
cg04348872	0.01223	0.5051	0.613878	0.875094	chr2	25141696
cg22518097	-0.02189	-0.50498	0.61396	0.875123	chr4	101948183
cg16128363	-0.01069	-0.50262	0.615621	0.875751	chr18	3880558
cg06163629	0.009322	0.500652	0.617001	0.876401	chr20	57414884
cg13830799	-0.01528	-0.49944	0.617855	0.876614	chr6	146750462
cg00765653	0.005757	0.498962	0.618189	0.876791	chr20	57415144
cg03547757	-0.01279	-0.49882	0.61829	0.876838	chr20	57425515
cg11544138	-0.01037	-0.49871	0.618364	0.876867	chr19	1003455
cg14648237	-0.0088	-0.49848	0.618527	0.876966	chr17	64422393
cg09433558	-0.01716	-0.49829	0.618661	0.877032	chr3	171412917
cg26379672	-0.01164	-0.49674	0.619753	0.877465	chr3	4534954
cg24043604	-0.01085	-0.49641	0.619989	0.87754	chr12	2613948
cg06419562	0.013474	0.496309	0.620058	0.877568	chr16	24148747
cg11637718	-0.00971	-0.49574	0.620456	0.877748	chr16	4029254
cg00671386	0.015364	0.495391	0.620704	0.877842	chr16	851834
cg02105211	-0.0137	-0.49461	0.621252	0.878108	chr3	4625188
cg11357538	-0.00768	-0.49344	0.622079	0.878446	chr20	57463397
cg22632947	0.01085	0.4933	0.622179	0.878468	chr17	64787784
cg06532779	0.01808	0.492384	0.622826	0.878718	chr19	1003622
cg06783533	-0.01371	-0.49172	0.623297	0.878923	chr16	56388908
cg09858208	0.016183	0.491476	0.623467	0.879022	chr3	50283852
cg14746605	-0.01522	-0.49121	0.623656	0.879084	chr11	35374336
cg06137273	-0.026	-0.49054	0.624128	0.879289	chr3	6905031
cg00791406	-0.01648	-0.49047	0.624174	0.879289	chr12	14118880
cg03630683	0.013822	0.490027	0.624491	0.879318	chr3	4534997
cg24882525	-0.01124	-0.48986	0.624609	0.87937	chr11	70565226
cg15805568	0.014953	0.48976	0.624679	0.87937	chr19	51199000
cg27491190	-0.01089	-0.48566	0.627582	0.880752	chr12	46767943
cg05035616	0.012061	0.485275	0.627852	0.880886	chr16	56374523
cg19365406	0.017221	0.483917	0.628815	0.88137	chr9	140043007
cg25407736	0.021169	0.483508	0.629105	0.881483	chr1	68296179
cg09146232	0.012041	0.483441	0.629152	0.881496	chr17	64672129
cg07178968	-0.00933	-0.48314	0.629365	0.881606	chr15	42130662
cg10474377	0.012254	0.483025	0.629447	0.881619	chr15	42131658
cg24000444	0.020931	0.482705	0.629674	0.88172	chr18	3771452
cg13280108	0.014951	0.481924	0.630228	0.881888	chr11	70398751
cg03679394	-0.01641	-0.48172	0.63037	0.881944	chr11	70516997
cg25960479	-0.01103	-0.47858	0.632604	0.882758	chr11	88243569

cg11647651	0.021118	0.478169	0.632895	0.882907	chr17	4710373
cg06401532	-0.01289	-0.47799	0.633023	0.88296	chr16	24220008
cg24086869	0.012088	0.477914	0.633077	0.882987	chr15	52471581
cg11971789	0.010171	0.476821	0.633854	0.88334	chr19	42545623
cg09722397	0.01192	0.47674	0.633911	0.883357	chr17	72855943
cg08644463	0.014255	0.474877	0.635237	0.883851	chr1	110106962
cg09257092	-0.01898	-0.4742	0.635722	0.884061	chr12	26986805
cg22335074	0.015719	0.472791	0.636723	0.884536	chr11	70733258
cg19251850	0.014558	0.472315	0.637062	0.884677	chr3	171428254
cg21233003	0.019136	0.471829	0.637408	0.884756	chr9	140057464
cg11836212	0.018536	0.471475	0.637661	0.884867	chr1	37447865
cg19577617	0.012672	0.471251	0.637821	0.884927	chr2	191745301
cg05147077	0.014508	0.470752	0.638176	0.885019	chr5	36606601
cg03657031	0.011401	0.469269	0.639235	0.885277	chr19	54385215
cg03510435	0.017235	0.467315	0.64063	0.885779	chr12	14094558
cg26160180	0.034531	0.466956	0.640887	0.885924	chr1	1822883
cg02725014	0.013393	0.46581	0.641706	0.886282	chr5	78809520
cg01538522	0.00911	0.465585	0.641867	0.886319	chr20	57463974
cg07746960	0.017449	0.465477	0.641944	0.886342	chr19	42546662
cg25983305	-0.01727	-0.46436	0.642745	0.886787	chr8	22298586
cg03697708	0.017975	0.464149	0.642894	0.886839	chr19	13617549
cg26279745	-0.02214	-0.46362	0.643269	0.886937	chr14	24801970
cg07699277	0.011244	0.463597	0.643289	0.886937	chr6	34004226
cg17752088	-0.01758	-0.46205	0.644396	0.88743	chr5	78810367
cg16890681	0.013906	0.46109	0.645084	0.887733	chr2	68479269
cg02602411	-0.01017	-0.46062	0.64542	0.887818	chr9	104357177
cg22133366	-0.01497	-0.46032	0.645636	0.887926	chr11	70385327
cg21163960	0.025867	0.458219	0.647143	0.888556	chr11	35441777
cg15995075	-0.01194	-0.45738	0.647747	0.888781	chr3	142451487
cg07391392	-0.01376	-0.45661	0.648301	0.88902	chr5	7826900
cg02624701	-0.02731	-0.45639	0.648458	0.889066	chr19	49937176
cg02500883	-0.01165	-0.45629	0.648528	0.889081	chr9	104356619
cg16359985	-0.01012	-0.45613	0.648643	0.889149	chr7	100276087
cg03070741	0.011765	0.456022	0.64872	0.889203	chr19	2650727
cg12191293	0.01117	0.455723	0.648935	0.889281	chr12	56882314
cg08111863	-0.01059	-0.45453	0.649789	0.889587	chr11	70882036
cg08374499	0.018643	0.453648	0.650426	0.889823	chr19	49941241
cg09868882	0.010185	0.453645	0.650428	0.889823	chr7	126883640
cg25894071	0.021093	0.451849	0.65172	0.89043	chr4	101953935
cg22692013	-0.01149	-0.45024	0.652881	0.890894	chr12	2354618
cg13533759	-0.01007	-0.44922	0.653617	0.891224	chr9	114431056
cg01130792	0.018387	0.449163	0.653654	0.891243	chr22	51112683
cg26626089	0.01537	0.449051	0.653735	0.891282	chr19	54385865
cg06200857	-0.02157	-0.44878	0.653931	0.891342	chr20	57426420
cg00233948	0.011741	0.448045	0.65446	0.891542	chr5	36619356
cg03723730	0.008359	0.447999	0.654493	0.891559	chr6	34031694
cg14416930	0.011992	0.447281	0.65501	0.891668	chr17	64498178
cg13641156	0.01522	0.447238	0.655042	0.891675	chr11	70515650

cg11663780	0.010759	0.446527	0.655554	0.891957	chr19	1001892
cg15972294	0.014089	0.444726	0.656854	0.892531	chr3	50273096
cg23686556	0.025398	0.443832	0.657499	0.892778	chr8	131961316
cg16361867	-0.01457	-0.44355	0.657705	0.892861	chr11	120581355
cg11169463	0.010255	0.442868	0.658196	0.893044	chr6	33653411
cg10002103	0.016941	0.441253	0.659363	0.893448	chr12	46766730
cg26568075	-0.0105	-0.43993	0.660319	0.893859	chr1	1718809
cg12446629	0.015068	0.439521	0.660616	0.893969	chr8	132052044
cg02840199	-0.01264	-0.43952	0.66062	0.893969	chr16	4165257
cg10487659	-0.01187	-0.43744	0.662125	0.89456	chr16	4152081
cg11996914	-0.00493	-0.43742	0.662137	0.894563	chr20	57414578
cg07284407	-0.00793	-0.43485	0.663997	0.895347	chr20	57429858
cg17151604	0.012034	0.43365	0.66487	0.895695	chr10	75197928
cg09239744	0.016526	0.433606	0.664901	0.895703	chr16	10276580
cg03193168	-0.01006	-0.43296	0.665367	0.895915	chr22	51159995
cg08619378	-0.00914	-0.43288	0.665431	0.895926	chr7	45616358
cg10668781	-0.00891	-0.4325	0.665703	0.896049	chr12	2307325
cg12691534	0.009942	0.432416	0.665765	0.896053	chr3	50275394
cg10181414	-0.02067	-0.43094	0.666834	0.896541	chr19	2546598
cg01047778	-0.00911	-0.43072	0.666999	0.89662	chr11	70584252
cg20401058	-0.00876	-0.42968	0.667748	0.89695	chr20	57426240
cg17214089	0.01052	0.428477	0.668626	0.897267	chr1	182354912
cg01091261	-0.01559	-0.42792	0.669029	0.897403	chr16	4029363
cg07824914	-0.01105	-0.4271	0.66963	0.897701	chr20	57465815
cg05484458	0.009574	0.424098	0.671813	0.898496	chr12	6949260
cg20741386	-0.00925	-0.42393	0.671938	0.89858	chr1	53556189
cg05414613	-0.01189	-0.42204	0.673314	0.899148	chr1	53558470
cg27369641	0.009354	0.421378	0.673795	0.899315	chr7	100274361
cg25900614	0.021617	0.420961	0.6741	0.899381	chr7	126079083
cg24868926	-0.01942	-0.42022	0.674642	0.899563	chr1	182360594
cg06646622	0.014364	0.419739	0.674991	0.899707	chr5	78766902
cg26429499	0.015777	0.419412	0.67523	0.899754	chr11	70563792
cg26381514	-0.01365	-0.41845	0.675934	0.900041	chr12	26963489
cg07091154	0.009618	0.417734	0.676455	0.900288	chr11	70562728
cg10283969	-0.00847	-0.4174	0.676698	0.900428	chr11	70666639
cg02224372	-0.01402	-0.41709	0.676925	0.900482	chr11	70858695
cg02607130	0.013136	0.41704	0.676962	0.900496	chr19	1008643
cg18619398	0.006165	0.416392	0.677435	0.900622	chr20	57416506
cg14943539	-0.01323	-0.41589	0.677803	0.900812	chr20	57420942
cg21462934	-0.01323	-0.41519	0.678311	0.901041	chr19	2622858
cg13298384	-0.00953	-0.41506	0.678409	0.901095	chr19	42546648
cg04106389	0.009894	0.413933	0.679233	0.901499	chr17	7117241
cg08578734	0.01278	0.41337	0.679646	0.901659	chr5	152870490
cg22746789	0.010931	0.413035	0.679891	0.901709	chr19	2579221
cg08992229	0.01756	0.410559	0.681703	0.902416	chr7	126866923
cg10504751	-0.02306	-0.41036	0.68185	0.902448	chr16	56390830
cg14728235	0.005639	0.408748	0.683031	0.902772	chr20	57415177
cg03344105	0.006297	0.407759	0.683756	0.903151	chr20	57426131

cg02423534	0.006805	0.40698	0.684327	0.903408	chr12	49160180
cg16812352	0.008813	0.406464	0.684706	0.903509	chr16	4049855
cg06887224	-0.00849	-0.4058	0.685193	0.903754	chr5	7399037
cg03650282	0.012211	0.405437	0.685459	0.903877	chr17	64451448
cg17074573	0.010052	0.40262	0.687529	0.904692	chr22	51165537
cg12128893	0.007901	0.402529	0.687596	0.904728	chr10	75255807
cg13936125	-0.00995	-0.40224	0.687809	0.904815	chr16	56225599
cg24092939	-0.0159	-0.40221	0.687829	0.90482	chr12	49181056
cg08266286	-0.00836	-0.40213	0.687889	0.904852	chr2	25141901
cg27630678	-0.00844	-0.40211	0.687901	0.904853	chr11	70565201
cg15122327	-0.01561	-0.40137	0.688446	0.905098	chr20	57435146
cg25456593	-0.0274	-0.40124	0.688545	0.9051	chr11	70672858
cg11464074	0.010591	0.401184	0.688584	0.905107	chr7	126417126
cg26904140	-0.0134	-0.39983	0.689582	0.905493	chr19	2703086
cg27529848	-0.01077	-0.39974	0.689649	0.905502	chr15	52472330
cg00925020	0.013405	0.398631	0.690463	0.905814	chr19	2543877
cg22454005	0.01036	0.397803	0.691072	0.906076	chr19	54392916
cg08193910	0.010236	0.397739	0.691119	0.906096	chr19	54410103
cg26968767	0.010302	0.397213	0.691507	0.906202	chr17	72843650
cg22885821	0.0115	0.396026	0.692381	0.906527	chr20	57465921
cg11814875	0.014246	0.39426	0.693683	0.907037	chr19	2611237
cg13707945	0.014253	0.391421	0.695777	0.907998	chr3	4714992
cg04260676	0.007916	0.389358	0.697301	0.908529	chr1	1774322
cg04279973	0.009421	0.38819	0.698164	0.908898	chr16	23846968
cg13180375	-0.01179	-0.38708	0.698987	0.909124	chr8	22298119
cg02892153	0.01159	0.386311	0.699553	0.909308	chr18	3593461
cg04266169	-0.0102	-0.38612	0.699693	0.909341	chr1	1822862
cg26266429	0.006213	0.38576	0.699961	0.909424	chr12	49174651
cg06456864	0.013859	0.384556	0.700852	0.909755	chr6	34101399
cg15436476	0.009139	0.383731	0.701463	0.909903	chr19	2626283
cg21988465	0.008254	0.383172	0.701877	0.910075	chr20	57429277
cg01900555	-0.00636	-0.38316	0.701884	0.910075	chr12	6948846
cg11727252	-0.00672	-0.38199	0.70275	0.910483	chr3	51752143
cg05960039	0.006163	0.381982	0.702758	0.910483	chr20	57465123
cg16175911	-0.01065	-0.37902	0.704955	0.911337	chr12	26985133
cg02521996	-0.01762	-0.37822	0.70555	0.911589	chr16	30134825
cg18162783	0.009484	0.377614	0.705997	0.911838	chr1	1795891
cg12913957	0.012962	0.377143	0.706347	0.912028	chr6	33589131
cg18950779	0.010992	0.375798	0.707346	0.912455	chr17	64685057
cg14872036	-0.01009	-0.37554	0.707534	0.912544	chr3	123049031
cg01846046	0.01423	0.374702	0.70816	0.912801	chr11	64034019
cg18411150	0.01093	0.374508	0.708304	0.912844	chr19	51172144
cg09447435	-0.01003	-0.37335	0.709167	0.913232	chr6	101854480
cg03871526	-0.01935	-0.37318	0.709288	0.913279	chr3	142447949
cg25557995	-0.01092	-0.37227	0.709966	0.913559	chr12	2761091
cg24825722	-0.00761	-0.37159	0.710474	0.913786	chr17	7121848
cg14910395	0.006329	0.37141	0.710608	0.913807	chr19	48941986
cg09031790	-0.00836	-0.37097	0.710932	0.913982	chr16	24129948

cg10106561	-0.00816	-0.36969	0.71189	0.914303	chr2	25050913
cg17106653	-0.01172	-0.36946	0.712055	0.914395	chr19	48897279
cg03011594	0.014174	0.369067	0.712351	0.914531	chr16	56370697
cg01697794	0.01289	0.369047	0.712366	0.914539	chr17	7117125
cg03489495	0.011095	0.36879	0.712557	0.914609	chr6	33588875
cg06173536	0.010971	0.368761	0.712579	0.914614	chr1	235814462
cg20019489	-0.01435	-0.36858	0.71271	0.914629	chr20	57414351
cg09505516	-0.01124	-0.36668	0.714131	0.91515	chr2	25110296
cg06614951	0.010029	0.365857	0.714742	0.915359	chr19	14229385
cg08134671	0.016142	0.3655	0.715008	0.915453	chr19	2542837
cg24607686	-0.01337	-0.36546	0.715041	0.915453	chr2	191827930
cg02525785	0.010551	0.364995	0.715385	0.915557	chr17	7117684
cg14414124	-0.00951	-0.36306	0.71683	0.916027	chr2	68465373
cg26952925	-0.02386	-0.36245	0.717287	0.916188	chr16	4166391
cg23143233	-0.01111	-0.36111	0.718288	0.916614	chr20	57465864
cg03132806	0.010066	0.360755	0.718549	0.916728	chr5	178414179
cg00070899	-0.02174	-0.36054	0.71871	0.916772	chr6	34024479
cg04507426	-0.02461	-0.3597	0.719338	0.916935	chr16	56229180
cg10599507	-0.00819	-0.35919	0.719718	0.917043	chr6	33653337
cg18589960	-0.01181	-0.35858	0.72017	0.91718	chr19	51220392
cg25852492	-0.0092	-0.35831	0.720373	0.917258	chr15	42140150
cg11343713	0.005567	0.358252	0.720419	0.91726	chr7	45749313
cg08712808	-0.01488	-0.3582	0.720458	0.917274	chr3	7342929
cg21213853	0.021001	0.357957	0.72064	0.917333	chr3	51741473
cg18225409	0.009283	0.357265	0.721157	0.917491	chr11	70713375
cg06979118	-0.00862	-0.35725	0.721166	0.917491	chr11	70601971
cg16395366	-0.02422	-0.35299	0.724357	0.918797	chr1	53558245
cg09436713	0.008484	0.352824	0.72448	0.918858	chr12	2323135
cg02218260	-0.00971	-0.35261	0.72464	0.918912	chr3	51742878
cg11046772	-0.00866	-0.35207	0.725048	0.919095	chr12	2353479
cg15131024	0.014193	0.35191	0.725165	0.919134	chr11	70338408
cg25144574	0.007226	0.35182	0.725232	0.91915	chr5	36655019
cg18011401	0.013814	0.351667	0.725347	0.919152	chr19	13617366
cg04658038	-0.00761	-0.34947	0.726993	0.919727	chr17	64800166
cg26752663	-0.00602	-0.34933	0.727101	0.919766	chr2	25142016
cg06872721	-0.00803	-0.34917	0.727216	0.919801	chr19	19040258
cg14869721	0.008226	0.347444	0.728513	0.920192	chr2	25065924
cg15916804	-0.00839	-0.34614	0.729492	0.920553	chr10	75255500
cg12544392	-0.00508	-0.34582	0.729731	0.920657	chr19	42544587
cg24155399	-0.01111	-0.3432	0.7317	0.921484	chr1	235781578
cg25419928	-0.00521	-0.34309	0.731785	0.921515	chr6	33656793
cg03969219	0.010318	0.342592	0.732157	0.921664	chr19	2611456
cg15705536	-0.01282	-0.34208	0.732544	0.92178	chr5	7825292
cg13856573	-0.00711	-0.33976	0.734287	0.922532	chr11	62475078
cg21156276	0.012981	0.338934	0.734908	0.922811	chr9	4491917
cg02808075	0.014764	0.338732	0.735061	0.922854	chr3	4534881
cg00732970	0.006622	0.338163	0.735488	0.923034	chr20	57414162
cg16143105	-0.0169	-0.3377	0.735839	0.923083	chr5	78689278

cg19244300	0.0074	0.337466	0.736013	0.92311	chr1	110113304
cg14158769	0.020698	0.336664	0.736617	0.923292	chr7	126698156
cg03263685	-0.01007	-0.33558	0.737433	0.923607	chr2	68480160
cg10024799	-0.00802	-0.33536	0.737602	0.923661	chr12	2641381
cg07067241	-0.01163	-0.33532	0.73763	0.923662	chr3	142442711
cg16200584	-0.00863	-0.33468	0.738108	0.923805	chr11	62473981
cg00091960	0.0086	0.333921	0.738684	0.924008	chr7	126829514
cg23956071	-0.0166	-0.33356	0.738953	0.924035	chr17	4710044
cg02415992	0.009988	0.33262	0.739665	0.924276	chr16	10102278
cg25322847	-0.00799	-0.33256	0.739711	0.924307	chr7	45617892
cg20306837	-0.01164	-0.33198	0.74015	0.92444	chr11	105481988
cg10486865	0.01506	0.331217	0.740723	0.924522	chr17	64355892
cg16512895	0.009817	0.331025	0.740868	0.924606	chr19	13410117
cg03938110	-0.01023	-0.33052	0.741248	0.924706	chr21	31120485
cg23159970	-0.07675	-0.3287	0.74262	0.925184	chr12	2690385
cg03029664	-0.0074	-0.32637	0.744381	0.925679	chr17	72840306
cg01686093	0.006734	0.325782	0.744828	0.925854	chr11	70491582
cg22953407	0.006253	0.324551	0.745758	0.926266	chr5	178408081
cg24171047	-0.01156	-0.32251	0.747302	0.926756	chr17	64765921
cg12350325	-0.02117	-0.32203	0.747662	0.926934	chr12	2800909
cg07982896	0.013103	0.320995	0.748448	0.92714	chr19	13365938
cg24788034	-0.00932	-0.32097	0.748466	0.927157	chr19	2588241
cg03264550	-0.01579	-0.32065	0.748713	0.927243	chr20	57465448
cg17767099	0.01111	0.319628	0.749484	0.927605	chr19	1009048
cg17354190	-0.01153	-0.31885	0.75007	0.927789	chr17	72856064
cg17726655	0.007235	0.318389	0.750422	0.927962	chr19	42509860
cg05926269	-0.00721	-0.31726	0.751276	0.928282	chr20	57463906
cg26632831	0.008256	0.316513	0.751844	0.928567	chr11	70935863
cg11669839	-0.00409	-0.3162	0.752078	0.928656	chr20	57426322
cg03654504	0.009183	0.315051	0.752953	0.928988	chr1	37495105
cg06541349	0.006426	0.314708	0.753213	0.929106	chr16	4015096
cg21102121	0.013958	0.314629	0.753273	0.92913	chr19	54401295
cg06940168	0.01283	0.314241	0.753567	0.929245	chr17	64370665
cg11480627	0.018892	0.313484	0.754142	0.929434	chr11	70672876
cg10793758	-0.00696	-0.31223	0.755092	0.929724	chr22	51133417
cg05460776	-0.0072	-0.3121	0.755192	0.92976	chr16	4031231
cg13396607	0.00786	0.311936	0.755316	0.9298	chr6	102055059
cg16601231	0.015902	0.31118	0.75589	0.93	chr19	13367946
cg22620614	-0.00846	-0.31099	0.756038	0.93002	chr11	70794709
cg25423752	0.014459	0.310803	0.756176	0.930068	chr5	178422415
cg08091561	-0.01532	-0.31061	0.75632	0.930083	chr20	57426425
cg20459126	0.008227	0.309058	0.757503	0.930486	chr3	142443247
cg22986870	0.007312	0.307776	0.758477	0.930806	chr7	126765549
cg03503758	-0.00818	-0.30708	0.759007	0.930998	chr19	2614104
cg17959824	-0.00855	-0.30564	0.760098	0.93139	chr11	70391706
cg19200285	0.008315	0.305626	0.760112	0.93139	chr12	2800755
cg06025216	0.006876	0.305114	0.760502	0.931481	chr16	4164891
cg04994975	0.013167	0.304315	0.761109	0.931658	chr1	1795945

cg01174786	0.006335	0.301656	0.763133	0.932212	chr16	4027541
cg16182691	0.006054	0.299341	0.764897	0.932859	chr7	79762956
cg20822365	0.016439	0.29893	0.76521	0.932957	chr15	83621694
cg21636577	-0.0052	-0.2978	0.766072	0.933269	chr17	7121881
cg14331853	0.009381	0.296865	0.766785	0.933504	chr9	140054850
cg10698424	-0.00803	-0.29629	0.767227	0.933631	chr9	114423570
cg04338055	-0.00906	-0.29613	0.767349	0.933675	chr19	1000955
cg09340615	0.006811	0.29491	0.768276	0.933955	chr16	4021030
cg13559773	0.009067	0.294601	0.768513	0.934054	chr19	48562267
cg05065846	0.006326	0.293097	0.769661	0.934467	chr7	126885001
cg13401531	-0.00812	-0.29206	0.770456	0.934642	chr11	70333281
cg02557189	-0.00744	-0.29205	0.770463	0.934642	chr7	100273384
cg05082609	-0.01243	-0.29119	0.771115	0.934892	chr11	105692831
cg20779373	0.010787	0.289591	0.772339	0.935414	chr1	37428969
cg01749530	-0.00828	-0.28953	0.772387	0.935445	chr10	75255289
cg09965996	0.00721	0.287825	0.773689	0.935948	chr16	56390429
cg04473078	0.016015	0.287538	0.773909	0.936035	chr16	4165886
cg09997760	0.007691	0.287451	0.773975	0.936035	chr3	179169556
cg20882260	-0.00609	-0.28625	0.774895	0.936321	chr12	2374427
cg05577548	-0.01347	-0.28609	0.775018	0.936337	chr11	70666748
cg01140008	-0.00868	-0.28566	0.775345	0.936509	chr6	34002114
cg08240335	0.015271	0.284101	0.776539	0.936863	chr3	50273314
cg04835297	-0.0117	-0.2839	0.77669	0.936915	chr3	142443257
cg10887945	-0.00733	-0.28241	0.777833	0.937265	chr3	4805396
cg06930757	-0.00665	-0.28223	0.777969	0.937338	chr19	51216389
cg21028562	-0.00493	-0.28219	0.777999	0.937338	chr3	50287909
cg01866630	-0.0079	-0.28126	0.778716	0.937545	chr6	33601722
cg01192061	0.006144	0.281011	0.778905	0.93758	chr11	70368264
cg00318899	0.006565	0.280718	0.779129	0.937642	chr3	50284137
cg24287125	0.007587	0.279541	0.780032	0.937962	chr12	2692308
cg13139998	0.004885	0.279438	0.780111	0.938001	chr3	51740201
cg21370856	0.007705	0.279202	0.780292	0.938032	chr16	23848003
cg15212295	0.006545	0.277983	0.781226	0.938387	chr17	64710687
cg27552287	-0.01239	-0.27745	0.781633	0.93856	chr19	54385396
cg06952422	-0.00654	-0.27725	0.78179	0.938657	chr6	33647685
cg16848712	0.007761	0.277098	0.781905	0.938703	chr12	46767747
cg15174564	-0.02165	-0.27616	0.782623	0.938938	chr11	120856801
cg12150066	-0.00754	-0.27611	0.782666	0.938938	chr1	1823305
cg06085579	0.007333	0.275747	0.782941	0.939052	chr3	171509822
cg11207372	-0.00795	-0.27449	0.783905	0.939449	chr11	70385365
cg24620508	0.00856	0.269824	0.78749	0.940476	chr21	31310605
cg17641631	-0.00843	-0.26947	0.787759	0.940569	chr3	4535021
cg25228562	0.007095	0.269178	0.787987	0.940697	chr17	64718121
cg06693667	-0.00641	-0.26903	0.7881	0.940749	chr20	57426570
cg22060073	0.011098	0.268457	0.788541	0.940923	chr8	132052942
cg03716942	0.01116	0.268403	0.788583	0.940923	chr1	1821981
cg01419479	-0.01199	-0.26831	0.788651	0.940923	chr1	182360822
cg15828915	-0.01143	-0.26726	0.789461	0.941226	chr12	26801163

cg24203465	-0.00484	-0.26573	0.790635	0.941585	chr20	57425986
cg00683984	0.01135	0.265048	0.791164	0.941743	chr7	45615337
cg04017672	-0.0067	-0.26478	0.791372	0.941838	chr1	182359056
cg01364969	-0.00565	-0.26424	0.791784	0.941988	chr16	56389029
cg16909293	0.007276	0.263943	0.792014	0.942041	chr1	68171537
cg09437522	0.008321	0.263918	0.792033	0.94205	chr20	57431202
cg26201811	-0.01539	-0.26192	0.79357	0.94266	chr22	51111714
cg22134372	0.008158	0.261785	0.793676	0.942698	chr15	52455211
cg12372477	-0.00701	-0.26149	0.793901	0.942718	chr20	57465915
cg26674800	0.007509	0.261433	0.793947	0.942731	chr17	7108653
cg09403559	-0.00577	-0.26135	0.794007	0.942765	chr16	56334857
cg07906046	-0.00532	-0.26022	0.794878	0.943024	chr16	4131584
cg10639428	-0.00465	-0.26011	0.79497	0.943039	chr2	25138879
cg27083019	0.010341	0.258864	0.795927	0.943412	chr19	49945958
cg11444428	0.007778	0.258609	0.796123	0.943445	chr16	850802
cg25335435	0.014626	0.25776	0.796777	0.943594	chr11	22399705
cg16218964	-0.01365	-0.25772	0.796805	0.943601	chr11	62473680
cg22147917	-0.00634	-0.25765	0.796863	0.943629	chr6	33656031
cg13647052	-0.00625	-0.25589	0.798221	0.94414	chr12	2800382
cg15812599	-0.00637	-0.25543	0.798575	0.944288	chr11	70849065
cg10241462	0.006405	0.25474	0.799107	0.944373	chr2	191746790
cg26601922	-0.00679	-0.25457	0.799239	0.944412	chr3	50243174
cg00110790	0.006022	0.252723	0.800664	0.944892	chr6	33655814
cg07237830	0.005333	0.251742	0.801422	0.945217	chr11	62474725
cg07080031	-0.01839	-0.25133	0.801742	0.945332	chr11	70675170
cg02371119	0.007135	0.251026	0.801974	0.945458	chr3	171527346
cg23766996	0.009607	0.250708	0.80222	0.945493	chr7	86272023
cg14111697	0.0045	0.249903	0.802842	0.945731	chr9	80462928
cg04533189	-0.00698	-0.24982	0.802904	0.945736	chr17	64298763
cg02274788	0.006421	0.249124	0.803444	0.945897	chr1	68232457
cg13591723	-0.00611	-0.24879	0.803704	0.94597	chr12	46765135
cg06736148	-0.0054	-0.24772	0.804532	0.94618	chr15	52416833
cg10139742	0.007387	0.247672	0.804566	0.946189	chr16	56352151
cg15056189	-0.0057	-0.24684	0.805209	0.946381	chr12	49176428
cg23484981	0.023571	0.243264	0.807975	0.947235	chr20	57426626
cg21350778	0.0081	0.241279	0.809512	0.947892	chr17	64297603
cg09554596	0.007135	0.240921	0.809789	0.947996	chr19	1004620
cg17818798	-0.00631	-0.2408	0.809882	0.948044	chr19	14228473
cg13631572	0.007778	0.240776	0.809901	0.948044	chr14	24803903
cg00788521	-0.00693	-0.24022	0.810332	0.948195	chr12	2229269
cg03626208	0.004861	0.239985	0.810514	0.948258	chr12	2443169
cg25904372	-0.00563	-0.23948	0.810908	0.948391	chr11	70628970
cg05313261	-0.01914	-0.23915	0.81116	0.948496	chr16	30134350
cg15219163	0.007316	0.238682	0.811523	0.948623	chr11	70842128
cg25963822	0.006668	0.23726	0.812625	0.948975	chr7	100270831
cg04107939	-0.00466	-0.23698	0.812842	0.949091	chr3	171520494
cg20008140	-0.00827	-0.23692	0.812888	0.949097	chr20	57463455
cg12650926	-0.00729	-0.23571	0.813826	0.949359	chr6	33589118

cg13650938	-0.00824	-0.23565	0.813871	0.949368	chr19	2579075
cg22934516	0.009574	0.235523	0.813971	0.949429	chr11	35413951
cg26863600	-0.00517	-0.2354	0.814066	0.949462	chr19	2616921
cg21340148	0.006832	0.235224	0.814203	0.949495	chr19	2702986
cg13757263	-0.00637	-0.23514	0.814266	0.949518	chr3	53807593
cg26914334	-0.00523	-0.23479	0.814537	0.949613	chr11	120561236
cg10276272	-0.00655	-0.23316	0.815804	0.949918	chr16	10271822
cg22900607	-0.01178	-0.23299	0.815935	0.949954	chr19	2546938
cg17702518	0.005623	0.232547	0.816279	0.95009	chr7	100271260
cg04865531	0.005897	0.232413	0.816383	0.950118	chr22	51159147
cg01793368	0.004755	0.231369	0.817193	0.950474	chr11	64022905
cg07687951	0.008185	0.230425	0.817926	0.950723	chr11	70666560
ch.5.240336F	0.017723	0.227467	0.820223	0.95127	chr5	7757969
cg06181697	-0.0057	-0.22703	0.820564	0.951344	chr3	50294603
cg07121488	-0.00449	-0.22696	0.82062	0.951347	chr15	40581105
cg15993383	0.012425	0.226159	0.82124	0.951488	chr3	123167507
cg19696388	0.005087	0.225484	0.821764	0.951641	chr19	19042720
cg03078593	-0.00615	-0.22531	0.821903	0.951693	chr12	26789311
cg04903916	-0.00503	-0.22509	0.822066	0.951709	chr6	33638413
cg21809160	-0.0055	-0.22394	0.822964	0.95199	chr20	57428309
cg21151432	0.006018	0.223803	0.82307	0.952	chr2	25142229
cg01118752	-0.01047	-0.22368	0.823167	0.95202	chr4	102264326
cg08834938	-0.00405	-0.22276	0.823885	0.952228	chr16	4136053
cg26393354	0.00546	0.222525	0.824064	0.952305	chr11	70713937
cg21997766	0.007173	0.222448	0.824124	0.952309	chr17	72846113
cg08897759	-0.0089	-0.22194	0.824521	0.952431	chr5	178415944
cg11025960	-0.00548	-0.22177	0.824649	0.952463	chr3	51749188
cg14975881	0.005574	0.221583	0.824796	0.952531	chr19	54389945
cg26985666	0.007757	0.220747	0.825447	0.952696	chr11	35441088
cg27074174	0.006677	0.220657	0.825517	0.952711	chr6	101847318
cg14694901	0.009315	0.220559	0.825593	0.952755	chr10	75255186
cg03047070	0.004328	0.220475	0.825658	0.952767	chr12	2787827
cg00150025	0.006838	0.220216	0.82586	0.95284	chr15	42448079
cg25592107	0.008259	0.219763	0.826212	0.952957	chr19	19042864
cg17500055	-0.00917	-0.2192	0.826653	0.953119	chr1	235805560
cg01962496	-0.00754	-0.21861	0.827111	0.953211	chr5	78809740
cg12079381	0.008827	0.215807	0.829292	0.953862	chr21	31310920
cg10820904	0.005966	0.215259	0.829718	0.953968	chr17	64412745
cg24058407	-0.00529	-0.21467	0.830175	0.95411	chr20	57428282
cg16400825	0.0062	0.21387	0.830801	0.954328	chr6	33589418
cg04576607	0.005305	0.213394	0.831172	0.954444	chr1	235787279
cg19228334	-0.01134	-0.21292	0.831545	0.95456	chr6	101851283
cg00672228	0.005397	0.212493	0.831874	0.954633	chr17	7123130
cg12536809	0.005017	0.211127	0.832938	0.954928	chr17	72852514
cg18318307	-0.00486	-0.2111	0.832963	0.954928	chr11	70368307
cg17329110	0.011497	0.210763	0.833222	0.954991	chr11	70708812
cg27066052	0.00478	0.209551	0.834167	0.955364	chr16	4015761
cg00701890	-0.0036	-0.20678	0.836331	0.956047	chr16	4102293

cg07386190	0.005838	0.205122	0.837623	0.956413	chr1	182361453
cg05029189	0.007168	0.203504	0.838886	0.956839	chr3	123168386
cg02991464	-0.00504	-0.20258	0.83961	0.956979	chr12	2788732
cg13000134	-0.0056	-0.20155	0.840413	0.957231	chr19	48896922
cg05120716	-0.00383	-0.20143	0.840507	0.957273	chr16	23881993
cg03182218	-0.00637	-0.20115	0.840728	0.957344	chr17	7100221
cg16850687	0.007264	0.201109	0.840756	0.957352	chr18	3594398
cg06864895	0.005263	0.200321	0.841372	0.957501	chr12	46767683
cg10116505	0.005759	0.19922	0.842232	0.957733	chr16	10274064
cg09980522	-0.00717	-0.19911	0.842319	0.957733	chr11	105481802
cg09134640	-0.00418	-0.19871	0.842627	0.957856	chr1	37337877
cg02306526	0.004456	0.198427	0.842852	0.957931	chr12	2202821
cg01596520	-0.00418	-0.19583	0.844883	0.958619	chr19	14225029
cg17789138	0.005883	0.195131	0.845429	0.958697	chr19	49936880
cg09001143	-0.00575	-0.19364	0.846594	0.958932	chr16	9857475
cg16600501	-0.00808	-0.19336	0.846813	0.958987	chr19	15083842
cg20259256	-0.00697	-0.19267	0.847355	0.959136	chr3	142443266
cg06898306	-0.00766	-0.19227	0.847669	0.959213	chr6	33996673
cg11155924	-0.00725	-0.19174	0.84808	0.959372	chr11	70449258
cg06111374	0.005752	0.191727	0.848092	0.959372	chr12	14109584
cg23202253	0.00635	0.191124	0.848564	0.959506	chr12	26902211
cg00951869	-0.00309	-0.19056	0.849008	0.959643	chr14	24805349
cg04122657	0.007671	0.190017	0.84943	0.959741	chr16	4014295
cg17961101	-0.00776	-0.18891	0.8503	0.959993	chr22	22222050
cg25399541	0.006713	0.188192	0.85086	0.960193	chr7	45622395
cg18160880	-0.00432	-0.18787	0.85111	0.960278	chr20	57463903
cg09726240	-0.00843	-0.18746	0.851431	0.960368	chr11	70672878
cg27184649	0.004347	0.185906	0.85265	0.960638	chr11	70935963
cg20018057	0.00341	0.183774	0.854321	0.961028	chr20	57465139
cg27012424	-0.00637	-0.183	0.854924	0.961249	chr18	3773224
cg06716686	-0.00454	-0.18171	0.855938	0.961557	chr3	4535154
cg17260383	-0.00368	-0.18068	0.856746	0.961712	chr8	22298246
cg02799411	-0.00381	-0.18058	0.856829	0.961715	chr3	4794061
cg19120580	0.003429	0.179998	0.857282	0.961846	chr12	49173414
cg05944877	-0.00464	-0.17935	0.857788	0.962053	chr16	24197863
cg11264539	-0.0033	-0.17908	0.858001	0.962082	chr11	62474940
cg23542572	0.003531	0.178972	0.858086	0.962103	chr17	64780054
cg08104845	-0.00611	-0.17735	0.859358	0.962453	chr1	182356337
cg14907788	-0.00404	-0.17434	0.861724	0.963164	chr19	2555976
cg15008401	0.004862	0.173982	0.862003	0.963243	chr4	102267974
cg04127894	-0.0041	-0.17305	0.862732	0.963486	chr15	40594732
cg15154232	0.007377	0.172162	0.863432	0.963751	chr19	48615306
cg24204556	0.008418	0.171391	0.864038	0.96396	chr22	22222030
cg25960567	-0.00708	-0.1702	0.864975	0.964306	chr12	26985181
cg14420982	0.004307	0.170049	0.865092	0.964349	chr9	80647609
cg17540499	0.007597	0.169242	0.865726	0.964516	chr7	126698451
cg12076692	0.00634	0.168956	0.865951	0.964565	chr1	1718852
cg09655520	0.002994	0.168908	0.865988	0.964565	chr17	64786064

cg11112257	-0.00525	-0.16869	0.866159	0.964573	chr11	88781135
cg26213368	-0.00348	-0.16867	0.866179	0.964573	chr11	62474978
cg11953334	0.004256	0.168508	0.866302	0.964582	chr19	48897863
cg10623198	0.003065	0.168326	0.866446	0.964616	chr12	6949114
cg02953559	-0.00366	-0.16689	0.867571	0.965032	chr3	123164964
cg09103960	0.004286	0.166672	0.867746	0.965054	chr16	56225504
cg15692593	-0.00599	-0.16663	0.867782	0.965066	chr6	101993140
cg11435239	0.005272	0.166235	0.86809	0.965169	chr19	51220297
cg19742341	-0.00451	-0.16497	0.869086	0.965471	chr11	70385301
cg12996903	-0.00395	-0.16404	0.869818	0.965695	chr3	50275575
cg07287255	0.00531	0.163574	0.870182	0.965758	chr16	56374688
cg22459924	-0.00295	-0.16096	0.872241	0.966415	chr19	2607850
cg00997853	-0.00447	-0.1608	0.872363	0.966443	chr12	26844579
cg02261541	0.004594	0.160191	0.872844	0.966608	chr16	4050315
cg02937055	0.006315	0.159565	0.873336	0.966783	chr3	171489625
cg05297437	-0.00475	-0.15943	0.873442	0.966794	chr20	57471672
cg20103018	0.003417	0.159226	0.873603	0.966869	chr6	33996522
cg03173525	0.004189	0.157205	0.875194	0.96729	chr7	100273221
cg01025883	-0.00442	-0.15677	0.875536	0.967391	chr16	23867088
cg07036561	0.003152	0.155599	0.876458	0.967634	chr15	42118869
cg02441543	0.007003	0.154961	0.876961	0.967802	chr22	51157530
cg05119467	0.003612	0.154663	0.877196	0.967856	chr1	1765440
cg26322763	0.003539	0.153983	0.877732	0.968032	chr19	48914444
cg07947033	-0.00576	-0.15374	0.877924	0.968098	chr20	57426545
cg10622236	-0.00337	-0.15293	0.878562	0.968243	chr15	42120082
cg13804196	-0.00437	-0.15198	0.879312	0.968434	chr9	71628906
cg27112585	0.004039	0.15136	0.879799	0.96856	chr11	70378117
cg02374107	-0.00302	-0.15097	0.880107	0.968636	chr16	4163907
cg10577016	0.00655	0.149958	0.880904	0.968876	chr9	114423680
cg00267746	-0.00297	-0.14895	0.881702	0.969046	chr20	57463984
cg04084618	-0.00587	-0.14891	0.881731	0.969055	chr5	36607065
cg15844381	0.007544	0.148584	0.881987	0.969099	chr19	14228577
cg20091384	-0.00386	-0.14847	0.882079	0.969134	chr19	2700927
cg02738298	0.002546	0.148465	0.882081	0.969134	chr3	51749852
cg01638185	0.004889	0.14618	0.883882	0.969697	chr17	64530027
cg18851100	0.003683	0.146026	0.884004	0.969771	chr22	51158550
cg08413366	0.004319	0.145756	0.884217	0.969844	chr10	75255930
cg25766748	-0.00606	-0.1451	0.884737	0.969945	chr19	13614882
cg04132853	0.003079	0.143818	0.885745	0.970313	chr20	57414039
cg01332711	0.003609	0.14381	0.885752	0.970313	chr15	42120681
cg07267600	0.003209	0.14364	0.885886	0.970357	chr12	2750053
cg09053902	-0.00711	-0.14348	0.886016	0.970397	chr16	4034298
cg21931938	0.003332	0.143451	0.886035	0.9704	chr15	40600493
cg07502066	0.008682	0.143321	0.886137	0.970443	chr1	110091086
cg05432017	-0.00346	-0.14285	0.886507	0.970554	chr15	42119684
cg08997444	0.002481	0.142	0.88718	0.970711	chr20	57464970
cg01010868	-0.0092	-0.14157	0.887521	0.970807	chr19	14228654
cg04696980	0.003339	0.139734	0.888969	0.971207	chr19	2586206

cg27109030	-0.00439	-0.1395	0.88915	0.971268	chr19	2702898
cg27642181	-0.00362	-0.13876	0.889741	0.971375	chr20	57413694
cg07218663	-0.00628	-0.13821	0.890169	0.971464	chr6	146350618
cg10943398	-0.00383	-0.13674	0.891333	0.971825	chr11	70319250
cg14099468	0.006344	0.135576	0.892252	0.972069	chr1	235814814
cg02748316	-0.00821	-0.13556	0.892267	0.972069	chr3	50273710
cg01895482	0.003157	0.135232	0.892523	0.972136	chr19	2556145
cg19853565	-0.00331	-0.13509	0.892638	0.972163	chr19	2540907
cg07268119	0.002949	0.134094	0.893422	0.972432	chr7	45717573
cg24902435	-0.00317	-0.1338	0.893653	0.972496	chr12	2790095
cg17841572	0.002201	0.133216	0.894117	0.972679	chr20	57426368
cg05087008	-0.00383	-0.1332	0.894129	0.972679	chr11	105483680
cg23325230	-0.00444	-0.13219	0.894929	0.972778	chr7	100272578
cg12216470	-0.00605	-0.13202	0.895063	0.972782	chr19	51200481
cg27318000	0.006139	0.131935	0.895129	0.972782	chr18	3845667
cg00808175	0.002752	0.13193	0.895132	0.972782	chr12	6949119
cg01450274	-0.00626	-0.13149	0.895483	0.972882	chr3	171396325
cg11803392	-0.00349	-0.13124	0.895676	0.972908	chr17	64712432
cg20789595	-0.00302	-0.13032	0.896407	0.973038	chr3	123063477
cg05564552	0.00312	0.129983	0.896671	0.973138	chr15	42120091
cg12204773	0.004388	0.129665	0.896923	0.973206	chr17	7123253
cg10161743	-0.00389	-0.12943	0.897105	0.973224	chr19	48917816
cg03973705	-0.00253	-0.12758	0.898567	0.973627	chr16	24174850
cg11227541	0.003876	0.125809	0.899972	0.974041	chr1	84972317
cg02380983	0.003335	0.125647	0.900099	0.974104	chr19	48568071
cg18554395	0.003499	0.125572	0.900159	0.974112	chr19	2555791
cg06088782	-0.00329	-0.12554	0.900183	0.974128	chr11	70563839
cg08364956	-0.00402	-0.12521	0.900442	0.974151	chr5	178407122
cg23054925	0.004485	0.124918	0.900677	0.974211	chr1	84972704
cg27644733	-0.00371	-0.12472	0.900829	0.97427	chr16	9857216
cg17665552	-0.00533	-0.12291	0.902262	0.974723	chr11	70455599
cg19128261	0.002395	0.119835	0.904698	0.975462	chr3	53839251
cg20950146	0.003035	0.119681	0.90482	0.975483	chr5	7827524
cg22198397	0.002461	0.119584	0.904896	0.975489	chr19	15067457
cg26613742	-0.00144	-0.11861	0.905671	0.975658	chr19	14225000
cg13680388	-0.00296	-0.11824	0.905963	0.975672	chr20	57471844
cg17986880	-0.00329	-0.11784	0.906279	0.97572	chr7	79848290
cg00050312	0.002994	0.11734	0.906673	0.97582	chr17	64299065
cg20326410	0.002437	0.117098	0.906865	0.975822	chr1	53600821
cg22125912	0.004305	0.116354	0.907453	0.975956	chr3	171428263
cg11820929	-0.00424	-0.11514	0.908419	0.976234	chr4	102258631
cg16418734	0.00281	0.11512	0.908431	0.976244	chr1	1720537
cg04675204	-0.00333	-0.11386	0.909426	0.97659	chr16	10179771
cg00369202	-0.00278	-0.11204	0.910869	0.97703	chr6	33989844
cg11414276	-0.00224	-0.11199	0.910909	0.97703	chr12	2166831
cg22749173	0.002492	0.11121	0.911528	0.977083	chr19	2614039
cg19747632	0.002316	0.110676	0.911951	0.97716	chr15	52472703
cg24719827	-0.00263	-0.10834	0.913805	0.977534	chr1	110091174

cg16560679	0.002273	0.107423	0.914529	0.977752	chr7	100276684
cg16701848	0.00251	0.106828	0.915	0.977904	chr19	2588479
cg24591824	0.002863	0.105794	0.91582	0.978171	chr12	2762732
cg25702790	0.003217	0.105318	0.916197	0.978274	chr7	79765394
cg03242834	-0.00311	-0.10351	0.91763	0.978721	chr19	13317326
cg18801906	-0.0032	-0.10188	0.918926	0.979076	chr11	70584165
cg25229306	0.001766	0.101582	0.91916	0.9791	chr20	57426374
cg04677683	0.003552	0.10043	0.920073	0.979304	chr20	57426743
cg08901242	0.003938	0.099978	0.920432	0.979443	chr19	15083667
cg22598669	-0.00413	-0.099	0.921207	0.979628	chr19	51186978
cg00866976	0.005744	0.098869	0.921312	0.979668	chr16	56224782
cg12046677	0.00222	0.098689	0.921454	0.979711	chr12	2360663
cg27644513	0.003196	0.098127	0.9219	0.979785	chr15	42281679
cg04576491	-0.00262	-0.09811	0.921914	0.979785	chr19	1005427
cg11856810	0.004078	0.098104	0.921918	0.979785	chr2	155554961
cg07366462	0.001861	0.097267	0.922582	0.980035	chr3	123162899
cg13824515	0.004	0.095531	0.92396	0.980413	chr9	140047122
cg11692123	0.002217	0.094976	0.9244	0.980516	chr11	70935949
cg07986199	-0.00251	-0.09213	0.926662	0.981099	chr12	2743038
cg01944370	0.001861	0.09029	0.92812	0.981519	chr3	50286969
cg23753795	-0.00467	-0.09021	0.928181	0.981541	chr6	146531540
cg14061491	-0.00555	-0.08944	0.928793	0.981699	chr9	80433462
cg19325477	0.003164	0.087637	0.930227	0.982112	chr17	64688496
cg23808301	0.002325	0.087477	0.930353	0.982158	chr17	4710015
cg02902102	0.002946	0.085456	0.931959	0.982629	chr19	48902290
cg01865825	-0.0024	-0.08429	0.932885	0.982865	chr16	850240
cg05255330	-0.00182	-0.08312	0.933811	0.983158	chr18	3498963
cg01520586	0.002068	0.08218	0.934561	0.983263	chr3	50280404
cg12568707	-0.00228	-0.08154	0.935072	0.983422	chr19	19042904
cg17222829	-0.00195	-0.07911	0.937	0.983892	chr11	70433293
cg17921248	0.002461	0.078469	0.93751	0.984034	chr17	64298993
cg10738479	-0.00199	-0.0784	0.937565	0.98404	chr19	1000105
cg11830694	-0.0023	-0.07791	0.937957	0.984136	chr12	2289797
cg16377872	0.001938	0.077066	0.938625	0.984336	chr19	15084823
cg00269140	-0.00393	-0.07649	0.939083	0.984447	chr7	86389542
cg18827756	0.00148	0.075783	0.939645	0.984544	chr15	42130735
cg09950871	-0.00172	-0.07515	0.94015	0.984663	chr16	4029235
cg11317158	-0.00254	-0.07435	0.940782	0.984797	chr19	49938204
cg13592780	0.003104	0.073777	0.941239	0.984954	chr3	123010034
cg22827707	-0.00179	-0.07325	0.94166	0.985032	chr6	34100899
cg00041368	-0.00166	-0.07202	0.942637	0.985348	chr18	3879131
cg06996175	0.001691	0.071984	0.942665	0.98535	chr19	2546877
cg07636145	-0.00287	-0.07105	0.943407	0.985587	chr16	56228188
cg22960869	-0.00283	-0.07084	0.943572	0.98565	chr17	47287521
cg03153115	-0.00153	-0.06966	0.944514	0.985922	chr19	2604559
cg12863967	0.002151	0.069581	0.944576	0.985937	chr7	93534920
cg01035815	-0.00242	-0.0679	0.945914	0.986313	chr6	33600828
cg21650436	-0.00113	-0.06765	0.946109	0.986358	chr12	49162444

cg06293172	-0.0021	-0.06644	0.947073	0.986655	chr2	25045211
cg02774856	-0.00167	-0.06628	0.947204	0.986698	chr19	19052293
cg23055496	-0.00222	-0.06558	0.947755	0.986848	chr3	6906371
cg25869295	-0.00159	-0.06461	0.948529	0.987062	chr11	70433086
cg03425609	-0.00129	-0.06449	0.948625	0.98711	chr12	6952374
cg25693099	0.001412	0.063602	0.949332	0.987255	chr18	3879303
cg25804443	-0.00257	-0.06331	0.94956	0.987337	chr18	3875823
cg14583606	-0.00339	-0.0624	0.950286	0.987566	chr9	4490315
cg19008133	0.001362	0.062383	0.950301	0.987566	chr3	123124015
cg19367232	-0.00258	-0.06235	0.950325	0.987566	chr2	68478649
cg10538151	0.002673	0.060911	0.951473	0.987877	chr9	140033364
cg21146273	-0.00213	-0.0603	0.951956	0.98802	chr5	152869193
cg19892433	-0.00178	-0.05952	0.952582	0.988179	chr11	70331861
cg09428623	0.001518	0.058522	0.953374	0.988471	chr2	68480128
cg23465427	0.001122	0.058092	0.953716	0.988511	chr17	7111429
cg02203881	0.001138	0.057546	0.95415	0.988624	chr15	42386909
cg00498360	0.001286	0.057259	0.954379	0.988661	chr17	64504304
cg01187464	-0.0015	-0.05666	0.954852	0.98877	chr16	50351302
cg14351882	-0.00263	-0.05574	0.95559	0.989014	chr9	140061878
cg07028661	0.001885	0.054178	0.956831	0.989251	chr21	31312905
cg01728682	-0.00227	-0.05411	0.956885	0.989278	chr2	25057480
cg24889366	-0.00186	-0.05406	0.956922	0.989289	chr16	850646
cg13327846	-0.00146	-0.05335	0.957488	0.989444	chr15	52472389
cg03014008	-0.00112	-0.05252	0.958153	0.989661	chr20	57463767
cg12933359	-0.00122	-0.05212	0.958466	0.989691	chr7	86416314
cg16142824	0.001636	0.052034	0.958538	0.989697	chr5	178413721
cg02484455	0.001139	0.051634	0.958856	0.989792	chr11	70559534
cg03616148	-0.00231	-0.05096	0.959395	0.989889	chr16	9911529
cg04926767	-0.0011	-0.05049	0.959768	0.989969	chr11	62476194
cg06868991	0.001279	0.050376	0.959858	0.989985	chr11	70774323
cg22804770	0.001001	0.049368	0.96066	0.990103	chr12	2786316
cg27552955	-0.00249	-0.04937	0.960661	0.990103	chr3	142442915
cg10797197	-0.00167	-0.04887	0.961055	0.990241	chr20	57444000
cg13624528	-0.00251	-0.04846	0.961384	0.990285	chr6	101846409
cg14298577	-0.00119	-0.0483	0.961508	0.990314	chr7	100272703
cg02598335	-0.00114	-0.04804	0.96172	0.990393	chr7	86337918
cg16106068	0.000931	0.047929	0.961806	0.990437	chr14	24792081
cg04086239	0.001227	0.046153	0.96322	0.9908	chr16	24067174
cg09320113	0.001186	0.045295	0.963904	0.990993	chr1	186799481
cg20326682	-0.00085	-0.04471	0.964366	0.991024	chr17	64345488
cg19217955	0.001022	0.044646	0.96442	0.991041	chr17	7123994
cg20170028	-0.00209	-0.0442	0.964775	0.991164	chr11	70917283
cg11706780	0.00092	0.043527	0.965312	0.991281	chr15	42289766
cg14295482	-0.00101	-0.04257	0.966071	0.991503	chr19	2555717
cg20801637	-0.00118	-0.04243	0.966185	0.991537	chr1	1795408
cg00161247	-0.00105	-0.04206	0.966484	0.991643	chr9	140060986
cg02309655	-0.00097	-0.04097	0.967345	0.991819	chr19	2588629
cg13065504	0.001019	0.040085	0.968053	0.992069	chr15	42448234

cg03478199	0.0015	0.039877	0.968219	0.99209	chr6	146348913
cg26635576	0.001176	0.039237	0.968729	0.992271	chr11	35275997
cg21330323	0.000378	0.039223	0.96874	0.992276	chr20	57414596
cg07587653	0.000833	0.03521	0.971937	0.992987	chr11	70338480
cg06932616	-0.00151	-0.03382	0.973041	0.993247	chr19	48908335
cg24226238	0.000715	0.032977	0.973716	0.993423	chr11	120530774
cg08535918	-0.00063	-0.03102	0.975272	0.993798	chr16	56256748
cg03503785	-0.00083	-0.03087	0.975393	0.99383	chr16	23962572
cg24824840	0.000832	0.029151	0.976765	0.994167	chr19	51219975
cg07774177	-0.00091	-0.0291	0.976804	0.994167	chr15	42387287
cg27371466	0.000605	0.029061	0.976836	0.994167	chr6	34003640
cg10371483	-0.00083	-0.02902	0.976871	0.994167	chr16	4152045
cg09143713	0.000611	0.027427	0.978138	0.994489	chr20	9141615
cg27433516	-0.00067	-0.02696	0.978508	0.994569	chr1	37266621
cg25193885	0.000618	0.026349	0.978997	0.994702	chr11	70328867
cg15218096	0.000977	0.026282	0.979051	0.994704	chr11	70858342
cg12116020	0.000911	0.024811	0.980223	0.995054	chr19	14228622
cg22990158	0.001663	0.023951	0.980909	0.995202	chr14	24802150
cg18151275	-0.00071	-0.02373	0.981083	0.995262	chr12	56873616
cg04803128	0.000583	0.023221	0.98149	0.995401	chr7	100273280
cg24611631	0.000665	0.022995	0.981671	0.99544	chr9	4490288
cg22639787	0.00039	0.020992	0.983267	0.995776	chr20	57464973
cg20495738	0.000608	0.020952	0.983298	0.995776	chr12	2338399
cg23374892	0.001307	0.020181	0.983913	0.995906	chr19	51165845
cg22076160	0.000925	0.019924	0.984118	0.995971	chr1	235805690
cg05509359	0.000708	0.019374	0.984556	0.996083	chr11	70432884
cg08848088	0.000432	0.018351	0.985372	0.996353	chr1	235714526
cg27173374	0.000647	0.018043	0.985617	0.996405	chr14	52413159
cg27179693	-0.00039	-0.0171	0.986368	0.996547	chr11	120530818
cg26564874	0.00039	0.016212	0.987076	0.996724	chr5	178416134
cg15878555	0.000474	0.015908	0.987319	0.996789	chr11	62473962
cg06525750	-0.00044	-0.01575	0.987444	0.99681	chr2	25141152
cg02993882	0.000255	0.01428	0.988616	0.997167	chr16	4043463
cg18356448	-0.00061	-0.01372	0.989063	0.997314	chr18	3881547
cg15160746	-0.00037	-0.01242	0.9901	0.99758	chr12	2743239
cg12201698	0.000263	0.011799	0.990594	0.997718	chr11	70634975
cg06192619	-0.00027	-0.01017	0.991891	0.998037	chr19	15083616
cg15985106	-0.00018	-0.00924	0.992636	0.998306	chr12	6954791
cg00586732	-0.00024	-0.0088	0.992982	0.998391	chr3	7755472
cg21518089	-0.00023	-0.00841	0.993295	0.998467	chr11	22362708
cg24617313	-0.00088	-0.00814	0.993513	0.998467	chr20	57427146
cg00646241	-0.00023	-0.00659	0.994749	0.998879	chr11	70563878
cg04884798	0.000123	0.00651	0.994811	0.998895	chr14	24791720
cg08982381	-0.00017	-0.006	0.995213	0.998977	chr9	80647290
cg09927287	-0.0003	-0.00541	0.995689	0.999058	chr15	42447989
cg12667048	0.000144	0.004832	0.996148	0.999184	chr11	70644526
cg01255513	-0.00013	-0.00394	0.996861	0.999384	chr19	13365923
cg18229071	-5.51E-05	-0.00346	0.997242	0.999413	chr19	2695245

cg12228229	-6.57E-05	-0.00301	0.997603	0.999477	chr17	7122261
cg03104569	-0.0001	-0.00281	0.997762	0.999509	chr19	13615864
cg08486432	-2.96E-05	-0.00133	0.998939	0.999767	chr6	33598003
cg09890339	-1.17E-05	-0.00051	0.999596	0.99993	chr12	2734150
cg00652727	1.59E-05	0.000414	0.99967	0.999936	chr1	235812198
