# Supplementary material for: Differential DNA Methylation of the Serotonin Receptor Signaling and Glutamatergic Synapse Pathways in Adult Twins Born Preterm
Source: Genes (Basel). 2026 Jun 10;17(6):683. doi: 10.3390/genes17060683 (PMC13299586; doi:10.3390/genes17060683)
Supplement: Supplementary file 1 [file genes-17-00683-s001.zip › Supplementary Table S5_serotonin_young.pdf]

CpG	logFC	t	P.Value	adj.P.Val	chr	pos
cg27549720	-0.30541	-4.70285	4.00E-06	0.010435	chr1	19992167
cg06291867	-0.2168	-4.68165	4.40E-06	0.010435	chr10	92617162
cg23764129	0.12579	4.484463	1.06E-05	0.012612	chr11	113846017
cg18023598	-0.18239	-4.36258	1.80E-05	0.014678	chr1	19992504
cg07664198	-0.17199	-4.34151	1.97E-05	0.014764	chr7	136553882
cg01281175	0.273247	4.055637	6.45E-05	0.019718	chr5	175109114
cg00183186	-0.20185	-3.99047	8.39E-05	0.021504	chr6	78174065
cg00903099	-0.16937	-3.87693	0.000131	0.024643	chr7	154862441
cg11615755	-0.17432	-3.85661	0.000142	0.025292	chr5	63257867
cg19500607	0.156572	3.685574	0.000273	0.030934	chr5	148034319
cg24845274	-0.17114	-3.65031	0.000311	0.032464	chr7	136555697
cg22059812	-0.163	-3.6217	0.000346	0.033352	chr1	19992564
cg17805202	0.146632	3.608353	0.000364	0.033994	chr1	239937440
cg09386376	0.278456	3.510947	0.000519	0.038831	chr11	638939
cg12103152	-0.14945	-3.50006	0.000539	0.03933	chr6	78173200
cg07102705	-0.17436	-3.48158	0.000576	0.040148	chr5	148033896
cg05717871	0.295981	3.462266	0.000618	0.041044	chr11	638507
cg07833420	-0.14623	-3.41561	0.000729	0.043578	chr6	87647147
cg06531741	-0.14657	-3.40825	0.000748	0.044087	chr11	113775450
cg04427003	-0.13665	-3.4053	0.000756	0.044293	chr5	63257499
cg08186362	-0.4188	-3.3843	0.000813	0.045442	chr20	60794868
cg08323651	-0.16267	-3.36107	0.000882	0.04665	chr7	136553855
cg03909863	0.51416	3.335203	0.000965	0.048141	chr11	638404
cg24575234	-0.15411	-3.32295	0.001007	0.049054	chr7	136553884
cg00840960	-0.14617	-3.30084	0.001086	0.05038	chr5	148034030
cg16280141	-0.16453	-3.28378	0.001152	0.051462	chr5	63257753
cg17220584	0.145778	3.278779	0.001171	0.05173	chr5	148017424
cg24228819	-0.14847	-3.26365	0.001233	0.052671	chr7	136553868
cg21566860	0.099958	3.246355	0.001308	0.053875	chr3	11242797
cg01257383	0.070016	3.235636	0.001356	0.054577	chr11	46408399
cg09640960	-0.14857	-3.15994	0.001747	0.060235	chr20	60794676
cg09863441	-0.1399	-3.14006	0.001866	0.062128	chr6	87647129
cg14433983	0.237276	3.137382	0.001883	0.062387	chr11	636460
cg22471401	0.081464	3.110209	0.002058	0.064623	chr3	183824717
cg18019017	-0.09616	-3.06777	0.002363	0.068242	chr6	78173408
cg03138127	0.258814	3.067347	0.002367	0.06829	chr2	231989146
cg12825070	-0.23395	-3.05021	0.002501	0.069591	chr5	148033708
cg02266732	-0.14233	-3.04836	0.002516	0.069729	chr5	63257710
cg25632105	-0.15869	-3.04739	0.002524	0.069805	chr7	136553728
cg13077519	-0.13997	-3.04201	0.002568	0.070254	chr6	78174007
cg05919907	0.091866	2.982372	0.003107	0.075584	chr5	175105350
cg11298960	-0.14858	-2.97289	0.003201	0.076517	chr7	154862548
cg03986968	-0.10077	-2.95152	0.003424	0.07857	chr15	34330894
cg00120810	0.093231	2.933123	0.003627	0.080587	chr3	11195317
cg12198176	0.083021	2.930078	0.003662	0.080986	chr22	23415316
cg27615388	-0.19064	-2.91408	0.003849	0.082754	chr5	63257092
cg10979181	0.151635	2.864359	0.004488	0.087888	chr7	136586606

cg08614481	-0.12593	-2.85974	0.004552	0.088463	chr6	78173250
cg00454577	-0.10686	-2.84057	0.004827	0.090623	chr6	87646972
cg23247337	0.118523	2.833874	0.004926	0.091361	chr3	11178285
cg07212818	0.185816	2.831657	0.004959	0.091537	chr11	638076
cg08208133	-0.09928	-2.83146	0.004962	0.091573	chr11	113848161
cg02389195	0.079827	2.83022	0.004981	0.091687	chr5	175088015
cg06825142	-0.12864	-2.82663	0.005036	0.092079	chr11	637170
cg05756489	-0.194	-2.81963	0.005144	0.092876	chr10	92616870
cg05650628	0.083565	2.801977	0.005426	0.094913	chr11	62677384
cg14632899	0.07029	2.775903	0.005868	0.097927	chr11	62678618
cg21127286	-0.10593	-2.72272	0.006873	0.104211	chr7	136555154
cg11702866	0.076251	2.72143	0.006899	0.104362	chr3	11267098
cg19503977	-0.09584	-2.70774	0.007182	0.106226	chr6	78173287
cg04842426	-0.25924	-2.70055	0.007335	0.107218	chr7	136555777
cg13102079	-0.12047	-2.69897	0.007369	0.107482	chr7	136554731
cg20277670	0.088252	2.660033	0.008254	0.113092	chr5	175110375
cg20847733	-0.11372	-2.65733	0.008319	0.113391	chr7	136554160
cg15602074	-0.1231	-2.65089	0.008475	0.11435	chr6	78173720
cg24121172	-0.10466	-2.64834	0.008538	0.114643	chr20	60796414
cg19764436	-0.1183	-2.62936	0.009017	0.117483	chr22	23413260
cg03440850	0.088305	2.609055	0.009557	0.12059	chr11	46407440
cg13450708	-0.11137	-2.60499	0.009668	0.121158	chr7	154862157
cg11438011	-0.22668	-2.59931	0.009826	0.122193	chr5	148033882
cg13256912	0.082459	2.590673	0.01007	0.123386	chr3	11211081
cg15662768	-0.12027	-2.58627	0.010196	0.123952	chr20	60795818
cg02150536	0.0685	2.563569	0.010872	0.127622	chr3	11302179
cg06969845	0.094658	2.547101	0.011386	0.130396	chr5	175084250
cg14199144	0.079783	2.505538	0.012782	0.137144	chr11	113852043
cg07043494	0.134904	2.500044	0.012978	0.138024	chr3	11293681
cg16929739	0.080324	2.494865	0.013165	0.138979	chr3	11178593
cg02866106	-0.13835	-2.48589	0.013494	0.140546	chr7	136553110
cg06961323	0.100513	2.467839	0.014179	0.143366	chr11	113775900
cg24607283	0.065151	2.436885	0.015424	0.14846	chr3	11302249
cg26321066	0.102872	2.43498	0.015504	0.148743	chr3	183769987
cg15368905	-0.15581	-2.42176	0.016068	0.151206	chr6	78172337
cg00556112	-0.11151	-2.41025	0.016573	0.15328	chr11	637173
cg27579609	-0.10333	-2.41013	0.016578	0.15331	chr1	20003062
cg02701826	0.085598	2.367936	0.018553	0.161224	chr19	15903794
cg02928916	-0.10667	-2.35774	0.01906	0.163182	chr6	87647154
cg17176676	0.197834	2.353348	0.019282	0.164023	chr22	23441402
cg25058023	-0.11226	-2.34062	0.019939	0.166519	chr7	154863176
cg07826387	0.067206	2.305861	0.021834	0.172873	chr1	240072501
cg10454514	-0.09193	-2.30064	0.022131	0.173666	chr7	136553778
cg21286526	-0.08985	-2.29324	0.022559	0.175109	chr6	87646986
cg06020661	0.102349	2.282438	0.023198	0.177227	chr13	47472138
cg20102280	0.135874	2.242453	0.025698	0.185591	chr13	47470793
cg07318372	0.079941	2.238184	0.025979	0.186433	chr1	240071102
cg14458903	0.079405	2.228242	0.026642	0.188487	chr3	11203475

cg00902763	0.06242	2.217274	0.027391	0.19079	chr3	183750690
cg16109381	0.061626	2.212559	0.027718	0.191787	chr15	34339800
cg04694812	-0.09595	-2.20218	0.02845	0.194081	chr5	63257554
cg07162608	0.052352	2.191615	0.029214	0.196331	chr1	239828159
cg05157516	-0.10098	-2.1757	0.030396	0.199582	chr5	175085577
cg25763788	-0.10034	-2.16381	0.031307	0.202357	chr6	78172950
cg09714615	0.088676	2.151152	0.032302	0.20513	chr5	148033068
cg12449682	0.095329	2.149035	0.032471	0.2056	chr5	175111543
cg10538202	-0.1151	-2.14627	0.032693	0.206245	chr7	154863338
cg01991150	0.085258	2.143916	0.032883	0.206782	chr11	46407677
cg01616529	0.250727	2.137318	0.03342	0.208433	chr11	638424
cg27092248	0.056677	2.131038	0.033939	0.209897	chr3	11178798
cg04434491	0.1586	2.118186	0.035022	0.212659	chr19	16058640
cg02960016	0.056749	2.1172	0.035106	0.212936	chr3	11192067
cg09662616	0.066302	2.112729	0.03549	0.214027	chr1	240071263
cg01620540	0.080857	2.102545	0.03638	0.216318	chr13	47472064
cg08722720	-0.08093	-2.10194	0.036433	0.216451	chr15	34331557
cg02508664	-0.07949	-2.08027	0.038391	0.221908	chr6	87646738
cg00195561	0.070178	2.079007	0.038508	0.222181	chr11	46408584
cg21633143	-0.08971	-2.06642	0.039689	0.225285	chr7	154862021
cg18593668	-0.08498	-2.06182	0.040128	0.226475	chr22	23411986
cg01192538	0.0586	2.049749	0.041301	0.229473	chr13	47472050
cg15092168	-0.10159	-2.04869	0.041405	0.22974	chr5	63257873
cg04278702	-0.08016	-2.047	0.041571	0.230156	chr6	87647399
cg09623773	-0.13795	-2.03645	0.042626	0.232844	chr7	154863381
cg25368284	0.068407	2.03232	0.043045	0.233737	chr22	23438430
cg03855291	0.107968	2.024669	0.043831	0.235548	chr11	639423
cg04200192	0.057654	1.999763	0.046472	0.241786	chr3	183749414
cg12440040	0.060822	1.986534	0.047928	0.245004	chr6	87725662
cg01751188	-0.06499	-1.95971	0.051001	0.251857	chr22	23412225
cg02236913	0.064331	1.948378	0.052348	0.254776	chr1	20005598
cg26043322	0.069785	1.908595	0.057315	0.265138	chr1	159507162
cg13593758	0.054713	1.90422	0.057885	0.266337	chr3	11178365
cg12089079	0.107523	1.897969	0.058707	0.267883	chr13	47470350
cg12583095	-0.07404	-1.8876	0.060093	0.27079	chr10	92618141
cg07963181	-0.09819	-1.88573	0.060345	0.271271	chr3	11195902
cg24539937	0.047413	1.879637	0.061174	0.273027	chr7	136633191
cg06299284	0.280442	1.862176	0.063604	0.277997	chr11	636659
cg09297468	-0.0804	-1.85989	0.063928	0.278561	chr6	87647376
cg12793238	0.054532	1.849167	0.065466	0.281549	chr2	231977049
cg27447053	-0.08307	-1.84297	0.066368	0.283515	chr20	60795465
cg03024742	-0.08717	-1.83999	0.066805	0.284361	chr7	154863244
cg04799838	-0.06292	-1.82724	0.068706	0.287954	chr5	63256926
cg24134767	0.051051	1.818595	0.070019	0.290393	chr11	113845638
cg03472798	0.061606	1.817806	0.07014	0.29059	chr6	87646930
cg26393112	0.065999	1.812838	0.070905	0.291972	chr11	46408262
cg11811391	0.093054	1.803455	0.072369	0.294625	chr1	23520083
cg25388738	0.053598	1.803425	0.072374	0.294625	chr1	240071723

cg15919431	-0.10255	-1.80068	0.072807	0.295426	chr5	147862506
cg08831077	0.068417	1.78256	0.075719	0.300723	chr3	11178745
cg07630532	-0.07274	-1.77352	0.077207	0.303517	chr20	60795459
cg06251978	-0.08329	-1.75687	0.08001	0.308438	chr1	159507078
cg15068527	0.071155	1.745689	0.081939	0.311743	chr3	183817134
cg23757489	-0.07129	-1.72258	0.086045	0.319123	chr7	154862139
cg05327864	-0.07867	-1.71165	0.088045	0.322255	chr7	136554352
cg11158819	-0.0879	-1.70481	0.089315	0.32433	chr3	183817853
cg04315863	-0.05778	-1.69228	0.091681	0.328093	chr15	34330588
cg12816057	-0.06743	-1.68822	0.092459	0.329284	chr5	148034206
cg09798090	0.047004	1.680328	0.093985	0.331862	chr13	47472140
cg01459748	0.098499	1.677669	0.094504	0.332698	chr11	113817020
cg10644575	0.066827	1.66832	0.096347	0.335615	chr6	87725675
cg26253500	-0.07255	-1.66329	0.097349	0.337137	chr7	136641740
cg01004457	0.157934	1.663102	0.097388	0.337201	chr19	15851571
cg05596267	0.070561	1.620284	0.106273	0.350995	chr15	34331198
cg18200810	0.09681	1.617153	0.106948	0.35197	chr13	47472200
cg07839533	-0.09995	-1.60567	0.109451	0.35552	chr5	63257885
cg19630629	-0.07323	-1.60297	0.110044	0.356384	chr7	136556193
cg04291946	-0.07727	-1.59067	0.112788	0.360553	chr20	60791310
cg22442841	-0.07207	-1.58783	0.11343	0.361402	chr6	87646804
cg15835825	-0.06923	-1.58654	0.113722	0.361827	chr7	154862030
cg05942508	0.08766	1.584949	0.114083	0.362436	chr11	113846922
cg27527345	0.055462	1.578975	0.115447	0.364414	chr1	159505015
cg21960184	0.049467	1.57668	0.115975	0.365126	chr11	113804386
cg00365524	-0.05269	-1.57329	0.116756	0.366252	chr1	19992771
cg05506446	-0.0582	-1.56224	0.119337	0.369793	chr11	46409501
cg18412730	0.049523	1.561553	0.119499	0.370063	chr22	23435458
cg24101459	0.066313	1.561048	0.119618	0.370261	chr19	15919798
cg10323433	0.098316	1.554312	0.121216	0.37232	chr13	47471562
cg14059288	-0.04816	-1.55373	0.121355	0.372484	chr13	47468240
cg27068143	0.07709	1.549741	0.12231	0.373784	chr13	47471264
cg16921789	0.045571	1.539992	0.124668	0.37728	chr3	88031773
cg26333242	0.061369	1.509752	0.132211	0.387758	chr1	240072456
cg27530352	0.079769	1.501718	0.134273	0.390606	chr3	11294188
cg01953456	-0.04862	-1.49528	0.135945	0.392884	chr3	183817976
cg20991421	-0.05711	-1.49274	0.136609	0.393703	chr6	87646740
cg18708329	-0.05429	-1.48836	0.137758	0.395144	chr20	60795362
cg13530039	0.055653	1.483378	0.139075	0.396891	chr11	62689557
cg10685228	0.047584	1.468723	0.143007	0.401941	chr3	183750284
cg24714094	0.055234	1.456397	0.14638	0.406382	chr22	23467005
cg21200229	0.061374	1.441322	0.150588	0.411655	chr7	136588030
cg07116919	0.055154	1.434024	0.152658	0.414445	chr7	136558341
cg04042861	0.065302	1.423118	0.155792	0.418486	chr2	231989824
cg18236734	-0.05969	-1.41231	0.158947	0.422493	chr3	183817931
cg12974545	0.064145	1.404671	0.161204	0.425292	chr3	11198695
cg22614355	-0.05228	-1.40367	0.161501	0.425645	chr1	19991237
cg05551003	0.047351	1.393433	0.164571	0.429288	chr3	11267072

cg14944166	0.075128	1.386884	0.166557	0.431741	chr7	136686832
cg27022535	-0.08105	-1.38327	0.16766	0.433018	chr20	60794588
cg22368476	0.051179	1.357697	0.17563	0.442342	chr11	123814163
cg13666507	-0.07613	-1.35023	0.17801	0.445172	chr5	63257941
cg08726248	-0.1147	-1.33461	0.183067	0.451093	chr11	637032
cg19045531	0.056277	1.334346	0.183152	0.451165	chr19	15919022
cg06804815	0.046904	1.328506	0.18507	0.45331	chr22	23438116
cg17405853	0.042753	1.322656	0.187006	0.455607	chr5	175084085
cg26864526	0.039587	1.286875	0.199179	0.469509	chr3	11178064
cg20967585	-0.08812	-1.28684	0.19919	0.469519	chr7	154862524
cg15668767	0.036927	1.25785	0.209472	0.48088	chr11	46407019
cg11131902	0.048687	1.231756	0.219052	0.491248	chr5	175084710
cg15861585	-0.10628	-1.2307	0.219448	0.491613	chr11	637038
cg22812013	0.033224	1.200347	0.230998	0.503527	chr5	147830713
cg22471517	-0.04407	-1.19505	0.233059	0.505494	chr7	136553682
cg17850597	0.050029	1.193708	0.233581	0.506073	chr19	15917833
cg10418044	0.091532	1.190213	0.23495	0.507501	chr7	136553170
cg07915206	0.045957	1.182339	0.238053	0.510596	chr15	34260555
cg04493143	-0.08653	-1.18185	0.238248	0.510788	chr5	147862421
cg00147248	0.045261	1.180221	0.238893	0.511544	chr7	136568546
cg00987015	0.037819	1.159385	0.247266	0.520169	chr11	62688751
cg21330960	0.037734	1.134837	0.257394	0.530211	chr22	23415915
cg02440199	0.042057	1.12849	0.260059	0.532788	chr7	136691229
cg12068949	0.04968	1.122857	0.26244	0.535184	chr1	159506033
cg17645664	0.037132	1.121349	0.26308	0.535849	chr3	183750429
cg18243460	0.028669	1.120876	0.263281	0.536055	chr1	20005511
cg27075786	0.037527	1.119824	0.263728	0.536444	chr22	23438059
cg14345676	0.051309	1.117477	0.264728	0.537394	chr5	175109098
cg12580770	-0.07897	-1.11038	0.267767	0.54044	chr7	154861569
cg16738940	0.053001	1.107658	0.26894	0.541568	chr10	92575878
cg20887241	0.045977	1.106173	0.269581	0.54212	chr1	23522636
cg12418071	0.045113	1.102868	0.271012	0.543434	chr19	15919836
cg11335335	0.088323	1.098462	0.272927	0.545202	chr11	637885
cg12528649	0.044952	1.095826	0.274077	0.546342	chr11	46407116
cg00078348	-0.04592	-1.09108	0.276157	0.548315	chr11	113845487
cg24397241	0.035853	1.089129	0.277014	0.549081	chr3	11227410
cg20178075	0.048693	1.088256	0.277398	0.54948	chr11	113860607
cg02052721	0.040848	1.087205	0.277862	0.549913	chr1	23518539
cg03657040	0.03232	1.084932	0.278865	0.5508	chr5	175083981
cg00310588	0.04723	1.078168	0.281867	0.553696	chr3	183770589
cg11990309	-0.06164	-1.07131	0.284934	0.556599	chr6	87647644
cg15207662	0.038492	1.065334	0.287623	0.559089	chr5	175108315
cg05122082	-0.12746	-1.06237	0.288966	0.560368	chr14	20710905
cg12598837	0.026818	1.054827	0.292395	0.563425	chr11	113845788
cg04752263	0.043373	1.049607	0.294785	0.565702	chr20	60791717
cg18371750	0.034265	1.04089	0.298806	0.569417	chr5	175112799
cg14483391	-0.03158	-1.03601	0.301074	0.571561	chr3	183749227
cg17647537	-0.04287	-1.03492	0.301581	0.57203	chr11	113778957

cg06160669	0.035567	1.002422	0.316987	0.585959	chr1	240070975
cg05506829	0.05469	0.997686	0.319275	0.588032	chr13	47472349
cg06477056	-0.03997	-0.98526	0.325329	0.593365	chr5	175110609
cg26920451	0.038034	0.984715	0.325597	0.593651	chr15	34260956
cg16873130	0.054898	0.966419	0.334651	0.601412	chr7	136586950
cg02250787	-0.04723	-0.95816	0.338792	0.605082	chr13	47470989
cg16029939	-0.04883	-0.95182	0.341993	0.607728	chr11	640328
cg00967901	-0.05904	-0.94501	0.345453	0.610893	chr10	92617915
cg01331196	0.089388	0.938957	0.348545	0.613374	chr3	11287078
cg24137472	0.038786	0.938644	0.348705	0.613479	chr14	20710881
cg02579332	-0.0346	-0.93765	0.349216	0.61391	chr7	154875967
cg25599573	0.035109	0.933693	0.35125	0.615675	chr5	175108429
cg17200850	0.036752	0.931119	0.352577	0.616791	chr5	175107097
cg12296860	-0.02683	-0.93042	0.352941	0.617128	chr11	46409428
cg06718003	0.060447	0.926921	0.354749	0.618686	chr19	15851771
cg22347705	0.025744	0.905323	0.366056	0.628201	chr22	23438507
cg00170438	0.020399	0.904417	0.366535	0.628603	chr20	60792389
cg07075299	0.050839	0.898358	0.36975	0.631349	chr13	47472360
cg15108640	-0.0387	-0.88943	0.374521	0.635186	chr1	240071966
cg26135506	0.062268	0.87308	0.383352	0.642088	chr10	92617562
cg01406506	0.035726	0.867337	0.386485	0.644595	chr5	147938667
cg17578539	-0.02855	-0.85396	0.393841	0.650436	chr5	175104957
cg13069918	0.048065	0.853253	0.394233	0.650755	chr1	20005744
cg09362722	0.039449	0.845697	0.398429	0.654177	chr7	136626256
cg01586609	0.029526	0.827866	0.408437	0.661812	chr11	113846937
cg00378234	0.034103	0.8165	0.414894	0.666825	chr19	15904416
cg03056854	-0.03659	-0.80488	0.421558	0.671852	chr1	20005414
cg10772974	-0.02587	-0.80119	0.423688	0.673641	chr15	34332433
cg10605520	-0.03654	-0.79554	0.426958	0.676192	chr20	60796141
cg27051089	-0.03255	-0.79516	0.42718	0.676311	chr6	87653726
cg25150440	-0.05017	-0.7876	0.431581	0.679484	chr7	136553088
cg03321592	-0.04096	-0.7686	0.442767	0.687872	chr1	19991676
cg12749468	0.039275	0.767921	0.443168	0.688178	chr3	183755507
cg18190847	-0.02734	-0.76687	0.443794	0.688706	chr3	11195751
cg23300659	-0.04283	-0.76573	0.444469	0.689187	chr7	136553822
cg00363114	0.031538	0.758096	0.449018	0.692623	chr11	113844663
cg22863118	0.02311	0.753779	0.451602	0.694578	chr7	136701166
cg01920563	0.031421	0.736772	0.461865	0.702553	chr7	136648231
cg27090784	-0.05404	-0.71663	0.47419	0.711419	chr5	147862681
cg15888097	0.018592	0.716297	0.474392	0.711554	chr6	87646462
cg19116351	0.047537	0.712962	0.476451	0.713072	chr3	88031048
cg17637877	0.028995	0.709667	0.478489	0.714633	chr11	113779788
cg00973677	-0.02528	-0.7054	0.481133	0.716612	chr7	136553595
cg17723143	-0.08499	-0.70168	0.483452	0.718166	chr5	148033473
cg05888433	0.034683	0.701258	0.483712	0.718376	chr14	20711344
cg08258494	0.026098	0.696562	0.486643	0.720416	chr1	239987344
cg11553153	0.025736	0.695018	0.487608	0.721137	chr15	34348521
cg01791421	0.024734	0.679221	0.497547	0.72815	chr1	19996240

cg00307530	0.022685	0.672622	0.501731	0.731059	chr5	147834517
cg17571559	-0.01994	-0.67041	0.50314	0.732053	chr3	11267525
cg24875857	0.019743	0.669049	0.504004	0.732653	chr3	183817230
cg17564844	0.031298	0.668919	0.504087	0.732703	chr22	23413784
cg01468656	-0.02651	-0.66665	0.505533	0.73359	chr1	19991678
cg05680531	-0.04368	-0.6645	0.506904	0.734461	chr7	136553327
cg17405012	-0.03846	-0.65909	0.51037	0.736937	chr7	136553263
cg06457736	0.029678	0.654098	0.513574	0.7391	chr3	11178683
cg07648740	0.028109	0.63753	0.52429	0.74641	chr7	154864630
cg24682621	0.02738	0.636965	0.524657	0.746585	chr1	239918125
cg03360907	0.025208	0.617316	0.537518	0.755132	chr11	62688748
cg03737442	0.021649	0.609855	0.542442	0.758561	chr3	11177295
cg05762326	-0.01943	-0.58418	0.55956	0.769702	chr10	92591168
cg13690703	0.027536	0.57611	0.564994	0.773348	chr3	88030644
cg00308665	0.02668	0.566101	0.571769	0.777768	chr13	47469654
cg12452364	-0.03066	-0.55374	0.580189	0.783319	chr20	60795156
cg20678835	-0.02039	-0.54941	0.583153	0.785079	chr1	23521332
cg26332534	-0.0248	-0.54432	0.586643	0.787306	chr10	92618063
cg12513379	0.026732	0.543373	0.587296	0.787727	chr19	15838397
cg08372315	0.018868	0.520783	0.602921	0.797249	chr11	113844382
cg16543009	0.050319	0.512997	0.60835	0.800529	chr15	34331514
cg23881368	0.035609	0.508982	0.611157	0.802361	chr13	47472343
cg00576550	-0.01643	-0.50813	0.611751	0.802733	chr3	183749212
cg17483297	0.024056	0.501173	0.616635	0.805597	chr5	175084743
cg00783712	0.018807	0.491109	0.623726	0.809715	chr1	239974513
cg26153642	-0.01915	-0.48762	0.626192	0.811279	chr3	183818368
cg12639324	-0.02508	-0.48495	0.62808	0.812447	chr10	92617735
cg19699807	0.02479	0.484031	0.628734	0.812959	chr19	16060211
cg01625621	-0.01506	-0.47612	0.634352	0.816483	chr15	34260433
cg27230009	0.011177	0.464833	0.642404	0.82142	chr3	11241399
cg24661173	-0.019	-0.46372	0.643198	0.821937	chr7	154866132
cg01616732	-0.02628	-0.44393	0.657431	0.83025	chr20	60795457
cg25271892	0.02271	0.426693	0.669924	0.837528	chr11	62690462
cg09863950	-0.01516	-0.42501	0.671148	0.838306	chr1	19990768
cg10650018	-0.02313	-0.42172	0.673548	0.839647	chr10	92616759
cg02527199	-0.02652	-0.41348	0.679562	0.843217	chr5	175085245
cg11773243	-0.01323	-0.40768	0.683817	0.845678	chr7	136613718
cg18853490	-0.0201	-0.40529	0.685566	0.846611	chr1	239882840
cg17537380	0.009957	0.399483	0.689835	0.848942	chr6	87649596
cg11514288	-0.01796	-0.38	0.704227	0.856917	chr13	47471197
cg02517524	-0.01413	-0.37983	0.704356	0.856997	chr22	23412814
cg08622198	-0.0084	-0.37249	0.709807	0.860007	chr1	239979505
cg23343875	-0.02353	-0.3722	0.710019	0.860108	chr19	16058370
cg10842339	-0.02549	-0.36005	0.719076	0.864965	chr1	240071807
cg16188532	-0.02473	-0.35212	0.725007	0.868268	chr13	47471090
cg11666515	0.013181	0.348875	0.72744	0.869571	chr19	15919834
cg09666573	-0.01174	-0.34177	0.732772	0.872415	chr3	11267627
cg21232620	-0.01453	-0.32766	0.743411	0.878324	chr6	78172192

cg14841965	-0.01214	-0.31976	0.749382	0.881652	chr11	123814849
cg18859248	-0.01906	-0.29612	0.767352	0.891568	chr20	60791502
cg15625631	-0.01495	-0.27788	0.781302	0.898863	chr11	123814972
cg23424273	-0.02583	-0.27658	0.782302	0.899357	chr6	78173227
cg25276126	0.008876	0.271389	0.786288	0.901484	chr1	20005715
cg21536328	0.01059	0.255235	0.798725	0.908217	chr1	23522665
cg01614101	-0.01075	-0.24986	0.802878	0.910335	chr11	113778771
cg09133032	0.022697	0.244499	0.807019	0.912473	chr11	640094
cg01274715	0.010843	0.243623	0.807697	0.912789	chr10	92618033
cg26724798	0.008414	0.225844	0.821484	0.920002	chr11	113844828
cg00456868	0.01503	0.216928	0.828419	0.923302	chr15	34331390
cg09575258	-0.00654	-0.21144	0.832696	0.925463	chr5	175084718
cg22806527	0.008594	0.208437	0.835036	0.926573	chr5	175087093
cg22075328	0.007742	0.203518	0.838875	0.928545	chr22	23412381
cg25843439	-0.01005	-0.18431	0.853905	0.935555	chr19	15852574
cg02762115	-0.00907	-0.18346	0.854567	0.935879	chr11	640446
cg06096336	0.00743	0.180474	0.856908	0.936897	chr2	231989800
cg01808284	-0.03723	-0.17486	0.86131	0.938858	chr5	148031958
cg18271969	0.006466	0.169993	0.865136	0.940729	chr3	183771499
cg03448301	-0.00647	-0.16291	0.870704	0.943216	chr11	62678778
cg23720528	-0.00532	-0.16247	0.87105	0.943352	chr10	92501798
cg04607131	-0.00757	-0.15887	0.873887	0.944921	chr1	19990783
cg13245440	0.008638	0.153626	0.878013	0.946851	chr1	19990624
cg09361691	-0.00816	-0.15178	0.879465	0.947644	chr13	47471169
cg16126186	0.004699	0.150207	0.880708	0.948241	chr18	22053986
cg15484742	0.007777	0.147267	0.883025	0.949234	chr1	23519884
cg08417719	-0.00427	-0.14153	0.887554	0.951317	chr11	123814620
cg07579109	0.00405	0.138389	0.89003	0.952419	chr22	23437461
cg00023024	-0.00514	-0.13703	0.891102	0.95286	chr1	23521272
cg10876621	0.005457	0.120446	0.904214	0.958796	chr10	92565207
cg16057587	-0.00346	-0.10715	0.914748	0.963491	chr7	136698566
cg15015426	0.006025	0.094333	0.924911	0.968123	chr1	159506559
cg10953410	-0.00438	-0.09034	0.928081	0.969394	chr11	113846918
cg02027079	0.004718	0.085009	0.932314	0.971257	chr13	47471705
cg10276834	-0.0043	-0.07823	0.937697	0.973854	chr7	136575360
cg14986832	-0.00648	-0.06867	0.9453	0.977036	chr22	23412408
cg11247289	0.002473	0.059771	0.95238	0.980022	chr1	19991707
cg01296705	0.001848	0.059353	0.952712	0.980174	chr5	175108269
cg27143370	0.003074	0.052058	0.958519	0.982799	chr20	60795923
cg01812577	0.001647	0.051549	0.958924	0.982952	chr11	62689122
cg17660833	0.002072	0.047433	0.962201	0.984329	chr3	11267020
cg06476131	-0.00298	-0.03867	0.969179	0.98722	chr13	47471052
cg11788586	0.000679	0.031678	0.974751	0.989754	chr7	136606294
cg27551227	-0.00219	-0.03121	0.975127	0.989955	chr7	154877217
cg08872493	0.001823	0.031005	0.975287	0.990017	chr1	23521417
cg12243453	-0.00099	-0.02801	0.977675	0.991059	chr11	113775400
cg12775613	0.00107	0.025346	0.979797	0.991809	chr3	88040034
