# Supplementary material for: Differential DNA Methylation of the Serotonin Receptor Signaling and Glutamatergic Synapse Pathways in Adult Twins Born Preterm
Source: Genes (Basel). 2026 Jun 10;17(6):683. doi: 10.3390/genes17060683 (PMC13299586; doi:10.3390/genes17060683)
Supplement: Supplementary file 1 [file genes-17-00683-s001.zip › Supplementary Table S6_serotonin_old.pdf]

CpG	logFC	t	P.Value	adj.P.Val	chr	pos
cg15861585	0.363452	6.195188	2.02E-09	1.04E-05	chr11	637038
cg06291867	0.179591	5.441014	1.14E-07	0.000117	chr10	92617162
cg15662768	0.165958	5.106681	6.00E-07	0.000299	chr20	60795818
cg18023598	0.152312	5.106624	6.00E-07	0.000299	chr1	19992504
cg04842426	0.333091	4.947566	1.28E-06	0.000484	chr7	136555777
cg08186362	0.428353	4.897088	1.63E-06	0.000563	chr20	60794868
cg02762115	0.159766	4.641267	5.28E-06	0.001115	chr11	640446
cg20847733	0.135584	4.487575	1.05E-05	0.001718	chr7	136554160
cg19500607	-0.13576	-4.47592	1.10E-05	0.001772	chr5	148034319
cg00903099	0.138004	4.409599	1.47E-05	0.00214	chr7	154862441
cg08726248	0.264367	4.390736	1.59E-05	0.002255	chr11	637032
cg02928916	0.138342	4.332286	2.04E-05	0.002638	chr6	87647154
cg10323433	-0.1889	-4.24939	2.90E-05	0.00326	chr13	47471562
cg11438011	0.261251	4.229723	3.15E-05	0.00343	chr5	148033882
cg22059812	0.13492	4.18471	3.80E-05	0.003857	chr1	19992564
cg17176676	-0.24649	-4.14369	4.51E-05	0.004285	chr22	23441402
cg27068143	-0.14178	-4.0443	6.76E-05	0.005556	chr13	47471264
cg13077519	0.129943	3.955748	9.63E-05	0.007023	chr6	78174007
cg02266732	0.130667	3.917834	0.000112	0.007711	chr5	63257710
cg24575234	0.130021	3.913025	0.000114	0.007795	chr7	136553884
cg09297468	0.117394	3.835721	0.000154	0.009507	chr6	87647376
cg11335335	0.214854	3.786797	0.000186	0.010637	chr11	637885
cg06299284	0.403859	3.785472	0.000187	0.010661	chr11	636659
cg08323651	0.131136	3.773371	0.000196	0.010963	chr7	136553855
cg27549720	0.179039	3.771402	0.000197	0.011025	chr1	19992167
cg07839533	0.165205	3.750445	0.000214	0.011553	chr5	63257885
cg24228819	0.122288	3.746385	0.000217	0.01166	chr7	136553868
cg00840960	0.118317	3.720989	0.000239	0.012367	chr5	148034030
cg27051089	0.101528	3.506641	0.000527	0.02054	chr6	87653726
cg19764436	0.112362	3.492003	0.000555	0.021263	chr22	23413260
cg01616529	0.291586	3.489469	0.00056	0.021378	chr11	638424
cg07102705	0.126054	3.488308	0.000563	0.021452	chr5	148033896
cg06825142	0.112719	3.455326	0.000633	0.023049	chr11	637170
cg16280141	0.12368	3.426357	0.000701	0.024662	chr5	63257753
cg16029939	0.124284	3.419527	0.000719	0.025024	chr11	640328
cg21127286	0.094339	3.383136	0.000817	0.027115	chr7	136555154
cg00183186	0.1244	3.382739	0.000818	0.027135	chr6	78174065
cg24845274	0.114361	3.369159	0.000858	0.027983	chr7	136555697
cg07212818	0.156766	3.327821	0.00099	0.0306	chr11	638076
cg19630629	0.107406	3.304883	0.001071	0.032208	chr7	136556193
cg14433983	0.173934	3.188932	0.001587	0.041242	chr11	636460
cg22442841	0.101059	3.123888	0.001968	0.047173	chr6	87646804
cg24121172	0.08867	3.123725	0.001969	0.047185	chr20	60796414
cg05762326	0.073621	3.117925	0.002007	0.04771	chr10	92591168
cg07664198	0.09011	3.104145	0.0021	0.049048	chr7	136553882
cg20102280	-0.13374	-3.08234	0.002254	0.051289	chr13	47470793
cg13102079	0.098239	3.060861	0.002417	0.053574	chr7	136554731

cg07915206	-0.08364	-3.02184	0.00274	0.057873	chr15	34260555
cg05327864	0.09885	3.012159	0.002826	0.058998	chr7	136554352
cg07963181	0.110471	2.966614	0.003265	0.064426	chr3	11195902
cg25632105	0.110442	2.935711	0.003598	0.068279	chr7	136553728
cg05756489	0.144625	2.915753	0.003829	0.070894	chr10	92616870
cg03909863	0.321401	2.875318	0.004339	0.076758	chr11	638404
cg03024742	0.096111	2.833811	0.004927	0.082849	chr7	154863244
cg09133032	0.186579	2.823685	0.005081	0.084442	chr11	640094
cg27579609	0.085063	2.757978	0.006191	0.095002	chr1	20003062
cg02508664	0.075606	2.757355	0.006202	0.095106	chr6	87646738
cg05157516	0.091606	2.748528	0.006367	0.096699	chr5	175085577
cg05650628	-0.05782	-2.68342	0.007712	0.108653	chr11	62677384
cg00456868	-0.1316	-2.66456	0.008146	0.112005	chr15	34331390
cg17660833	0.082738	2.656517	0.008339	0.113562	chr3	11267020
cg21633143	0.082795	2.655679	0.008359	0.113751	chr7	154862021
cg14345676	0.08703	2.653019	0.008423	0.114295	chr5	175109098
cg00023024	-0.06925	-2.58763	0.010157	0.127825	chr1	23521272
cg12449682	-0.0825	-2.58649	0.01019	0.128103	chr5	175111543
cg07579109	0.05382	2.577908	0.010441	0.129819	chr22	23437461
cg27527345	-0.06486	-2.5771	0.010465	0.129999	chr1	159505015
cg05942508	-0.1015	-2.5609	0.010953	0.13334	chr11	113846922
cg22863118	-0.05494	-2.50802	0.012695	0.145466	chr7	136701166
cg07162608	-0.04294	-2.49683	0.013093	0.148138	chr1	239828159
cg12103152	0.076902	2.469954	0.014097	0.154392	chr6	78173200
cg03138127	-0.15065	-2.46018	0.014478	0.156799	chr2	231989146
cg00902763	-0.04985	-2.45857	0.014542	0.157203	chr3	183750690
cg12825070	0.135882	2.441182	0.015246	0.161666	chr5	148033708
cg05551003	0.058449	2.399279	0.017067	0.172339	chr3	11267072
cg27615388	0.113591	2.394934	0.017267	0.173473	chr5	63257092
cg00556112	0.07858	2.352623	0.019319	0.184624	chr11	637173
cg10454514	0.067663	2.347416	0.019586	0.186023	chr7	136553778
cg22471517	0.061584	2.330118	0.020496	0.190869	chr7	136553682
cg05717871	0.144295	2.312941	0.021435	0.195664	chr11	638507
cg04278702	0.064826	2.298428	0.022259	0.199732	chr6	87647399
cg15602074	0.07724	2.298069	0.022279	0.199765	chr6	78173720
cg00078348	0.069161	2.293165	0.022564	0.201179	chr11	113845487
cg07648740	-0.07207	-2.28396	0.023107	0.20383	chr7	154864630
cg22075328	-0.0617	-2.26755	0.024103	0.208754	chr22	23412381
cg07833420	0.070749	2.26483	0.024271	0.209424	chr6	87647147
cg15092168	0.080763	2.260618	0.024535	0.210823	chr5	63257873
cg01808284	0.340542	2.235559	0.026152	0.218038	chr5	148031958
cg22471401	-0.04255	-2.23326	0.026305	0.218729	chr3	183824717
cg04694812	0.069916	2.22408	0.026924	0.221497	chr5	63257554
cg11298960	0.08054	2.218279	0.027321	0.223252	chr7	154862548
cg21960184	0.049363	2.188944	0.029409	0.232024	chr11	113804386
cg03855291	0.083523	2.172875	0.030611	0.237104	chr11	639423
cg23247337	-0.06554	-2.15914	0.031671	0.24141	chr3	11178285
cg20991421	0.058697	2.134641	0.03364	0.249464	chr6	87646740

cg09662616	-0.04778	-2.10969	0.035754	0.257888	chr1	240071263
cg17723143	0.181623	2.092164	0.037305	0.263563	chr5	148033473
cg04200192	0.043462	2.090332	0.037471	0.264111	chr3	183749414
cg27143370	0.088313	2.088576	0.03763	0.264761	chr20	60795923
cg16929739	-0.04838	-2.07544	0.038839	0.269363	chr3	11178593
cg06531741	0.065112	2.072243	0.039139	0.270388	chr11	113775450
cg04493143	0.108419	2.062264	0.040086	0.273837	chr5	147862421
cg11615755	0.068171	2.052697	0.041012	0.277192	chr5	63257867
cg12243453	0.051971	2.045232	0.041747	0.279856	chr11	113775400
cg00454577	0.055161	2.018096	0.044515	0.289682	chr6	87646972
cg01331196	0.137593	2.014002	0.044945	0.29103	chr3	11287078
cg00576550	0.046373	2.000849	0.046354	0.295873	chr3	183749212
cg00170438	-0.03235	-1.99853	0.046606	0.296759	chr20	60792389
cg08614481	0.063222	1.975065	0.049222	0.305187	chr6	78173250
cg18200810	-0.0841	-1.95081	0.052056	0.313913	chr13	47472200
cg04427003	0.056722	1.932727	0.054257	0.320572	chr5	63257499
cg01004457	0.131486	1.921907	0.055611	0.324712	chr19	15851571
cg13666507	0.077081	1.900349	0.058393	0.332452	chr5	63257941
cg09640960	0.065138	1.899074	0.058561	0.332858	chr20	60794676
cg21286526	0.053713	1.894387	0.059182	0.334484	chr6	87646986
cg19503977	0.04863	1.891635	0.05955	0.335649	chr6	78173287
cg09714615	0.055787	1.871754	0.062261	0.343419	chr5	148033068
cg18708329	0.048929	1.862987	0.063489	0.346655	chr20	60795362
cg00365524	0.044394	1.839854	0.066826	0.355677	chr1	19992771
cg21232620	-0.0577	-1.8134	0.070818	0.366074	chr6	78172192
cg26321066	0.055467	1.81113	0.07117	0.366982	chr3	183769987
cg04607131	-0.06181	-1.80867	0.071553	0.36784	chr1	19990783
cg17483297	-0.06195	-1.79787	0.073252	0.372249	chr5	175084743
cg18853490	-0.06389	-1.79421	0.073835	0.373639	chr1	239882840
cg25763788	0.059086	1.760869	0.079329	0.386868	chr6	78172950
cg06457736	-0.05725	-1.75658	0.08006	0.388459	chr3	11178683
cg15108640	0.054727	1.750104	0.081173	0.391053	chr1	240071966
cg23881368	-0.08789	-1.74938	0.081297	0.39132	chr13	47472343
cg21536328	0.052016	1.74642	0.081812	0.392415	chr1	23522665
cg09863441	0.056702	1.743181	0.082377	0.393737	chr6	87647129
cg25368284	-0.04236	-1.74065	0.082821	0.394642	chr22	23438430
cg10876621	0.055526	1.707022	0.088903	0.408112	chr10	92565207
cg15835825	0.053709	1.706756	0.088953	0.408186	chr7	154862030
cg25150440	0.078025	1.704331	0.089405	0.409058	chr7	136553088
cg26043322	-0.04486	-1.69786	0.090622	0.411516	chr1	159507162
cg08208133	0.042737	1.673948	0.095234	0.421297	chr11	113848161
cg21200229	0.051116	1.665534	0.096901	0.424659	chr7	136588030
cg17637877	0.048789	1.661303	0.097748	0.426237	chr11	113779788
cg12793238	-0.03531	-1.65739	0.098536	0.427894	chr2	231977049
cg27092248	-0.03175	-1.64884	0.100278	0.431553	chr3	11178798
cg09863950	-0.04071	-1.58781	0.113435	0.457189	chr1	19990768
cg25599573	-0.04283	-1.58282	0.114568	0.459143	chr5	175108429
cg15668767	-0.03335	-1.57686	0.115932	0.461544	chr11	46407019

cg15015426	-0.07213	-1.57172	0.117121	0.463795	chr1	159506559
cg02866106	0.063517	1.571493	0.117174	0.463872	chr7	136553110
cg13256912	-0.03619	-1.56419	0.118879	0.466938	chr3	11211081
cg16543009	-0.10999	-1.5599	0.119889	0.468584	chr15	34331514
cg23764129	-0.0321	-1.53919	0.124865	0.477242	chr11	113846017
cg13593758	0.031706	1.525673	0.128196	0.483068	chr3	11178365
cg05506829	-0.05999	-1.52013	0.129582	0.485402	chr13	47472349
cg27530352	-0.0581	-1.51582	0.130669	0.487363	chr3	11294188
cg01791421	0.039706	1.515808	0.130672	0.487366	chr1	19996240
cg18593668	0.044958	1.506407	0.133066	0.491353	chr22	23411986
cg06969845	-0.04044	-1.49698	0.135501	0.495324	chr5	175084250
cg09361691	0.057781	1.494362	0.136183	0.496295	chr13	47471169
cg17647537	0.044444	1.489747	0.137393	0.498203	chr11	113778957
cg08622198	0.023956	1.477273	0.140703	0.503295	chr1	239979505
cg23757489	0.04417	1.476983	0.14078	0.503446	chr7	154862139
cg14632899	-0.02713	-1.47049	0.142529	0.506152	chr11	62678618
cg22812013	-0.02916	-1.46148	0.144981	0.510105	chr5	147830713
cg18019017	0.032918	1.437018	0.151806	0.520421	chr6	78173408
cg27022535	-0.05983	-1.41538	0.158045	0.529491	chr20	60794588
cg10276834	-0.05591	-1.41308	0.15872	0.530536	chr7	136575360
cg26724798	0.037856	1.412907	0.15877	0.530625	chr11	113844828
cg07043494	-0.05448	-1.38865	0.166021	0.540495	chr3	11293681
cg13450708	0.042968	1.38116	0.168308	0.54358	chr7	154862157
cg03440850	-0.03399	-1.37995	0.168679	0.544129	chr11	46407440
cg17564844	0.046135	1.369831	0.171814	0.548703	chr22	23413784
cg05506446	0.036646	1.36179	0.174336	0.552279	chr11	46409501
cg24397241	-0.03207	-1.35157	0.177579	0.55648	chr3	11227410
cg10418044	-0.07484	-1.34967	0.178188	0.557318	chr7	136553170
cg02440199	0.036244	1.348973	0.178413	0.557589	chr7	136691229
cg24101459	-0.04127	-1.3447	0.179788	0.559461	chr19	15919798
cg00308665	-0.04515	-1.33102	0.184244	0.565092	chr13	47469654
cg14458903	-0.03419	-1.32203	0.187214	0.568649	chr3	11203475
cg14483391	0.028966	1.318339	0.188445	0.570155	chr3	183749227
cg07075299	-0.05355	-1.31347	0.190076	0.57211	chr13	47472360
cg10979181	-0.05007	-1.29613	0.195975	0.579542	chr7	136586606
cg09575258	-0.02865	-1.28736	0.199008	0.583346	chr5	175084718
cg13530039	-0.03487	-1.2866	0.199276	0.583722	chr11	62689557
cg03986968	-0.03175	-1.27327	0.203955	0.588981	chr15	34330894
cg06804815	-0.03245	-1.27317	0.203993	0.589029	chr22	23438116
cg05919907	-0.02794	-1.24116	0.215564	0.602452	chr5	175105350
cg12513379	-0.04363	-1.23165	0.219092	0.606604	chr19	15838397
cg09386376	0.071264	1.222404	0.222561	0.610212	chr11	638939
cg18412730	-0.02797	-1.21998	0.223478	0.611266	chr22	23435458
cg08722720	0.033812	1.210737	0.226996	0.615259	chr15	34331557
cg27075786	0.02921	1.208316	0.227924	0.61623	chr22	23438059
cg02052721	-0.03239	-1.19527	0.232971	0.621559	chr1	23518539
cg20967585	0.059074	1.194985	0.233083	0.621668	chr7	154862524
cg24134767	-0.02423	-1.19238	0.234101	0.622743	chr11	113845638

cg11990309	0.049058	1.18217	0.23812	0.627224	chr6	87647644
cg17645664	-0.02816	-1.17865	0.239515	0.628788	chr3	183750429
cg09798090	-0.02357	-1.16473	0.2451	0.634733	chr13	47472140
cg23424273	0.078286	1.164429	0.245221	0.634856	chr6	78173227
cg27447053	0.037178	1.13883	0.255727	0.645004	chr20	60795465
cg17805202	-0.03369	-1.12593	0.261137	0.650203	chr1	239937440
cg02250787	0.039965	1.12421	0.261867	0.650917	chr13	47470989
cg08831077	-0.03087	-1.11076	0.267606	0.656196	chr3	11178745
cg01406506	-0.03298	-1.11048	0.267727	0.656305	chr5	147938667
cg00147248	-0.03071	-1.10952	0.268138	0.65672	chr7	136568546
cg06020661	-0.03598	-1.10433	0.270379	0.658814	chr13	47472138
cg21330960	0.026366	1.098685	0.27283	0.661045	chr22	23415915
cg01991150	0.0317	1.098187	0.273046	0.661209	chr11	46407677
cg15888097	-0.0205	-1.0958	0.274087	0.662291	chr6	87646462
cg19699807	0.040318	1.092757	0.27542	0.663634	chr19	16060211
cg10605520	0.03609	1.089801	0.276718	0.664632	chr20	60796141
cg02389195	-0.02233	-1.08416	0.279206	0.666851	chr5	175088015
cg00310588	-0.03422	-1.08242	0.279977	0.667437	chr3	183770589
cg18243460	-0.01992	-1.07888	0.281549	0.668857	chr1	20005511
cg11702866	0.021873	1.070149	0.285455	0.672311	chr3	11267098
cg03657040	0.022979	1.068838	0.286044	0.672884	chr5	175083981
cg07116919	-0.02934	-1.05531	0.292174	0.678598	chr7	136558341
cg24607283	-0.02051	-1.05435	0.292614	0.679008	chr3	11302249
cg25058023	0.036763	1.054225	0.29267	0.679082	chr7	154863176
cg04752263	0.031346	1.051212	0.294049	0.68015	chr20	60791717
cg14199144	-0.02389	-1.03038	0.303704	0.688324	chr11	113852043
cg06160669	-0.02632	-1.02792	0.304855	0.689383	chr1	240070975
cg13245440	0.041562	1.026213	0.305658	0.690133	chr1	19990624
cg26333242	-0.02999	-1.02032	0.308441	0.692208	chr1	240072456
cg10842339	-0.05188	-1.0172	0.309917	0.693284	chr1	240071807
cg17571559	0.02141	0.998289	0.318983	0.700944	chr3	11267525
cg05888433	-0.03552	-0.99617	0.320012	0.70177	chr14	20711344
cg04799838	0.024848	0.995925	0.320129	0.701862	chr5	63256926
cg03321592	0.03787	0.985425	0.325248	0.705793	chr1	19991676
cg11788586	-0.01519	-0.98353	0.326179	0.706776	chr7	136606294
cg12440040	-0.02169	-0.97661	0.329586	0.709263	chr6	87725662
cg24539937	-0.01753	-0.95868	0.338528	0.716151	chr7	136633191
cg06096336	0.028166	0.949499	0.343169	0.719555	chr2	231989800
cg12639324	0.03532	0.947608	0.344129	0.72028	chr10	92617735
cg25388738	-0.02012	-0.93417	0.351004	0.725393	chr1	240071723
cg12974545	-0.03074	-0.9312	0.352537	0.726537	chr3	11198695
cg26393112	0.024459	0.927081	0.354666	0.728094	chr11	46408262
cg24682621	0.028456	0.918094	0.359343	0.731764	chr1	239918125
cg03360907	0.026966	0.915854	0.360515	0.732635	chr11	62688748
cg02960016	-0.01766	-0.90726	0.365033	0.736084	chr3	11192067
cg20277670	0.021878	0.903986	0.366764	0.737581	chr5	175110375
cg00195561	0.02205	0.899719	0.369027	0.73922	chr11	46408584
cg14059288	0.020086	0.895478	0.371284	0.74085	chr13	47468240

cg11247289	-0.02621	-0.87892	0.380182	0.746996	chr1	19991707
cg18859248	0.040073	0.863854	0.388392	0.75296	chr20	60791502
cg00363114	-0.02552	-0.85044	0.395794	0.758108	chr11	113844663
cg10644575	-0.02459	-0.84758	0.39738	0.759231	chr6	87725675
cg09362722	0.028473	0.845867	0.398334	0.759916	chr7	136626256
cg06961323	-0.02489	-0.83914	0.402089	0.76236	chr11	113775900
cg16738940	-0.02854	-0.82586	0.409574	0.767161	chr10	92575878
cg12583095	0.022934	0.806139	0.420832	0.774294	chr10	92618141
cg20887241	-0.02389	-0.79562	0.426913	0.778363	chr1	23522636
cg15207662	-0.02075	-0.79505	0.427244	0.778531	chr5	175108315
cg01616732	0.033427	0.7831	0.434216	0.782494	chr20	60795457
cg01468656	0.022376	0.780063	0.435999	0.783644	chr1	19991678
cg01586609	0.019892	0.77277	0.440297	0.786368	chr11	113846937
cg17220584	-0.02519	-0.77148	0.44106	0.786861	chr5	148017424
cg02150536	0.01503	0.771456	0.441074	0.786868	chr3	11302179
cg04291946	-0.02709	-0.77029	0.441763	0.787263	chr20	60791310
cg23300659	0.030587	0.757858	0.44916	0.791525	chr7	136553822
cg22806527	0.022208	0.747093	0.455621	0.795263	chr5	175087093
cg08872493	-0.03151	-0.74348	0.4578	0.796556	chr1	23521417
cg15919431	0.03058	0.740631	0.459525	0.797729	chr5	147862506
cg10650018	0.02899	0.732868	0.464239	0.800511	chr10	92616759
cg12089079	0.029645	0.721309	0.471309	0.804728	chr13	47470350
cg26920451	0.019843	0.711373	0.477433	0.807977	chr15	34260956
cg06718003	0.033075	0.702431	0.482982	0.811068	chr19	15851771
cg15368905	0.032608	0.695872	0.487074	0.813288	chr6	78172337
cg14986832	-0.04725	-0.6948	0.487748	0.813668	chr22	23412408
cg16126186	-0.01566	-0.69432	0.488042	0.813772	chr18	22053986
cg12418071	0.020359	0.688834	0.491486	0.815757	chr19	15919836
cg13069918	-0.0278	-0.68356	0.494808	0.817319	chr1	20005744
cg11773243	-0.0154	-0.65789	0.511139	0.826208	chr7	136613718
cg22347705	-0.01346	-0.65565	0.512578	0.826912	chr22	23438507
cg01257383	-0.01025	-0.64528	0.519264	0.8303	chr11	46408399
cg00378234	-0.01933	-0.64095	0.522071	0.8315	chr19	15904416
cg20178075	0.020548	0.635539	0.525585	0.833218	chr11	113860607
cg02527199	0.029322	0.63393	0.526633	0.833913	chr5	175085245
cg12598837	0.011506	0.626305	0.531614	0.836428	chr11	113845788
cg08258494	0.016772	0.620228	0.535602	0.838535	chr1	239987344
cg26332534	-0.02021	-0.61459	0.539317	0.840256	chr10	92618063
cg11666515	0.016689	0.612418	0.540748	0.840848	chr19	15919834
cg08372315	0.015767	0.603184	0.546864	0.843759	chr11	113844382
cg27090784	-0.03281	-0.60274	0.547157	0.843907	chr5	147862681
cg00973677	0.015437	0.596647	0.551215	0.845956	chr7	136553595
cg16109381	0.011921	0.588435	0.556705	0.848439	chr15	34339800
cg25271892	-0.02253	-0.58677	0.557822	0.848957	chr11	62690462
cg18236734	-0.01785	-0.58357	0.559971	0.850023	chr3	183817931
cg03056854	-0.01913	-0.58302	0.560336	0.850216	chr1	20005414
cg14944166	0.022455	0.57285	0.567196	0.853561	chr7	136686832
cg24875857	0.01202	0.564325	0.572975	0.856256	chr3	183817230

cg26253500	-0.01746	-0.55236	0.581136	0.859963	chr7	136641740
cg12068949	-0.01759	-0.54989	0.582827	0.860802	chr1	159506033
cg15068527	0.016212	0.548562	0.583734	0.861267	chr3	183817134
cg01920563	-0.01688	-0.54811	0.584043	0.861463	chr7	136648231
cg02579332	-0.01459	-0.5474	0.584529	0.861701	chr7	154875967
cg20678835	0.014486	0.540764	0.589091	0.863915	chr1	23521332
cg22368476	0.014586	0.534778	0.593219	0.865806	chr11	123814163
cg12452364	-0.02093	-0.52377	0.600845	0.869208	chr20	60795156
cg02701826	-0.01355	-0.51471	0.607155	0.872222	chr19	15903794
cg01281175	-0.02543	-0.50887	0.611238	0.87396	chr5	175109114
cg00120810	-0.01163	-0.49961	0.617732	0.876587	chr3	11195317
cg12296860	-0.0103	-0.49452	0.621319	0.878157	chr11	46409428
cg05596267	-0.01542	-0.48879	0.625367	0.879751	chr15	34331198
cg01953456	-0.01124	-0.4774	0.633443	0.883213	chr3	183817976
cg00307530	-0.01162	-0.47734	0.633482	0.883216	chr5	147834517
cg19116351	0.022968	0.477152	0.633618	0.883246	chr3	88031048
cg11158819	0.017608	0.471028	0.63798	0.884972	chr3	183817853
cg10538202	0.017519	0.449283	0.653568	0.891182	chr7	154863338
cg00987015	-0.01052	-0.44617	0.655811	0.892092	chr11	62688751
cg00783712	0.012288	0.444628	0.656925	0.892554	chr1	239974513
cg16057587	0.010174	0.436256	0.66298	0.894916	chr7	136698566
cg06476131	0.023623	0.424548	0.671485	0.898441	chr13	47471052
cg01614101	0.013104	0.422022	0.673326	0.899148	chr11	113778771
cg17537380	0.007259	0.403523	0.686865	0.904393	chr6	87649596
cg07630532	0.011445	0.38468	0.70076	0.909741	chr20	60795459
cg03448301	0.01059	0.369405	0.7121	0.914422	chr11	62678778
cg16921789	-0.00765	-0.35665	0.721618	0.917723	chr3	88031773
cg01751188	0.008396	0.348618	0.727633	0.919936	chr22	23412225
cg05680531	0.016246	0.342313	0.732367	0.921704	chr7	136553327
cg23343875	0.015531	0.340449	0.733768	0.922306	chr19	16058370
cg16873130	-0.01379	-0.33598	0.737132	0.923465	chr7	136586950
cg24661173	-0.00987	-0.3336	0.738929	0.924028	chr7	154866132
cg25843439	-0.0129	-0.32793	0.743204	0.925351	chr19	15852574
cg25276126	0.007625	0.32306	0.746886	0.926647	chr1	20005715
cg01459748	0.013508	0.317343	0.751215	0.928257	chr11	113817020
cg01296705	0.00707	0.314738	0.75319	0.929096	chr5	175108269
cg15484742	0.01136	0.298133	0.765819	0.933176	chr1	23519884
cg01812577	0.006857	0.297513	0.766291	0.933335	chr11	62689122
cg13690703	-0.01009	-0.29229	0.770278	0.934608	chr3	88030644
cg09623773	0.014384	0.292186	0.770356	0.934628	chr7	154863381
cg01625621	-0.00657	-0.28797	0.773575	0.935892	chr15	34260433
cg03472798	-0.00695	-0.28242	0.777824	0.937264	chr6	87646930
cg18371750	-0.00653	-0.27444	0.783947	0.939451	chr5	175112799
cg17200850	-0.00766	-0.26859	0.78844	0.940895	chr5	175107097
cg01274715	0.008291	0.258152	0.796475	0.943517	chr10	92618033
cg02517524	0.006852	0.255186	0.798763	0.944339	chr22	23412814
cg06477056	0.007394	0.252152	0.801105	0.945058	chr5	175110609
cg26153642	0.007056	0.248851	0.803655	0.945969	chr3	183818368

cg18190847	-0.00617	-0.23974	0.810702	0.948327	chr3	11195751
cg06251978	-0.00808	-0.23501	0.814373	0.949532	chr1	159507078
cg26864526	0.005136	0.230712	0.817704	0.950635	chr3	11178064
cg12749468	-0.00846	-0.22908	0.818971	0.950937	chr3	183755507
cg17850597	0.006265	0.206643	0.836436	0.95608	chr19	15917833
cg27230009	-0.00358	-0.20635	0.836667	0.956117	chr3	11241399
cg18271969	0.005578	0.203215	0.839112	0.956912	chr3	183771499
cg12580770	0.010263	0.199568	0.84196	0.957712	chr7	154861569
cg07826387	0.004155	0.195766	0.844932	0.958619	chr1	240072501
cg12775613	-0.00591	-0.19411	0.846231	0.958856	chr3	88040034
cg26135506	0.009884	0.191806	0.84803	0.959356	chr10	92617562
cg07318372	0.004846	0.186414	0.852253	0.960556	chr1	240071102
cg05122082	0.015664	0.180582	0.856824	0.961715	chr14	20710905
cg17405853	-0.00402	-0.17188	0.863653	0.963824	chr5	175084085
cg12198176	0.003471	0.167257	0.867286	0.96496	chr22	23415316
cg11131902	-0.00454	-0.15879	0.87395	0.96696	chr5	175084710
cg14841965	0.004338	0.158275	0.874352	0.967043	chr11	123814849
cg11514288	0.004931	0.144558	0.885162	0.970122	chr13	47471197
cg19045531	-0.00418	-0.13681	0.891276	0.971812	chr19	15919022
cg02027079	-0.0054	-0.13494	0.892755	0.972201	chr13	47471705
cg11811391	0.004932	0.131723	0.895296	0.972826	chr1	23520083
cg24137472	-0.00377	-0.1264	0.899504	0.973889	chr14	20710881
cg21566860	-0.00268	-0.1184	0.905832	0.975658	chr3	11242797
cg02236913	-0.00283	-0.11781	0.906299	0.97572	chr1	20005598
cg01192538	-0.00241	-0.11589	0.907822	0.976025	chr13	47472050
cg27551227	-0.00584	-0.11518	0.908384	0.976216	chr7	154877217
cg23720528	-0.00266	-0.11233	0.910639	0.976963	chr10	92501798
cg03737442	0.002845	0.110992	0.9117	0.977105	chr3	11177295
cg01620540	0.003062	0.109497	0.912885	0.977367	chr13	47472064
cg15625631	0.003725	0.095971	0.923611	0.980288	chr11	123814972
cg10685228	0.00218	0.092904	0.926045	0.98094	chr3	183750284
cg08417719	-0.00197	-0.09041	0.928021	0.981478	chr11	123814620
cg10953410	-0.00294	-0.08404	0.933081	0.982888	chr11	113846918
cg22614355	-0.00197	-0.07288	0.941953	0.985122	chr1	19991237
cg00967901	0.00317	0.070201	0.944083	0.985787	chr10	92617915
cg24714094	0.001824	0.066405	0.947102	0.98666	chr22	23467005
cg10772974	0.001526	0.06541	0.947893	0.986859	chr15	34332433
cg12528649	0.00165	0.05563	0.955676	0.989023	chr11	46407116
cg16188532	-0.00277	-0.05469	0.956424	0.989159	chr13	47471090
cg04434491	-0.00289	-0.05313	0.957665	0.989485	chr19	16058640
cg04315863	-0.00111	-0.04472	0.96436	0.991024	chr15	34330588
cg04042861	-0.00137	-0.04116	0.967195	0.991773	chr2	231989824
cg17578539	-0.00099	-0.04099	0.96733	0.991819	chr5	175104957
cg11553153	0.000835	0.031216	0.975119	0.993765	chr15	34348521
cg17405012	0.00067	0.0159	0.987325	0.996789	chr7	136553263
cg12816057	0.000223	0.007711	0.993853	0.998554	chr5	148034206
cg09666573	8.04E-05	0.003243	0.997415	0.999439	chr3	11267627
